# Preparation of 2-phospholene oxides by the isomerization of 3-phospholene oxides

**DOI:** 10.3762/bjoc.16.75

**Published:** 2020-04-22

**Authors:** Péter Bagi, Réka Herbay, Nikolett Péczka, Zoltán Mucsi, István Timári, and György Keglevich

**Affiliations:** 1 Department of Organic Chemistry and Technology, Budapest University of Technology and Economics, Műegyetem rkp. 3., H-1111 Budapest, Hungary; 2 Femtonics Ltd., H-1094 Budapest, Hungary; 3 Department of Inorganic and Analytical Chemistry, University of Debrecen, H-4032 Debrecen, Hungary

**Keywords:** chlorophosphonium salts, isomerization, 2-phospholene oxides, 3-phospholene oxides, quantum chemistry

## Abstract

A series of 1-substituted-3-methyl-2-phospholene oxides was prepared from the corresponding 3-phospholene oxides by double bond rearrangement. The 2-phospholene oxides could be obtained by heating the 3-phospholene oxides in methanesulfonic acid, or via the formation of cyclic chlorophosphonium salts. Whereas mixtures of the 2- and 3-phospholene oxides formed, when the isomerization of 3-phospholene oxides was attempted under thermal conditions, or in the presence of a base. The mechanisms of the various double bond migration pathways were elucidated by quantum chemical calculations.

## Introduction

P-Heterocyclic derivatives are valuable targets in synthetic organophosphorus chemistry [1–4]. A heterocyclic ring containing a P atom appears in many popular P(III)-ligands, such as BPE, DuPhos, TangPhos, DuanPhos, ZhangPhos [5–11], among which a number of species may be considered as privileged ligands for transition metal-catalyzed enantioselective transformations [12]. Among the P-heterocycles with various ring size, the five-membered derivatives received special attention due to their optimal bond angles and shapes [13–14]. Besides their application as ligands [13,15], the five-membered heterocycles may also be valuable organocatalysts in reactions involving a P(III)–P(V) redox cycle, such as in catalytic Wittig-, aza-Wittig-, Staudinger-, Appel- and other reactions [16–21]. The ring strain of the four- and five-membered derivatives makes them highly susceptible to deoxygenation, thus they are ideal organocatalysts in P(III)–P(V) redox-coupled transformations [18,22–23]. Polycyclic compounds incorporating a P-heterocyclic moiety are of special importance due to their optoelectronic properties and applications [24–26]. Moreover, a few biologically active 5-membered P-heterocyclic derivatives are also known, which showed promising antitumor [27–30], antifungal [31–32] or GABA receptor antagonist activity [33].

**Figure 1:**
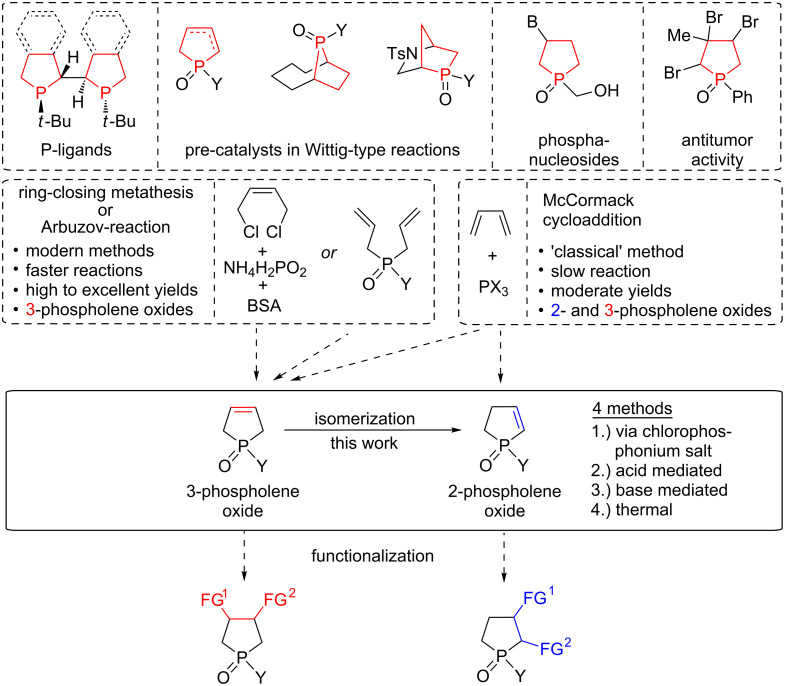
Examples for catalytically or biologically active molecules containing five-membered P-heterocyclic rings. General strategies for the preparation and functionalization of 2- and 3-phospholene oxides.

The most common synthesis of a given functionalized P-heterocyclic compound comprises the synthesis of the heterocyclic core, followed by its functionalization [27,29,34–35]. This latter step is of special importance for biologically active compounds, as specific functional groups are necessary to have a desired biological activity. Considering the functionalization of the five-membered P-heterocycles, the derivatives containing double bond(s) (i.e., phospholenes or phospholes) are of special importance, as a double bond may be functionalized by diverse reactions [36]. Considering the phospholenes, the position of the double bond (2- or 3-phospholenes) offers different possibilities for further transformations (Figure 1) [37–46]. Moreover, the presence of the double bond in the 2-phospholene derivatives is beneficial for having enhanced organocatalytic activity, in a few instances [47].

The McCormack cycloaddition is a key reaction for the preparation of the five-membered P-heterocyclic scaffold, as both the 2- or 3-phospholene core can be prepared depending on the butadiene derivative and P-reagent used [36,48–50]. However, the long reaction times, as well as the moderate or low yields make this approach less appealing. More recent syntheses of the phospholene oxides involve either a ring-closing metathesis [35,51–54], or a double Arbuzov reaction of 1,4-dichlorobut-2-ene and a silylated P-reagent [34–35,55]. However, these syntheses generally afford the corresponding 3-phospholene oxides, and only a few examples are known to provide the corresponding 2-phospholene derivatives [56–57], which raises the necessity of complementary synthetic strategies for 2-phospholene oxides (Figure 1).

A convenient strategy may involve the isomerization of 3-phospholene oxides to the corresponding 2-phospholene oxides. A few isomerizations resulting 2-phospholene derivatives have already been observed and published in the literature [58–61]. However, either the isomerization remained incomplete [58–59], or it was an undesired side-reaction caused by the high temperature and the excess of the reagent [62–64]. Our recent synthetic efforts were directed to the novel preparation of cyclic chlorophospholenium chlorides, and the utilization of these reactive derivatives in silane-free deoxygenations or dynamic resolutions [65–67]. In these studies, the isomerization of the 1-chloro-3-methyl-1-phenyl-3-phospholenium chloride to the corresponding 2-phospholenium salt was also observed as a side-reaction upon prolonged reaction times. It was also shown that the corresponding 2-phospholene oxides are the thermodynamically more stable isomers.

This paper seeks simple synthetic methods for the preparation of 2-phospholene oxides via the isomerization of the corresponding 3-phospholene derivatives. The isomerization reactions were carried out under thermal conditions, in the presence of acids or bases or via the formation of chlorophosphonium salts. In our study, the 3-methyl-3-phospholene oxides were considered as model compounds, the double bond rearrangement of a 3-phospholene oxide and a 3,4-dimethyl-3-phospholene oxide was also investigated. Moreover, we wish to interpret the different isomerization mechanisms by quantumchemical calculations.

## Results and Discussion

### Preparation of 1-substituted-3-methyl-2-phospholene oxides (**4**) via chlorophospholenium chlorides (**2** and **3**)

The preparation of 2-phospholene oxides **4** was first attempted via the formation of chlorophospholenium salts **2** and **3**. According to our procedure [65], the corresponding 1-substituted-3-methyl-3-phospholene oxide **1** was reacted with oxalyl chloride to give chloro-3-phospholenium salts **2** with complete conversion. In our experience, a synthetic protocol involving the use of oxalyl chloride in a slight excess, followed by the removal of the unreacted reagent under reduced pressure gave the corresponding chloro-3-methyl-3-phospholenium chlorides **2** in the most reproducible manner. The chloro-3-phospholenium salts **2** were then dissolved in chloroform, and were heated at 80 °C for 48 h. Upon this period, nearly complete isomerization of the chloro-3-phospholenium salts **2** to the corresponding chloro-2-phospholenium salts **3** occurred. The mechanism of this isomerization was investigated by quantumchemical calculations in our previous study [65]. However, the formation of the 2-phospholenium chlorides **3** was only postulated. Herein, two additional derivatives, the 4-methylphenyl- and ethyl-1-chloro-3-methyl-2-phospholenium chlorides **3c** and **3h** were isolated and characterized, which confirmed the key intermediate of the proposed mechanism. The chloro-2-phospholenium salts **3** were then hydrolyzed to give the corresponding 3-methyl-2-phospholene oxides **4** in an isomeric ratio above 97:3 and in yields of 71–96% (Table 1). If necessary, the traces of starting material **1** could be removed by simple column chromatography. Efficient chromatographic separation of 2- and 3-phospholene oxide isomers **4** and **1** could be accomplished in a mixture of acetone and 2-PrOH. The 2-phospholene oxides **4** obtained were characterized by spectroscopic methods (see Supporting Information File 1 for details).

**Table 1:** Preparation of 1-substituted-3-methyl-2-phospholene oxides **4** via chlorophospholenium chlorides (**2** and **3**).

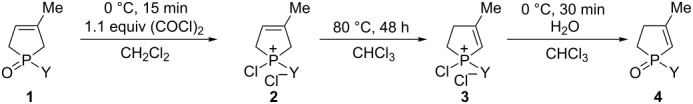			

Entry	Y	Yield (%)^a^	Ratio of **4**:**1** (%)^b^

1	Ph (**a**)	85	99:1
2	2-Me-C_6_H_4_ (**b**)	84	97:3
3	4-Me-C_6_H_4_ (**c**)	86	99:1
4	4-CF_3_-C_6_H_4_ (**d**)	89	98:2
5	4-MeO-C_6_H_4_ (**e**)	95	97:3
6	2,6-diMe-C_6_H_3_ (**f**)	96	99:1
7	1-naphthyl (**g**)	90	100:0
8	Et (**h**)	71	97:3
9	*n*-Pr (**i**)	75	99:1
10	*n*-Bu (**j**)	87	98:2
11	iBu (**k**)	87	99:1
12	iPent (**l**)	86	98:2

^a^Isolated yield of the mixture of **1** and **4**; ^b^determined by GC.

### Preparation of 3-methyl-2-phospholene oxides **4** under acidic conditions

As the next step of this study, we wished to find another isomerization pathways leading to the corresponding 2-phospholene oxides **4**, which does not require inert reaction conditions. Literature data indicated that such izomerizations may take place in the presence of acids [59,62,64]. 1-Phenyl-3-methyl-3-phospholene oxide (**1a**) was selected as the model compound, and a series of acids was tested. When phenylphospholene oxide **1a** was heated in aqueous HCl, or in the presence of *p*-toluenesulfonic acid in a toluene solution significant isomerization could not be observed. When 1-phenyl-3-phospholene oxide (**1a**) was refluxed in trifluoroacetic acid, a mixture containing the corresponding 2- and 3-phospholene oxides (**4a** and **1a**) in a ratio of 11:89 was obtained. However, when phospholene oxide **1a** was heated in neat methanesulfonic acid at 160 °C for 24 h, **4a** and **1a** in a ratio of 97:3 were obtained, but the yield of the mixture was 57% due to decomposition caused by the vigorous reaction conditions. Based on this promising preliminary result with MeSO_3_H, the effect of both the reaction temperature and the reaction time was investigated. It was found that complete isomerization to the 2-phospholene oxide derivative **4a** could not be reached with MeSO_3_H at 25 °C even after prolonged reaction times. Setting the reaction temperature to 50 °C and the reaction time to 60 h seemed to be the reasonable choice, as nearly the same isomeric composition (96:4) could be achieved as at 160 °C, but with significantly increased yield (up to 81%) (See Supporting Information File 1 for the optimization of the reaction conditions). Under these conditions, a series of 1-aryl- or 1-alkyl-3-methyl-3-phospholene oxides **1** was isomerized, and the products containing 2-phospholene oxides **4** in a range of 96–100% were obtained in yields of 81–96%. From the series of 3-phospholene oxides investigated, ethyl derivative **4h** was somewhat the exception, as the yield of the crude product was only 55%, which contained 92% of desired 2-phospholene derivative **4h**. This may be the consequence of a higher degree of decomposition occurring in case of this derivative (Table 2).

**Table 2:** Preparation of 1-substituted-3-methyl-2-phospholene oxides **4** in the presence of methanesulfonic acid.

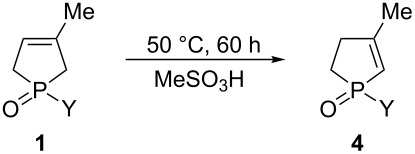			

Entry	Y	Yield (%)^a^	Ratio of **4**:**1** (%)^b^

1	Ph (**a**)	81	96:4
2	2-Me-C_6_H_4_ (**b**)	90	100:0
3	4-Me-C_6_H_4_ (**c**)	94	99:1
4	4-CF_3_-C_6_H_4_ (**d**)	88	98:2
5	4-MeO-C_6_H_4_ (**e**)	87	97:3
6	2,6-diMe-C_6_H_3_ (**f**)	94	99:1
7	1-naphthyl (**g**)	96	98:2
8	Et (**h**)	55	92:8
9	*n*-Pr (**i**)	85	98:2
10	*n*-Bu (**j**)	95	99:1
11	iBu (**k**)	86	97:3
12	iPent (**l**)	84	98:2

^a^Isolated yield of the mixture of **1** and **4**; ^b^determined by GC.

### Investigation of the isomerization of 3-phospholene oxides **1** to 2-phospholene oxides **4** under basic conditions

In the next stage, the base-mediated isomerization of 1-phenyl-3-methyl-3-phospholene oxide (**1a**) was also investigated. In the screening, several organic and inorganic bases, such as trimethylamine, *N*,*N*-diisopropylethylamine, pyridine, 4-dimethylaminopyridine, *n*-butyllithium, NaH and Cs_2_CO_3_ were tested. The initial experiments showed that practically no isomerization occurred when phenyl-3-phospholene oxide **1a** was refluxed in toluene in the presence of organic bases (see Supporting Information File 1 for the results). Whereas the decomposition of the starting material was observed, when phenyl-3-phospholene oxide **1a** was treated with *n*-butyllithium or NaH, as it was also observed by Stankevič et. al. [68] However, when phenyl-3-phospholene oxide was heated in the presence of 1 equiv of Cs_2_CO_3_ in toluene, the crude product obtained after the reaction contained the mixture of 1-phenyl-2-phospholene oxide **4a** and the corresponding 3-phospholene oxide **1a** in a 77:23 ratio. Based on this promising preliminary result, 1-ethyl-3-methyl-3-phospholene oxide (**1h**) was also included as a model compound for the alkyl derivatives. The isomerization of these two model compounds (**1a** and **1h**) was tested in the presence of several inorganic bases, such as Na_2_CO_3_, K_2_CO_3_, Cs_2_CO_3_, NaOH and NaOEt in toluene, DMF or DMSO, with or without a phase transfer catalyst. The results indicated that the ratio of the 2- and 3-phospholene oxide isomers (**4** and **1**) did not exceed a ratio of ca. 80:20 and 70:30 in case of the phenyl- and the ethyl derivative, respectively, despite the base, solvent and reaction temperature used. The fact that practically the same results were obtained with a wide variety of bases suggested that the base-mediated isomerization leads to a thermodynamic equilibrium between phospholene oxides **1** and **4** catalyzed by the base (see Supporting Information File 1 for the results). In order to test this hypothesis, the two model compounds (**1a** and **1h**) were refluxed with Cs_2_CO_3_ in DMF in the presence of TEBAC. The crude products obtained were reacted again two times under the same conditions. The results of these runs showed that the ratio of the corresponding 2- and 3-phospholene oxides (**4a** and **1a** or **4h** or **1h**) remained practically unchanged throughout the reaction sequence (Table 3). These experiments suggested that the base-mediated isomerization leads to an equilibrium, which is in accordance with other reports in the literature [42]. Thus, this base-mediated isomerization was not studied further with other 3-phospholene oxide derivatives **1**.

**Table 3:** Investigation of the isomerization of 1-phenyl- and 1-ethyl-3-methyl-3-phospholene oxides **1a** or **1h** to the corresponding 2-phospholene oxides **4** in the presence of Cs_2_CO_3_.

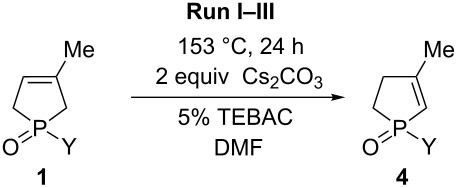				

Entry	Y	Run	Yield (%)^a^	Ratio of **4**:**1** (%)^b^

1	Ph (**a**)	I.	77	84:16
2	Ph (**a**)	II.	76	80:20
3	Ph (**a**)	III.	67	81:19
4	Et (**h**)	I.	68	66:34
5	Et (**h**)	II.	78	67:33
6	Et (**h**)	III.	73	65:35

^a^Isolated yield of the mixture of **1** and **4**; ^b^determined by GC.

### Isomerization of 3-phospholene oxides **1** to 2-phospholene oxides **4** under thermal conditions

The isomerization of 3-phospholene oxides **1** was also investigated under thermal conditions without using any reagent. In our initial attempts, 1-phenyl-3-methyl-3-phospholene oxide (**1a**) was refluxed in high boiling point solvents, such as toluene, DMF, or DMSO. However, the maximal content of phenyl-2-phospholene oxide **4a** in the crude product was only 4%. In contrast, when neat 3-phospholene oxide **1a** was heated to 200 °C without any solvent, the ratio of the 2- and 3-phospholene oxide **4a** and **1a** increased to 71:29 in the crude product after 24 h.

In order to study how the different substituents influence the rate of isomerization, a series of 1-substituted-3-methyl-3-phospholene oxides **1** was heated at 200 °C for 1 day in the absence of any solvent. The results indicated that the isomerization was not complete in any case even with prolonged reaction time, as the ratio of the 2- and 3-phospholene oxide isomers **4** and **1** was in the range of 43:57 and 60:40 for the alkyl derivatives (**4h–l** and **1h–l**), and in between 63:37 and 85:15 for the arylphospholene oxides (**4a–g** and **1a–g**). Due to the decomposition at the high temperature applied, yields of the crude products were moderate dropping in the range of 46–84% (Table 4). For the phenyl and the ethyl derivative (**1a** and **1h**), the reaction time was extended to 5 days. However, this extension did not influence the ratio of the 2- and 3-phospholene oxide isomers (**4** and **1**) significantly (see Supporting Information File 1 for the results).

**Table 4:** Isomerization of 3-phospholene oxides **1** to 2-phospholene oxides **4** under thermal conditions.

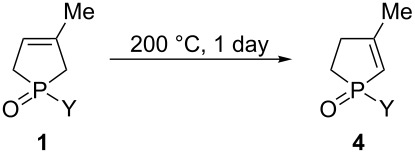			

Entry	Y	Yield (%)^a^	Ratio of **4**:**1** (%)^b^

1	Ph (**a**)	58	71:29
2	2-Me-C_6_H_4_ (**b**)	84	69:31
3	4-Me-C_6_H_4_ (**c**)	83	63:37
4	4-CF_3_-C_6_H_4_ (**d**)	81	72:28
5	4-MeO-C_6_H_4_ (**e**)	82	73:27
6	2,6-diMe-C_6_H_3_ (**f**)	75	85:15
7	1-naphthyl (**g**)	69	73:27
8	Et (**h**)	50	55:45
9	*n*-Pr (**i**)	75	52:48
10	*n*-Bu (**j**)	68	49:51
11	iBu (**k**)	76	60:40
12	iPent (**l**)	46	43:57

^a^Isolated yield of the mixture of **1** and **4**; ^b^determined by GC.

### Comparison of the isomerization of 1-phenyl-3-phospholene oxide (**5**), 1-phenyl-3-methyl-3-phospholene oxide (**1a**) and 1-phenyl-3,4-dimethyl-3-phospholene oxide (**8**) under the optimized conditions

**Scheme 1:**
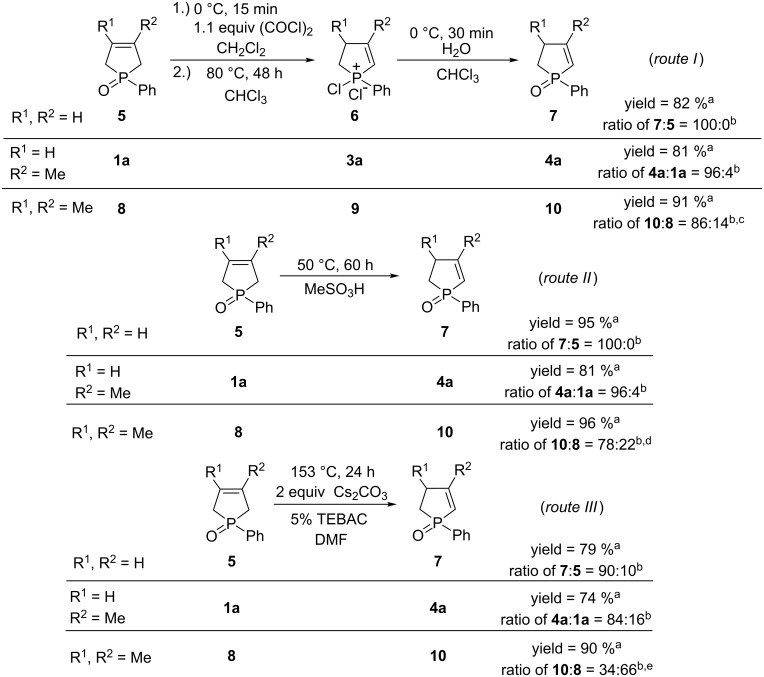
Comparison of the isomerization of 1-phenyl-3-phospholene oxide (**5**), 1-phenyl-3-methyl-3-phospholene oxide (**1a**) and 1-phenyl-3,4-dimethyl-3-phospholene oxide (**8**). ^a^Isolated yield of the mixture of **8**–**10**, **1a**–**4a**, **5**–**7,** respectively. ^b^Determined by GC. ^c^Ratio of *trans–cis* isomers of **10**: 44:56. ^d^Ratio of *trans–cis* isomers of **10**: 45:55. ^e^Ratio of *trans–cis* isomers of **10**: 40:60.

As the last step of this study, it was investigated, how the methyl substituents on the P-heterocyclic core influence the outcome of the isomerization. Methyl group(s) in positon 3 and 4 represents a typical substitution pattern. Thus, the isomerization of 1-phenyl-3-phospholene oxide (**5**) and 1-phenyl-3,4-dimethyl-3-phospholene oxide (**8**) was investigated under the optimized conditions via the formation of chlorophospholenium salts (Scheme 1, route I), or using MeSO_3_H or Cs_2_CO_3_ (Scheme 1, routes II or III). These results were compared with those obtained for the 1-phenyl-3-methyl-3-phospholene oxide (**1a**). Scheme 1 shows that, considering all reaction pathways, the highest extent of isomerization was observed for 1-phenyl-3-phospholene oxide (**5**). Pure phenyl-2-phospholene oxide (**7**) could be prepared using MeSO_3_H, or via the formation of chlorophospholenium chloride (**6**), which indicates that these two routes may be applicable for the isomerization of other 1-substituted-3-phospholene oxides, as well. As it was expected, the lowest level of isomerization was observed in the presence of Cs_2_CO_3_, as the crude products contained the phenyl-2- and 3-phospholene oxides (**7** and **5**) in a ratio of 90:10.

Considering the mono- and dimethyl-3-phospholene oxides **1a** and **8**, the ratio of the corresponding 2-phospholene oxides **4a** or **10** was in the range of 84–96% for the 3-methyl-, and in the range of 34–86% for the 3,4-dimethyl derivative indicating that the additional methyl groups at position 3 or 4 hinder the migration of the double bond.

It is noteworthy, that in case of 1-phenyl-3,4-dimethyl-3-phospholene oxide (**8**), the isomerization involves the formation of an additional stereogenic center and the corresponding 2-phospholene oxide (**10**) was obtained as a mixture of *cis*- and *trans*-diastereomers. The ratio of these two diastereomers was in the range of 60:40 to 55:45. The *cis*- and *trans*-diastereomers (*cis*- and *trans*-**10**) were separated by column chromatography, and they were identified by nuclear Overhauser effect (NOE) NMR spectroscopy. In the ROESY spectrum of the *cis*-diastereomer (*cis*-**10**), the phenyl ^1^H resonances give NOE/ROE crosspeaks with the C(4)H and one of the C(5)H_2_^1^H resonances. By contrast, in the spectrum of *trans*-diastereomer (*trans*-**10**), phenyl ^1^H resonances give NOE/ROE crosspeaks with C(4)-methyl and the other one of the C(5)H_2_^1^H signals (see Supporting Information File 1 for details). Thus, based on the distinctive NOE patterns of phenyl resonances, the two diastereomers (*cis*- and *trans*-**10**) could be unambiguously identified.

### Theoretical aspects of the isomerization of the 3-phospholene oxides **1** to 2-phospholene oxides **4**

#### Thermodynamic description

The different isomerization pathways leading to the corresponding 2-phospholene oxides **4** were also investigated by quantum chemical calculations at MP2/6-31++G(d,p) and MP2/6-311++G(2d,2p) levels of theory, including the PCM solvent model with the parameters of THF. For these investigations, the 1-phenyl or 1-ethyl-3-methylphospholene oxides (**1a** or **1h**) were considered as model compounds, but phospholene oxides incorporating substituted phenyl moiety (**1c–f**) were also calculated in a few instances. 1-Phenyl-3-phospholene oxide (**5**) and 1-phenyl-3,4-dimethyl-3-phospholene oxide (**8**) were also included in our calculations to interpret how the substituents on the P-heterocyclic ring affect the extent of the isomerization.

The conversion of 3-phospholene oxides **1** to the corresponding 2-phospholene derivatives **4** was first interpreted by means of thermodynamic data, and the olefinicity concept [69–72]. The calculations were performed at a high MP2/6-311G++(2d,2p) level of theory (Table 5). The reaction enthalpies (Δ*H*), as well as Gibbs free energies (Δ*G*) of the **1**→**4** transformation exhibited typically low values, and these reactions can be considered as nearly thermoneutral processes. Interestingly, the aromatic derivatives **1a** or **1c–f** prefer more the formation of the 2-phospholene oxide isomer (**4a** or **4c–f**, Table 5, entries 3–7). In contrast, there is no benefit for the ethylphospholene oxides (**1h** or **4h**) (Table 5, entry 8). The olefinicity concept and the olefinicity value (OL%) was developed to measure and describe quantitatively the degree of conjugation of an alkene group with the neighboring functional groups relative to ethylene (0%) and allyl anion (100%) as references on a linear scale. Large values mean a high degree of conjugations, while low values refer to a rather isolated double bond. The calculated olefinicity values (OL%) for 3- and 2-phospholene oxides **1** and **4** are within a narrow range, between 29–35%, and the change during the isomerization of **1**→**4** also confirms the thermodynamic data. When 3-phospholene oxide **1** exhibits a lower OL%, it is transformed in an exothermic fashion to the corresponding 2-phospholene oxide **4** having a higher OL% value. The phospholene oxides with aromatic substituents exhibit slightly higher OL% (ca*.* 4%) towards 2-phospholene oxide **4** than to the corresponding 3-phospholene isomer **1** (Table 5, entries 3–7). In contrast, the difference of the OL% values is almost negligible for the ethyl-3- or 2-phospholene oxides **1h** or **4h** (Table 5, entry 8). Both the thermodynamic data and the OL% values indicate that the additional methyl groups on the phospholene ring also influence the ratio of the corresponding 2- or 3-phospholene oxides **4** or **1**. The calculations suggest that the 3-phospholene oxide isomer **8** is the most stable form in case of the phenyl-3,4-dimethylphospholene oxide, while the corresponding 2-phospholene oxides **4a** or **7** are the preferred isomers for the unsubstituted or methyl-substituted phospholene oxide derivatives (Table 5, entries 1–3). According to the thermodynamic data without the consideration of the reaction mechanisms and transition states (Table 5), one may predict a well-balanced equilibrium between the 2- or 3-phospholene oxides **4** or **1** in a final state. The experimental results obtained under basic or thermal conditions support this equilibrium concept. In contrast, nearly complete isomerization was observed via the formation of chlorophospholenium chlorides or in the presence of methanesulfonic acid, which may indicate that the intermediates formed over the course of the reaction may perturb the thermodynamic equilibrium of the 2- or 3-phospholene oxides **4** or **1**. It was shown in our previous study [65] that the relative stability shifts towards the 2-phospholene isomer, when chlorophospholenium salts are involved, and it is the underlying reason why the 2-phospholene oxides **4** can be prepared effectively by this reaction. Under strong acidic conditions, the P=O moiety tends to be protonated, which may be responsible for the complete isomerization in the presence of methanesulfonic acid. The thermodynamic data and the olefinicity values (OL%) calculated for the corresponding protonated derivatives **1+H^+^** and **4+H^+^** showed that indeed this protonation perturbs the original equilibrium significantly. The protonated 2-phospholene oxide **4+H^+^** became the most preferred isomer against protonated 3-phospholene oxide **1+H^+^** with the isomeric ratio exceeding 90:10 (Table 5, entries 12–16.). The aqueous work-up of the reaction mixture is an irreversible process, and it freezes the results of the acidic equilibrium of (**1+H^+^**)→(**4+H^+^**), providing the corresponding 2-phospholene-oxides **4** with good yields, despite the original enthalpy differences of **1** and **4**. The calculations also showed that additional methyl groups on the P-heterocyclic ring influence the acid mediated isomerization (**4** or **1**, Table 5, entries 9–11.). The 2-phospholene oxides (**4a** or **7**) are the preferred isomers for the unsubstituted and methyl-substituted phospholene core. In contrast, the calculations predicted a 73:27 ratio for the dimethyl-substituted 2- and 3-phoshopholene oxides (**10** and **8**) (Table 5, entry 10). These predictions showed strong correlation with the experimental results.

**Table 5:** Reaction enthalpies (Δ*H* in kJ mol^−1^) of the isomerization processes of **1**→**4** and (**1+H^+^**)→(**4+H^+^**) at MP2/6-311G++(2d,2p) level of theory. Their calculated olefinicity values (OL%) are also given in percentages at the same level of theory.

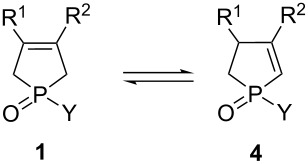									

	R^1^	R^2^	**Y**	Entry	Δ*H* (**1**→**4**)	Δ*G* (**1**→**4**)	**1**/**4** ratio	OL%(**1**) OL%(**4**)	ΔOL%

	H	H	Ph	1	−1.7	0.2	51:49	31.6% 29.8%	−2.1%
	Me	Me	Ph	2	9.5	6.1	83:17	31.0% 27.3%	−3.7%
**a**	H	Me	Ph	3	−4.7	−2.5	35:65	31.9% 36.1%	+4.2%
**c**	H	Me	4-Me-C_6_H_4_	4	−4.3	−2.5	35:65	32.3% 36.3%	+4.0%
**f**	H	Me	4-OMe-C_6_H_4_	5	−4.2	−3.5	29:71	32.5% 36.3%	+3.8%
**e**	H	Me	4-CF_3_-C_6_H_4_	6	−4.5	−3.6	28:72	32.5% 36.7%	+4.2%
**g**	H	Me	2,6-diMe-C_6_H_3_	7	−4.1	−5.1	21:79	31.5% 35.2%	+4.1%
**h**	H	Me	Et	8	+2.0	−0.1	51:49	31.6% 29.8%	−1.8%

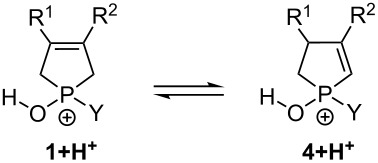									

	R^1^	R^2^	**Y**	Entry	Δ*H*(**1+H^+^** →**4+H^+^**)	ΔG(**1+H^+^** →**4+H^+^**)	**1+H^+^**/ **4+H^+^** ratio	OL%(**1+H^+^**) OL%(**4+H^+^**)	ΔOL%

	H	H	Ph	9	−11.3	−11.1	6:94	54.1% 66.2%	+12.1%
	Me	Me	Ph	10	−7.9	−4.0	27:73	53.8% 67.1%	+13.3%
**a**	H	Me	Ph	11	−12.4	−12.6	4:96	55.4% 69.1%	+13.7%
**c**	H	Me	4-Me-C_6_H_4_	12	−10.6	−10.9	6:94	55.7% 69.2%	+13.5%
**f**	H	Me	4-OMe-C_6_H_4_	13	−10.1	−10.3	7:93	55.0% 69.5%	+14.5%
**e**	H	Me	4-CF_3_-C_6_H_4_	14	−13.7	−13.6	3:97	55.1% 68.1%	+13.0%
**g**	H	Me	2,6-diMe-C_6_H_3_	15	−9.3	−9.9	7:93	54.4% 66.9%	+12.5%
**h**	H	Me	Et	16	−8.8	−9.2	9:91	52.8% 58.2%	+5.4%

#### Mechanistic investigations of the isomerization of 3-phospholene oxides **1** under thermal conditions

**Scheme 2:**
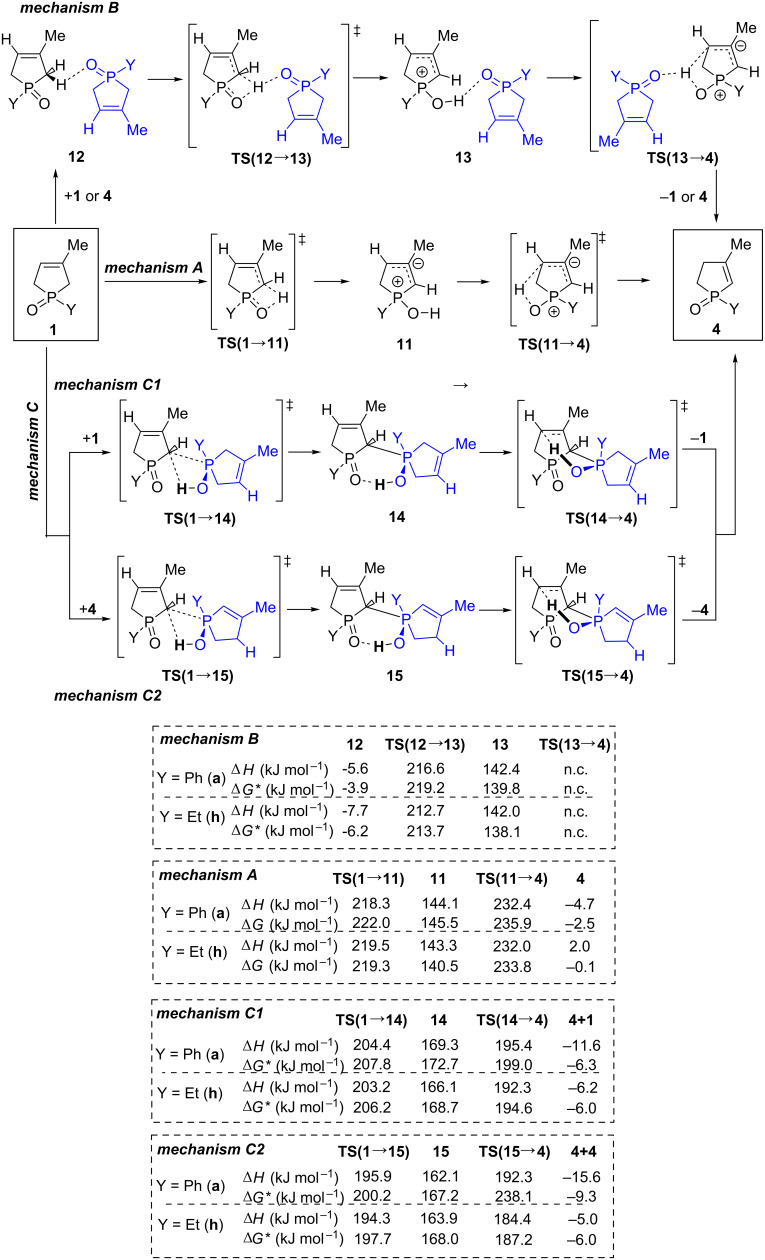
Three possible reaction mechanisms considered in the theoretical studies for the isomerization of 3-phospholene oxides **1** under thermal conditions. *The calculated Δ*G* values for mechanism B and C were compensated by the dimerization entropy (ca. 128 J mol^−1^ K^−1^) due to the fact that the entropy obtained in vacuo calculations are not realistic in condensed phase.

For the isomerization under thermal conditions, three mechanisms (mechanism A, B or C in Scheme 2) were outlined and examined by theoretical methods. Mechanism A involves a monomolecular rearrangement, involving zwitterionic structure **11** via high energy and a four-membered ring transition state [**TS**(**1→11**)]. This stressed structure has a rigid, entropically unbeneficial transition. The second transition state [**TS**(**11→4**)] represents a significantly lower energy gap with a more comfortable five-membered ring. As the thermal rearrangement was only feasible under solvent-free conditions, one may assume mechanism B, which involves the formation of phospholene oxide **1** dimers, and the second molecule only assists the rearrangement similarly to mechanism A. However, according to computations, in this arrangement the neighboring P=O group has only marginal beneficial effect on the activation enthalpies, compared to mechanism A. In the third possibility (mechanism C1), the proton from the C(2)H_2_ moiety can migrate to the P=O group in the second molecule, forming quasi-deprotonated (anionic) and protonated (cationic) molecules [**TS**(**1**→**14**)]. However, this arrangement is not stable, and a stabilized homodimer with a pentavalent P atom **14** is formed immediately. This quasi-autocatalytic process decreases significantly the enthalpy demand of the TS to a reliable level, and finally it gives the corresponding 2-phospholene oxide **4**. More interestingly, mechanism C2 shows that the enthalpy level of the process decreases by 7–10 kJ mol^−1^ if a heterodimer (**1**–**4**) is formed instead of a homodimer (**1**–**1**). Thus, the 2-phospholene oxide **4** can enhance the rate of the isomerization by 6 times at temperature of the reaction (570 K).

**Figure 2:**
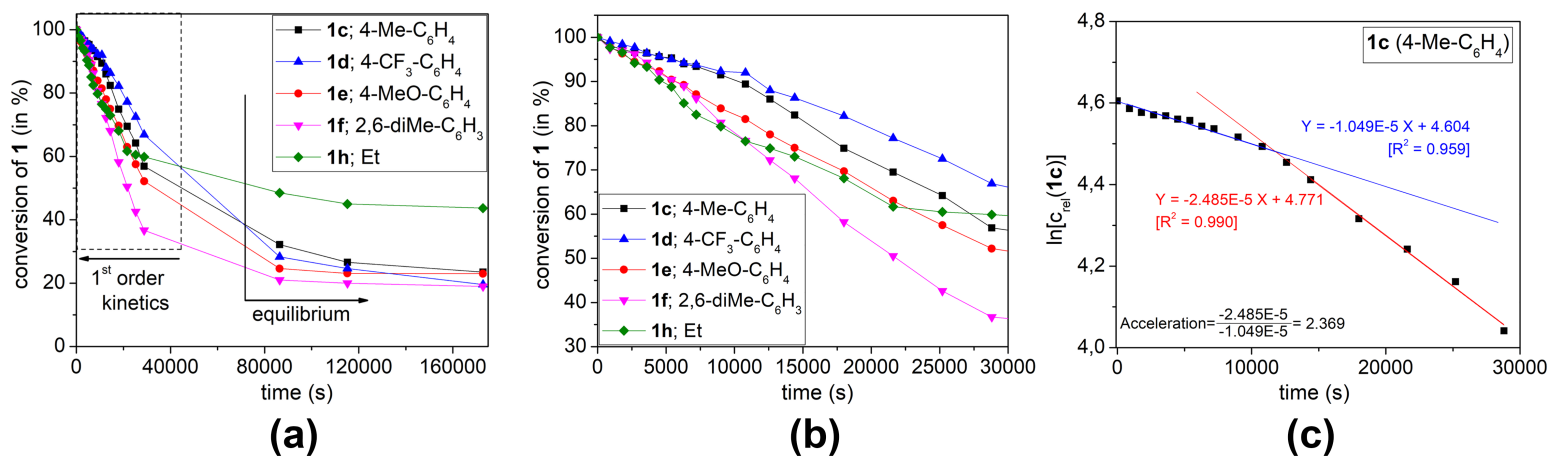
The full time experimental kinetic curves (a); The initial part of the kinetic curves of **1c**–**f** and **1h** (b); Initial and second fittings on the kinetic curve of **1c** (c). For more kinetic curves, see Supporting Information File 1, Figure S1).

In order to verify the computed reaction mechanism of the 3-phospholene oxide **1**–2-phospholene oxide **4** rearrangement, standard kinetic experiments were run for the substituted aryl- or ethyl-3-methylphospholene oxides (**1c–f** or **1h**) at a constant temperature. The reactions were carried out at 200 °C in neat, and the progression of the reactions was followed by gas chromatography for two days using biphenyl as internal standard. According to repeated experiments, the errors in measured relative concentrations were below 5%. The concentration curves undoubtedly refer to an equilibrium reaction, reaching a final concentration of **1** and **4** (Figure 2a). The results of this kinetic study indicated that the electron-donating groups on the phenyl ring accelerated, while the electron-withdrawing groups decelerated the isomerization in the first few hours.

All thermal isomerization processes lead to an equilibrium (Figure 2a). The mathematical analysis of the initial part of these kinetic curves resulted in a first order-like mechanism in contrast to other assumptions (e.g., second order) (Figure 2b). Interestingly, the later points started deviating from the initial linear fitting on the graph after the conversion of 3-phospholene oxides **1c**–**f** or **1h** reached the 15–20% (after 10 000 s). After 5 h (>20 000 s), the reaction rates of the first order approximation increased in all cases, and these values were doubled for phospholene oxides **1c**, **1d** and **1f** (Figure 2c and Supporting Information File 1). This acceleration observed in the isomerization of the phospholene oxides is in accordance with the proposed mechanism (mechanism C2, Scheme 2), which predicted that the 2-phospholene oxides **4** can enhance the rate of the isomerization. Thus, the increasing amount of the corresponding 2-phospholene oxide **4** takes a more and more dominant role as the catalysis of the 3-phospholene oxide (**1**) 2-phospholene oxide (**4**) rearrangement, which explains the change in the reaction rate in the kinetic experiments.

#### Mechanistic studies of the isomerization of 3-phospholene oxides **1** under acidic conditions

**Scheme 3:**
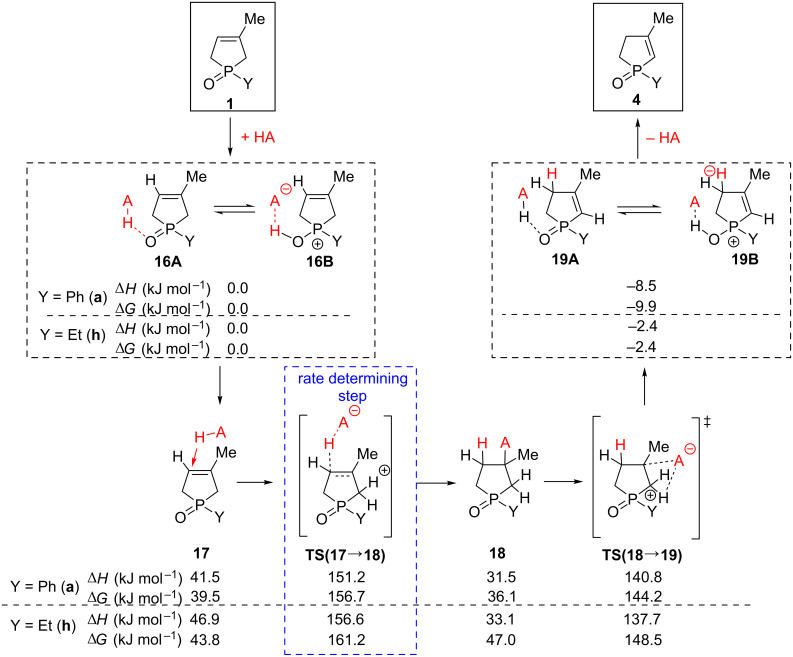
Computed reaction mechanism of the 3-phospholene oxide (**1**) 2-phospholene oxide (**4**) isomerization under acidic conditions at MP2/6-311++G(2d,2p)//PCM(THF), including implicit solvent model.

The isomerization of 3-phospholene oxides **1** also proceeds in the presence of strong acids, such as methanesulfonic acid. On the one hand, the reaction rate increased in the presence of MeSO_3_H, on the other hand, the reaction mixture contained a much higher amount of the 2-phospholene oxide **4**, as compared to the thermal- or base-catalyzed reactions. Scheme 3 shows the computed reaction mechanism of the acidic transformation, which starts from the complex formed from MeSO_3_H and the 3-phospholene oxide **16A**, involving in a protonation equilibrium with **16B**. In the next step, the acid migrates to position C(4), and the olefinic carbon atom can be easily protonated via a high enthalpy transition state [**TS(17**→**18)**], which is the rate determining step of this reaction sequence. We suppose that this rather high enthalpy is much lower due to the tunneling effect of the proton, whose energy gap is estimated to be reduced to about 130 kJ mol^−1^ in an optimal arrangement of the proton [73–74]. After the transition state, the mesylate anion attacks consecutively the position C(3), forming a neutral, relatively low-enthalpy intermediate **18**. The departure of the mesylate anion from position C(3) attracts the proton from C(2) via a lower-enthalpy transition state [**TS**(**17**→**18**)] yielding the expected product, complexed by the acid **19A**. Considering the equilibrium only between **16A–B** and **19A–B**, the product side is already shifted by −8.5 kJ mol^−1^ or −2.4 kJ mol^−1^, which makes the formation of **4a** or **4h** a beneficial process, meaning a shifted equilibrium towards the corresponding 2-phospholene oxide (**4a** or **4h**). Comparing the calculations for the phenyl- and ethylphospholene derivatives, one can conclude that there is no significant difference in the reaction enthalpies for the aryl- and alkylphospholene oxide models.

#### Mechanistic investigations of the isomerization of 3-phospholene oxides **1** under basic conditions

**Scheme 4:**
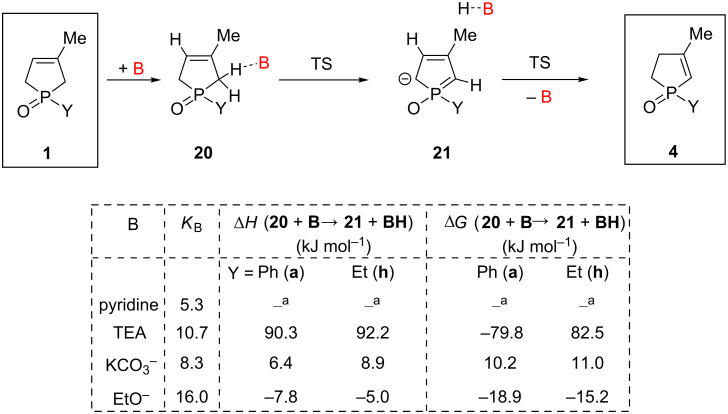
Computed reaction mechanism of the 3-phospholene oxide (**1**) 2-phospholene oxide (**4**) isomerization under basic conditions computed at MP2/6-311G(2d,2p) level of theory, including implicit solvent model.

The calculations showed that the reaction mechanism follows a simple sequence under basic conditions (Scheme 4). In the first step the base makes the reactant–base complex **20**, and the consequent deprotonation occurs at position C(2), which is more favorable than the same process at position C(5) (by ca. 3–4 kJ mol^−1^). An anionic intermediate **21** is formed in this manner, and the related transition state represents a low enthalpy barrier (ca. 55 kJ mol^−1^), which is lowered by the tunneling effect of the proton. In the final step, the protonated base gives back the proton to the position C(4) also via a transition state with a low enthalpy barrier (ca. +20 kJ mol^−1^) yielding the corresponding 2-phospholene oxide **4** The calculations showed that the base does not affect the enthalpy difference between the 3- and 2-phospholene oxides **1** and **4** maintaining the thermodynamic equilibrium, it has only an accelerating catalytic effect on the reaction rate. Consequently, the expected ratio of the 2- and 3-phospholene oxides **4** and **1** is similar to the results obtained under thermal conditions. These theoretical findings are in accordance with the experimental results. Interestingly, the strength of the base applied may change the reaction rate significantly, as the reaction enthalpy of the intermediate **21** changes compared to **20**. Pyridine is not able to take over the proton from the molecule (the structure **20** is not a minimum on the potential hypersurface (PES)). Triethylamine can initiate the deprotonation, but the reaction enthalpy towards **21** is very endothermic, making the reaction rate negligible, as observed in the experiment. In the case of KCO_3_^−^ (instead of Cs_2_CO_3_), the deprotonation proceeds smoothly with almost a thermoneutral fashion, predicting a significant improvement in the reaction rate. According to the estimated enthalpy value of the TS for **20**→**21**, one may expect a relatively fast process with Cs_2_CO_3_ even at moderate temperatures, however, the poor solubility of the inorganic salt demands a higher reaction temperature. Finally, the deprotonation by NaOEt is already a slightly exothermic process, suggesting a fast transformation.

## Conclusion

In this comprehensive study, the isomerization of 3-phospholene oxides **1** to the corresponding 2-phospholene oxides **4** was investigated. Complete isomerization took place either in the presence of methanesulfonic acid, or via the formation of chlorophospholenium salts (**2** and **3**), and a series of 1-substituted-3-methyl-2-phospholene oxides **4** were prepared. It was also shown that the isomerization may occur to some extent in the presence of inorganic bases, or at elevated temperatures. However, the isomerization remained incomplete under these conditions. It was found that methyl group(s) at positon 3 or 4 in the P-heterocyclic ring hinder the isomerization in all reaction pathways investigated. The mechanisms of acid- or base-mediated, as well as thermal isomerizations were elucidated by quantum chemical calculations, and the results justified why a given isomerization pathway leads to an equilibrium or proceeds until completion.

McCormack cycloaddition is still an established method for the synthesis of both 2- or 3-phospholene oxides. However, our paper outlined that the preparation of 2-phospholene oxide derivatives **4** is also feasible from 3-phospholene oxides **1** via the formation of chlorophosphonium salts or in the presence of MeSO_3_H. These isomerization reactions may be regarded as complementary procedures for the more recent preparation of the five-membered heterocyclic core, which generally affords the corresponding 3-phospholene oxide isomers **1**. Our study also showed that high temperature or basic conditions should also be taken into consideration, when new or even existing reactions of 3-phospholene oxides **1** are carried out, in order to avoid the undesired isomerization, and thus diminished yields.

## Experimental

**General procedure for the preparation of 2-phospholene oxides 4, 7 or 10 via the formation of chlorophospholenium chlorides 3, 6 and 9:** To the solution of 1.0 mmol of 3-phospholene oxide (**1a**: 0.19 g, **1b**: 0.21 g, **1c**: 0.21 g, **1d**: 0.26 g, **1e**: 0.22 g, **1f**: 0.22 g, **1g**: 0.24 g, **1h**: 0.14 g, **1i**: 0.16 g, **1j**: 0.17 g, **1k**: 0.17 g, **1l**: 0.19 g, **5**: 0.18 g, **8**: 0.21 g) in 1.0 mL of dichloromethane, 0.094 mL (1.1 mmol) of oxalyl chloride was added dropwise at 0 °C in a sealed tube. The reaction mixture was stirred for 15 minutes, then the volatiles were evaporated to remove the solvent and the excess of the oxalyl chloride. The cyclic halophosphonium salt thus prepared was dissolved in 1.0 mL of chloroform, and the solution was stirred at 80 °C for 2 days. The reaction mixture was hydrolyzed with 1.0 mL of water. The phases were separated, and the aqueous phase was extracted with 5 × 1.0 mL of dichloromethane. The organic phases were combined, washed with 2.0 mL of saturated NaHCO_3_, dried (Na_2_SO_4_) and evaporated. The crude reaction mixture was filtered through a plug of silica with 3% methanol in dichloromethane to give the corresponding 2-phospholene oxide (**4**, **7** or **10**). The results are summarized in Table 1 and Scheme 1.

**General procedure for the preparation of 2-phospholene oxides (4, 7 or 10) using methanesulfonic acid:** 1.0 mmol of the 3-phospholene oxide (**1a**: 0.19 g, **1b**: 0.21 g, **1c**: 0.21 g, **1d**: 0.26 g, **1e**: 0.22 g, **1f**: 0.22 g, **1g**: 0.24 g, **1h**: 0.14 g, **1i**: 0.16 g, **1j**: 0.17 g, **1k**: 0.17 g, **1l**: 0.19 g, **5**: 0.18 g, **8**: 0.21 g) was dissolved in 1.0 mL of methanesulfonic acid, and the reaction mixture was heated at 50 °C for 60 h. The reaction mixture was then cooled to 0 °C, and 2.0 mL of water was added. Then, the reaction mixture was neutralized with saturated NaHCO_3_ at 0 °C. The solution was extracted with 5 × 3.0 mL dichloromethane. The organic phase dried (Na_2_SO_4_), evaporated to give 0.15 g (81%) of 1-phenyl-3-methyl-2-phospholene oxide (**4a**). The results are summarized in Table 2 and Scheme 1.

## Supporting Information

The general methods and instruments, the preparation of the starting materials, a few experimental procedures, the details of the optimization, kinetic studies and computations, as well as the characterization and NMR spectra of the new compounds can be found in the Supporting Information.

File 1 General methods, experimental and analytical data.
