# Supplementary material for: Preparation of 2-phospholene oxides by the isomerization of 3-phospholene oxides
Source: Beilstein J Org Chem. 2020 Apr 22;16:818–32. doi: 10.3762/bjoc.16.75 (PMC7189000; doi:10.3762/bjoc.16.75)
Supplement: File 1 — General methods, experimental and analytical data. [file Beilstein_J_Org_Chem-16-818-s001.pdf]

BEILSTEIN JOURNAL OF ORGANIC CHEMISTRY

Supporting Information

for

Preparation of 2-phospholene oxides by the isomerization of 3-phospholene oxides

Péter Bagi, Réka Herbay, Nikolett Péczka, Zoltán Mucsi, István Timári and and György Keglevich

Beilstein J. Org. Chem. **2020,** *16,* 818–832. doi:10.3762/bjoc.16.75

General methods, experimental and analytical data

License and Terms: This is a supporting information file under the terms of the Creative Commons Attribution License (http://creativecommons.org/licenses/by/4.0). Please note that the reuse, redistribution and reproduction in particular requires that the authors and source are credited.
The license is subject to the *Beilstein Journal of Organic Chemistry* terms and conditions: (https://www.beilstein-journals.org/bjoc)

Table of Contents

General methods (instruments) S3

Preparation of 1-(4-triflouromethylphenyl)-, (4-methoxyphenyl)- and (2,6-dimethylphenyl)-3-methyl-3-phospholene oxides **1d–f** (General procedure).. S5

General procedure for the preparation of 2-phospholene oxides **4**, **7** or **10** via the formation of chlorophospholenium chlorides **3**, **6** and **9**.. S6

Preliminary experiments for the preparation of 1-phenyl-3-methyl-2-phospholene oxide (**4a**) under acidic conditions.. S7

General procedure for the preparation of 2-phospholene oxides **4**, **7** or **10** using methanesulfonic acid S8

Preliminary experiments for the preparation of 1-phenyl-3-methyl-2-phospholene oxide (**4a**) under basic conditions.. S8

Preliminary experiments for the preparation of 1-phenyl- and 1-ethyl-3-methyl-2-phospholene oxide (**4a** and **4h**) using inorganic bases S10

Preliminary experiments for the investigation of the isomerization of 1-phenyl-3-methyl-3-phospholene oxide (**1a**) under thermal conditions using a solvent S11

General procedure for the isomerization of 3-phospholene oxides **1**, **5** and **8** under thermal conditions S12

Kinetic studies for the isomerization of 3-phospholene oxides **1c–f** under thermal conditions S13

Characterization of the 1-substituted-3-methyl-2-phospholene oxides **4**, **7** and **10**............... S16

^{31}P, ^{1}H and ^{13}C NMR spectra of the compounds **1d–f**, **3c**, **3h**, **4**, **7** and **10** prepared S21

Theoretical calculations... S62

General methods (instruments)

The oxalyl chloride, methanesulfonic acid, TEBAC and magnesium were purchased from Sigma Aldrich Ltd. 1-Bromo-4-(trifluoromethyl)benzene, 4-bromoanisole, 2-bromo-1,6-dimethylbenzene and LiCl were purchased from Fluorochem Ltd. Cs_2CO_3, K_2CO_3, Na_2CO_3 were purchased from Molar Chemicals. The thionyl chloride was purchased from Merck Chemicals Ltd. The reagents were used without further purification unless otherwise stated.
The solvents were purchased from Merck Chemicals Ltd. and they were used without further purification.

The 1-substituted-3-methyl-3-phospholene 1-oxides (**1a–c and 1g–l**), 1-phenyl-3,4-dimethyl-3-phospholene oxide (**5**) and 1-phenyl-3-phospholene oxide (**8**) were synthesized as described earlier [1-5].

The reactions involving organometallic reagents and oxalyl chloride were carried out under nitrogen atmosphere in Schlenk-type reaction vessels [6]. The solvents used in these reactions were purified and dried according to the standard procedures [7].

The ^{31}P, ^{19}F, ^{13}C, ^{1}H NMR spectra were taken on a Bruker AV-300 or DRX-500 spectrometer operating at 121.5, 282.4, 75.5 and 300 or 202.4, 470.7, 125.7 and 500 MHz, respectively. The ROESY spectra were recorded on a Bruker Avance II 500 MHz spectrometer equipped with a 5 mm TXI probe. The chemical shifts (δ) are given in parts per million (ppm). The chemical shifts (δ) for ^{1}H and ^{13}C in $CDCl_3$ and referenced to 7.26 and 77.16 ppm, respectively. 85% Solution of H_3PO_4 was the external reference for ^{31}P NMR chemical shifts. Coupling constants are expressed in Hertz (Hz). The following abbreviations are used: s = singlet, d = doublet, t = triplet, q = quadruplet, m = multiplet, dd = doublet of doublets, dt = doublet of triplets, dq = doublet of quadruplets.
The exact mass measurements were performed using an Agilent 6230C TOF LCMS System with Agilent Jet Stream source in positive ESI mode (buffer: ammonium formate in water/acetonitrile; drying gas: 325 °C; capillary: 3000 V; fragmentor 100 V).

Thin layer chromatography (TLC) was performed on Merck pre-coated Silica gel 60 F_{254} aluminium plates with realization by UV irradiation. Column chromatography was performed on Silica gel 60 with a particle size of 0.063–0.200 mm supplied by Merck. Flash column

chromatography was performed using a Combi-Flash® (Teledyne ISCO) using gradient elution (silica gel column; acetone: 2-propanol).

GC measurements were performed on an Agilent 4890 instrument equipped with a Restek, Rtx-5 column (15 m × 0.18 mm, 0.20 µm film, FID detector, nitrogen as carrier gas, injector 290 °C, detector 300 °C, head pressure: 10 psi, at 1:100 split ratio). Temperature program: 1 min at 40 °C, 25 °C/min to 300 °C, then kept at 300 °C for 20 min. Biphenyl was used as an internal standard. The retention times of the corresponding 3- and 2-phospholene oxides (**1**, **4**, **5**, **7**, **8** and **10**) are detailed in Table S1.

Table S1: GC retention time of 1-substutituted-3-methyl-3- and 2-phospholene oxides (**1**, **4**, **5**, **7**, **8** and **10**)

**Y**	**R^1**	**R^2**	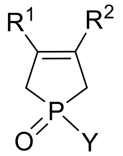 **Retention time (min)**	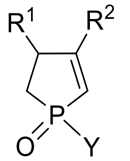 **Retention time (min)**
Ph	H	Me	10.6 (**1a**)	11.0 (**4a**)
2-Me-C_6H_4	H	Me	11.0 (**1b**)	11.7 (**4b**)
4-Me-C_6H_4	H	Me	11.2 (**1c**)	11.7 (**4c**)
4-CF_3-C_6H_4	H	Me	10.2 (**1d**)	10.6 (**4d**)
4-OMe-C_6H_4	H	Me	11.8 (**1e**)	12.3 (**4e**)
2,6-diMe-C_6H_3	H	Me	11.6 (**1f**)	11.9 (**4f**)
1-Naph	H	Me	13.3 (**1g**)	14.0 (**4g**)
Et	H	Me	8.1 (**1h**)	8.6 (**4h**)
^{n}Pr	H	Me	8.7 (**1i**)	9.1 (**4i**)
^{n}Bu	H	Me	9.2 (**1j**)	9.7 (**4j**)
^{i}Bu	H	Me	8.8 (**1k**)	9.3 (**4k**)
^{i}Pent	H	Me	9.5 (**1l**)	10.0 (**4l**)
Ph	Me	Me	11.1 (**5**)	11.2 (*cis*) and 11.4 (*trans*) (**7**)
Ph	H	H	10.5 (**8**)	10.8 (**10**)

Preparation of 1-(4-triflouromethylphenyl)-, (4-methoxyphenyl)- and (2,6-dimethylphenyl)-3-methyl-3-phospholene oxides (1d–f) (General procedure)

3.4 mL (47.4 mmol) of $SOCl_2$ was added dropwise to the solution of 4.8 g (36.4 mmol) of 1-hydroxy-3-methyl-3-phospholene oxide in 20 mL of dichloromethane at 0 °C. The reaction mixture was stirred at 26 °C for 12 hours. The volatiles were removed by vacuum distillation to afford the corresponding phosphinic chloride which was used immediately without further purification.

The solution of 7.7 mL (54.7 mmol) of 1-bromo-4-(trifluoromethyl)benzene in 35 mL THF was added dropwise to the suspension of 1.5 g (60.1 mmol) of Mg and 2.3 g (54.7 mmol) of LiCl in 15 mL of THF at 26 °C. The reaction mixture boiled spontaneously, and a gentle reflux was maintained by the addition of the reagent. The reaction mixture was then stirred at room temperature for 30 minutes.

The Grignard-reagent thus prepared was added dropwise to the phosphinic chloride in 20 mL of THF at 0 °C, and the reaction mixture was stirred overnight at 26 °C. Then, the reaction mixture was hydrolyzed by 20 mL of saturated NH_4Cl solution. The THF was removed under reduced pressure, the aqueous phase was neutralized with saturated $NaHCO_3$, and then it was extracted with 4 × 50 mL of EtOAc. The organic phase was dried (Na_2SO_4) and evaporated. The crude product was purified by column chromatography (silica gel, 3% methanol in dichloromethane) to give 6.8 g (yield = 72%) of 1-(4-triflourmethylphenyl)-3-methyl-3-phospholene oxide (**1d**) as a yellow oil.

^{19}F NMR ($CDCl_3$) δ -63.2. ^{31}P NMR ($CDCl_3$) δ 56.3. ^{1}H NMR ($CDCl_3$) δ 7.91 – 7.87 (m, 2H, ArH), 7.77 – 7.74 (m, 2H, ArH), 5.69 (d, J = 32.1, 1H, CH=), 2.95 – 2.59 (m, 4H, CH_2PCH_2), 1.91 (s, 3H, C_3-CH_3). ^{13}C NMR ($CDCl_3$) δ 138.7 ($^{1}J_{P\text{-}C}$ = 88.1, C_1'), 137.7 ($^{2}J_{P\text{-}C}$ = 13.2, C_3), 133.9 ($^{2}J_{F\text{-}C}$ = 32.8, $^{4}J_{P\text{-}C}$ = 2.6, C_4'), 130.2 ($^{2}J_{P\text{-}C}$ = 9.9, C_2'), 125.9 – 125.5 (m, C_3'), 123.7 ($^{1}J_{F\text{-}C}$ = 272.8, CF_3), 121.5 ($^{2}J_{P\text{-}C}$ = 8.0, C_4), 38.0 ($^{1}J_{P\text{-}C}$ = 69.2, C_2), 35.0 ($^{1}J_{P\text{-}C}$ = 66.4, C_5), 20.4 ($^{3}J_{P\text{-}C}$ = 11.1, C_3-CH_3). HRMS $[M+H]^{+}_{found}$ = 261.0652, $C_{12}H_{13}F_3OP$ requires 261.0658.

1-(4-Methoxyphenyl)-3-methyl-3-phospholene oxide (**1e**) was synthesized according to the general procedure described above using a Grignard-reagent prepared from 1.5 g (60.1 mmol) of Mg, 2.3 g (54.7 mmol) LiCl and 6.8 mL (54.7 mmol) 4-bromo-anisole to give 6.1 g (75%) of **1e**.

^{31}P NMR ($CDCl_3$) δ 57.9. ^{1}H NMR ($CDCl_3$) δ 7.72 – 7.57 (m, 2H, ArH), 7.00 – 6.98 (m, 2H, ArH), 5.63 (d, J = 31.2, 1H, CH=), 3.85 (s, 3H, OCH_3), 2.86 – 2.55 (m, 4H, CH_2PCH_2), 1.87 (bs, 3H, C_3-CH_3). ^{13}C NMR ($CDCl_3$) δ 162.4 ($^4J_{P\text{-}C}$ = 3.0, C_4'), 137.2 ($^2J_{P\text{-}C}$ = 12.7, C_3), 131.3 ($^2J_{P\text{-}C}$ = 11.0, C_2'), 124.7 ($^1J_{P\text{-}C}$ = 97.9, C_1'), 121.2 ($^2J_{P\text{-}C}$ = 7.7, C_4), 114.2 ($^3J_{P\text{-}C}$ = 12.6, C_3'), 55.2 (OCH_3), 38.1 ($^1J_{P\text{-}C}$ = 69.2, C_2), 35.1 ($^1J_{P\text{-}C}$ = 66.3, C_5), 20.2 ($^3J_{P\text{-}C}$ = 11.1, C_3-CH_3). HRMS $[M+H]^+_{found}$ = 223.0883, $C_{12}H_{16}O_2P$ requires 223.0890.

1-(2,6-Dimethylphenyl)-3-methyl-3-phospholene oxide (**1f**) was synthesized according to the general procedure described above (without LiCl) using a *Grignard*-reagent prepared from 1.5 g (60.1 mmol) of Mg and 7.3 mL (54.7 mmol) 2-bromo-1,6-dimethylbenzene to give 3.1 g (39 %) of **1f**.

^{31}P NMR ($CDCl_3$) δ 60.3. ^{1}H NMR ($CDCl_3$) δ 7.27 (t, J = 7.7, 1H, C_4'(H)), 7.11 – 7.07 (m, 2H, C_3'(H) and C_5'(H)), 5.67 (d, J = 29.6, 1H, CH=), 2.97 – 2.80 (m, 4H, CH_2PCH_2), 2.59 (s, 6H, 2 x Ar-CH_3), 1.90 (bs, 3H, C_3-CH_3). ^{13}C NMR ($CDCl_3$) δ 141.3 ($^2J_{P\text{-}C}$ = 10.0, C_3), 136.6 ($^2J_{P\text{-}C}$ = 11.1 Hz, C_2' and C_6'), 131.3 ($^1J_{P\text{-}C}$ = 90.8, C_1'), 131.1 ($^4J_{P\text{-}C}$ = 2.5, C_4'), 129.6 ($^2J_{P\text{-}C}$ = 10.4, C_3'), 120.6 ($^2J_{P\text{-}C}$ = 6.5, C_4), 41.6 ($^1J_{P\text{-}C}$ = 67.7, C_2), 38.4 ($^1J_{P\text{-}C}$ = 64.8, C_5), 23.0 ($^3J_{P\text{-}C}$ = 4.4, 2 x Ar-CH_3), 19.9 ($^3J_{P\text{-}C}$ = 10.5, C_3-CH_3). HRMS $[M+H]^+_{found}$ = 221.1095, $C_{13}H_{18}OP$ requires 221.1097.

General procedure for the preparation of 2-phospholene oxides 4, 7 or 10 via the formation of chlorophospholenium chlorides 3, 6 and 9

2-Phospholene oxides **4**, **7** or **10** were prepared as described in the Experimental section of the main article.

The 1–3% of 3-phospholene oxide (**1**, **5** or **8**) starting material could be removed by column chromatography (silica gel, 5% 2-propanol in acetone). The *cis*- and *trans*-1-phenyl-3,4-dimethyl-2-phospholene oxide (**10**) was separated by dry column vacuum chromatography [8] (silica gel, 20 % 2-propanol in EtOAc).

For the characterization of the 1-(4-methylphenyl)- and 1-ethyl-1-chloro-3-methyl-2-phospholenium chloride (**3c** and **3h**), the chlorophospholenium salts **3c** and **3h** were isolated prior to hydrolysis by the removal of the solvent under reduced pressure.

1-Chloro-3-methyl-1-(4-methylphenyl)-2-phospholenium chloride (**3c**)

Yield: 100%. ^{31}P NMR ($CDCl_3$) δ 97.6. ^{1}H NMR ($CDCl_3$) δ 7.96 (dd, J = 8.0, 16.0, 2H, Ar-H), 7.46 (dd, J = 4.5, 8.0, 2H, Ar-H), 6.46 (d, J = 32.7, 1H, CH=), 3.49 – 3.38 (m, 4H, C(4)H_2 and C(5)H_2), 2.41 (s, 1H, Ar-CH_3)[a], 2.36 (s, 3H, C_3-CH_3)[a]. [a] tentative assignment. ^{13}C NMR ($CDCl_3$) δ 183.5 ($^{2}J_{P\text{-}C}$ = 30.2, C_3), 148.0 ($^{4}J_{P\text{-}C}$ = 3.5, C_4'), 132.2 ($^{2}J_{P\text{-}C}$ = 14.6, C_2')[a], 131.1 ($^{3}J_{P\text{-}C}$ = 15.9, C_3')[a], 117.4 ($^{1}J_{P\text{-}C}$ = 94.1, C_1'), 109.6 ($^{1}J_{P\text{-}C}$ = 87.0, C_2), 37.2 ($^{2}J_{P\text{-}C}$ = 8.2, C_4), 29.2 ($^{1}J_{P\text{-}C}$ = 53.5, C_5), 22.5 ($^{3}J_{P\text{-}C}$ = 19.6, C_3-CH_3), 22.0 ($^{5}J_{P\text{-}C}$ = 1.7, Ar-CH_3). [a] tentative assignment.

1-Chloro-1-ethyl-3-methyl-2-phospholenium chloride (**3h**)

Yield: 100%. ^{31}P NMR ($CDCl_3$) δ 116.0. ^{1}H NMR ($CDCl_3$) δ 6.31 (d, J = 33.1, 1H, CH=), 3.29 – 3.12 (m, 6H, C(4)H_2, C(5)H_2, CH_2-CH_3), 2.26 (bs, 3H, C_3-CH_3), 1.43 (dt, J = 7.1, 25.5, 3H, CH_2-CH_3). ^{13}C NMR ($CDCl_3$) δ 181.5 ($^{2}J_{P\text{-}C}$ = 28.6, C_3), 109.5 ($^{1}J_{P\text{-}C}$ = 82.1, C_2), 37.2 ($^{2}J_{P\text{-}C}$ = 7.2, C_4), 25.6 ($^{1}J_{P\text{-}C}$ = 47.1, C_1')[a], 25.3 ($^{1}J_{P\text{-}C}$ = 49.7, C_5)[a], 22.2 ($^{3}J_{P\text{-}C}$ = 18.9, C_3-CH_3), 6.9 ($^{2}J_{P\text{-}C}$ = 6.3, C_2'), [a] tentative assignment.

Preliminary experiments for the preparation of 1-phenyl-3-methyl-2-phospholene oxide (4a) under acidic conditions

0.19 g (1.0 mmol) of 1-phenyl-3-methyl-3-phospholene oxide (**1a**) was dissolved in 1.0 mL of trifluoroacetic acid, 37% aqueous solution of HCl or methanesulfonic acid, and the reaction mixture was heated at a temperature of 25–160 °C for 24–120 h (see Table S2 for details). The reaction mixture was then cooled to 0 °C, and 3.0 mL of water was added. Then, the reaction mixture was neutralized with saturated $NaHCO_3$ at 0 °C. The solution was extracted with 5 × 3.0 mL dichloromethane. The organic phase dried (Na_2SO_4), evaporated to give a mixture of 1-phenyl-3-methyl-2- and 3-phospholene oxides (**4a** and **1a**). The results are summarized in Table S2.

Table S2: Preliminary experiments for the preparation of 1-phenyl-3-methyl-2-phospholene oxide (**4a**) under acidic conditions

Entry	Acid	Temperature (°C)	Reaction time (h)	Yield (%)[a]	Ratio of 4a:1a (%)[b]
**1**[c]	*p*-TsOH	100	24	90	0:100
**2**	HCl (aq. 37 %)	100	24	93	2:98
**3**	CF_3COOH	72	24	89	11:89
**4**	$MeSO_3H$	160	24	57	97:3
**5**	$MeSO_3H$	25	24	89	56:44
**6**	$MeSO_3H$	25	120	85	85:15
**7**	$MeSO_3H$	50	60	81	96:4

[a] Isolated yield of the mixture of **1a** and **4a**.

[b] Determined by GC.

[c] Reaction conditions: 1 eq. of *p*-TsOH, 1 mL of toluene.

General procedure for the preparation of 2-phospholene oxides (4, 7 or 10) using methanesulfonic acid

The 2-phospholene oxides (**4**, **7** or **10**) were prepared as described in the Experimental section of the main article.

Preliminary experiments for the preparation of 1-phenyl-3-methyl-2-phospholene oxide (4a) under basic conditions

0.10 g (0.5 mmol) of 1-phenyl-3-methyl-3-phospholene oxide (**1a**) was dissolved in 2.0 ml of toluene and 0.5 mmol of base (0.07 mL of Et_3N, 0.09 mL of iPr_2EtN, 0.04 mL of pyridine, 0.06 g of 4-dimethylaminopyridine or 0.16 g Cs_2CO_3) was added. The reaction mixture was refluxed for 24 h. The volatiles were removed, the reaction mixture was then filtered through a plug of silica with a 97:3 mixture of dichloromethane and methanol to give a mixture of 1-phenyl-3-methyl-2- and 3-phospholene oxide (**4a** and **1a**). The results are summarized in Table S3.

Table S3: Preliminary experiments for the preparation of 1-phenyl-3-methyl-2-phospholene oxide (**4a**) under basic conditions

**Entry**	**Base**	**Yield (%)[a]**	**Ratio of 4a:1a (%)[b]**
**1**	Et_3N	98	2:98
**2**	iPr_2EtN	85	3:97
**3**	Pyridine	85	4:96
**4**	4-Dimethylaminopyridine	95	2:98
**5[c]**	Cs_2CO_3	67	77:23

[a] Isolated yield of the mixture of **1a** and **4a**.
[b] Determined by GC.
[c] 5% TEBAC was used as PTC.

0.02 g of NaH (0.5 mmol, 60 %) was washed with 2 x 2.0 mL of THF, then it was suspended in 1.0 mL of THF. 0.10 g (0.5 mmol) of 1-Phenyl-3-methyl-3-phospholene oxide (**1a**) dissolved in 1.0 mL of THF was added dropwise to the NaH suspension at -78 °C. The reaction mixture was stirred for 30 min at -78 °C, then it was allowed to warm to 25°C and it was stirred for 24 h. The solution was added to 5.0 mL of water at 0 °C, and it was stirred 30 min at 0 °C. The phases were separated and the aqueous phase was extracted with 3 x 3.0 ml of dichloromethane. The combined organic phase was dried (Na_2SO_4), evaporated to give 0.09 g of crude product.

0.10 g of 1-phenyl-3-methyl-3-phospholene oxide (**1a**) (0.5 mmol) was dissolved in 1.0 mL of THF and 0.31 ml (0.5 mmol) of *n*-butyllithium (1.6 M in hexane) was added dropwise at −78 °C. The reaction mixture was stirred for 30 min at −78 °C, then it was allowed to warm to 25 °C and it was stirred for 24 h. The solution was added to 5.0 ml of water at 0 °C, and it was stirred 30 min at 0 °C. The phases were separated and the aqueous phase was extracted with 3 × 3.0 ml of dichloromethane. The combined organic phase was dried (Na_2SO_4), evaporated to give 0.09 g of crude product.

Preliminary experiments for the preparation of 1-phenyl- and 1-ethyl-3-methyl-2-phospholene oxide (4a and 4h) using inorganic bases

Representative procedure

0.16 g (0.05 mmol) of Cs_2CO_3 and 0.01 g (0.025 mmol) TEBAC was added to the solution of 0.10 g (0.05 mmol) of 1-phenyl-3-methyl-3-phospholene oxide (**1a**) in 2.0 mL of toluene. The reaction mixture was refluxed for 24 h. The volatiles were removed, and the reaction mixture was filtered through a plug of silica with 97:3 mixture of dichloromethane and methanol to give 0.067 g (67%) of a product containing 1-phenyl-3-methyl-2- and 3-phospholene oxides (**4a** and **1a**) in a ratio of 77:23 (Table S4, entry 1).

Other reactions listed in Table S4 were carried out according to the general procedure. When DMF or DMSO were used as the solvent, the work-up procedure was changed. After the reaction, 2.0 mL of water was added to the reaction mixture, and it was extracted with 5 x 2.0 mL of EtOAc. The organic phase was dried (Na_2SO_4), and evaporated to give the corresponding product.

*Repeated isomerization experiments for 1-phenyl- and 1-ethyl-3-methyl-3-phospholene oxides (**1a** or **1h**) in the presence of Cs_2CO_3*

0.16 g (0.05 mmol) of Cs_2CO_3 and 0.01 g (0.025 mmol) TEBAC was added to the solution of 0.05 mmol of 1-phenyl- or 1-ethyl-3-methyl-3-phospholene oxide (**1a**: 0.10 g, **1h**: 0.07 g) in 2.0 mL of DMF. The reaction mixture was refluxed for 24 h. 2.0 mL of water was added to the reaction mixture, and it was extracted with 5 × 2.0 mL of EtOAc. The organic phase was dried (Na_2SO_4) to give the product mixture. The crude product was reacted again two times as described above. The results are summarized in Table 3.

Table S4: Preliminary experiments for the preparation of 1-phenyl- and 1-ethyl-3-methyl-2-phospholene oxide (**4a** and **4h**) using inorganic bases

**Entry**	**3-phopsholene oxide (1)**	**Base**	**Amount of base (eq.)**	**TEBAC (%)**	**Solvent**	**Temperature (°C)**	**Yield (%)[a]**	**Ratio of 4:1 (%)[b]**
**1**	Ph (**a**)	Cs_2CO_3	1	5	toluene	110	67	77:23
**2**	Ph (**a**)	Cs_2CO_3	2	-	toluene	110	62	75:25
**3**	Ph (**a**)	K_2CO_3	2	5	toluene	110	80	64:36
**4**	Ph (**a**)	Na_2CO_3	2	5	toluene	110	75	1:99
**5**	Ph (**a**)	Cs_2CO_3	1	5	DMF	153	83	81:19
**6**	Ph (**a**)	Cs_2CO_3	2	5	DMF	153	77	84:16
**7**	Ph (**a**)	Cs_2CO_3	2	-	DMF	153	80	70:30
**8**	Ph (**a**)	K_2CO_3	2	5	DMF	153	89	80:20
**9**	Ph (**a**)	K_2CO_3	2	-	DMF	153	57	79:21
**10**	Ph (**a**)	KOH	1	5	DMF	153	87	78:22
**11**	Ph (**a**)	KOH	2	5	DMF	153	92	76:24
**12**	Ph (**a**)	K_2CO_3	2	5	DMSO	189	68	78:22
**13**	Ph (**a**)	K_2CO_3	2	-	DMSO	189	98	79:21
**14**	Ph (**a**)	Na_2CO_3	2	5	DMF	153	96	76:24
**15**	Ph (**a**)	NaOH	2	5	DMF	153	80	77:23
**16**	Ph (**a**)	NaOEt	2	5	DMF	153	68	74:26
**17**	Ph (**a**)	NaOEt	2	5	DMSO	189	95	72:28
**18**	Et (**h**)	Cs_2CO_3	1	5	toluene	110	65	56:44
**19**	Et (**h**)	Cs_2CO_3	1	5	DMF	153	69	69:31
**20**	Et (**h**)	Cs_2CO_3	2	5	DMF	153	68	66:34

[a] Isolated yield of the mixture of **4a** and **1a**.
[b] Determined by GC.

Preliminary experiments for the investigation of the isomerization of 1-phenyl-3-methyl-3-phospholene oxide (1a) under thermal conditions using a solvent

0.19 g (1.0 mmol) of 1-phenyl-3-methyl-3-phospholene oxide (**1a**) was dissolved in 2.0 mL of toluene, DMF or DMSO and the solution was refluxed for 24 h. A solvent was evaporated, and the reaction mixture was filtered through a plug of silica with 97:3 mixture of dichloromethane and methanol to give a product containing 1-phenyl-3-methyl-2- and 3-phospholene oxides (**4a** and **1a**). The results are summarized Table S5.

Table S5: Preliminary experiments for the investigation of the isomerization of 1-phenyl-3-methyl-3-phospholene oxide (**1a**) under thermal conditions

Entry	Solvent	Temperature (°C)	Yield (%)[a]	Ratio of 4a:1a (%)[b]
**1**	toluene	110	95	2:98
**2**	DMF	157	98	2:98
**3**	DMSO	189	94	4:96

[a] Isolated yield of the mixture of **1a** and **4a**.
[b] Determined by GC.

General procedure for the isomerization of 3-phospholene oxides 1, 5 and 8 under thermal conditions

1.0 mmol of the 3-phospholene oxide (**1a**: 0.19 g, **1b**: 0.21 g, **1c**: 0.21 g, **1d**: 0.26 g, **1e**: 0.22, **1f**: 0.22, **1g**: 0.24, **1h**: 0.14 g, **1i**: 0.16 g, **1j**: 0.17 g, **1k**: 0.17 g, **1l**: 0.19 g, **5**: 0.18 g, **8**: 0.21 g) was stirred in a 1.5 mL vial at 200 °C for 24–120 h. The reaction mixture was then filtered through a plug of silica with 97:3 mixture of dichloromethane and methanol to give a product containing a mixture of 2- and 3-phospholene oxides (**4** and **1**, **7** and **5**, **10** and **8**). The results are summarized Table 4 and Scheme 1, and in Table S6.

Table S6: Comparison of the reaction time on the isomerization of 3-phospholene oxides (1) under thermal conditions

Entry	Y	Reaction time (h)	Yield (%)[a]	Ratio of 4:1 (%)[b]
**1**	Ph (**a**)	24	58	71:29
**2**	Ph (**a**)	120	40	71:29
**3**	Et (**h**)	24	50	55:45
**4**	Et (**h**)	120	40	58:42

[a] Isolated yield of the mixture of **1a** and **4a**.
[b] Determined by GC.

Kinetic studies for the isomerization of 3-phospholene oxides 1c–f and 1h under thermal conditions

1.0 mmol of the Corresponding 3-phospholene oxide (**1c**: 0.21 g, **1d**: 0.26 g, **1e**: 0.22 g, **1f**: 0.22 g, **1h**: 0.14 g) was stirred in a 1.5 mL vial at 200 °C. Samples were taken from the reaction mixture (every 15 min in the 1–2 h period; every 30 min in the 3–4 h period ; every 60 min in the 4–8 hour period; then at 24 h). Every sample was filtered through a plug of silica with a 97:3 mixture of dichloromethane and methanol. The ratio of the 2- and 3-phospholene oxides (**4** and **1**) was determined by GC. The results are summarized in Table S7.

Table S7: Kinetic studies for the isomerization of 3-phospholene oxides **1c-f** and **1h** under thermal conditions

	Relative amounts of **1** and **4** (%)									
Y=	4-Me-C_6H_4		4-CF_3-C_6H_4		4-MeO-C_6H_4		2,6-diMe-C_6H_3		Et	
Time (min)	**1c**	**4c**	**1d**	**4d**	**1e**	**4e**	**1f**	**4f**	**1h**	**4h**
15	1.9	98.1	0.9	99.1	2.2	97.8	2.6	97.4	2.3	97.7
30	2.8	97.2	1.6	98.4	3.7	96.3	3.0	97.0	3.4	96.6
45	3.4	96.6	2.3	97.7	5.5	94.5	3.8	96.2	5.8	94.2
60	3.6	96.4	3.6	96.4	6.6	93.4	5.7	94.3	6.7	93.3
75	4.4	95.6	4.3	95.7	7.7	92.3	8.1	91.9	9.6	90.4
90	4.7	95.3	5.0	95.0	9.6	90.4	9.6	90.4	11.2	88.8
105	6.0	94.0	5.8	94.2	10.8	89.2	11.0	89.0	14.9	85.1
120	6.6	93.4	6.2	93.8	12.9	87.1	13.8	86.2	17.5	82.5
150	8.5	91.5	7.7	92.3	16.1	83.9	19.3	80.7	20.2	79.8
180	10.6	89.4	8.0	92.0	18.5	81.5	23.4	76.6	23.5	76.5
210	14.0	86.0	12.0	88.0	22.0	78.0	27.8	72.2	25.1	74.9
240	17.6	82.4	13.7	86.3	25.0	75.0	31.9	68.1	27.0	73.0
300	25.1	74.9	17.8	82.2	30.3	69.7	41.8	58.2	31.9	68.1
360	30.5	69.5	22.8	77.2	37.0	63.0	49.5	50.5	38.3	61.7
420	35.8	64.2	27.5	72.5	42.5	57.5	57.4	42.6	39.5	60.5
480	43.1	56.9	33.1	66.9	47.8	52.2	63.3	36.7	40.1	59.9
1440	67.8	32.2	71.7	28.3	75.4	24.6	79.0	21.0	51.5	48.5
1920	73.4	26.6	75.4	24.6	76.9	23.1	79.8	20.2	54.7	45.3
2880	76.5	23.5	80.4	19.6	77.0	23.0	81.3	18.7	56.3	43.7

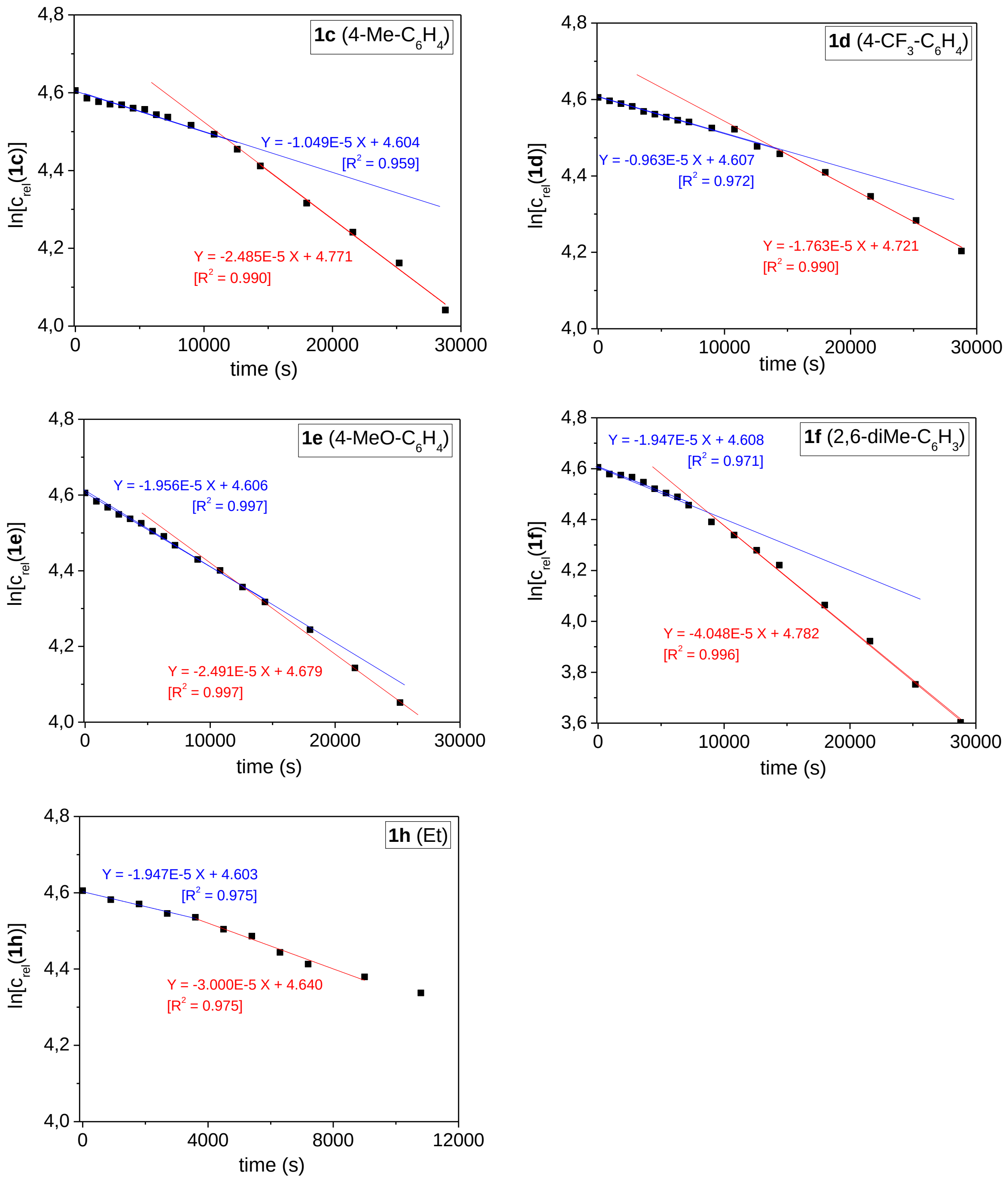

Figure S1. Initial fittings (blue) and second fittings (red) of the kinetic curves of the isomerization of 3-phospholene oxides (**1c-f** and **1h**) under thermal conditions

Table S8: Experimental reaction rates of the transformation of **1c–h**→**4c–h**. The initial and second fittings are described in Figure S1

		Initial fitting for 0 – 10 000s		Second fitting for 10 000 – 20 000s		Acceleration[a]
		rate (–m in 1/s)	intercept (b)	rate (–m in 1/s)	intercept (b)	
**1c**	4-Me-C_6H_4	1.049 10^{-5}	4.604	2.485 10^{-5}	4.771	2.369
**1d**	4-CF_3-C_6H_4	0.963 10^{-5}	4.607	1.763 10^{-5}	4.721	1.831
**1e**	4-MeO-C_6H_4	1.956 10^{-5}	4.606	2.491 10^{-5}	4.679	1.274
**1f**	2,6-diMe-C_6H_3	1.947 10^{-5}	4.608	4.048 10^{-5}	4.782	2.079
**1h**	Et	1.947 10^{-5}	4.640	3.001 10^{-5}	4.721	1.541

[a]Acceleration = rate(Second fitting) / Rate(Initial fitting)

Characterization of the 1-substituted-3-methyl-2-phospholene oxides 4, 7 and 10

1-Phenyl-3-methyl-2-phospholene oxide (**4a**):

Colourless oil. R_f = 0.40 (silica gel, 5% 2-propanol in acetone).

^{31}P NMR ($CDCl_3$) δ 61.9 (δ_{lit} 61.8) [9]. ^{1}H NMR ($CDCl_3$) δ 7.71 – 7.64 (m, 2H, ArH), 7.55 – 7.44 (m, 3H, ArH), 5.95 (d, *J* = 25.2, 1H, CH=), [2.92 – 2.77 (m, 1H) and 2.70 – 2.54 (m, 1H)] (C(4)H_2), 2.29 – 2.18 (m, 2H, C(5)H_2), 2.09 (s, 3H, C_3-CH_3). ^{13}C NMR ($CDCl_3$) δ 165.0 ($^2J_{P\text{-}C}$ = 25.4, C_3), 134.1 ($^1J_{P\text{-}C}$ = 98.4, C_1’), 131.7 ($^4J_{P\text{-}C}$ = 2.9, C_4’), 130.6 ($^2J_{P\text{-}C}$ = 10.3, C_2’), 128.6 ($^3J_{P\text{-}C}$ = 12.0, C_3’), 120.5 ($^1J_{P\text{-}C}$ = 99.9, C_2), 34.2 ($^2J_{P\text{-}C}$ = 8.4, C_4), 27.5 ($^1J_{P\text{-}C}$ = 70.1, C_5), 21.1 ($^3J_{P\text{-}C}$ = 17.3, C_3-CH_3). HRMS $[M+H]^+_{found}$ = 193.0777, $C_{11}H_{14}OP$ requires 193.0784.

1-(2-Methylphenyl)-3-methyl-2-phospholene oxide (**4b**):

Colourless oil. R_f = 0.46 (silica gel, 5% 2-propanol in acetone).

^{31}P NMR ($CDCl_3$) δ 63.6. ^{1}H NMR ($CDCl_3$) δ 7.65 – 7.60 (m, 1H, ArH), 7.41 – 7.37 (m, 1H, ArH), 7.29 – 7.22 (m, 2H, ArH), 6.12 (d, *J* = 24.3, 1H, CH=), 2.59 (s, 3H, Ar-CH_3), [2.88 – 2.80 (m, 1H), 2.57 – 2.49 (m, 1H)] (C(4)H_2), 2.32 – 2.19 (m, 2H, C(5)H_2), 2.06 (bs, 3H, C_3-CH_3). ^{13}C NMR ($CDCl_3$) δ164.2 ($^2J_{P\text{-}C}$ = 25.3, C_3), 140.9 ($^2J_{P\text{-}C}$ = 8.9, C_2’), 132.6 ($^1J_{P\text{-}C}$ = 96.5, C_1’), 131.6 ($^4J_{P\text{-}C}$ = 2.7, C_4’), 131.4 ($^2J_{P\text{-}C}$ = 10.4, C_6’)*, 131.2 ($^3J_{P\text{-}C}$ = 11.4, C_3’)*, 125.5 ($^3J_{P\text{-}C}$ = 12.0, C_5’)*, 120.3 ($^1J_{P\text{-}C}$ = 101.0, C_2), 34.2 ($^2J_{P\text{-}C}$ = 8.4, C_4), 26.9 ($^1J_{P\text{-}C}$ = 69.2, C_5), 21.1 ($^3J_{P\text{-}C}$ = 2.0, Ar-CH_3), 21.1 ($^3J_{P\text{-}C}$ = 19.7, C_3-CH_3). * may be reversed. HRMS $[M+H]^+_{found}$ = 207.0938, $C_{12}H_{16}OP$ requires 207.0941.

1-(4-Methylphenyl)-3-methyl-2-phospholene oxide (**4c**):

Colourless oil. R_f = 0.35 (silica gel, 5% 2-propanol in acetone).

^{31}P NMR ($CDCl_3$) δ 62.0. ^{1}H NMR ($CDCl_3$) δ 7.59 – 7.54 (m, 2H, ArH), 7.30 – 7.26 (m, 2H, ArH), 5.93 (d, *J* = 25.0, 1H, CH=), [2.86 – 2.78 (m, 1H) and 2.65 – 2.57 (m, 1H)] C(4)H_2, 2.40 (s, 3H, Ar-CH_3), 2.28 – 2.15 (m, 2H, C(5)H_2), 2.07 (bs, 3H, C_3-CH_3). ^{13}C NMR ($CDCl_3$) δ 164.4 ($^2J_{P\text{-}C}$ = 25.3, C_3), 142.0 ($^4J_{P\text{-}C}$ = 3.0, C_4’), 130.9 ($^1J_{P\text{-}C}$ = 100.4, C_1’), 130.6 ($^2J_{P\text{-}C}$ = 10.9, C_2’), 129.3 ($^3J_{P\text{-}C}$ = 12.4, C_3’), 120.9 ($^1J_{P\text{-}C}$ = 99.5, C_2), 34.2 ($^2J_{P\text{-}C}$ = 8.3, C_4), 27.7 ($^1J_{P\text{-}C}$ = 70.0, C_5), 21.6 (Ar-CH_3), 21.1 ($^3J_{P\text{-}C}$ = 17.2, C_3-CH_3). HRMS $[M+H]^+_{found}$ = 207.0937, $C_{12}H_{16}OP$ requires 207.0941.

1-(4-Trifluoromethylphenyl)-3-methyl-2-phospholene oxide (**4d**):

Yellow solid. R_f = 0.57 (silica gel, 5% 2-propanol in acetone).

^{19}F NMR ($CDCl_3$) δ -63.1. ^{31}P NMR ($CDCl_3$) δ 60.4. ^{1}H NMR ($CDCl_3$) δ 7.85 – 7.80 (m, 2H, ArH), 7.74 – 7.71 (m, 2H, ArH), 5.96 (d, *J* = 25.3, 1H, CH=), [2.92 – 2.83 (m, 1H) and 2.72 – 2.62 (m, 1H)] (C(4)H_2), 2.31 – 2.20 (m, 2H, (C(5)H_2)), 2.12 (bs, 3H, C_3-CH_3). ^{13}C NMR ($CDCl_3$) δ 166.0 ($^2J_{P\text{-}C}$ = 26.0, C_3), 138.6 ($^1J_{P\text{-}C}$ = 94.9, $C_{1'}$), 133.3 ($^2J_{F\text{-}C}$ = 32.7 and $^4J_{P\text{-}C}$ = 3.0, $C_{4'}$), 131.0 ($^2J_{P\text{-}C}$ = 10.7, $C_{2'}$), 125.3 ($^3J_{P\text{-}C}$ = 11.5 and $^3J_{F\text{-}C}$ = 3.7, $C_{3'}$), 123.6 ($^1J_{F\text{-}C}$ = 272.6, CF_3), 119.8 ($^1J_{P\text{-}C}$ = 100.8, C_2), 34.1 ($^2J_{P\text{-}C}$ = 8.8, C_4), 27.2 ($^1J_{P\text{-}C}$ = 70.1, C_5), 21.0 ($^3J_{P\text{-}C}$ = 17.4, C_3-CH_3). HRMS $[M+H]^+_{found}$ = 261.0650, $C_{12}H_{13}F_3OP$ requires 261.0658.

1-(4-Methoxyphenyl)-3-methyl-2-phospholene oxide (**4e**):

Colourless oil. R_f = 0.44 (silica gel, 5% 2-propanol in acetone).

^{31}P NMR ($CDCl_3$) δ 61.6. ^{1}H NMR ($CDCl_3$) δ 7.62 – 7.56 (m, 2H, ArH), 6.99 – 6.95 (m, 2H, ArH), 5.91 (d, *J* = 25.2, 1H, CH=), 3.84 (s, 3H, OCH_3), [2.85 – 2.76 (m, 1H) and 2.64 – 2.57 (m, 1H)] (C(4)H_2), 2.23 – 2.15 (m, 1H, C(5)H_2), 2.07 (bs, 3H, C_3-CH_3). ^{13}C NMR ($CDCl_3$) δ 164.2 ($^3J_{P\text{-}C}$ = 25.3, C_3), 162.2 ($^4J_{P\text{-}C}$ = 3.0, $C_{4'}$), 132.2 ($^3J_{P\text{-}C}$ = 11.8, $C_{2'}$), 125.0 ($^1J_{P\text{-}C}$ = 104.4, $C_{1'}$)[b], 120.7 ($^1J_{P\text{-}C}$ = 99.9, C_2)[b], 114.0 ($^3J_{P\text{-}C}$ = 12.9, $C_{3'}$), 55.2 (OCH_3), 33.9 ($^2J_{P\text{-}C}$ = 8.4, C_4), 27.5 ($^1J_{P\text{-}C}$ = 70.1, C_5), 20.9 ($^3J_{P\text{-}C}$ = 17.2, C_3-CH_3). [a,b] tentative assignment. HRMS $[M+H]^+_{found}$ = 223.0882, $C_{12}H_{16}O_2P$ requires 223.0890.

1-(2,6-Dimethylphenyl)-3-methyl-2-phospholene oxide (**4f**):

Colourless oil. R_f = 0.34 (silica gel, 5% 2-propanol in acetone).

^{31}P NMR ($CDCl_3$) δ 63.2. ^{1}H NMR ($CDCl_3$) δ 7.22 (t, *J* = 7.5, 1H, C(4')H), 7.06 (dd, *J* = 7.7, 3.8, 2H, 2 x C(3')H), 6.28 (d, *J* = 25.2, 1H, CH=), [2.82 – 2.74 (m, 1H), 2.54 – 2.44 (m, 1H)] (C(4)H_2), 2.61 (s, 6H, s, 6H, 2 x Ar-CH_3), 2.41 – 2.29 (m, 2H, C(5)H_2), 2.05 (s, 3H, C_3-CH_3). ^{13}C NMR ($CDCl_3$) δ 162.0 ($^2J_{P\text{-}C}$ = 25.8, C_3), 140.5 ($^2J_{P\text{-}C}$ = 9.7, $C_{2'}$), 133.7 ($^1J_{P\text{-}C}$ = 96.9, $C_{1'}$), 130.9 ($^4J_{P\text{-}C}$ = 2.5, $C_{4'}$), 129.6 ($^3J_{P\text{-}C}$ = 10.5, $C_{3'}$), 123.8 ($^1J_{P\text{-}C}$ = 98.1, C_2), 33.9 ($^2J_{P\text{-}C}$ = 8.2, C_4), 29.6 ($^1J_{P\text{-}C}$ = 68.9, C_5), 23.8 ($^3J_{P\text{-}C}$ = 4.6, Ar-CH_3), 21.1 ($^3J_{P\text{-}C}$ = 17.2, C_3-CH_3). HRMS $[M+H]^+_{found}$ = 221.1095, $C_{13}H_{18}OP$ requires 221.1097.

1-(1-Naphthyl)-3-methyl-2-phospholene oxide (**4g**):

Yellow solid. R_f = 0.50 (silica gel, 5% 2-propanol in acetone).

^{31}P NMR ($CDCl_3$) δ 63.6. ^{1}H NMR ($CDCl_3$) δ 8.54 (d, J = 8.4, 1H, ArH), 8.00 (d, J = 8.2, 1H, ArH), 7.92 (d, J = 8.2, 1H, ArH), 7.89 – 7.83 (m, 1H, ArH), 7.63 – 7.55 (m, 2H, ArH), 7.52 – 7.48 (m, 1H, ArH), 6.28 (d, J = 25.9, 1H, CH=), [2.94 – 2.85 (m, 1H) and 2.65 – 2.56 (m, 1H)] (C(4)H_2), 2.45 – 2.34 (m, 2H, C(5)H_2), 2.12 (s, 3H, C_3-CH_3). ^{13}C NMR ($CDCl_3$) δ 164.7 ($^2J_{P\text{-}C}$ = 25.8, C_4), 133.9 ($J_{P\text{-}C}$ = 8.9, C_{Ar}), 132.9 ($J_{P\text{-}C}$ = 9.0, C_{Ar}), 132.7 ($J_{P\text{-}C}$ = 2.9, C_{Ar}), 131.0 ($J_{P\text{-}C}$ = 10.7, C_{Ar}), 130.8 ($^1J_{P\text{-}C}$ = 95.5, C_1’), 129.1 (C_{Ar}), 127.4 (C_{Ar}), 126.5 (C_{Ar}), 126.1 ($J_{P\text{-}C}$ = 5.5, C_{Ar}), 124.6 ($J_{P\text{-}C}$ = 13.5, C_{Ar}), 120.6 ($^1J_{P\text{-}C}$ = 101.8, C_2), 34.4 ($^2J_{P\text{-}C}$ = 8.6, C_4), 27.5 ($^1J_{P\text{-}C}$ = 69.7, C_5), 21.2 ($^3J_{P\text{-}C}$ = 17.2, C_3-CH_3). HRMS $[M+H]^+_{found}$ = 243.0932, $C_{15}H_{16}OP$ requires 243.0941.

1-Ethyl-3-methyl-2-phospholene oxide (**4h**):

Colourless oil. R_f = 0.15 (silica gel, 5% 2-propanol in acetone).

^{31}P NMR ($CDCl_3$) δ 74.7. ^{1}H NMR ($CDCl_3$) δ 5.86 (d, J = 25.3, 1H, CH=), [2.76 – 2.68 (m, 1H), 2.49 – 2.41 (m, 1H)] (C(4)H_2), 2.02 – 2.09 (m, 2H, C(5)H_2), 1.98 (s, 3H, C_3-CH_3), 1.85 – 1.93 (m, 2H, CH_2-CH_3), 1.11 – 1.18 (m, 3H, CH_2-CH_3). ^{13}C NMR ($CDCl_3$) δ 163.3 ($^2J_{P\text{-}C}$ = 23.9, C_3), 119.5 ($^1J_{P\text{-}C}$ = 95.2, C_2), 34.1 ($^2J_{P\text{-}C}$ = 7.9, C_4), 24.1 ($^1J_{P\text{-}C}$ = 68.9, C_1’)[a], 24.0 ($^1J_{P\text{-}C}$ = 66.2, C_5)[a], 21.0 ($^3J_{P\text{-}C}$ = 16.5, C_3-CH_3), 6.6 ($^2J_{P\text{-}C}$ = 4.1, C_2’). [a] tentative assignment. HRMS $[M+H]^+_{found}$ = 145.0748, $C_7H_{14}OP$ requires 145.0784.

1-Propyl-3-methyl-2-phospholene oxide (**4i**):

Colourless oil. R_f = 0.16 (silica gel, 5% 2-propanol in acetone).

^{31}P NMR ($CDCl_3$) δ 72.4. ^{1}H NMR ($CDCl_3$) δ 5.86 (d, J = 25.4, 1H, CH=), [2.78 – 2.66 (m, 1H) and 2.50 – 2.40 (m, 1H)] (C(4)H_2), 2.09 – 1.98 (m, 2H, C(5)H_2), 1.96 (bs, 3H, C_3-CH_3), 1.90 – 1.81 (m, 2H, CH_2-CH_2-CH_3), 1.67 – 1.56 (m, 2H, CH_2-CH_2-CH_3), 1.05 (t, J = 7.3, 3H, CH_2-CH_2-CH_3). ^{13}C NMR ($CDCl_3$) δ 162.9 ($^2J_{P\text{-}C}$ = 24.1, C_3), 120.0 ($^1J_{P\text{-}C}$ = 94.9, C_2), 33.9 ($^2J_{P\text{-}C}$ = 7.8, C_4), 33.3 ($^1J_{P\text{-}C}$ = 68.0, C_1’), 24.6 ($^1J_{P\text{-}C}$ = 66.3, C_5), 20.8 ($^3J_{P\text{-}C}$ = 16.5, C_3-CH_3), 16.1 ($^2J_{P\text{-}C}$ = 3.5, C_2’), 15.6 ($^3J_{P\text{-}C}$ = 14.7, C_3’). HRMS $[M+H]^+_{found}$ = 159.0933, $C_8H_{16}OP$ requires 159.0941.

1-Butyl-3-methyl-2-phospholene oxide (**4j**):

Colourless oil. R_f = 0.20 (silica gel, 5% 2-propanol in acetone).

^{31}P NMR ($CDCl_3$) δ 72.5. ^{1}H NMR ($CDCl_3$) δ 5.86 (d, *J* = 25.3, 1H, CH=), [2.75 – 2.65 (m, 1H) and 2.50 – 2.38 (m, 1H)] (C(4)H_2), 2.07 – 2.00 (m, 2H, C(5)H_2), 1.96 (bs, 3H, C_3-CH_3), 1.89 – 1.83 (m, 2H, P-C*H*$_2$), 1.59 – 1.51 (m, 2H, C*H*$_2$-CH_2-CH_3), 1.47 – 1.40 (m, 2H, C*H*$_2$-CH_3), 0.93 (t, *J* = 7.0, 3H, CH_2-C*H*$_3$). ^{13}C NMR ($CDCl_3$) δ 162.8 ($^{2}J_{P\text{-}C}$ = 23.9, C_3), 120.0 ($^{1}J_{P\text{-}C}$ = 95.1, C_2), 33.9 ($^{2}J_{P\text{-}C}$ = 8.0, C_4), 31.0 ($^{1}J_{P\text{-}C}$ = 68.0, C_1’), 24.6 ($^{1}J_{P\text{-}C}$ = 66.7, C_5), 24.5 ($^{2}J_{P\text{-}C}$ = 3.7, C_2’), 24.1 ($^{3}J_{P\text{-}C}$ = 14.9, C_3’), 20.9 ($^{3}J_{P\text{-}C}$ = 16.4, C_3-CH_3), 13.7 (C_4’). HRMS $[M+H]^{+}_{found}$ = 173.1090, $C_9H_{18}OP$ requires 173.1097.

1-Isobutyl-3-methyl-2-phospholene oxide (**4k**):

Colourless oil. R_f = 0.24 (silica gel, 5% 2-propanol in acetone).

^{31}P NMR ($CDCl_3$) δ 70.5. ^{1}H NMR ($CDCl_3$) δ 5.88 (d, *J* = 25.5, 1H, CH=), [2.75 – 2.67 (m, 1H) and 2.50 – 2.42 (m, 1H)] C(4)H_2, 2.22 – 2.12 (m, 1H, C*H*(CH_3)$_2$), 2.06 – 1.98 (m, 2H, C(5)H_2), 1.96 (bs, 3H, C_3-CH_3), 1.85 – 1.74 (m, 2H, P-CH_2), 1.10 – 1.07 (m, 6H, CH(C*H*$_3$)$_2$). ^{13}C NMR ($CDCl_3$) δ 162.4 ($^{2}J_{P\text{-}C}$ = 24.0, C_3), 121.0 ($^{1}J_{P\text{-}C}$ = 94.6, C_2), 40.6 ($^{1}J_{P\text{-}C}$ = 67.4, C_1’), 33.8 ($^{2}J_{P\text{-}C}$ = 7.8, C_4), 26.0 ($^{1}J_{P\text{-}C}$ = 66.4, C_5), 24.6 – 24.3 (m, C_3’ and C_4’), 24.0 ($^{2}J_{P\text{-}C}$ = 4.0, C_2’), 20.8 ($^{3}J_{P\text{-}C}$ = 16.4, C_3-CH_3). HRMS $[M+H]^{+}_{found}$ = 173.1092, $C_9H_{18}OP$ requires 173.1097.

1-Isopentyl-3-methyl-2-phospholene oxide (**4l**):

Colourless oil. R_f = 0.22 (silica gel, 5% 2-propanol in acetone).

^{31}P NMR ($CDCl_3$) δ 73.2. ^{1}H NMR ($CDCl_3$) δ 5.86 (d, *J* = 25.3, 1H), [2.75 – 2.67 (m, 1H) and 2.48 – 2.40 (m, 1H)] (C(4)H2), 2.07 – 1.99 (m, 2H, C(5)H_2), 1.96 (bs, 3H, C_3-CH_3), 1.88 – 1.81 (m, 2H, P-C*H*$_2$), 1.65 – 1.57 (m, 1H, C*H*(CH_3)$_2$), 1.48 – 1.41 (m, 2H, P-CH_2-C*H*$_2$), 0.91 (d, *J* = 6.6, 6H, CH(C*H*$_3$)$_2$). ^{13}C NMR ($CDCl_3$) δ 162.9 ($^{2}J_{P\text{-}C}$ = 24.0, C_3), 119.7 ($^{1}J_{P\text{-}C}$ = 95.2, C_2), 33.8 ($^{2}J_{P\text{-}C}$ = 7.9, C_4), 31.0 ($^{4}J_{P\text{-}C}$ = 3.6, C_2’), 29.1 ($^{1}J_{P\text{-}C}$ = 68.2, C_1’), 29.0 ($^{3}J_{P\text{-}C}$ = 14.4, C_3’), 24.3 ($^{1}J_{P\text{-}C}$ = 66.6, C_5), 22.0 (C_4’ and C_5’), 20.8 ($^{3}J_{P\text{-}C}$ = 16.5, C_3-CH_3). HRMS $[M+H]^{+}_{found}$ = 187.1247, $C_{10}H_{20}OP$ requires 187.1254.

1-Phenyl-2-phospholene oxide (**7**):

White solid. R_f = 0.31 (silica gel, 5% 2-propanol in acetone).

^{31}P NMR ($CDCl_3$) δ 61.3 (δ_{lit} 62.4) [10]. ^{1}H NMR ($CDCl_3$) δ 7.70 – 7.66 (m, 2H, ArH), 7.53 – 7.48 (m, 3H, ArH), 7.15 (ddt, *J* = 43.1, 8.2, 2.5, 1H, C(2)H), 6.32 (ddt, *J* = 25.2, 8.5, 2.7, 1H, C(3)H), [3.02 – 2.94 (m, 1H) and 2.81 – 2.71 (m, 1H)] (C(5)H_2), 2.21 – 2.10 (m, 2H, C(4)H_2). ^{13}C NMR ($CDCl_3$) δ 152.8 ($^{2}J_{P\text{-}C}$ = 24.4, C_3), 133.4 ($^{1}J_{P\text{-}C}$ = 98.1, $C_{1'}$), 131.8 ($^{4}J_{P\text{-}C}$ = 3.0, $C_{4'}$), 130.4 ($^{2}J_{P\text{-}C}$ = 10.5, $C_{2'}$), 128.6 ($^{3}J_{P\text{-}C}$ = 12.1, $C_{3'}$), 126.2 ($^{1}J_{P\text{-}C}$ = 93.3, C_2), 30.1 ($^{2}J_{P\text{-}C}$ = 10.4, C_4), 25.5 ($^{1}J_{P\text{-}C}$ = 71.5, C_5). HRMS $[M+H]^{+}_{found}$ = 179.0621, $C_{10}H_{12}OP$ requires 179.0628.

cis-1-Phenyl-3,4-dimethyl-2-phospholene oxide (*cis*-**10**):

Colourless oil. R_f = 0.52 (silica gel, 20% 2-propanol in EtOAc).

^{31}P NMR ($CDCl_3$) δ 54.7. ^{1}H NMR ($CDCl_3$) δ 7.69 – 7.64 (m, 2H, ArH), 7.52 – 7.43 (m, 3H, ArH), 5.92 (d, *J* = 23.8, 1H, CH=), 2.90 – 2.81 (m, 1H, C(4)H), [2.44 (ddd, J = 15.3, 8.2, 6.8, 1H) and 1.88 (ddd, J = 17.8, 15.6, 4.5, 1H)] (C(5)H_2), 2.06 (s, 3H, C_3-CH_3), 1.35 (d, *J* = 7.1, 3H, C_4-CH_3). ^{13}C NMR ($CDCl_3$) δ 168.4 ($^{2}J_{P\text{-}C}$ = 24.1, C_3), 134.2 ($^{1}J_{P\text{-}C}$ = 98.8, $C_{1'}$), 131.6 ($^{4}J_{P\text{-}C}$ = 3.0, $C_{4'}$), 130.6 ($^{2}J_{P\text{-}C}$ = 10.4, $C_{2'}$), 128.6 ($^{3}J_{P\text{-}C}$ = 11.9, $C_{3'}$), 120.9 ($^{1}J_{P\text{-}C}$ = 99.0, C_2), 40.7 ($^{2}J_{P\text{-}C}$ = 8.5, C_4), 36.2 ($^{1}J_{P\text{-}C}$ = 69.1, C_5), 21.1 ($^{3}J_{P\text{-}C}$ = 4.6, C_4-CH_3), 19.3 ($^{3}J_{P\text{-}C}$ = 18.5, C_3-CH_3). HRMS $[M+H]^{+}_{found}$ = 207.0930, $C_{12}H_{16}OP$ requires 207.0941.

trans-1-Phenyl-3,4-dimethyl-2-phospholene oxide (*trans*-**10**):

Colourless oil. R_f = 0.40 (silica gel, 20% 2-propanol in EtOAc).

^{31}P NMR ($CDCl_3$) δ 55.2. ^{1}H NMR ($CDCl_3$) δ 7.74 – 7.69 (m, 2H, ArH), 7.53 – 7.46 (m, 3H, ArH), 5.89 (d, *J* = 23.8, 1H, CH=), 3.16 – 3.08 (m, 1H, C(4)H), [2.50 (td, *J* = 16.4, 8.3, 1H) and 1.77 (ddd, *J* = 15.8, 7.1, 4.4, 1H)] (C(5)H_2), 2.05 (s, 3H, C_4-CH_3), 1.24 (d, *J* = 7.3, 3H, C_3-CH_3). ^{13}C NMR ($CDCl_3$) δ 169.6 ($^{2}J_{P\text{-}C}$ = 23.8, C_3), 134.3 ($^{1}J_{P\text{-}C}$ = 98.9, $C_{1'}$), 131.5 ($^{4}J_{P\text{-}C}$ = 2.8, $C_{4'}$), 130.7 ($^{2}J_{P\text{-}C}$ = 10.6, $C_{2'}$), 128.5 ($^{3}J_{P\text{-}C}$ = 12.0, $C_{3'}$), 120.3 ($^{1}J_{P\text{-}C}$ = 98.6, C_2), 40.3 ($^{2}J_{P\text{-}C}$ = 8.4, C_4), 36.2 ($^{1}J_{P\text{-}C}$ = 69.8, C_5), 20.3 ($^{3}J_{P\text{-}C}$ = 7.2, C_4-CH_3), 19.0 ($^{3}J_{P\text{-}C}$ = 18.6, C_3-CH_3). HRMS $[M+H]^{+}_{found}$ = 207.0930, $C_{12}H_{16}OP$ requires 207.0941.

^{31}P, ^{1}H and ^{13}C NMR spectra of the compounds 1d-f, 3c, 3h, 4, 7 and 10 prepared

1-(4-Triflourmethyl-phenyl)-3-methyl-3-phospholene oxide (**1d**):

^{19}F NMR

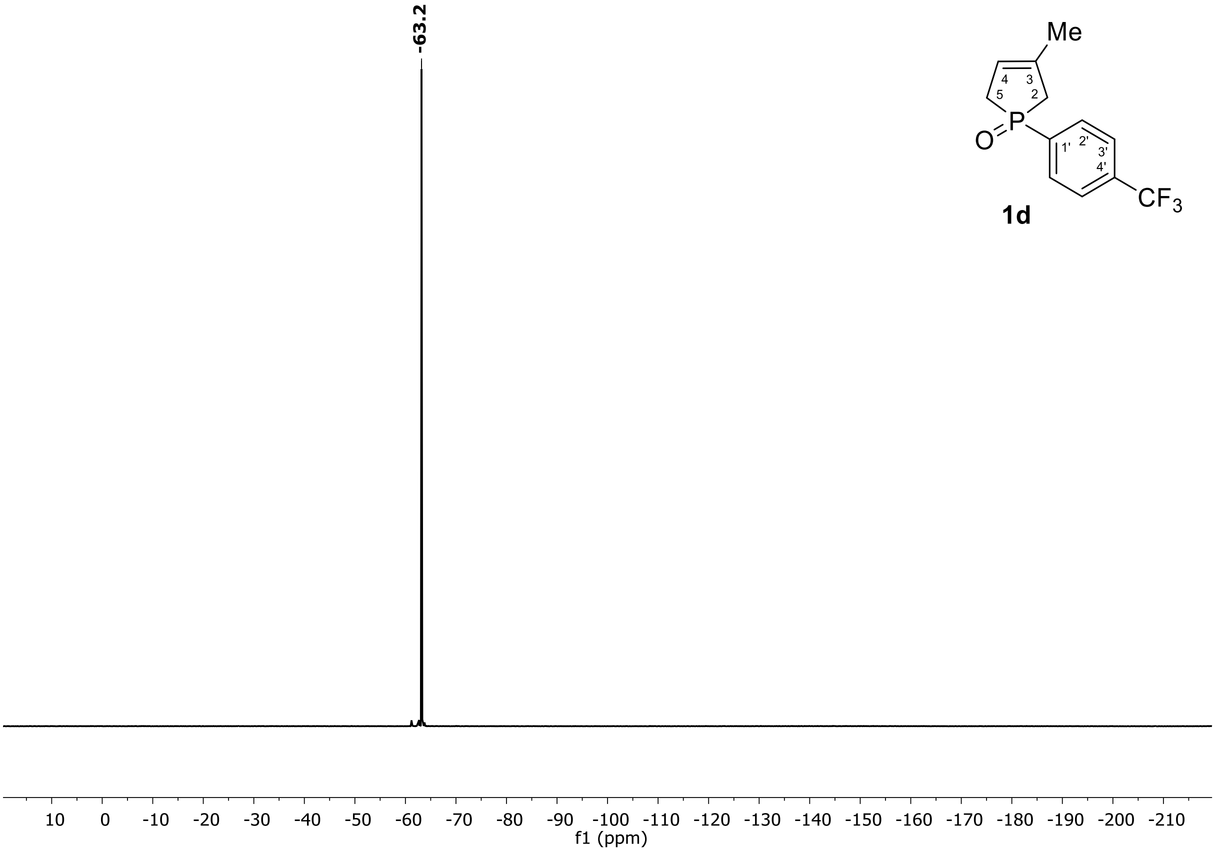

^{31}P NMR

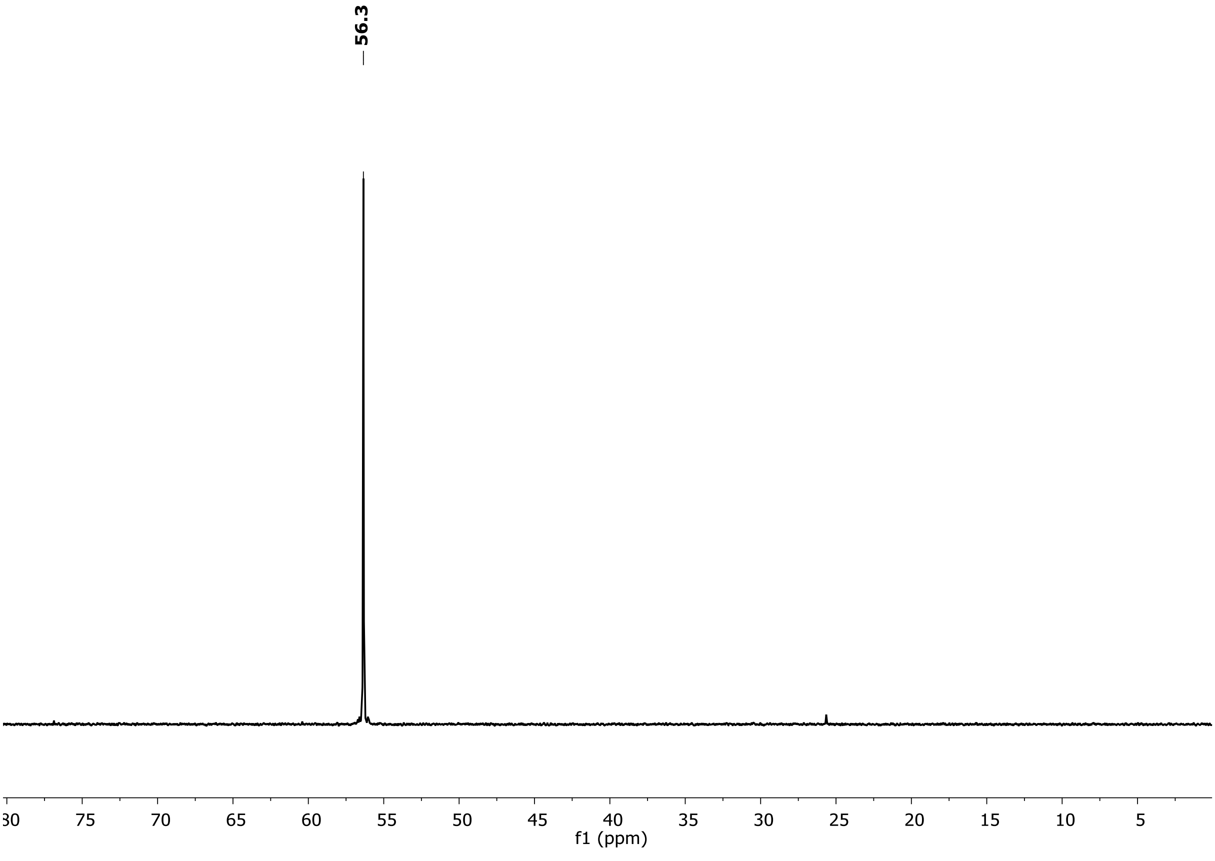

^{1}H NMR

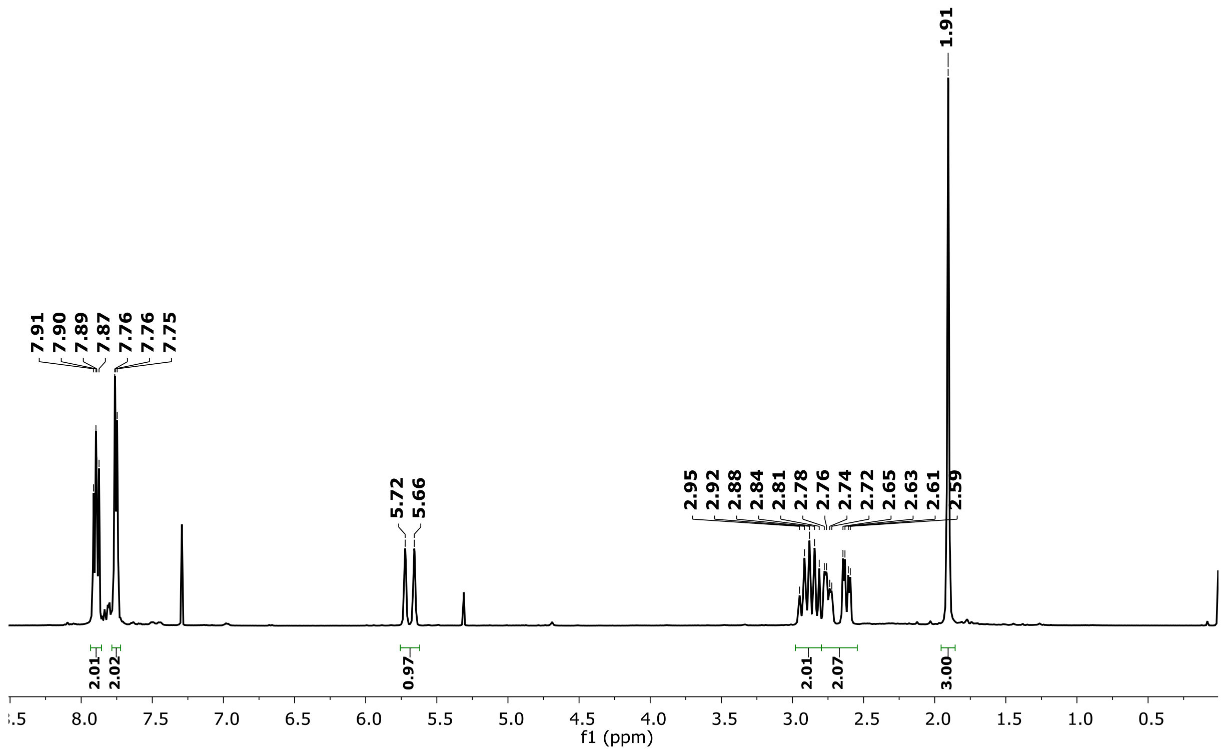

^{13}C NMR

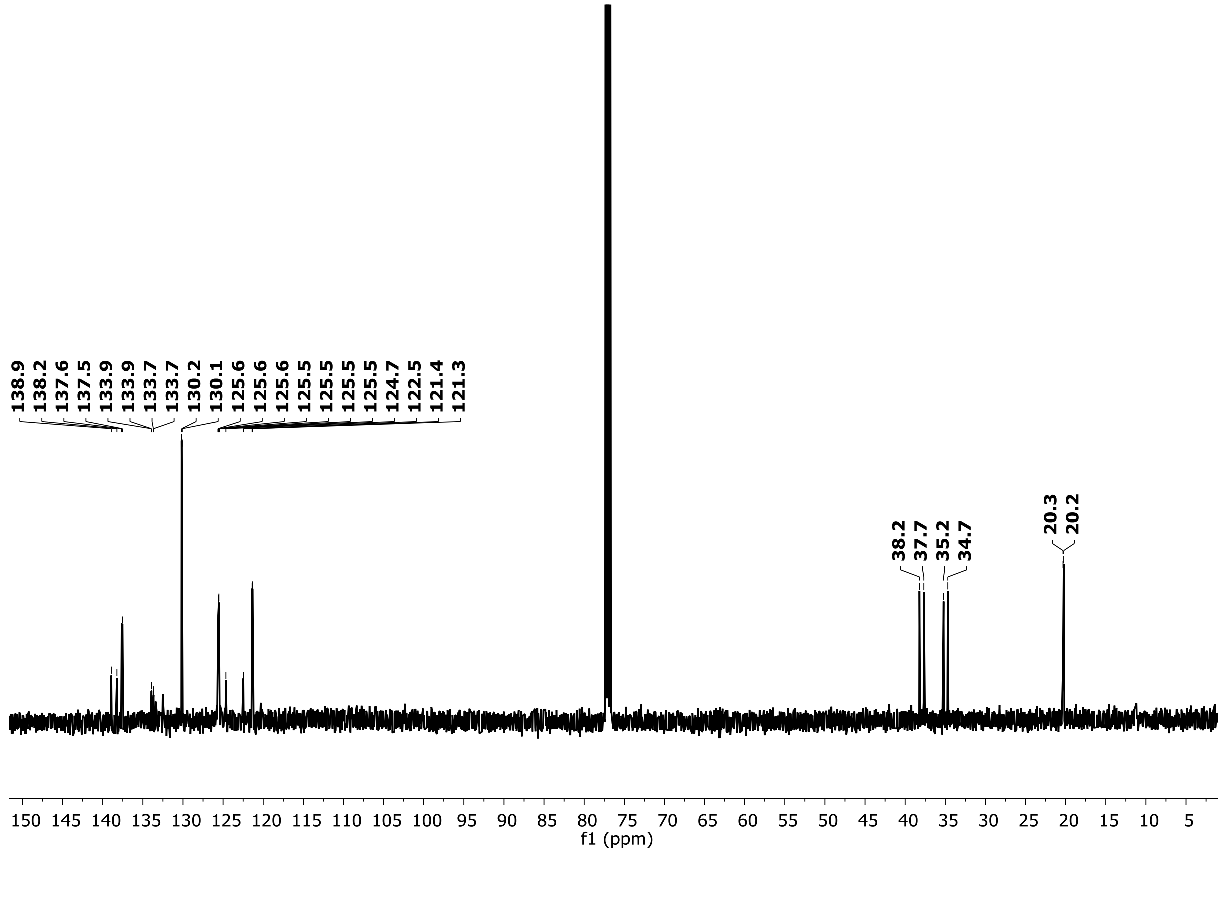

1-(4-Methoxyphenyl)-3-methyl-3-phospholene oxide (**1e**):

^{31}P NMR

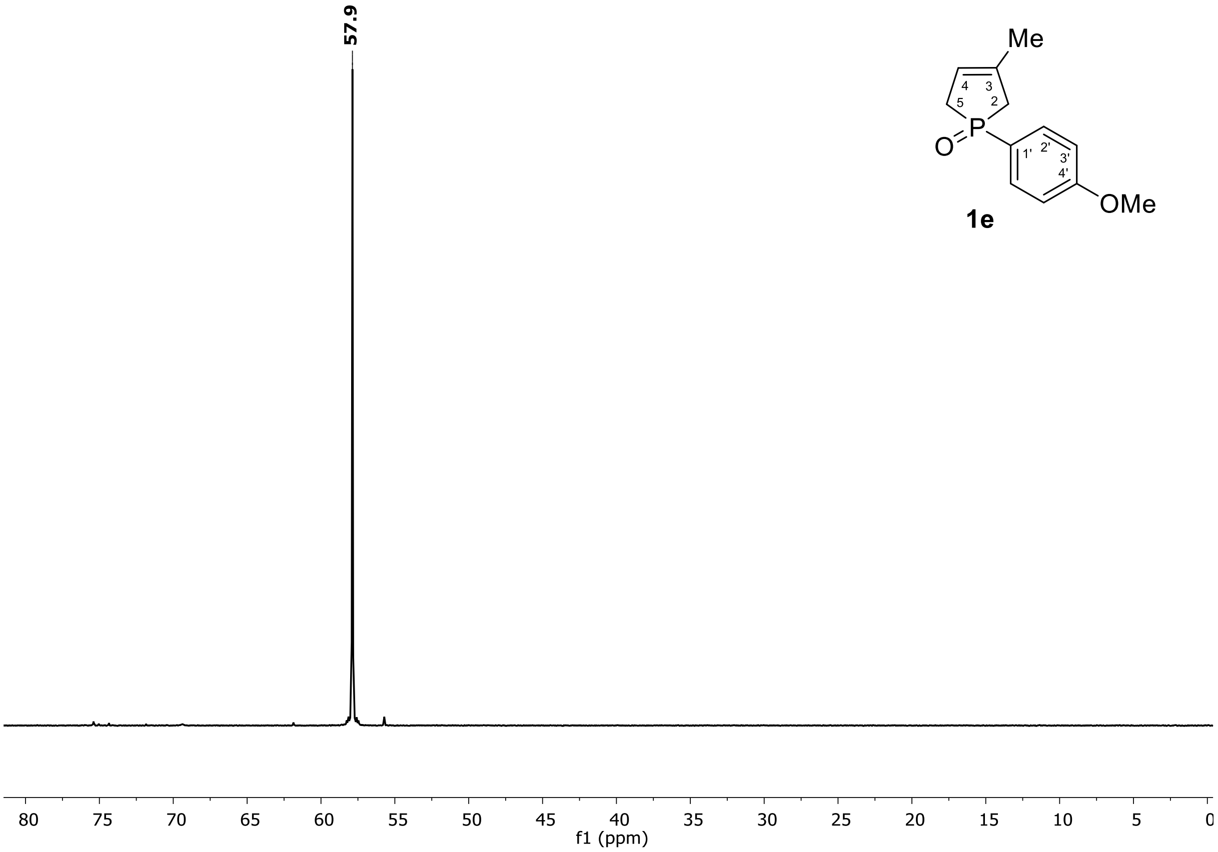

^{1}H NMR

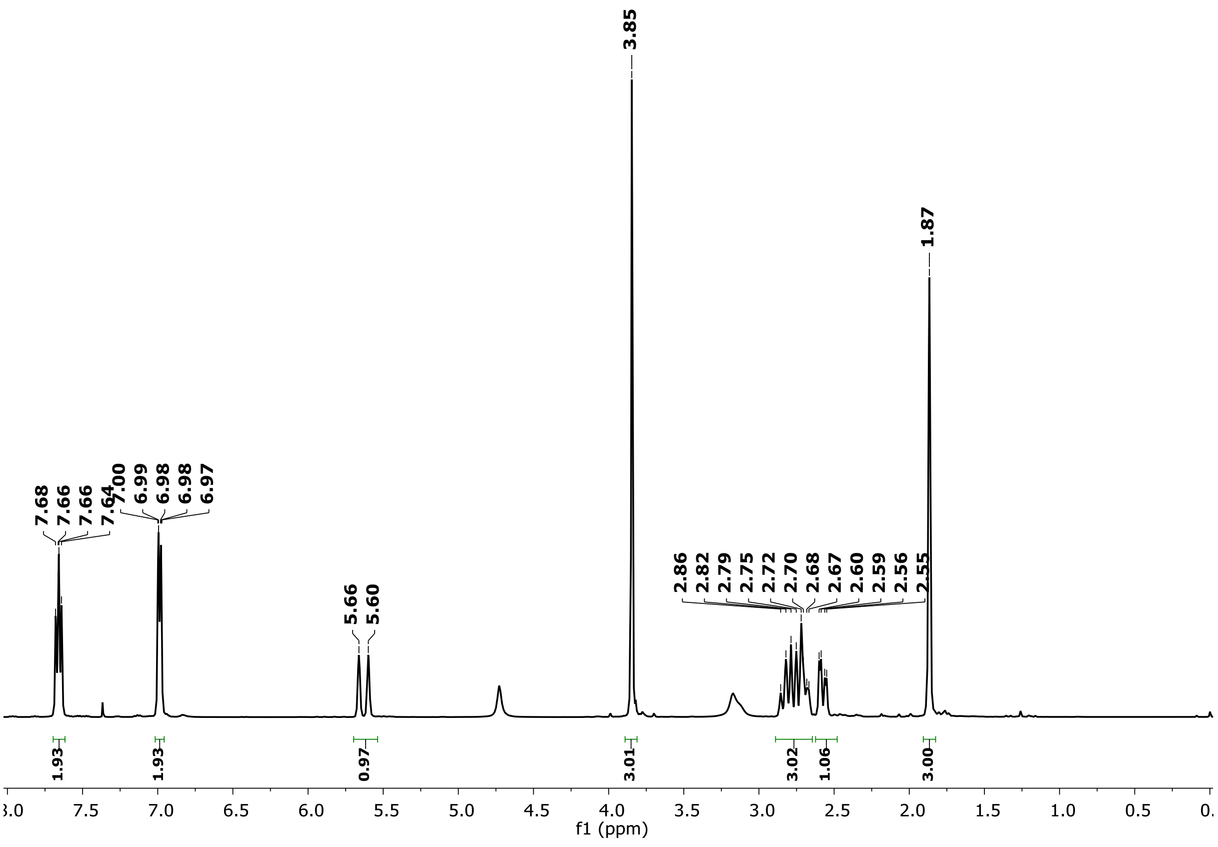

^{13}C NMR

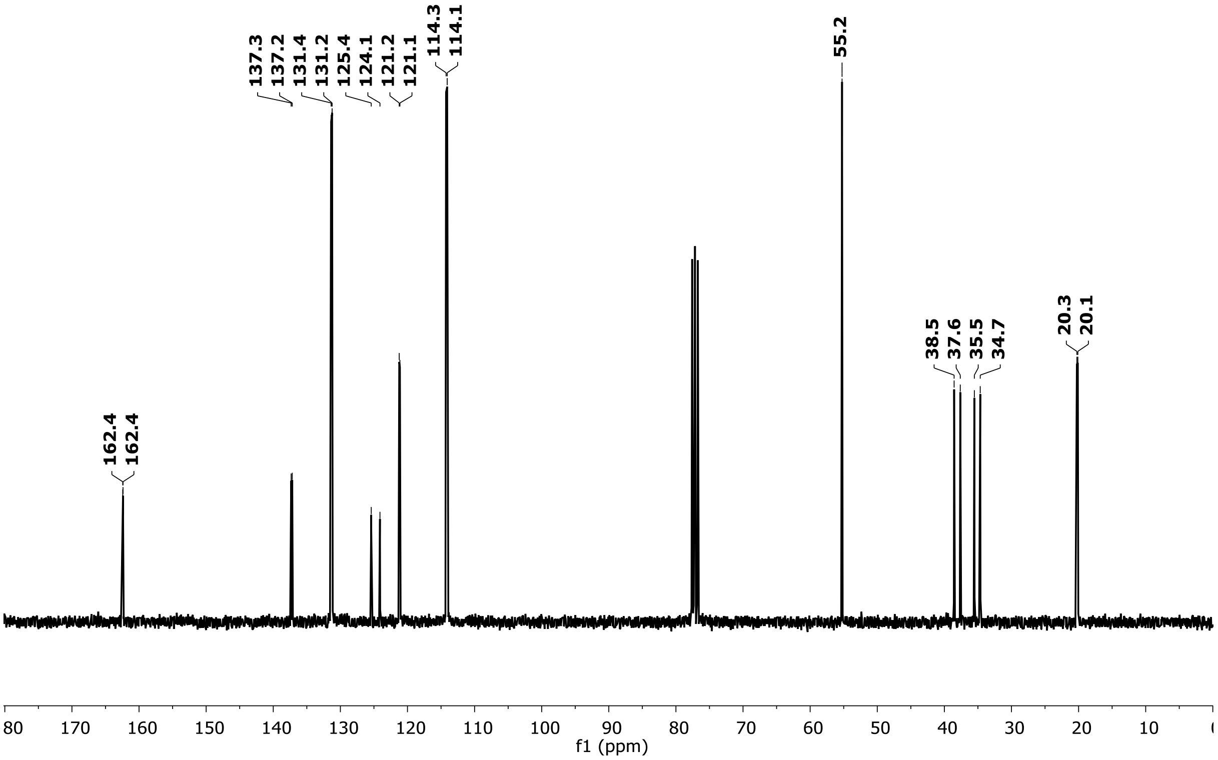
162.4
162.4
137.3
137.2
131.4
131.2
125.4
124.1
121.2
121.1
114.3
114.1
55.2
38.5
37.6
35.5
34.7
20.3
20.1
f1 (ppm)

1-(2,6-Dimethylphenyl)-3-methyl-3-phospholene oxide (**1f**):

^{31}P NMR

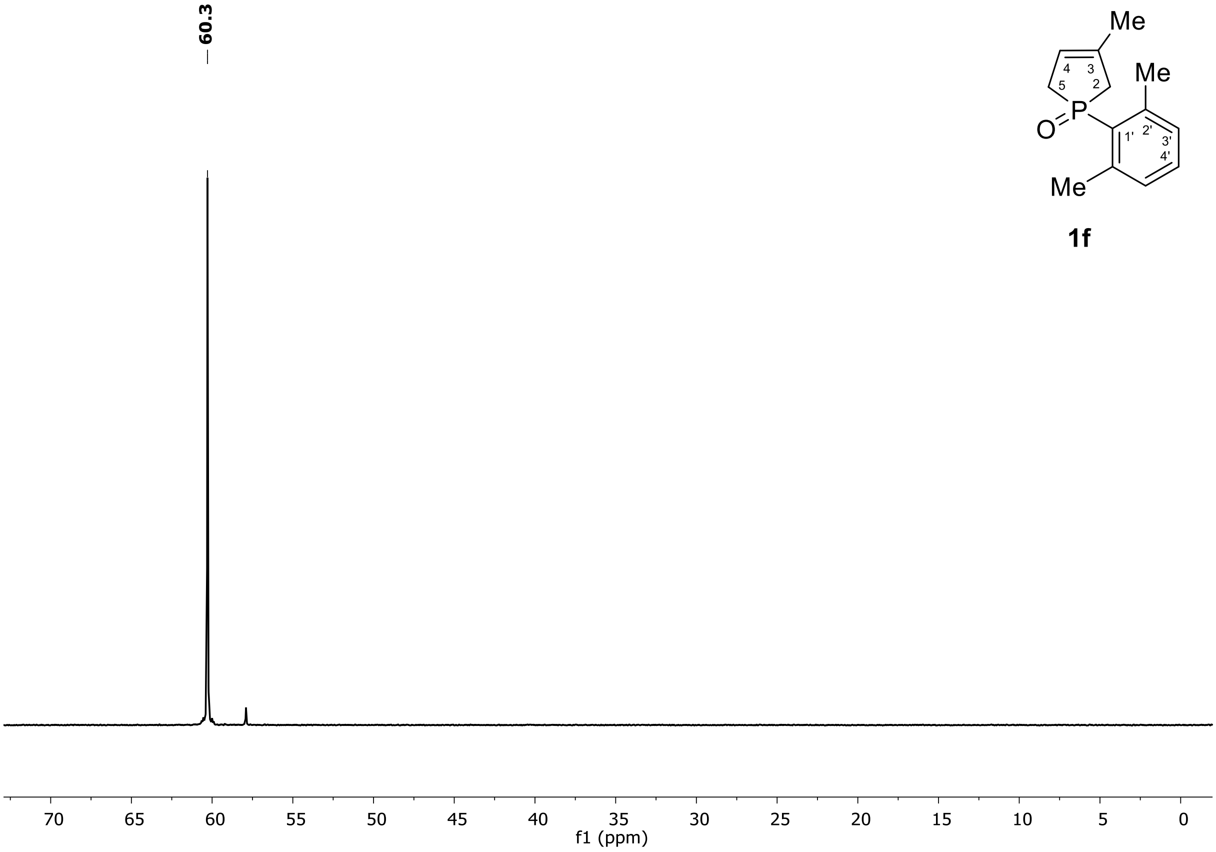

^{1}H NMR

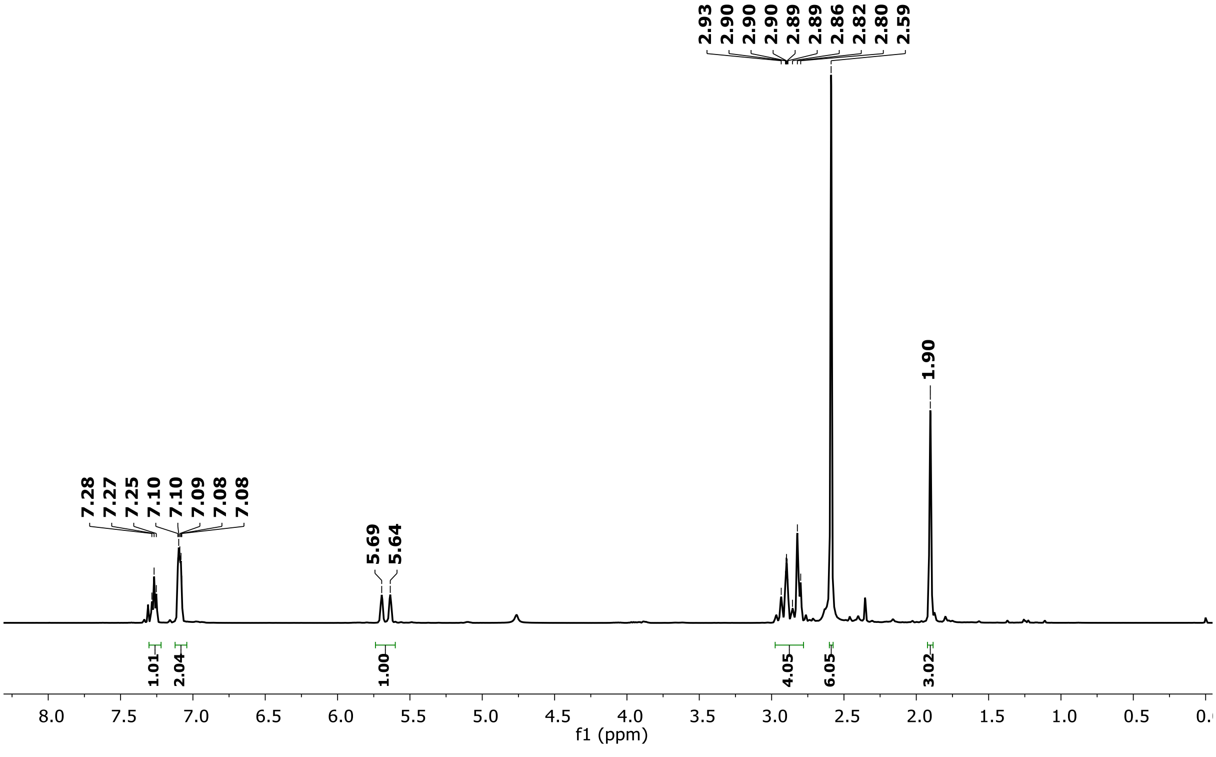

^{13}C NMR

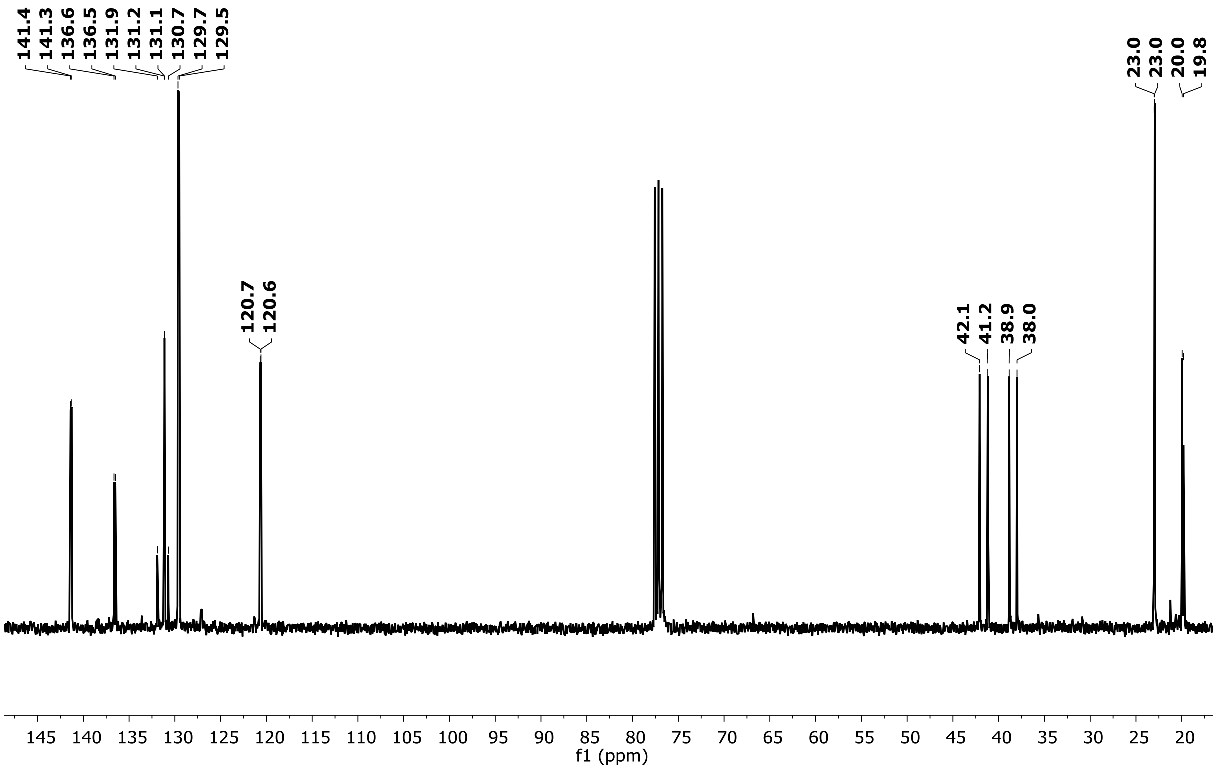

141.4
141.3
136.6
136.5
131.9
131.2
131.1
130.7
129.7
129.5
120.7
120.6
42.1
41.2
38.9
38.0
23.0
23.0
20.0
19.8
145 140 135 130 125 120 115 110 105 100 95 90 85 80 75 70 65 60 55 50 45 40 35 30 25 20
f1 (ppm)

1-Chloro-3-methyl-1-(4-methylphenyl)-2-phospholenium chloride (**3c**)

^{31}P NMR

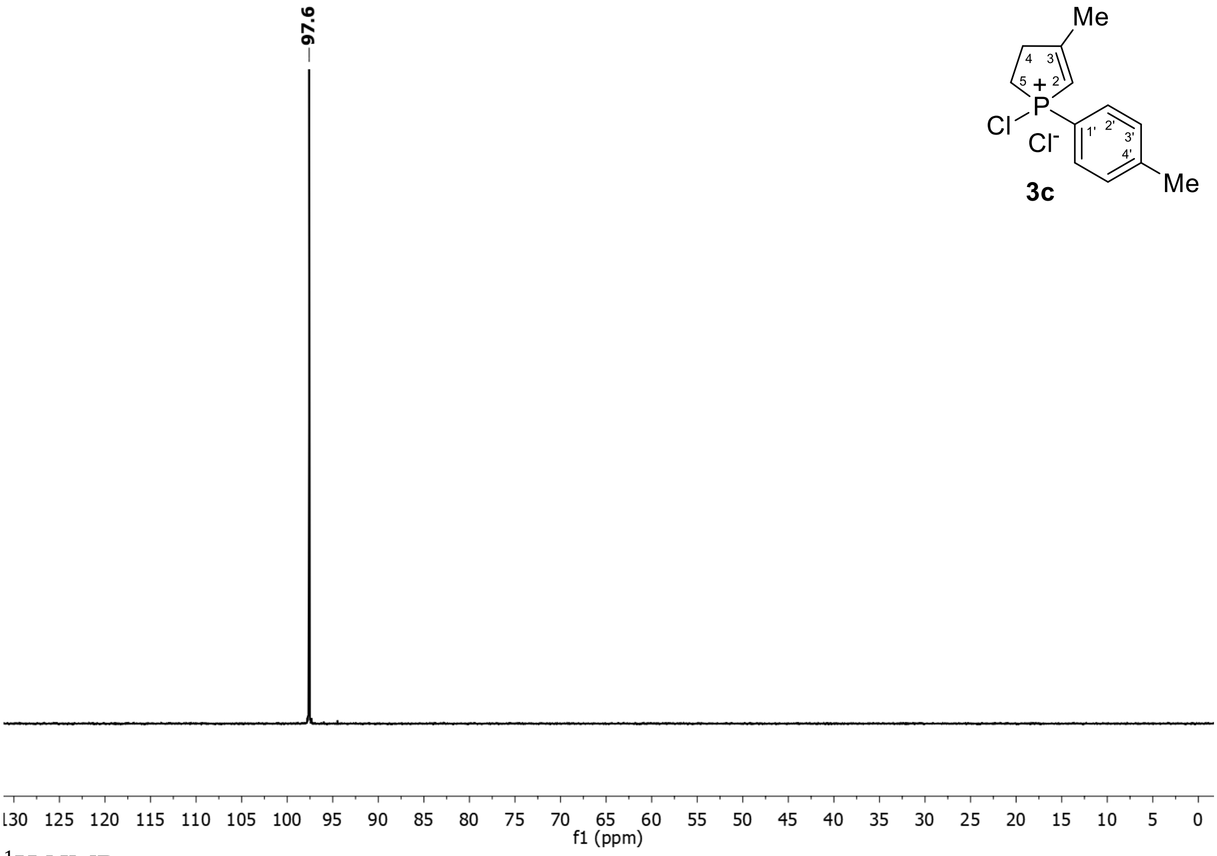

^{1}H NMR

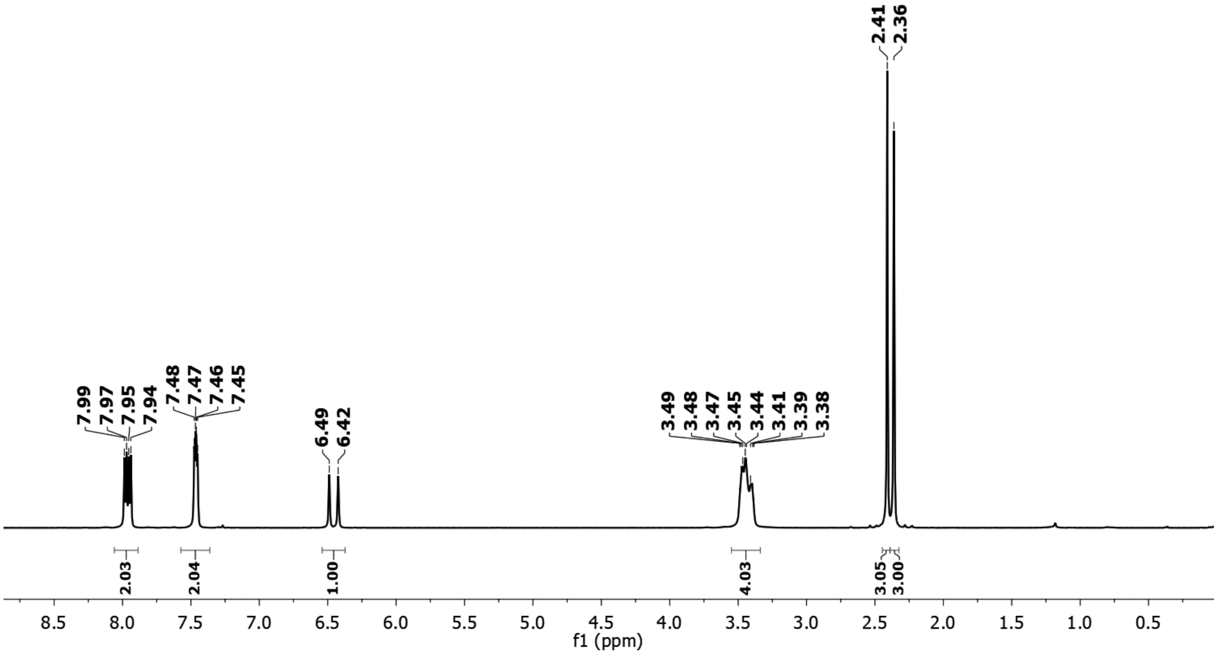

^{13}C NMR

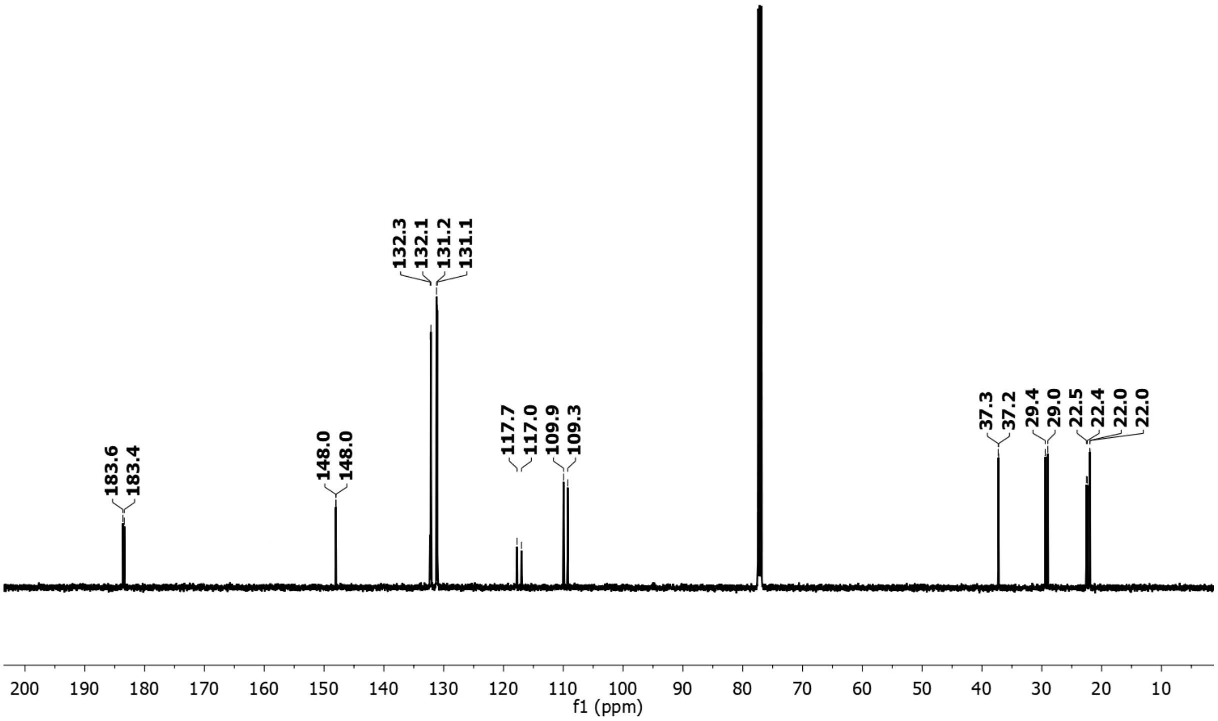
183.6
183.4
148.0
148.0
132.3
132.1
131.2
131.1
117.7
117.0
109.9
109.3
37.3
37.2
29.4
29.0
22.5
22.4
22.0
22.0
200 190 180 170 160 150 140 130 120 110 100 90 80 70 60 50 40 30 20 10
f1 (ppm)

1-Chloro-1-ethyl-3-methyl-2-phospholenium chloride (**3h**)

^{31}P NMR

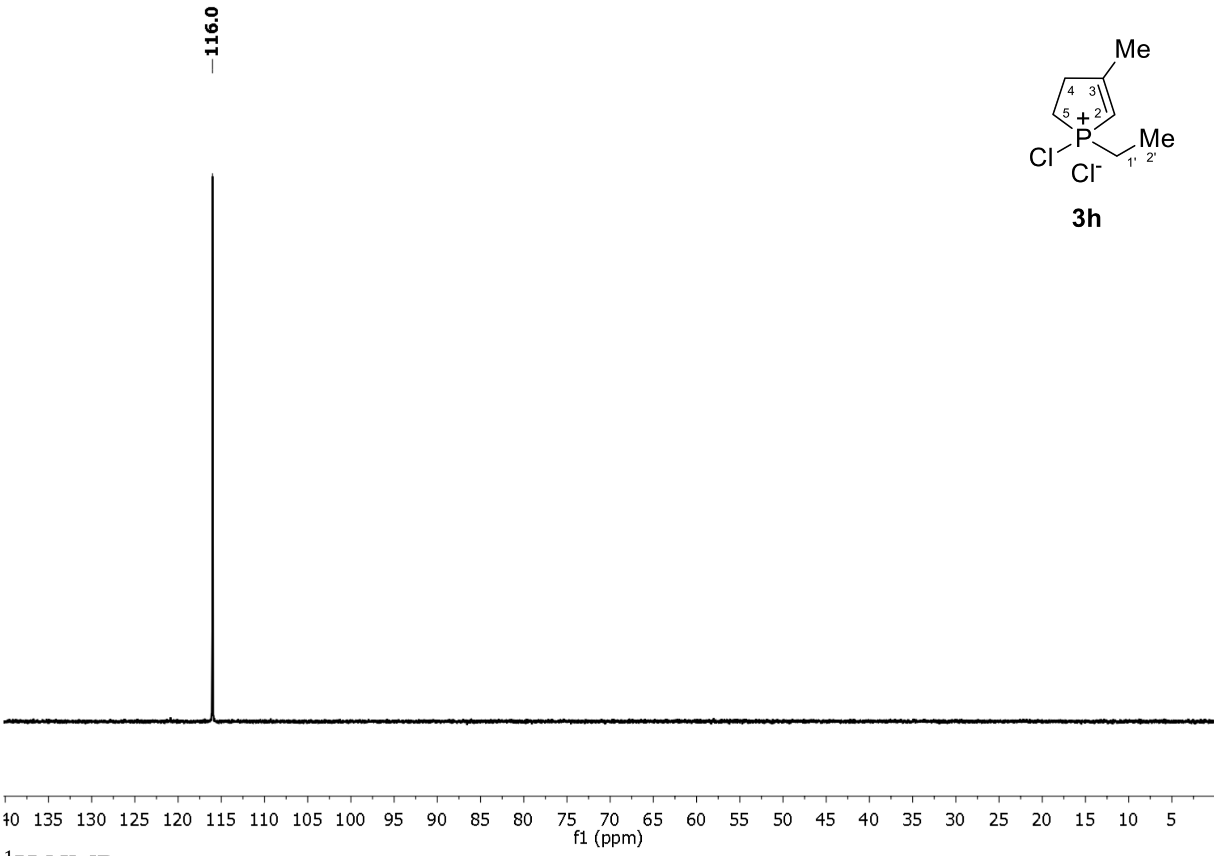

^{1}H NMR

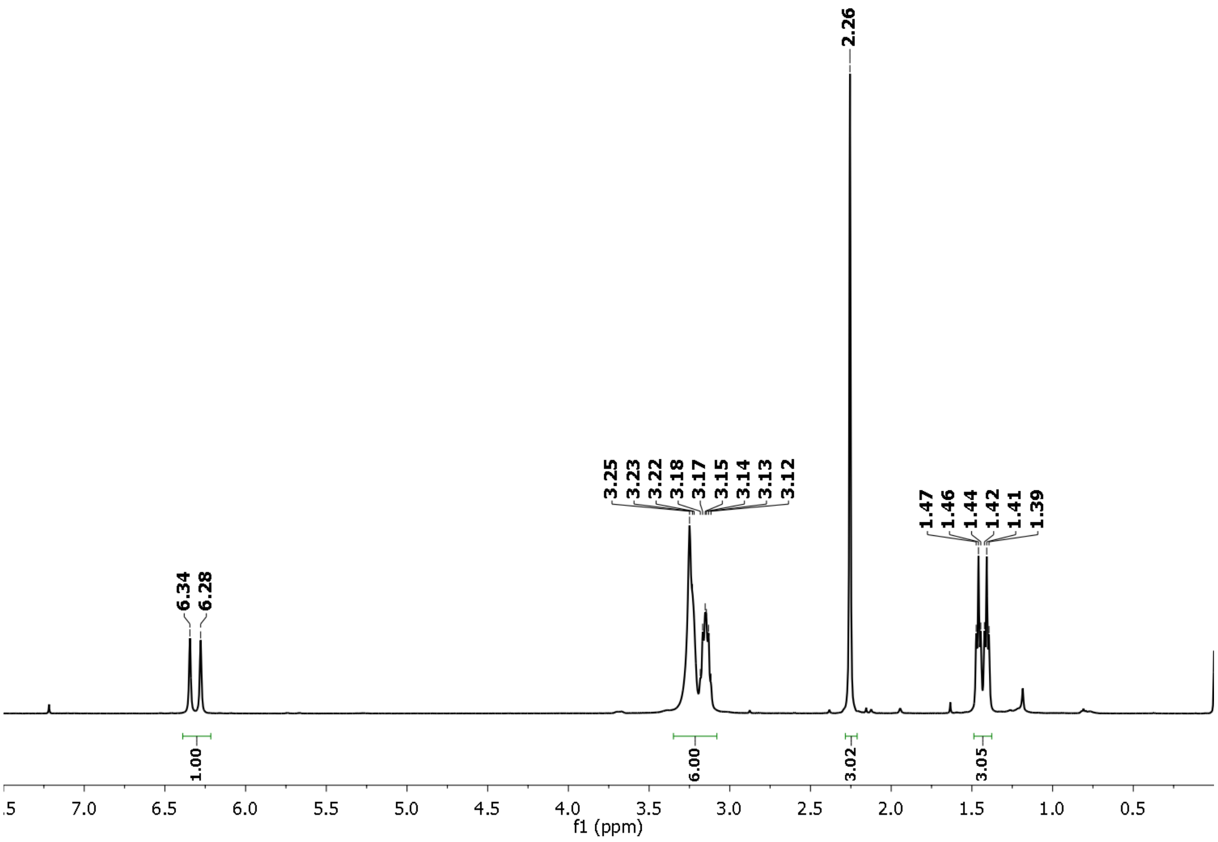

^{13}C NMR

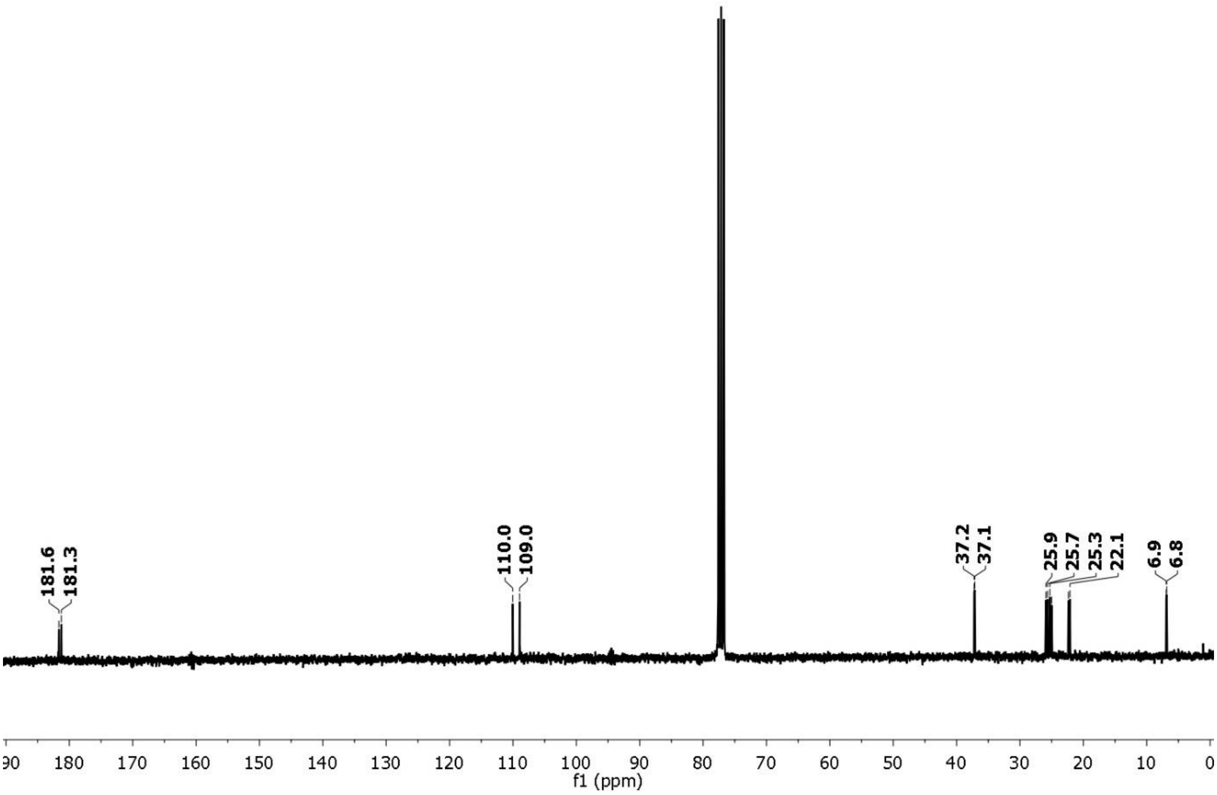
181.6
181.3
110.0
109.0
37.2
37.1
25.9
25.7
25.3
22.1
6.9
6.8
90 180 170 160 150 140 130 120 110 100 90 80 70 60 50 40 30 20 10 0
f1 (ppm)

1-Phenyl-3-methyl-2-phospholene oxide (**4a**):

^{31}P NMR

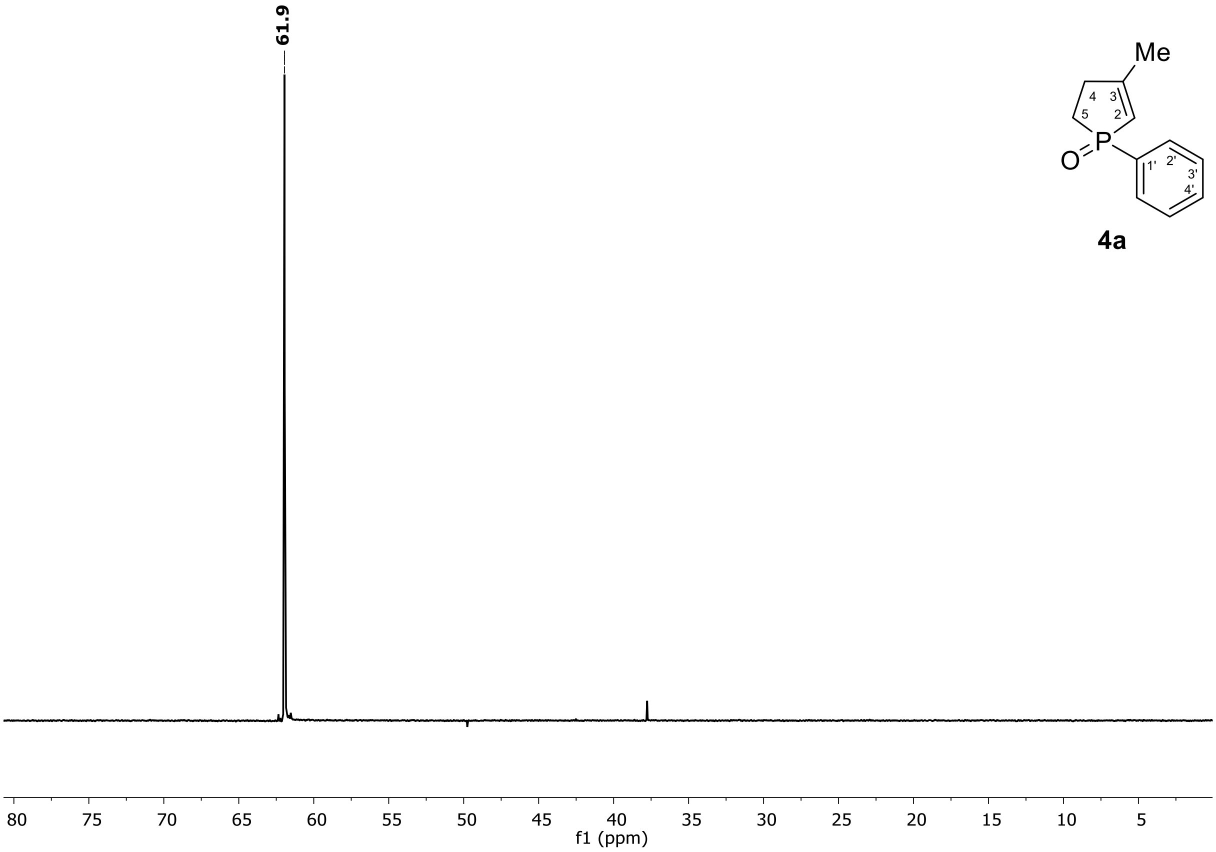

4a

^{1}H NMR

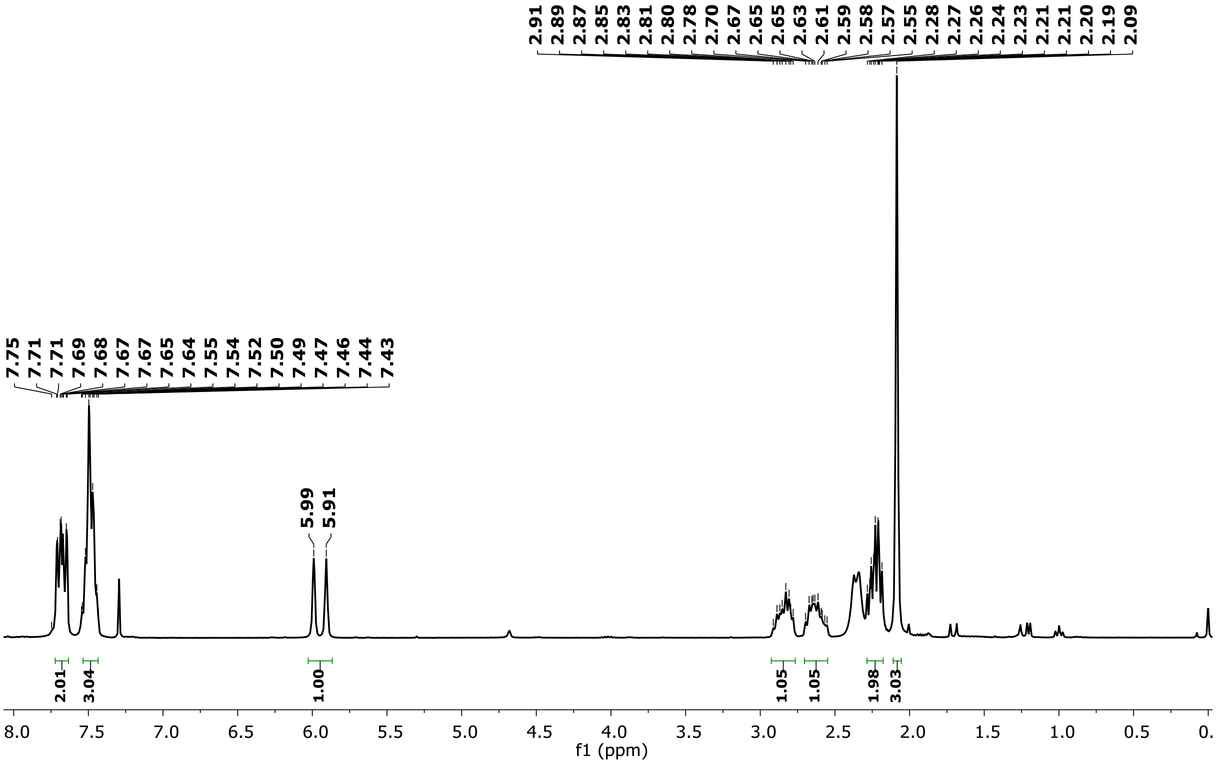

^{13}C NMR

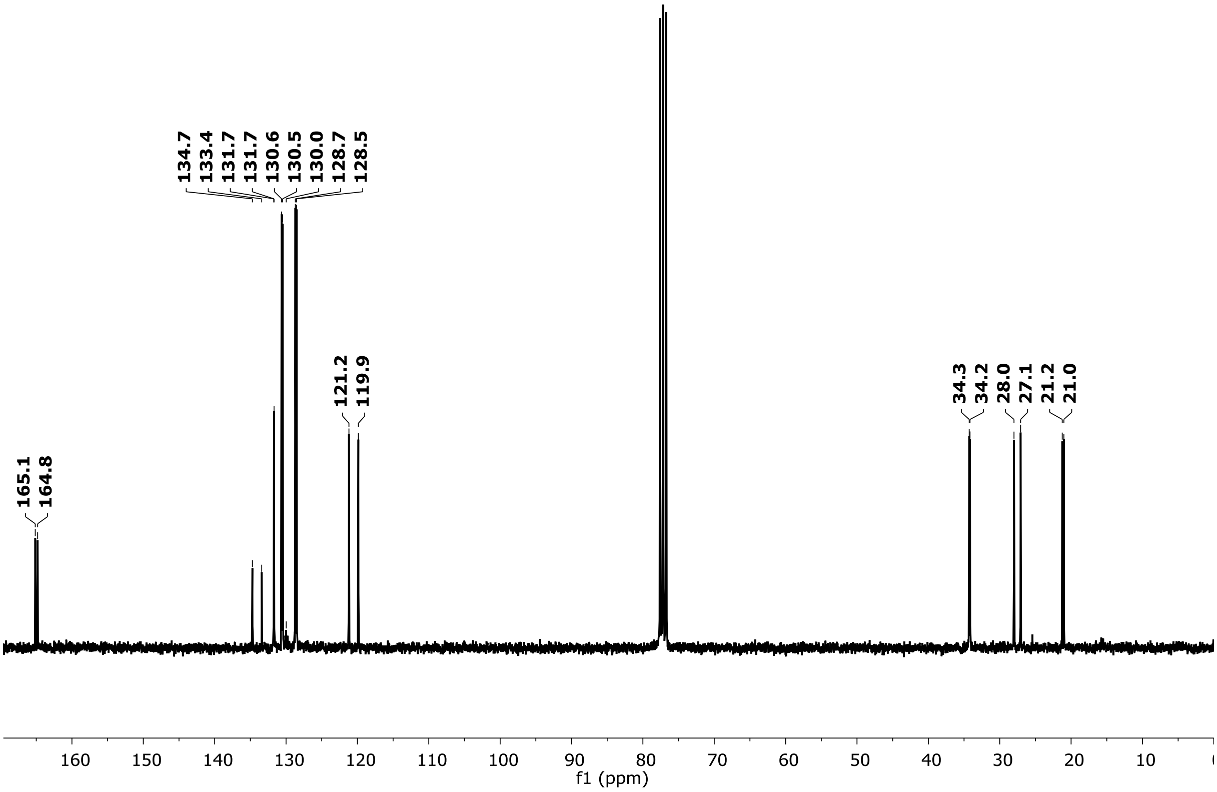

134.7
133.4
131.7
131.7
130.6
130.5
130.0
128.7
128.5
121.2
119.9
165.1
164.8
34.3
34.2
28.0
27.1
21.2
21.0
160
150
140
130
120
110
100
90
80
70
60
50
40
30
20
10
f1 (ppm)

1-(2-Methylphenyl)-3-methyl-2-phospholene oxide (**4b**):

^{31}P NMR

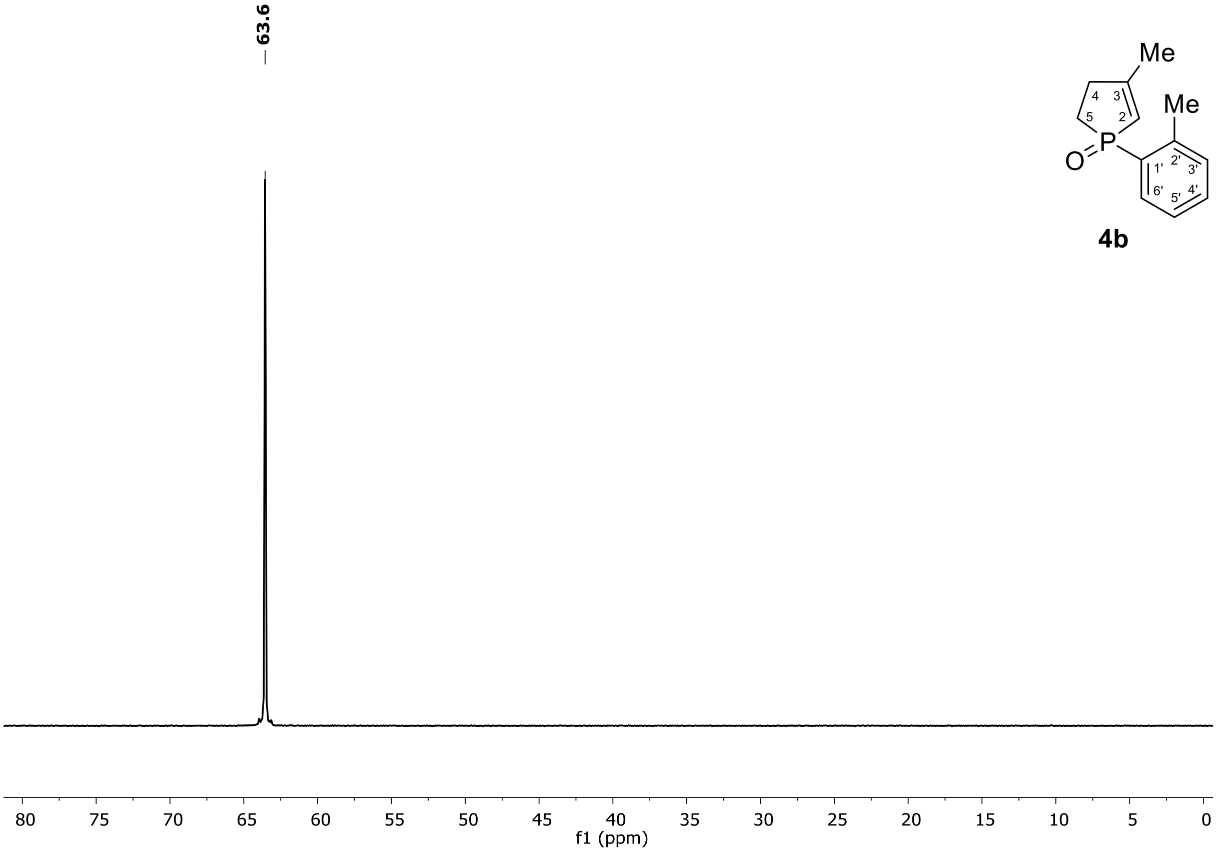

4b

^{1}H NMR

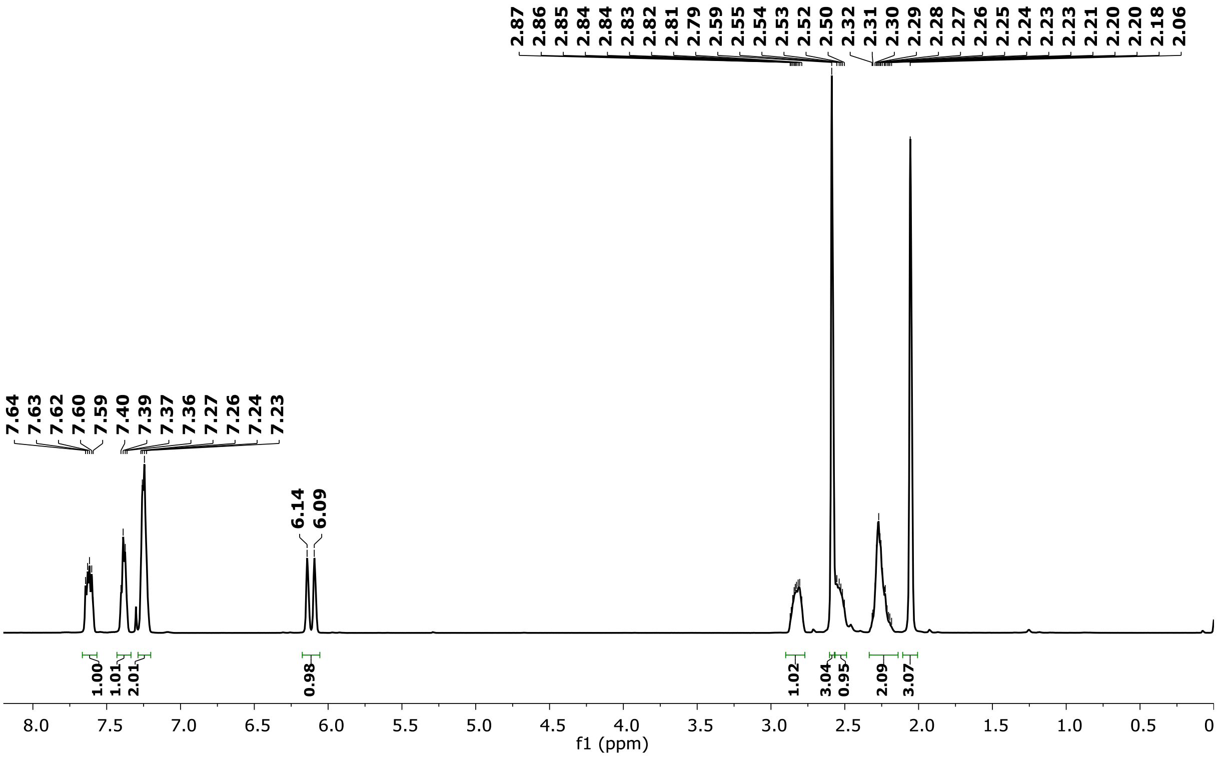

^{13}C NMR

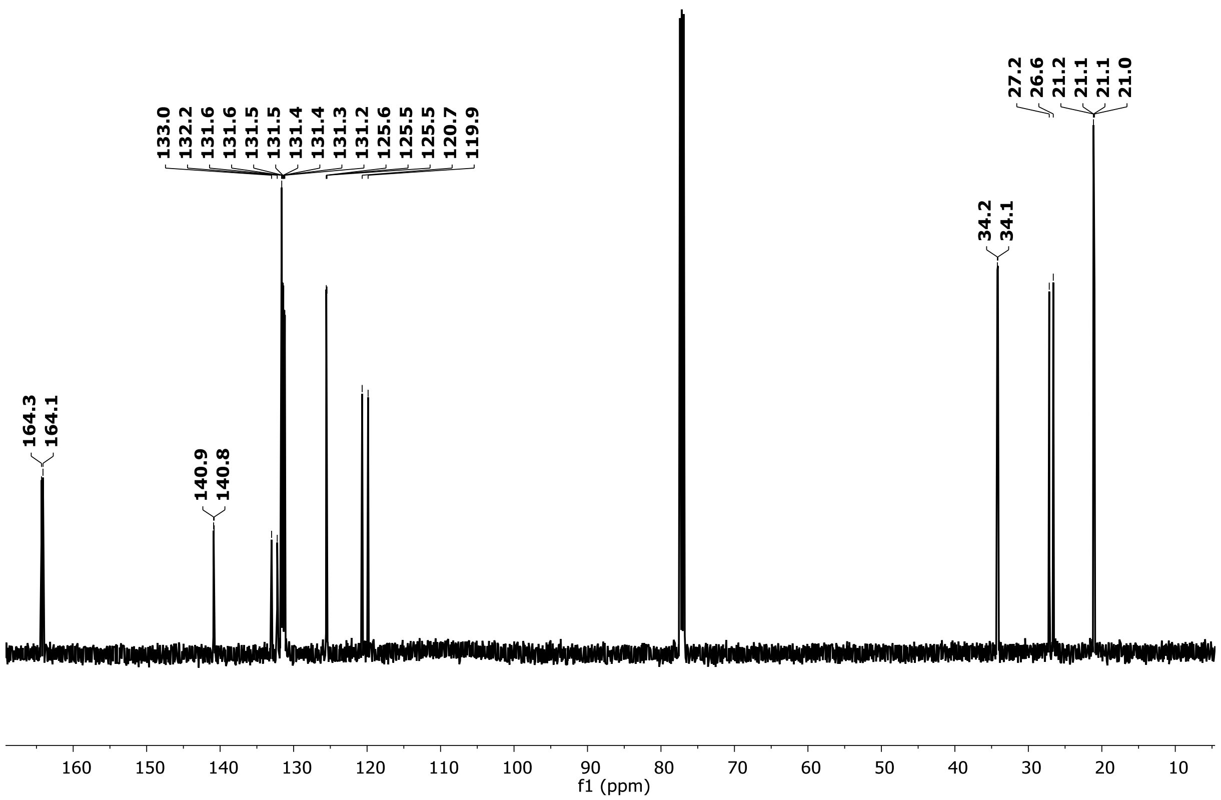

133.0
132.2
131.6
131.6
131.5
131.5
131.4
131.4
131.3
131.2
125.6
125.5
125.5
120.7
119.9
27.2
26.6
21.2
21.1
21.1
21.0
34.2
34.1
164.3
164.1
140.9
140.8
160 150 140 130 120 110 100 90 80 70 60 50 40 30 20 10
f1 (ppm)

1-(4-Methylphenyl)-3-methyl-2-phospholene oxide (**4c**):

^{31}P NMR

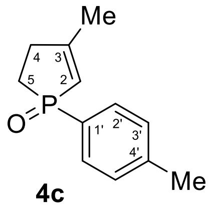

4c

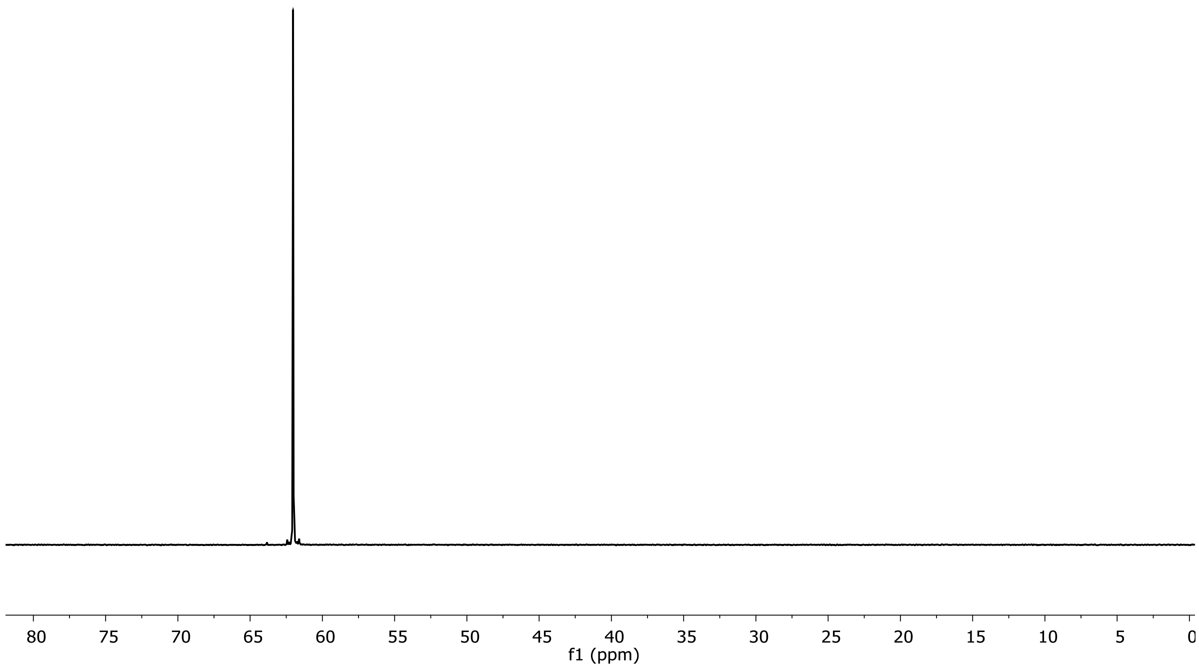

^{1}H NMR

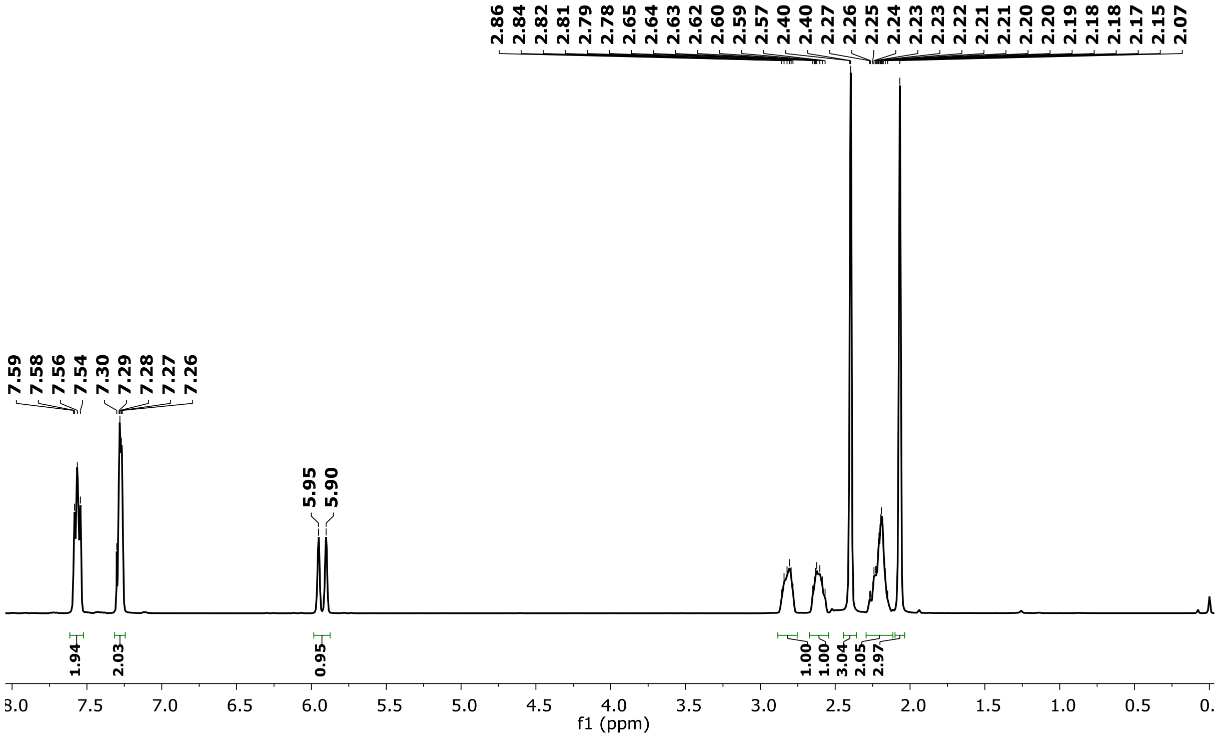

^{13}C NMR

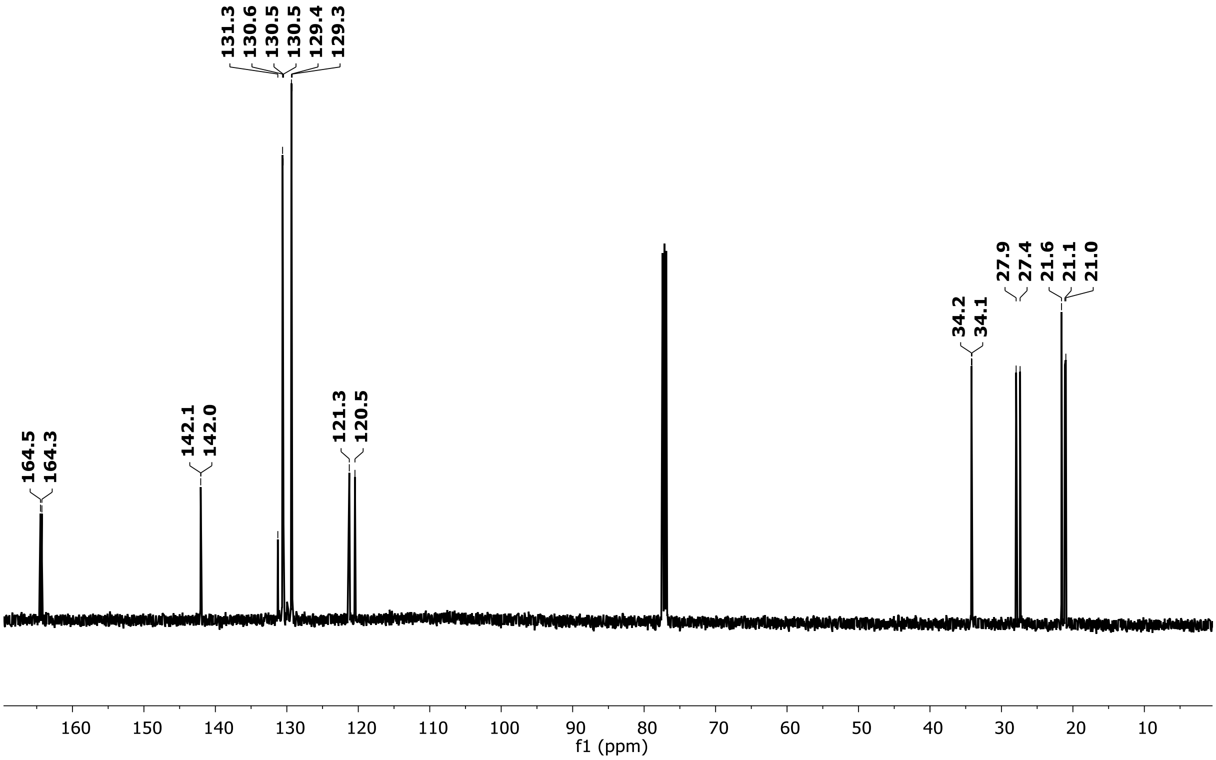

131.3
130.6
130.5
130.5
129.4
129.3
164.5
164.3
142.1
142.0
121.3
120.5
34.2
34.1
27.9
27.4
21.6
21.1
21.0
160
150
140
130
120
110
100
90
80
70
60
50
40
30
20
10
f1 (ppm)

1-(4-Trifluoromethyl-phenyl)-3-methyl-2-phospholene oxide (**4d**):

^{19}F NMR

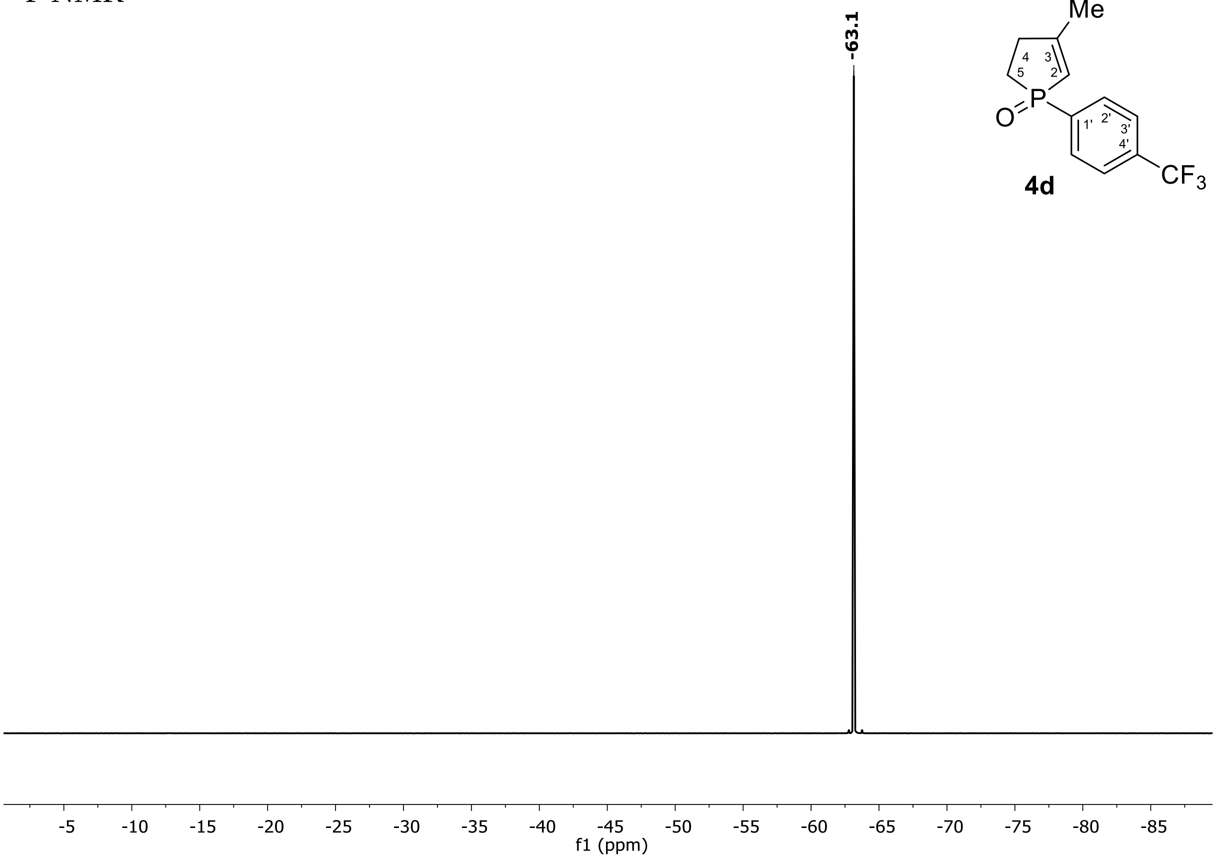

^{31}P NMR

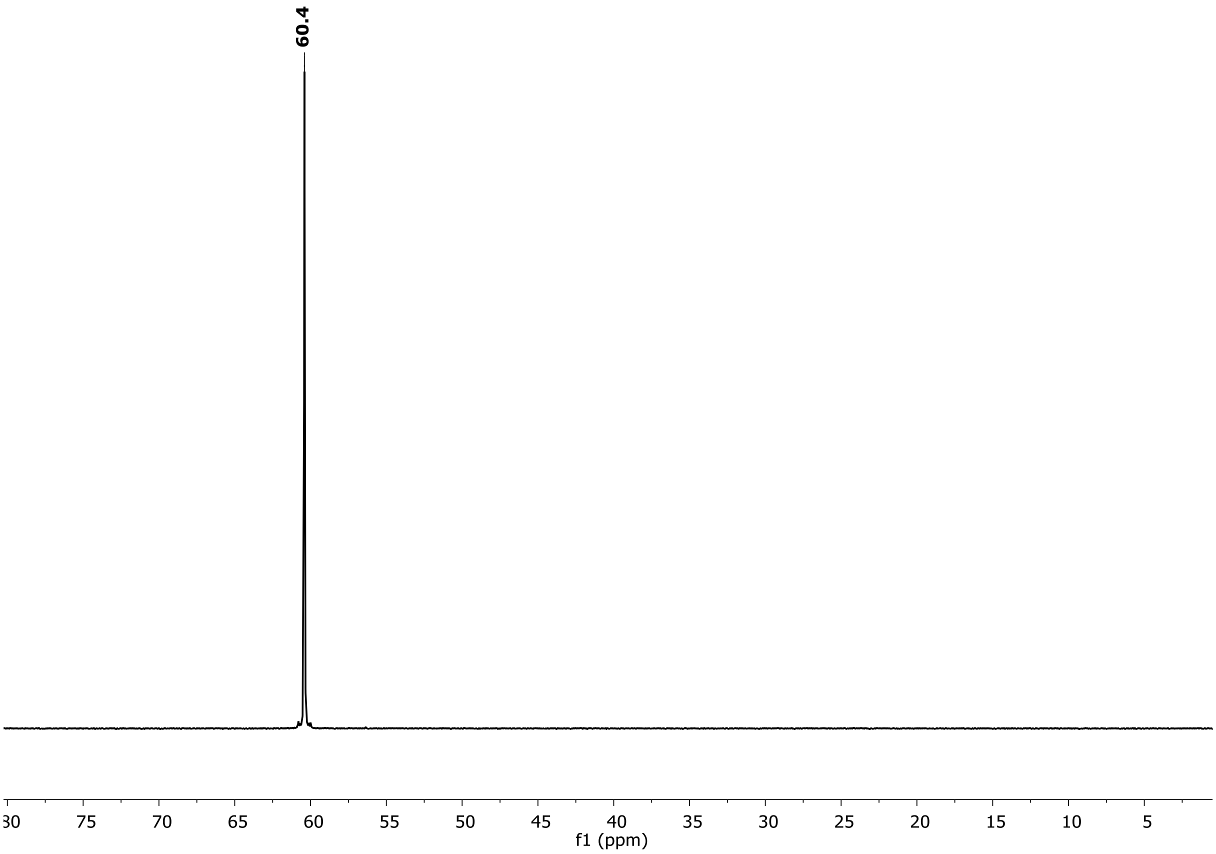

^{1}H NMR

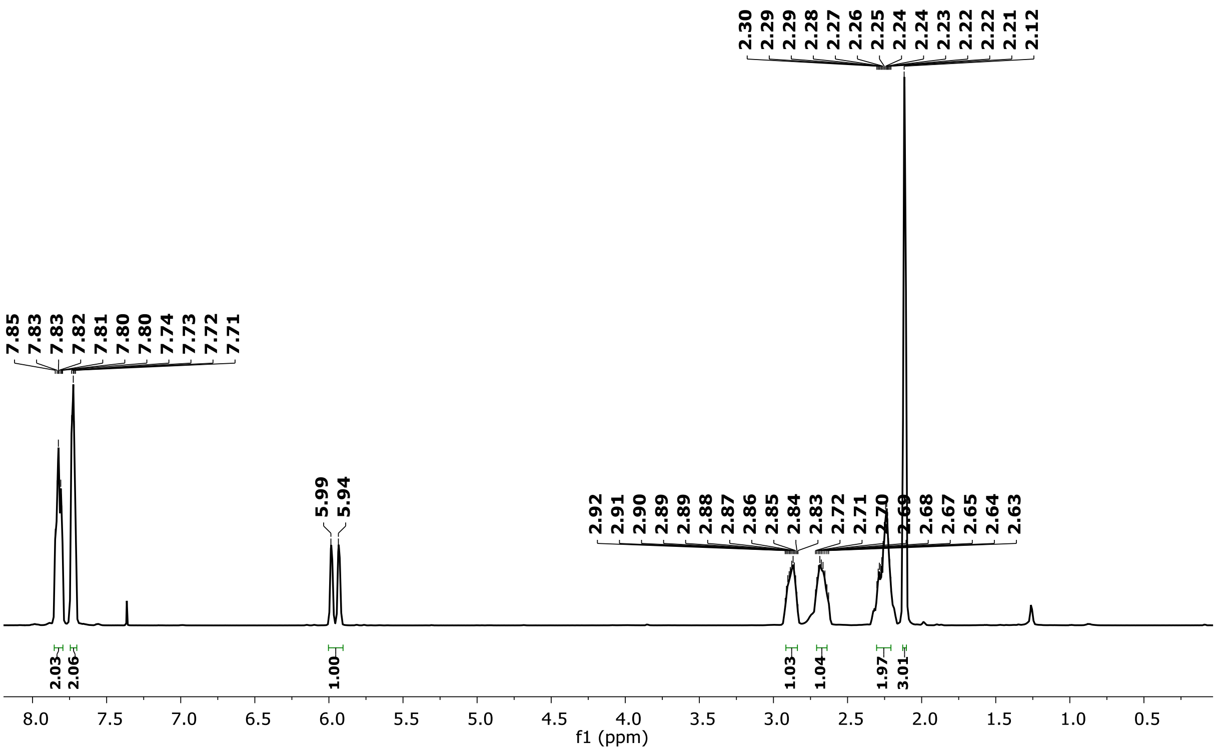

^{13}C NMR

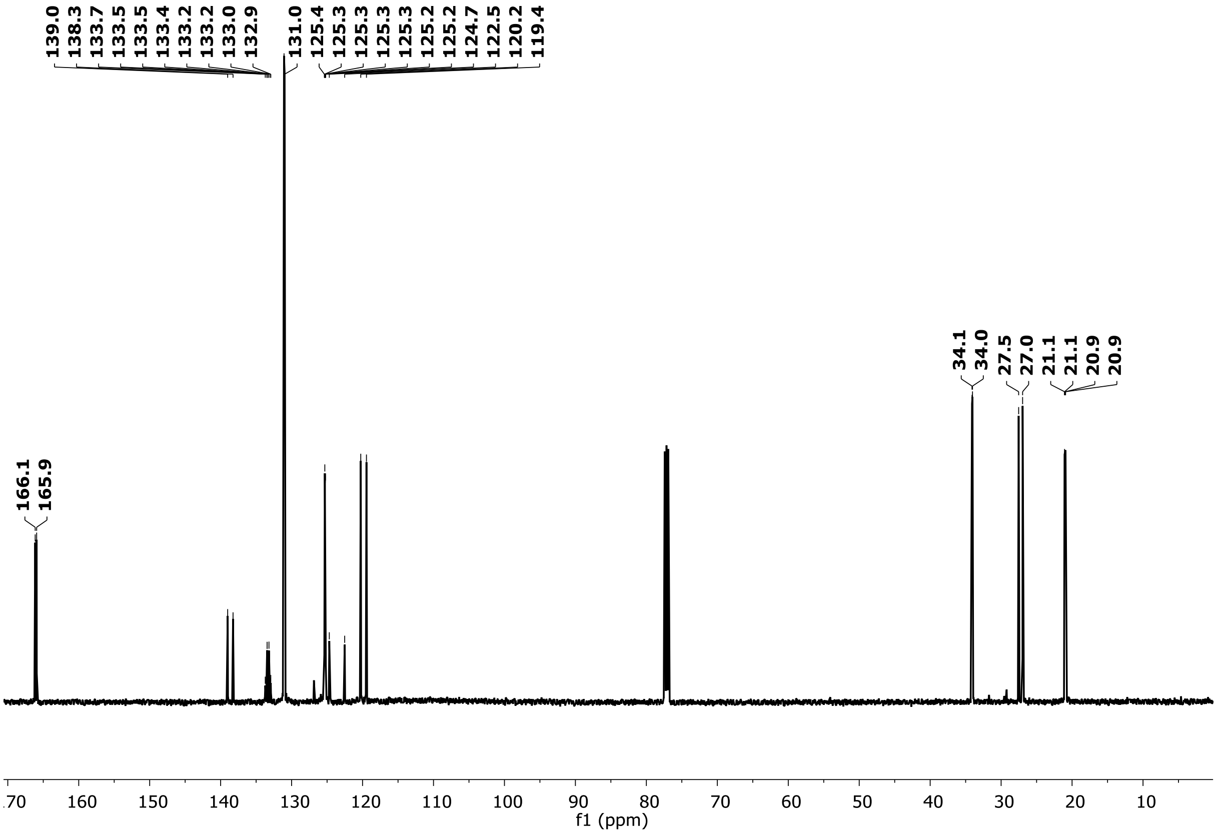

1-(4-Methoxyphenyl)-3-methyl-2-phospholene oxide (**4e**):

^{31}P NMR

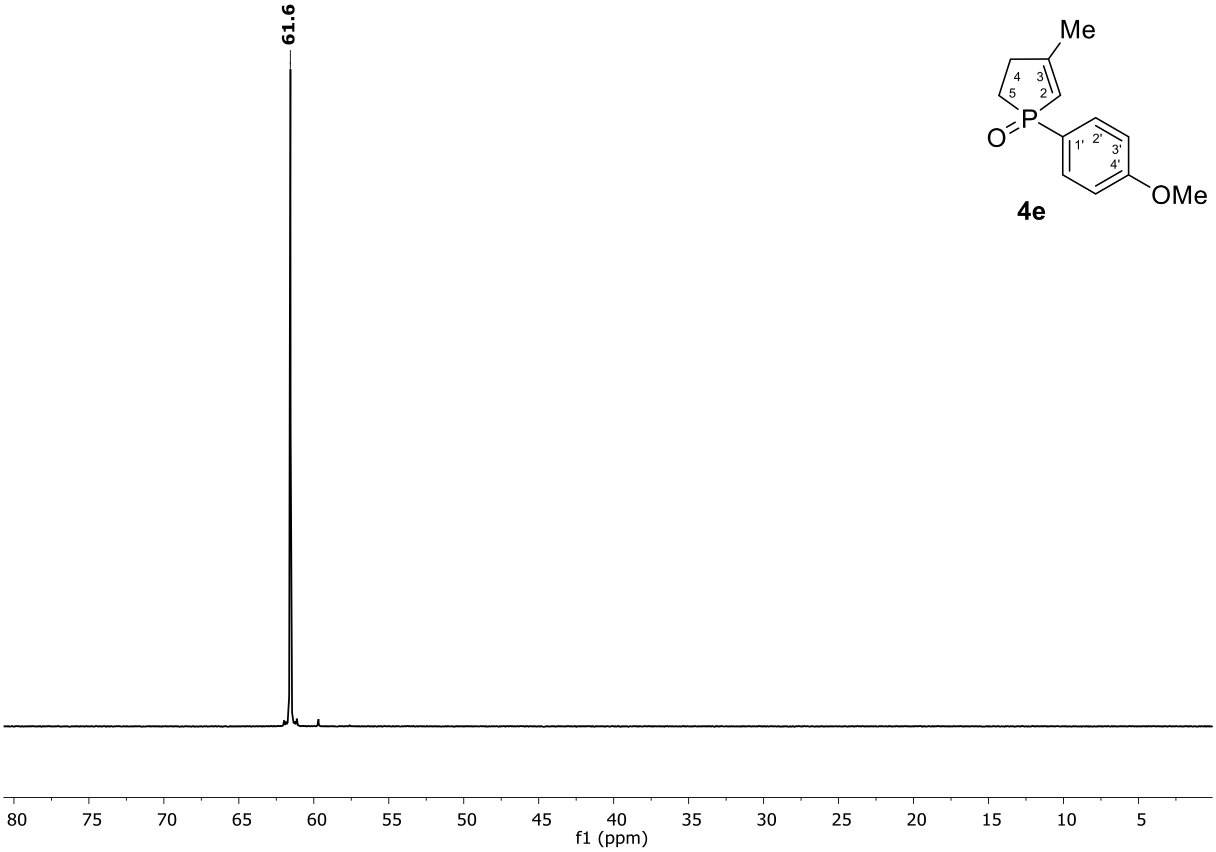

4e

^{1}H NMR

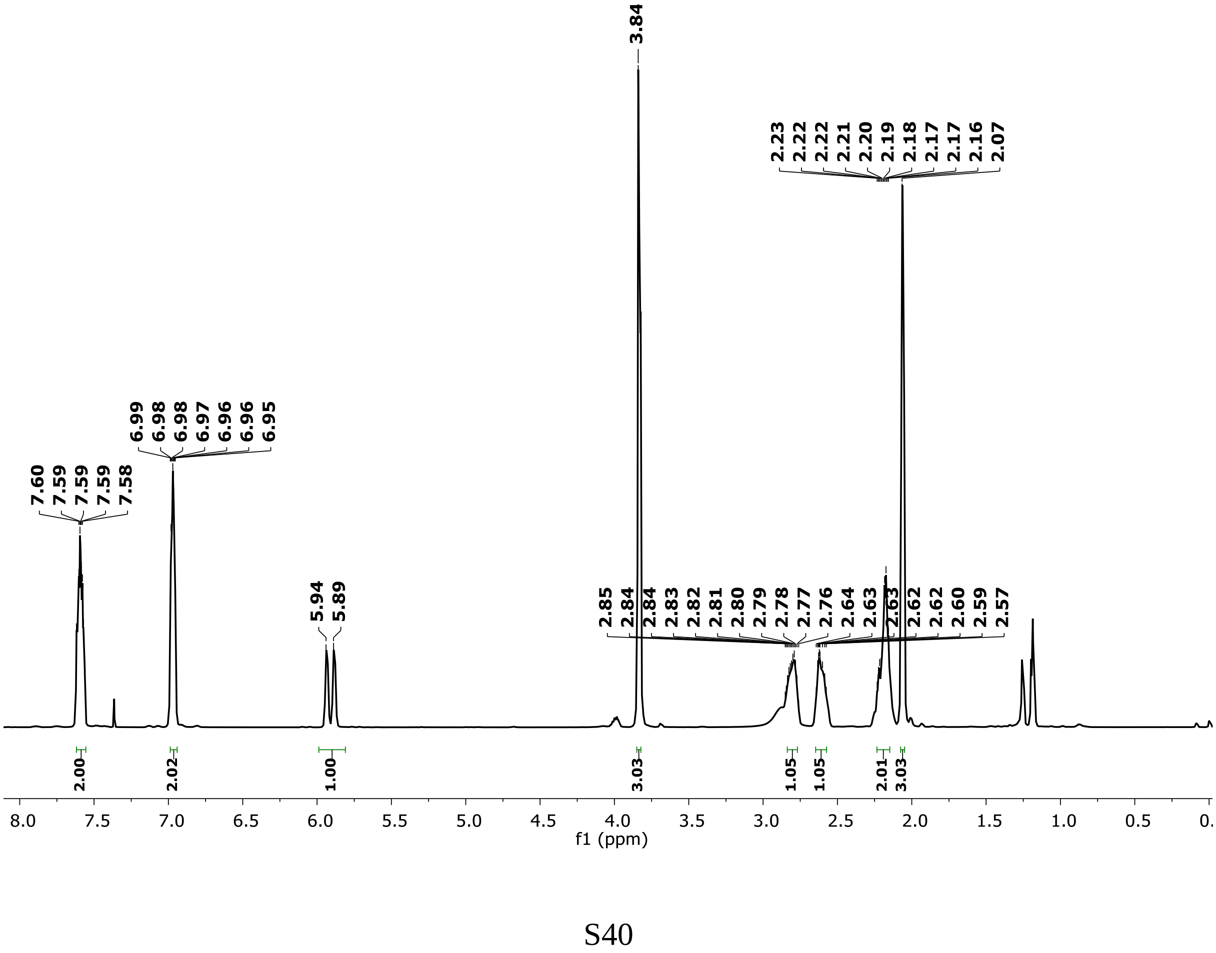

^{13}C NMR

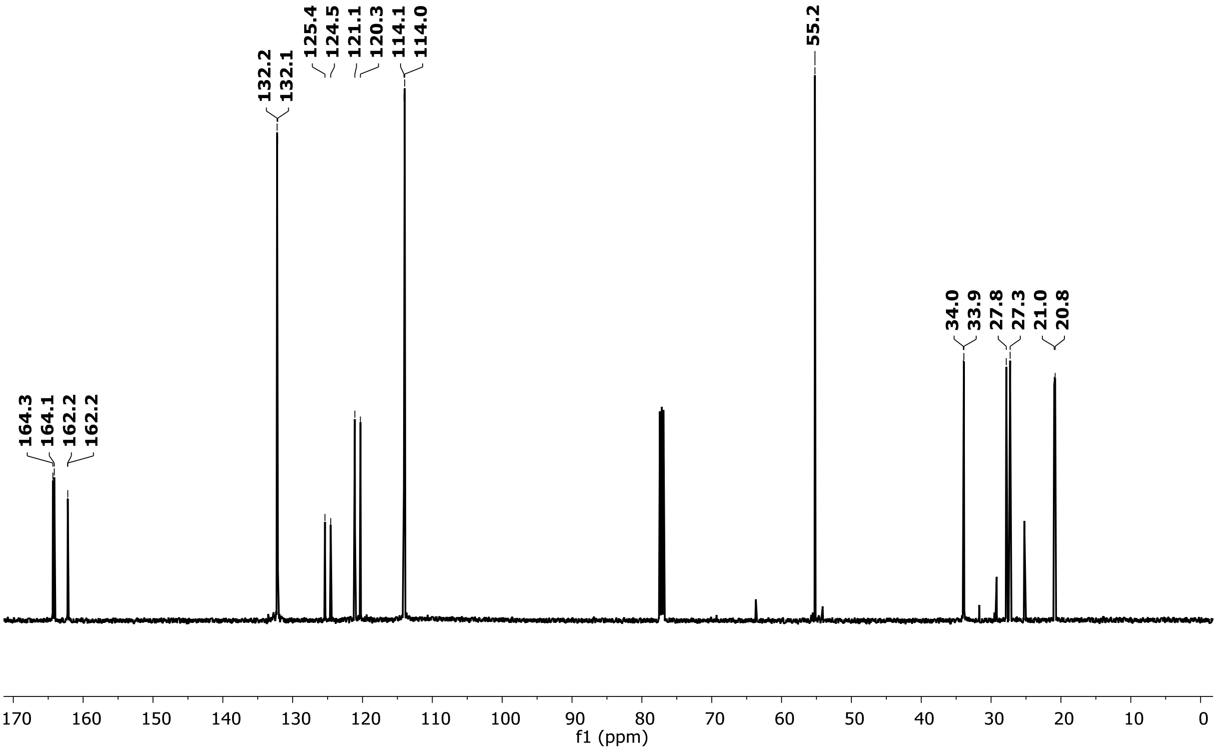
164.3
164.1
162.2
162.2
132.2
132.1
125.4
124.5
121.1
120.3
114.1
114.0
55.2
34.0
33.9
27.8
27.3
21.0
20.8
170 160 150 140 130 120 110 100 90 80 70 60 50 40 30 20 10 0
f1 (ppm)

1-(2,6-Dimethylphenyl)-3-methyl-2-phospholene oxide (**4f**):

^{31}P NMR

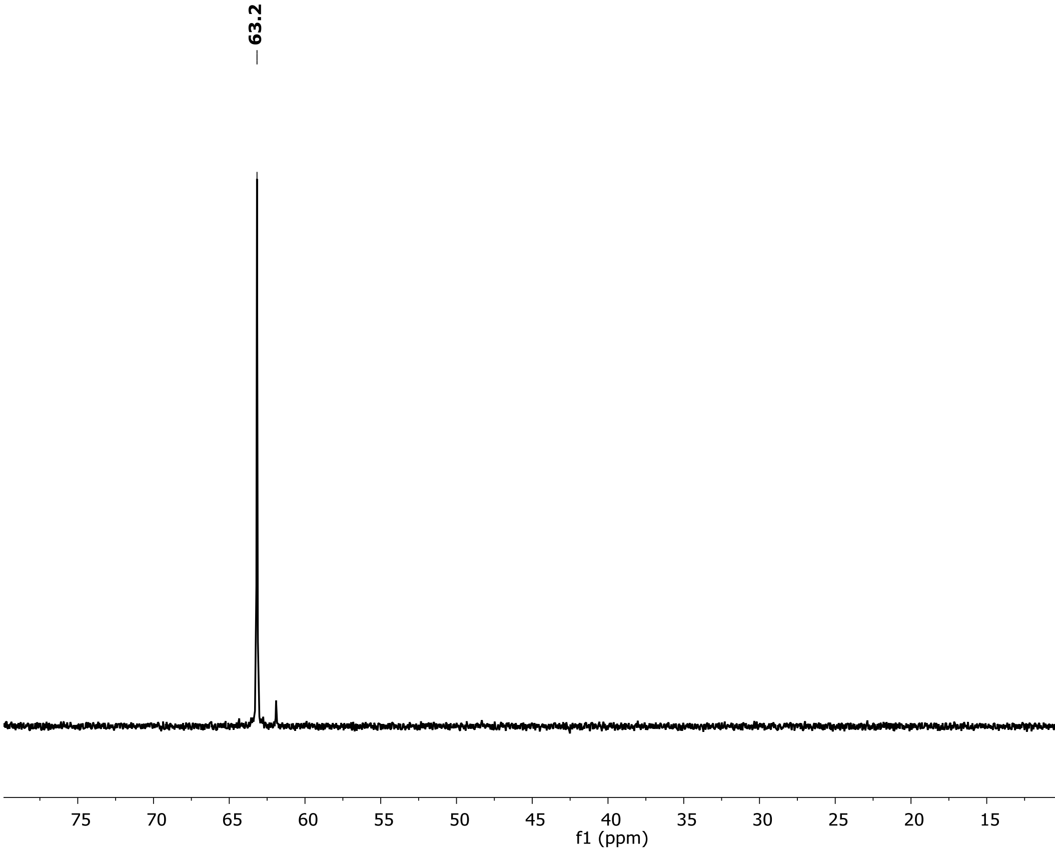

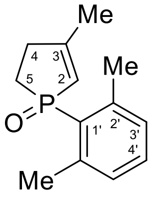

4f

^{1}H NMR

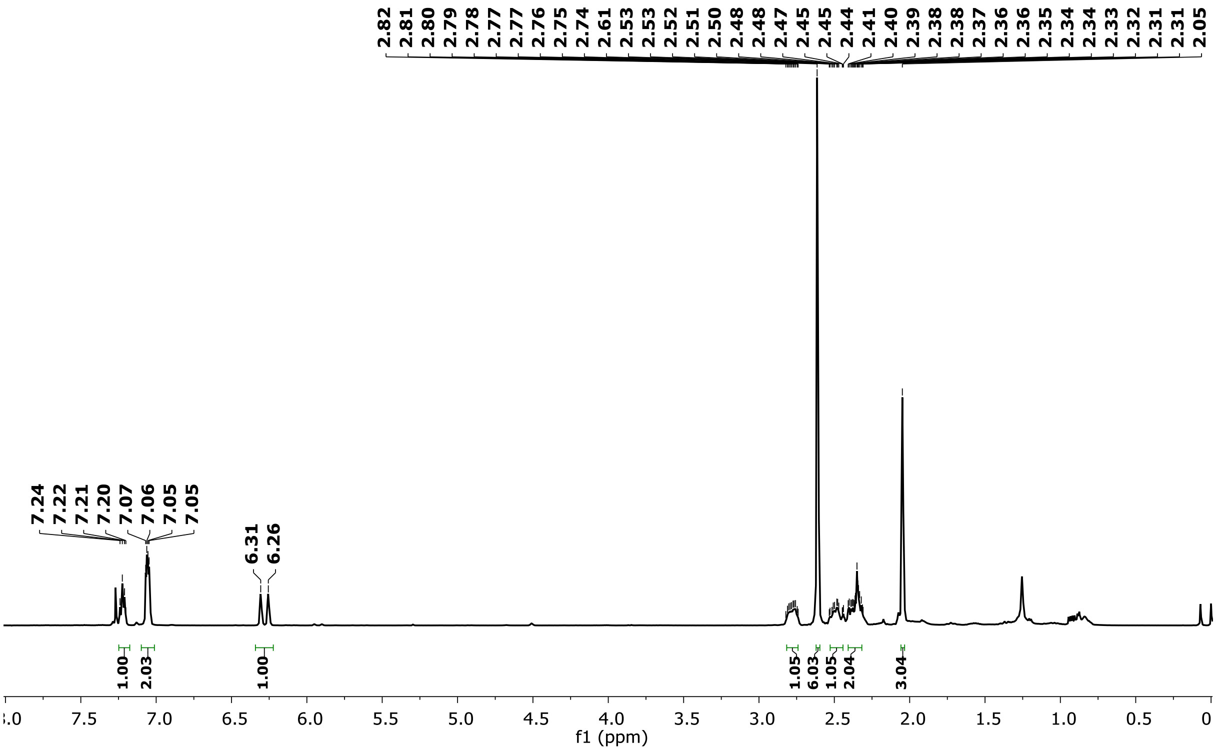

^{13}C NMR

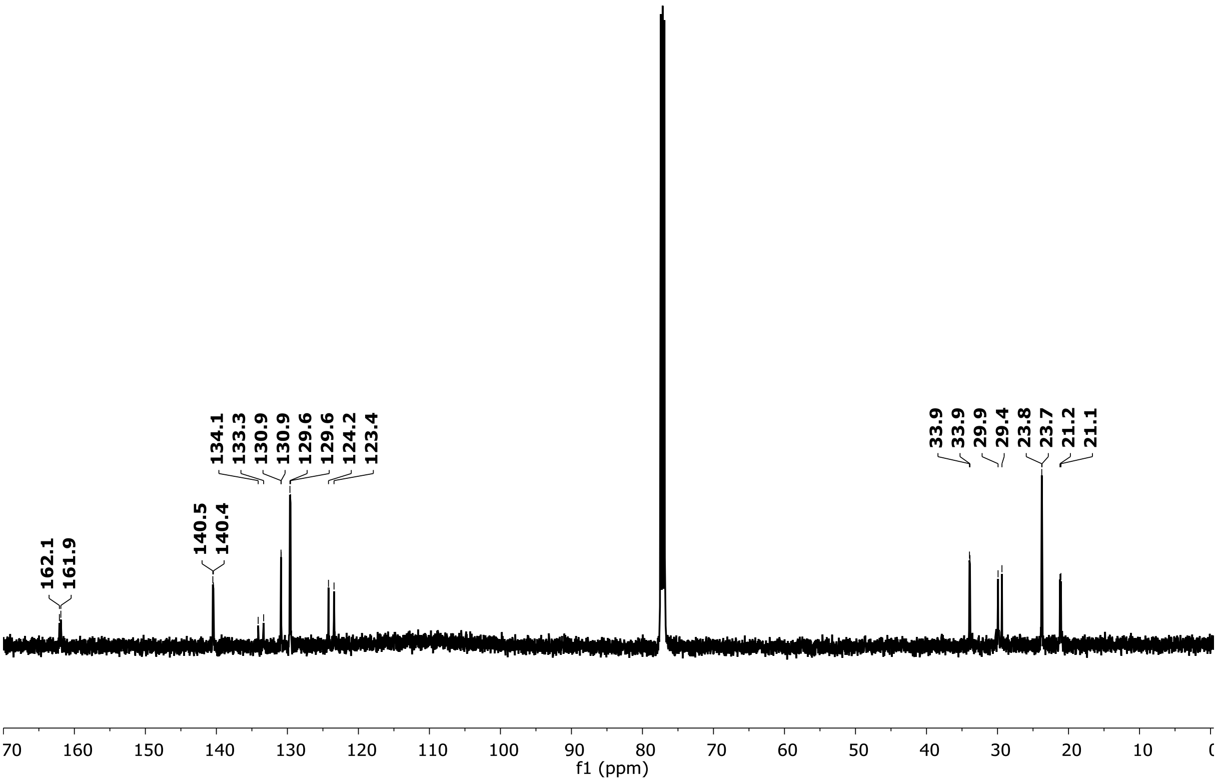

162.1
161.9
140.5
140.4
134.1
133.3
130.9
130.9
129.6
129.6
124.2
123.4
33.9
33.9
29.9
29.4
23.8
23.7
21.2
21.1
70
160
150
140
130
120
110
100
90
80
70
60
50
40
30
20
10
f1 (ppm)

1-(1-Naphtyl)-3-methyl-2-phospholene oxide (**4g**):

^{31}P NMR

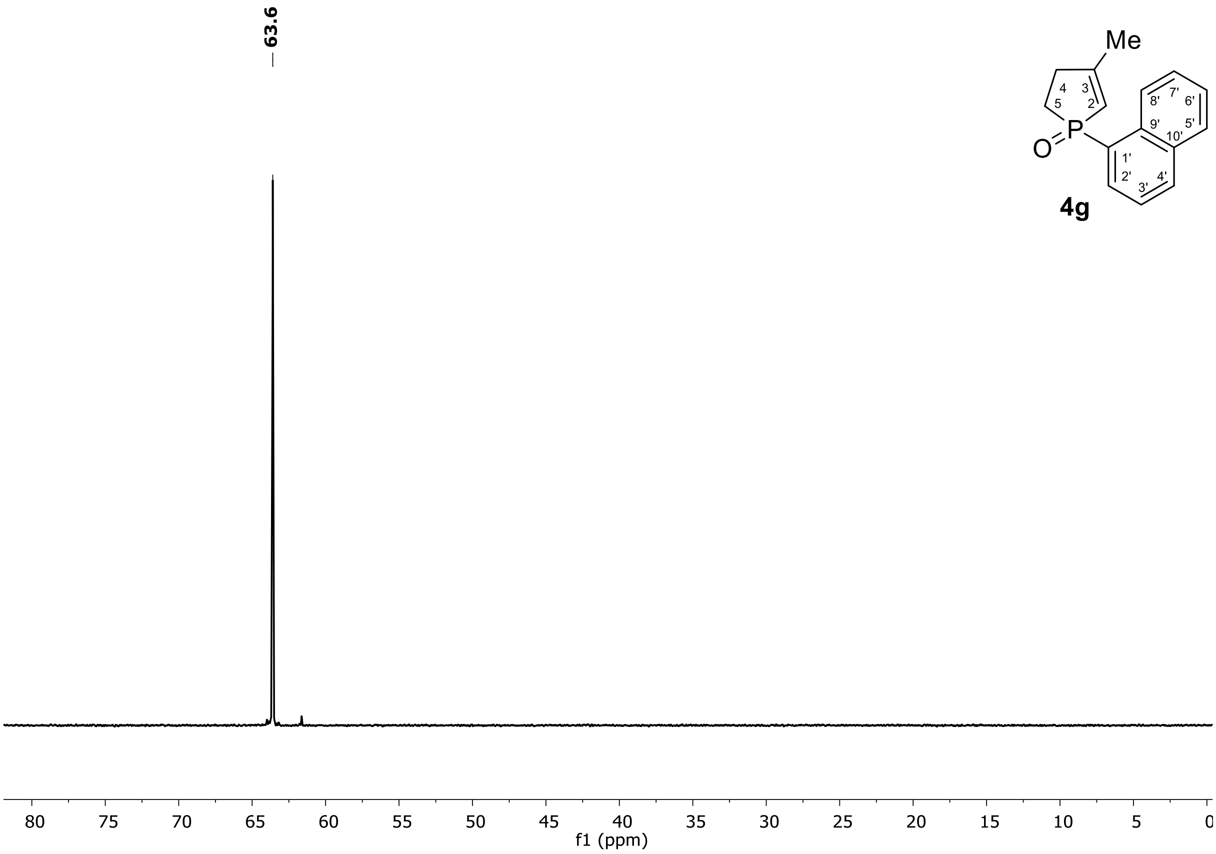

^{1}H NMR

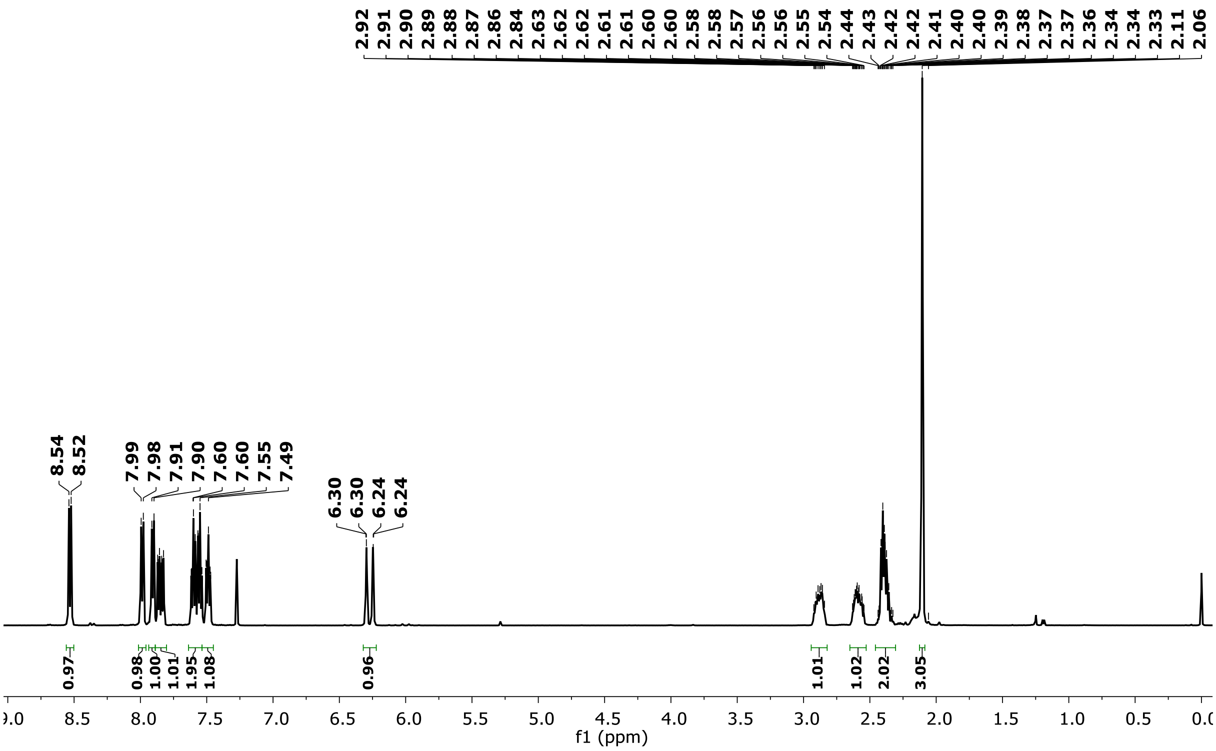

^{13}C NMR

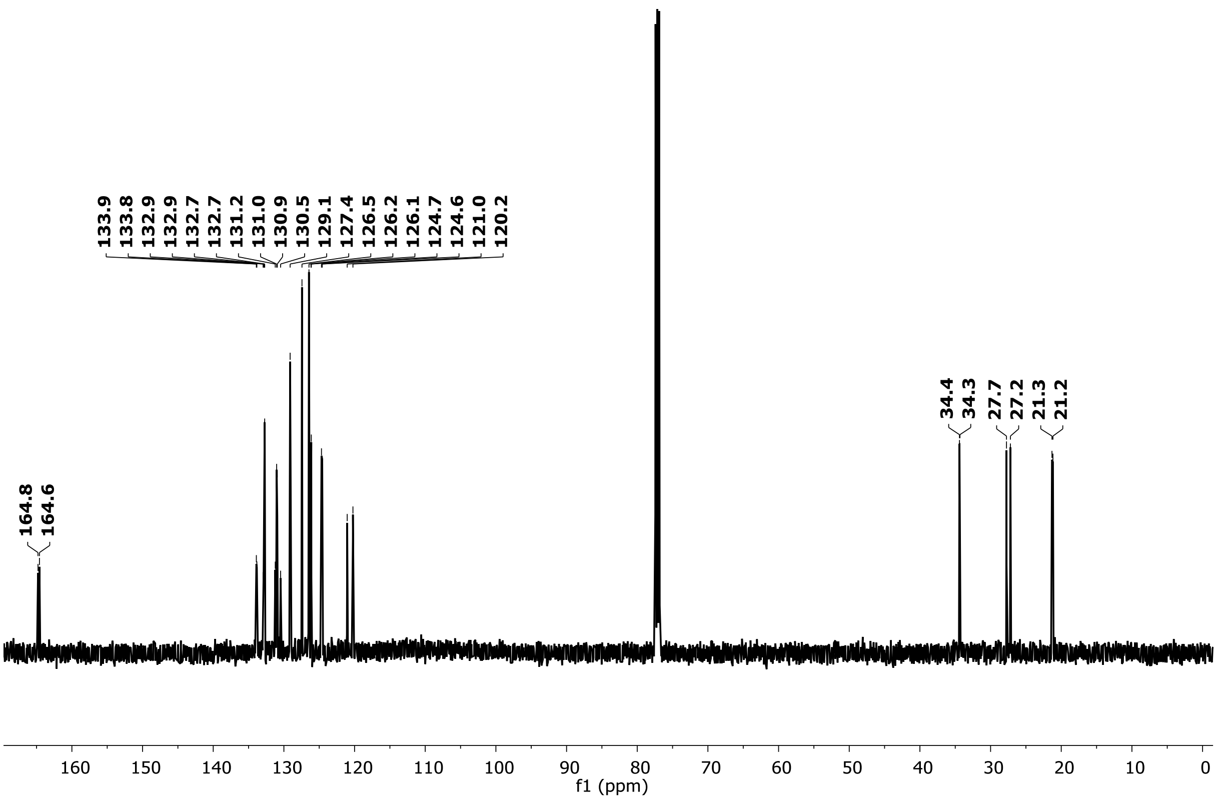

164.8
164.6
133.9
133.8
132.9
132.9
132.7
132.7
131.2
131.0
130.9
130.5
129.1
127.4
126.5
126.2
126.1
124.7
124.6
121.0
120.2
34.4
34.3
27.7
27.2
21.3
21.2
160 150 140 130 120 110 100 90 80 70 60 50 40 30 20 10 0
f1 (ppm)

1-Ethyl-3-methyl-2-phospholene oxide (**4h**):

^{31}P NMR

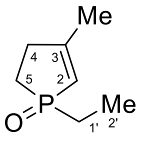

4h

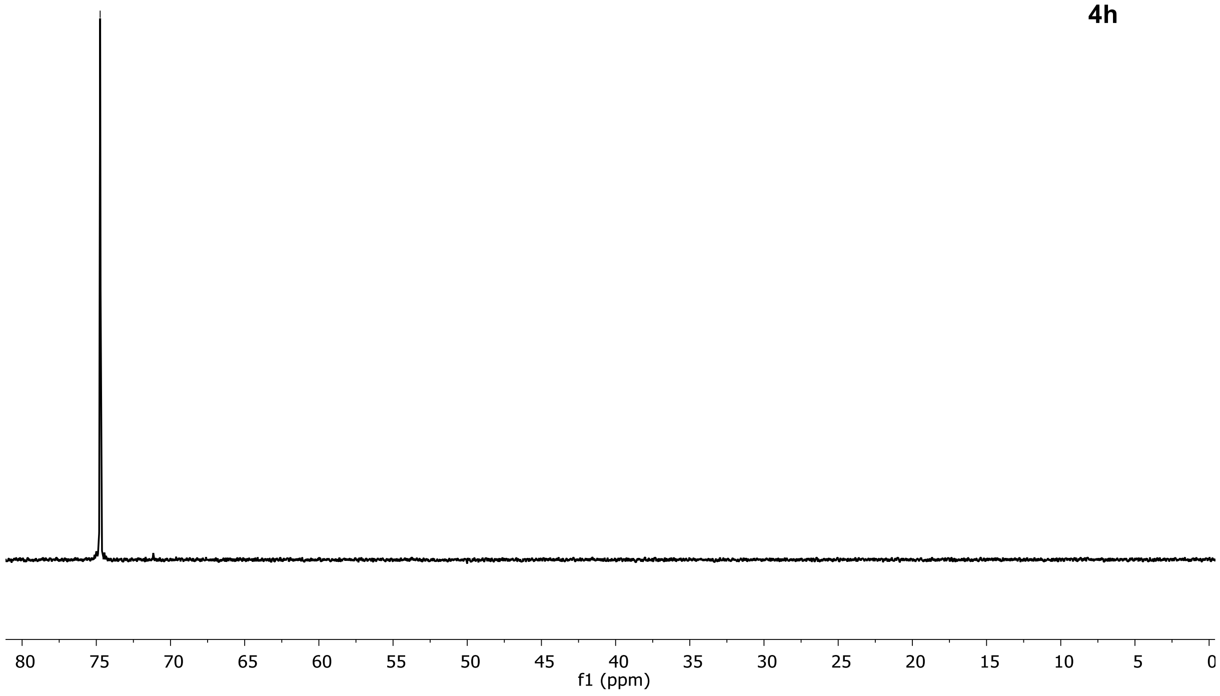

^{1}H NMR

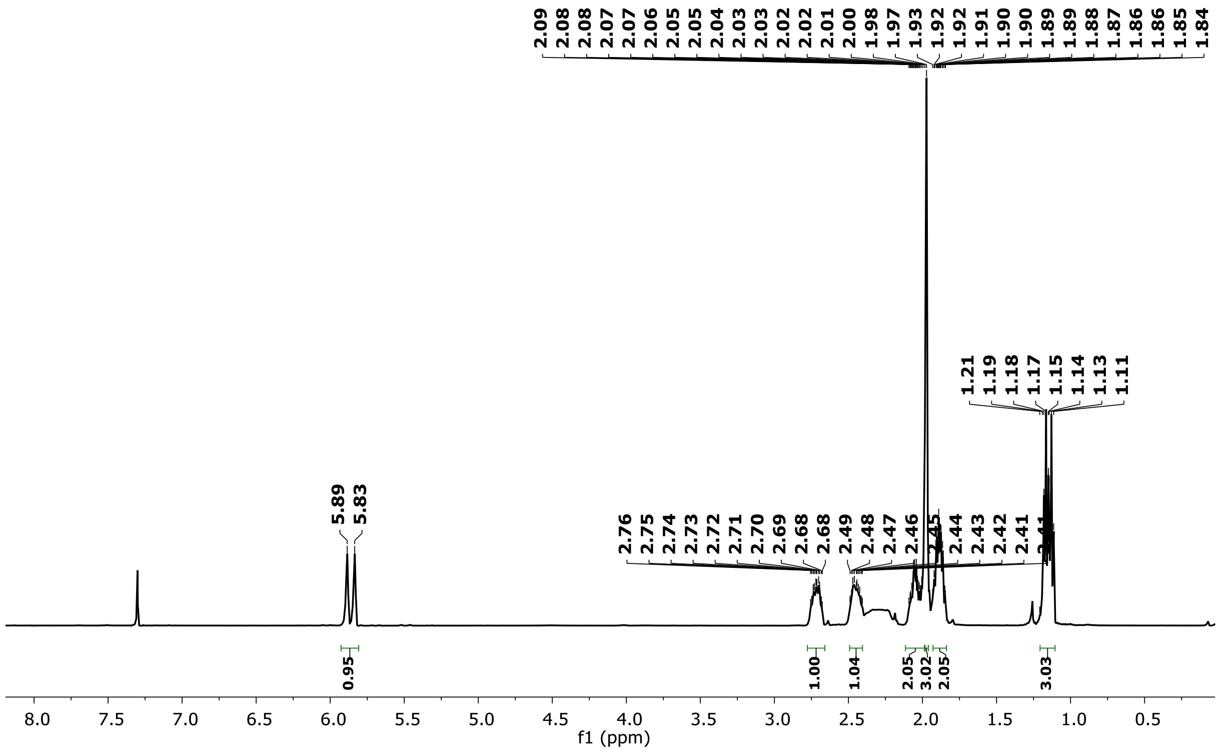

^{13}C NMR

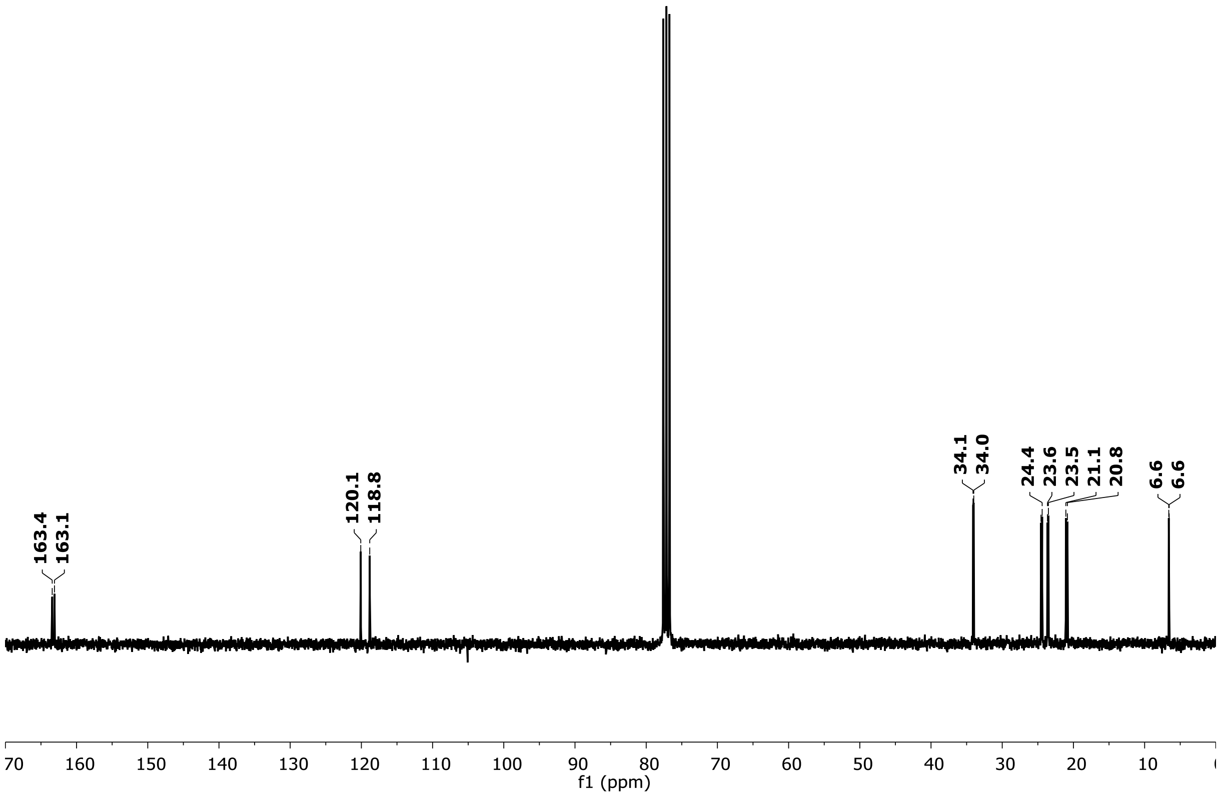
163.4
163.1
120.1
118.8
34.1
34.0
24.4
23.6
23.5
21.1
20.8
6.6
6.6
70
160
150
140
130
120
110
100
90
80
70
60
50
40
30
20
10
f1 (ppm)

1-Propyl-3-methyl-2-phospholene oxide (**4i**):

^{31}P NMR

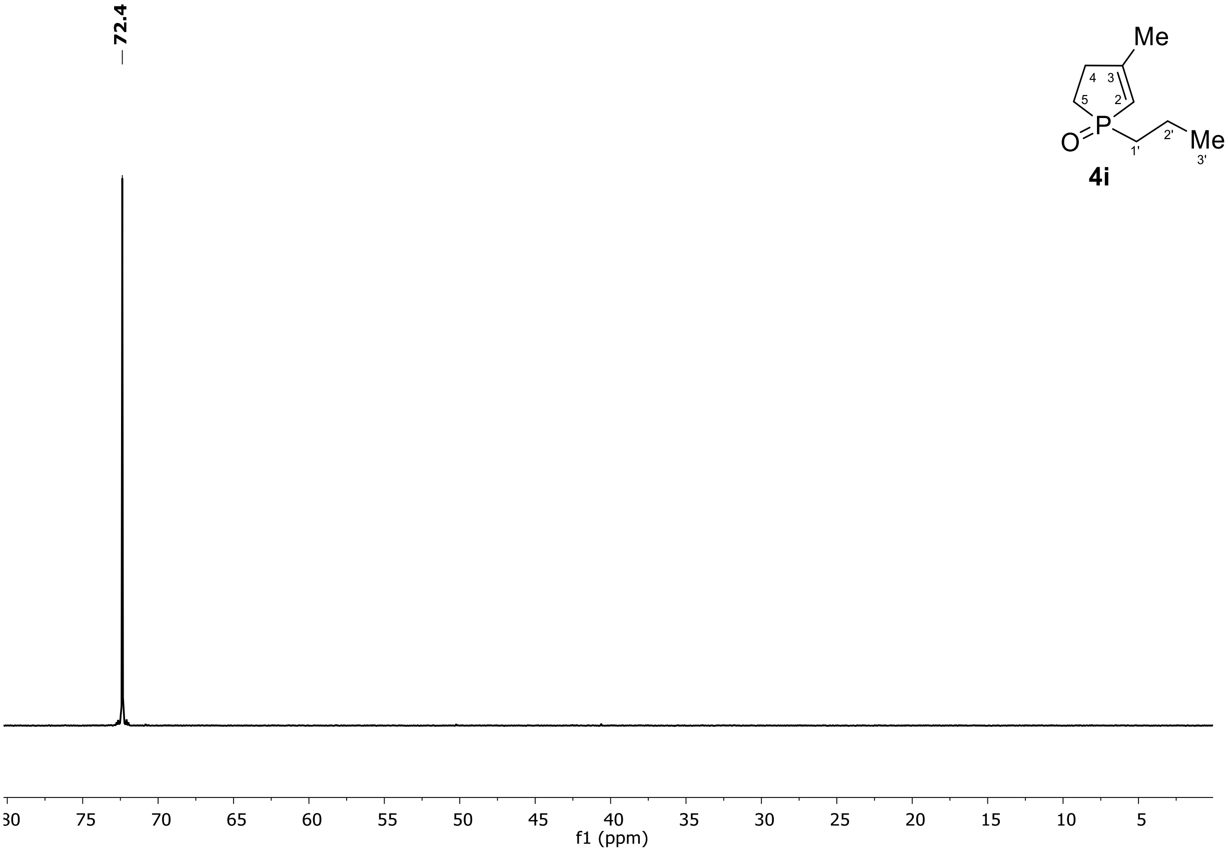

4i

^{1}H NMR

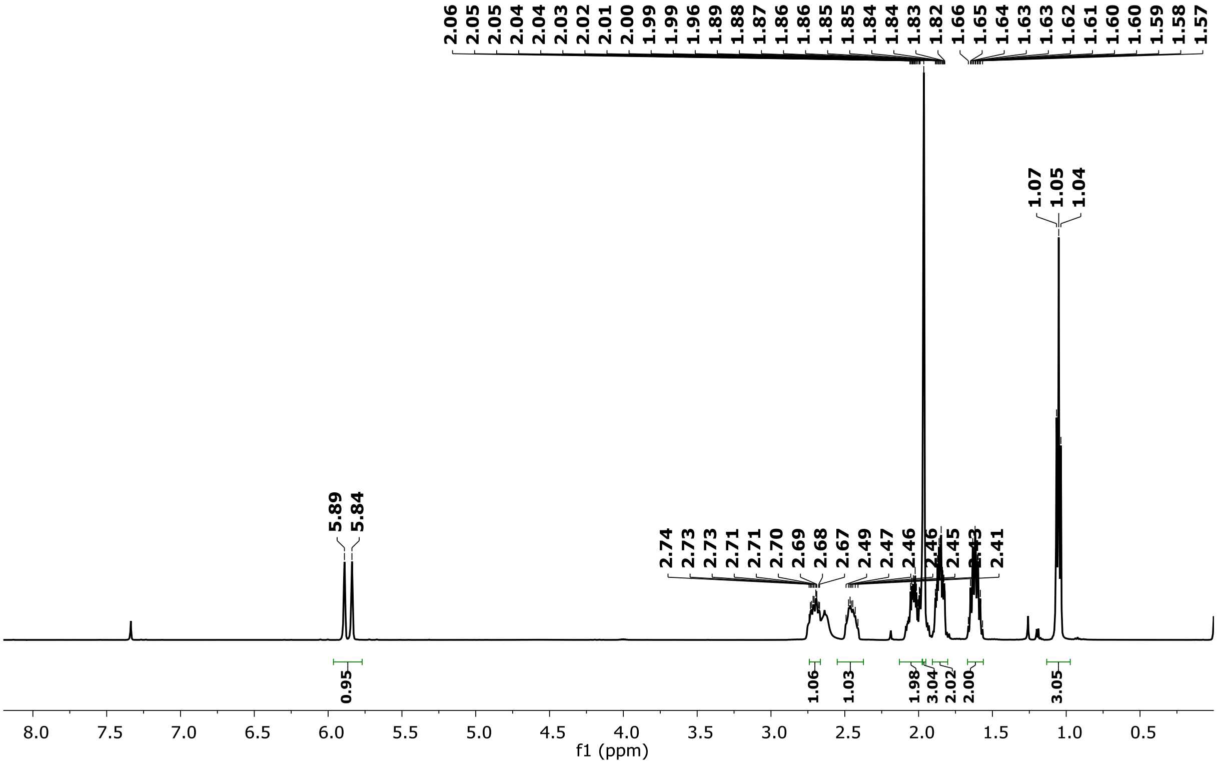

^{13}C NMR

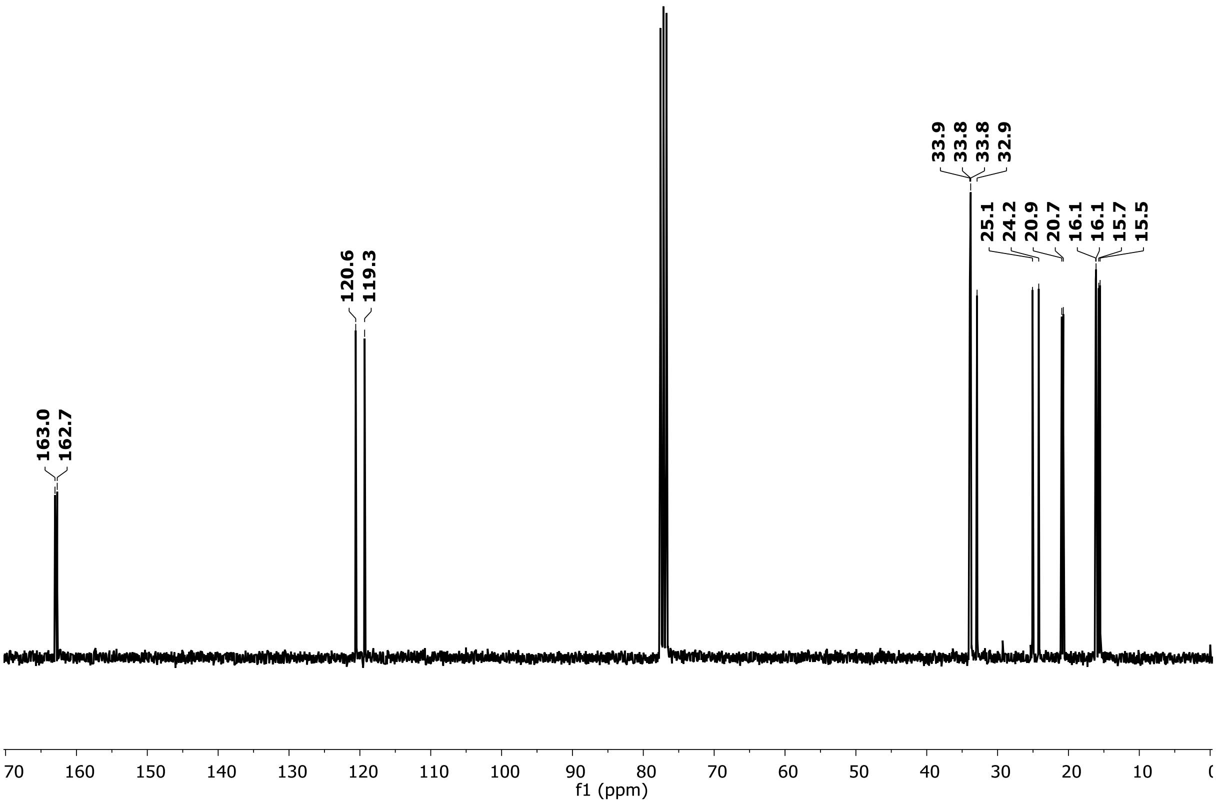

163.0
162.7
120.6
119.3
33.9
33.8
33.8
32.9
25.1
24.2
20.9
20.7
16.1
16.1
15.7
15.5
160
150
140
130
120
110
100
90
80
70
60
50
40
30
20
10
f1 (ppm)

1-Butyl-3-methyl-2-phospholene oxide (**4j**):

^{31}P NMR

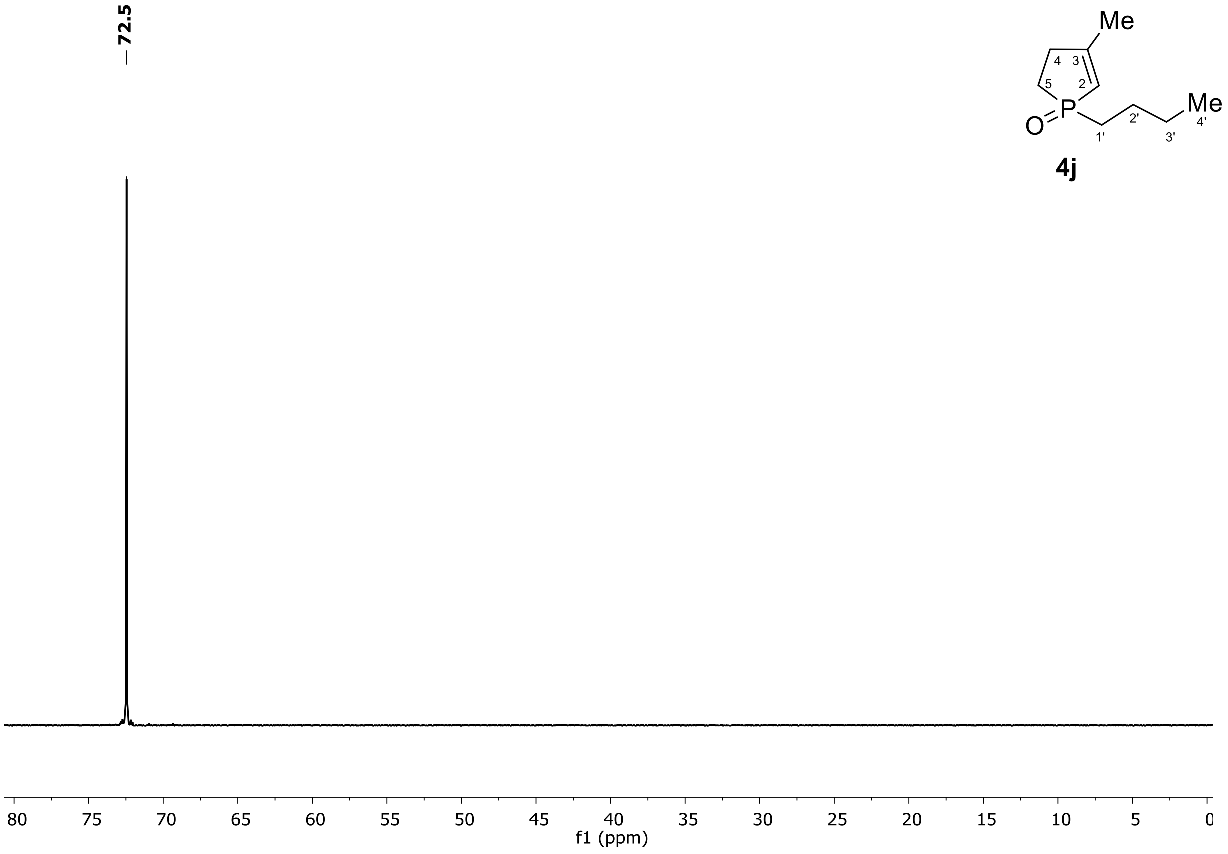

^{1}H NMR

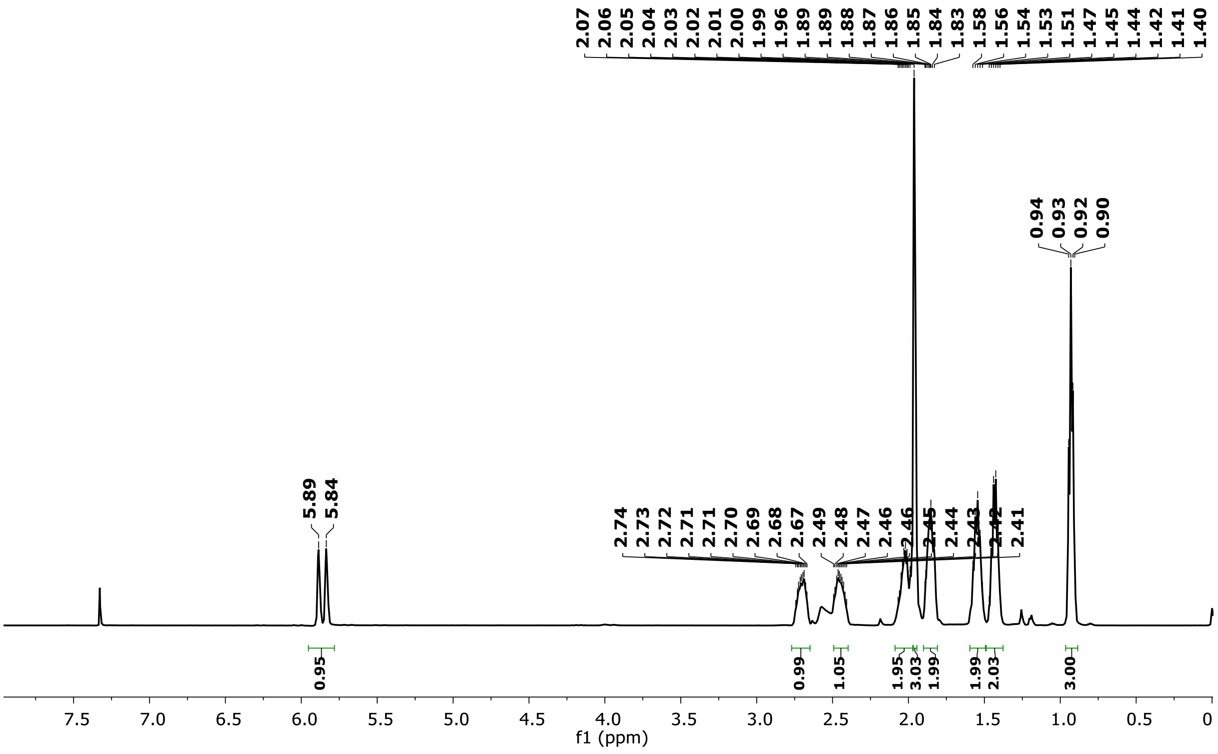

^{13}C NMR

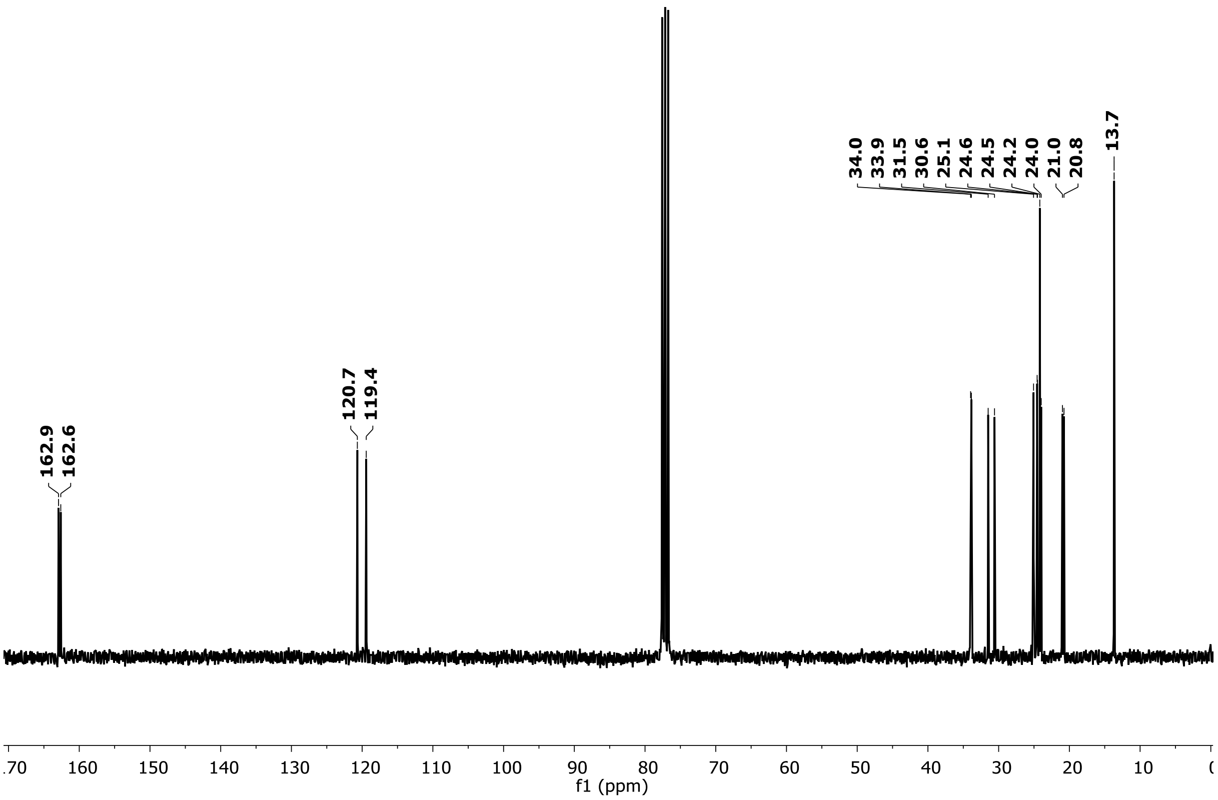
162.9
162.6
120.7
119.4
34.0
33.9
31.5
30.6
25.1
24.6
24.5
24.2
24.0
21.0
20.8
13.7
170 160 150 140 130 120 110 100 90 80 70 60 50 40 30 20 10 0
f1 (ppm)

1-*i*-Butyl-3-methyl-2-phospholene oxide (**4k**):

^{31}P NMR

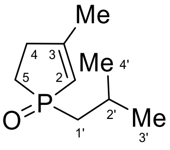

4k

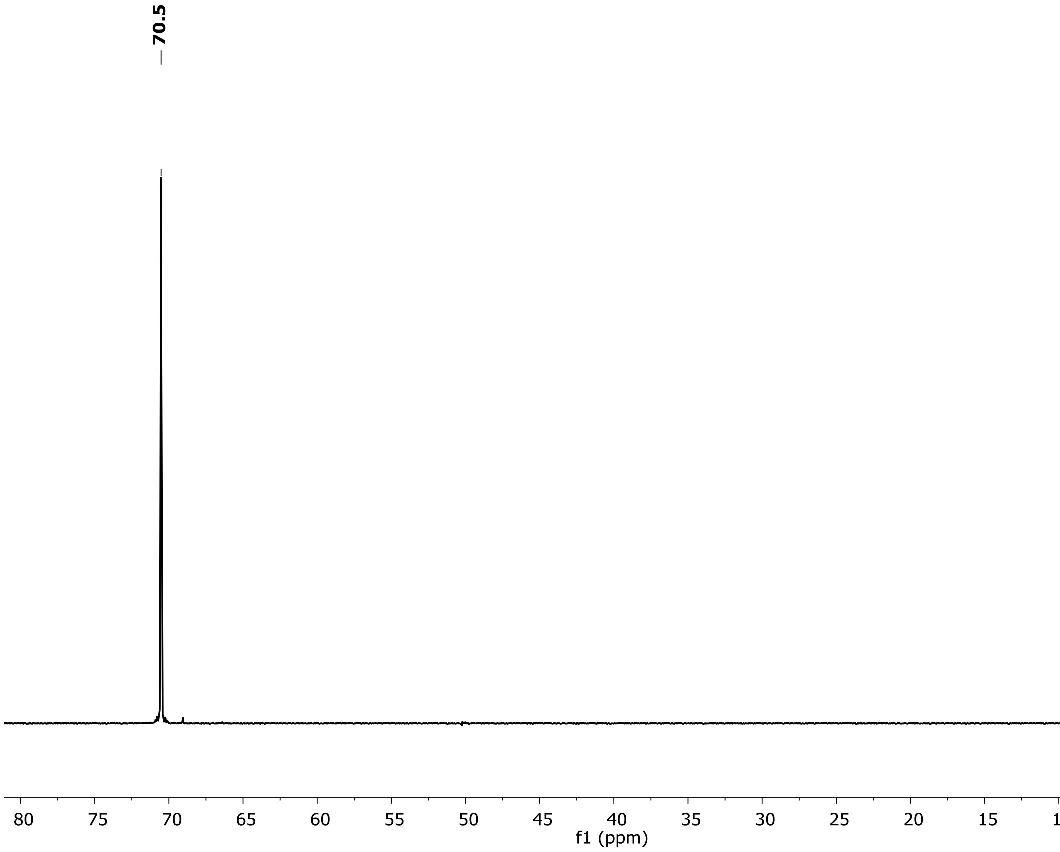

^{1}H NMR

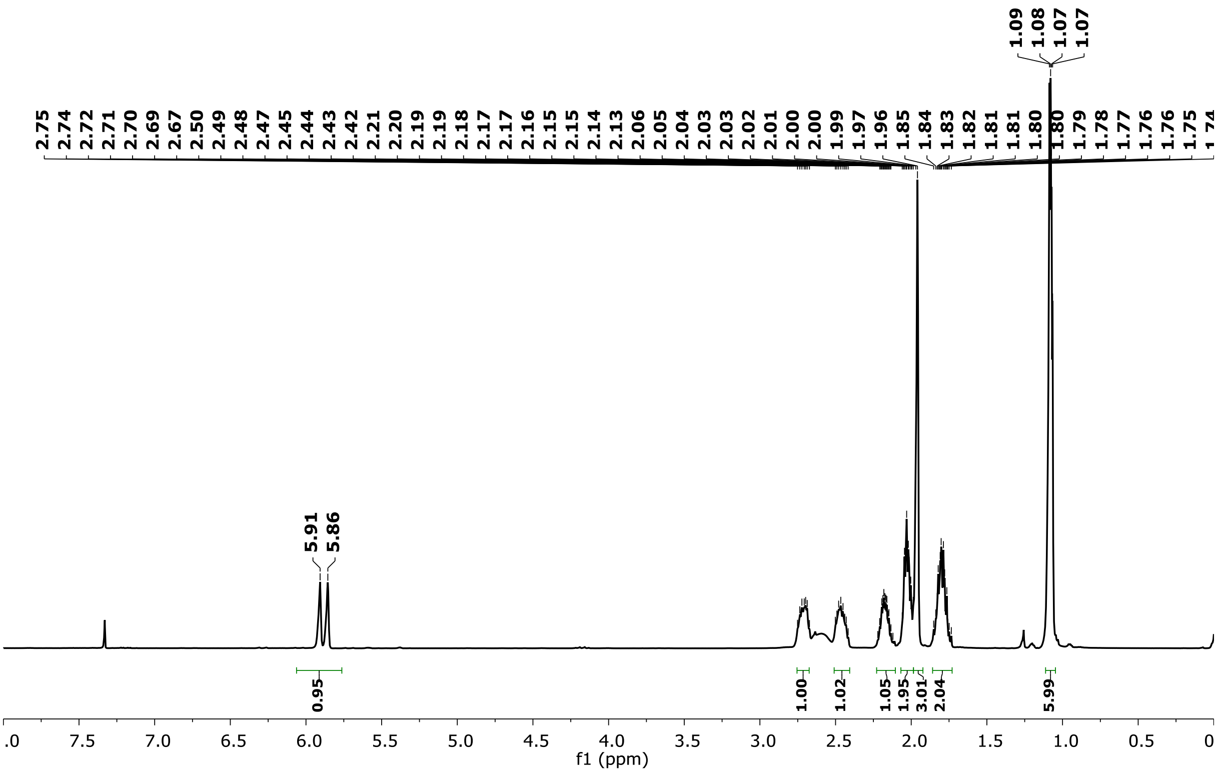

^{13}C NMR

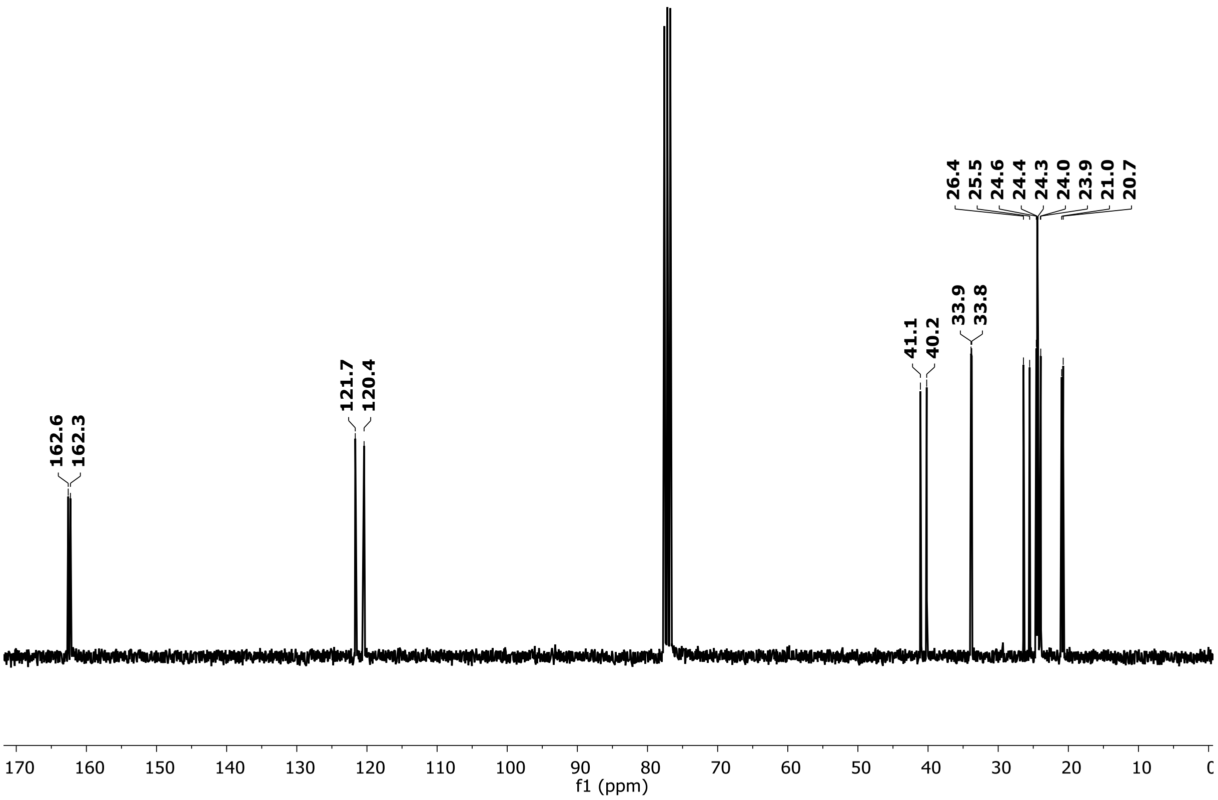

1-*i*-Pentyl-3-methyl-2-phospholene oxide (**4l**):

^{31}P NMR

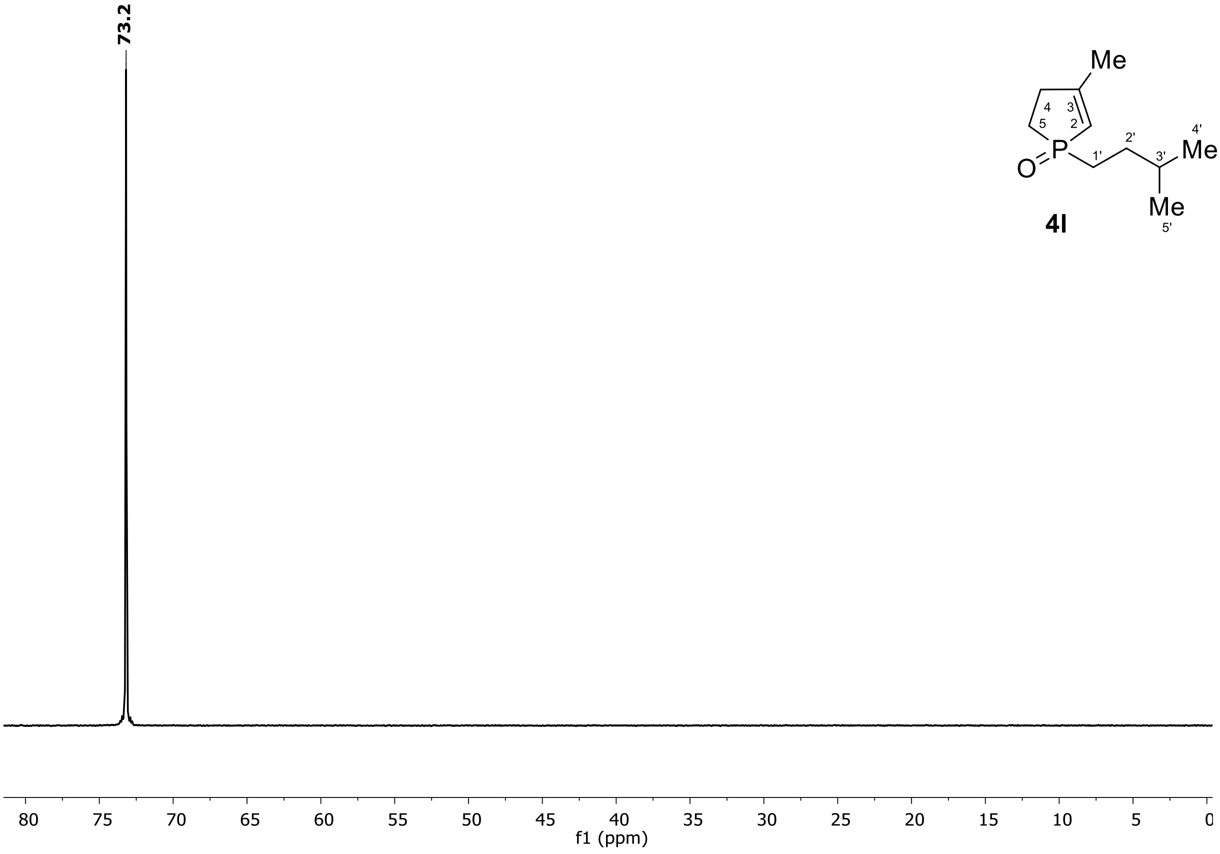

^{1}H NMR

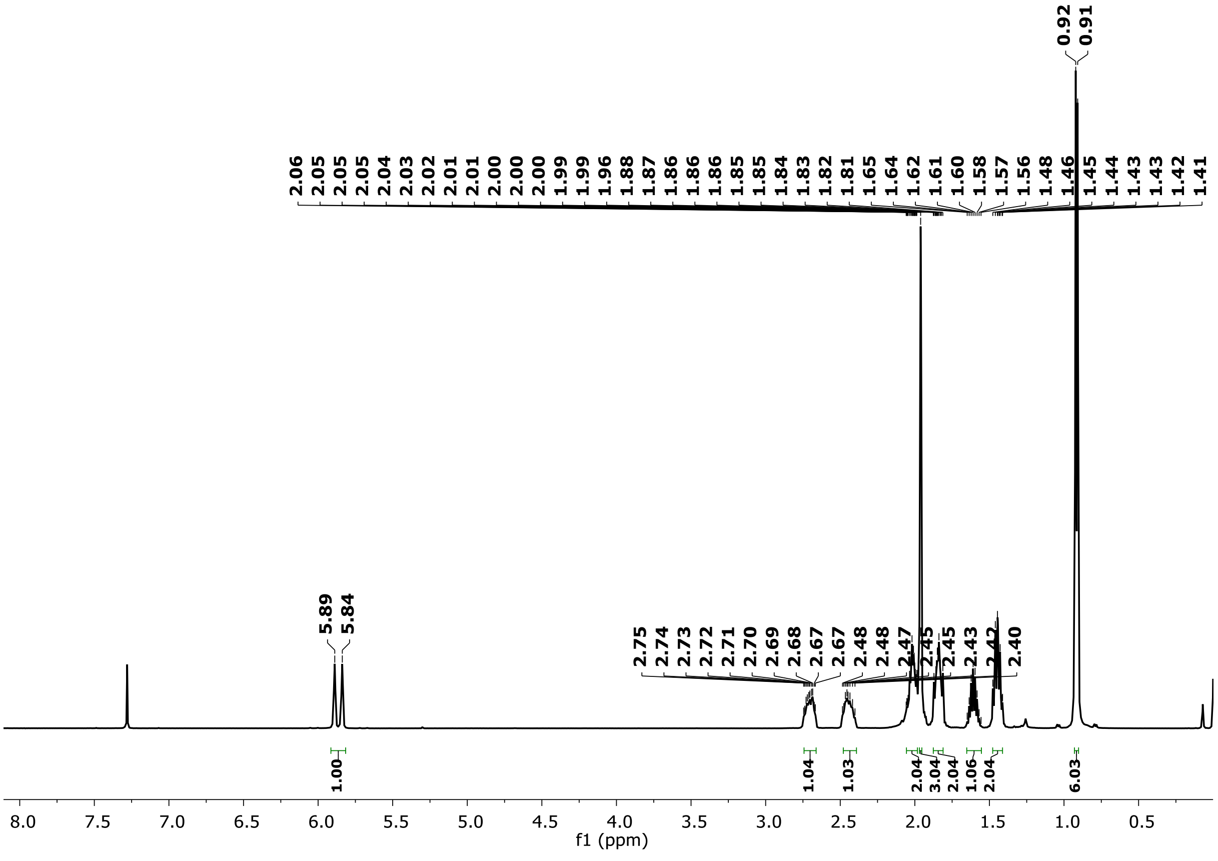

^{13}C NMR

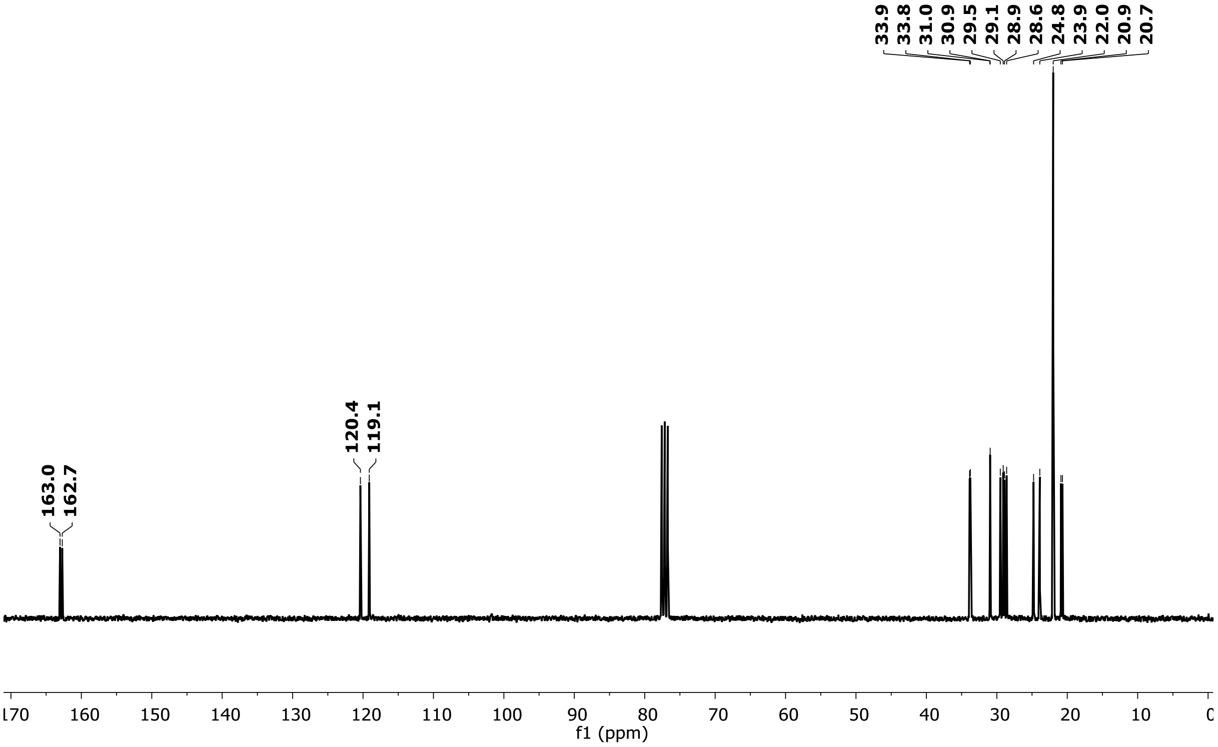

33.9
33.8
31.0
30.9
29.5
29.1
28.9
28.6
24.8
23.9
22.0
20.9
20.7
163.0
162.7
120.4
119.1
170
160
150
140
130
120
110
100
90
80
70
60
50
40
30
20
10
0
f1 (ppm)

1-Phenyl-2-phospholene oxide (**7**):

^{31}P NMR

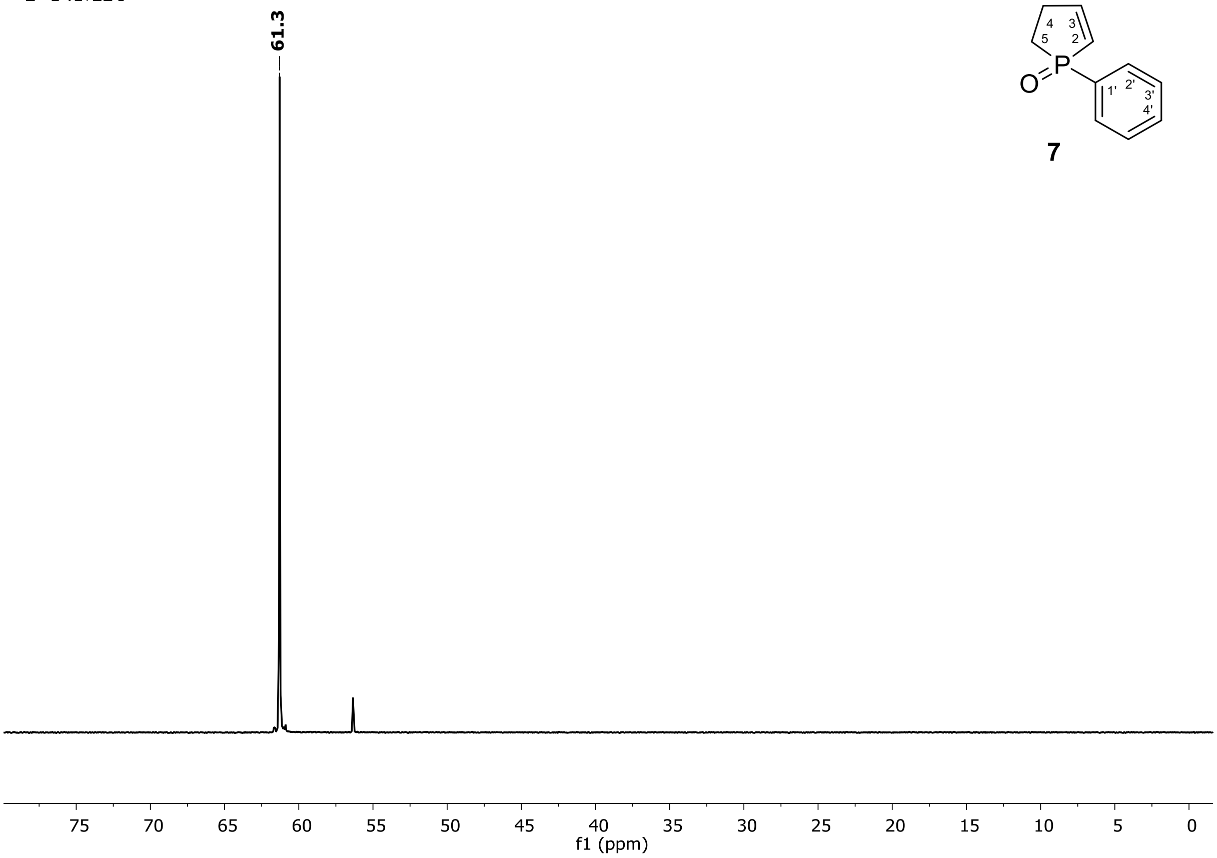

7

^{1}H NMR

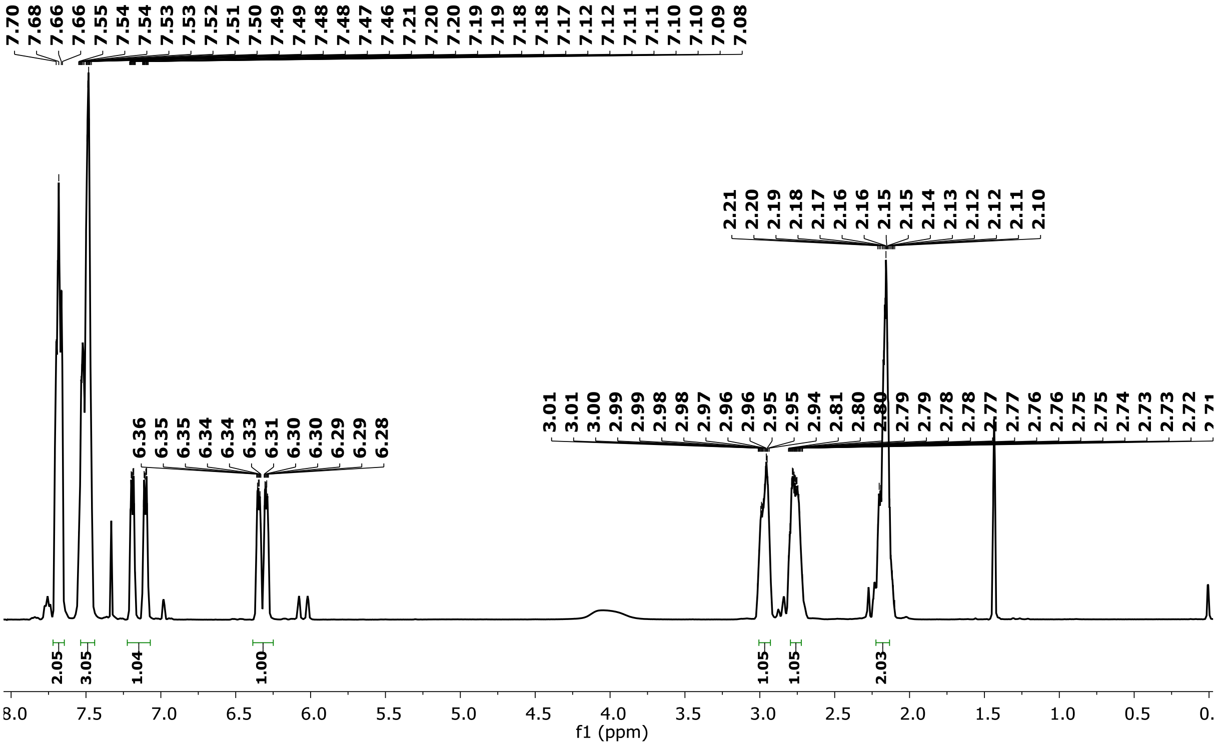

^{13}C NMR

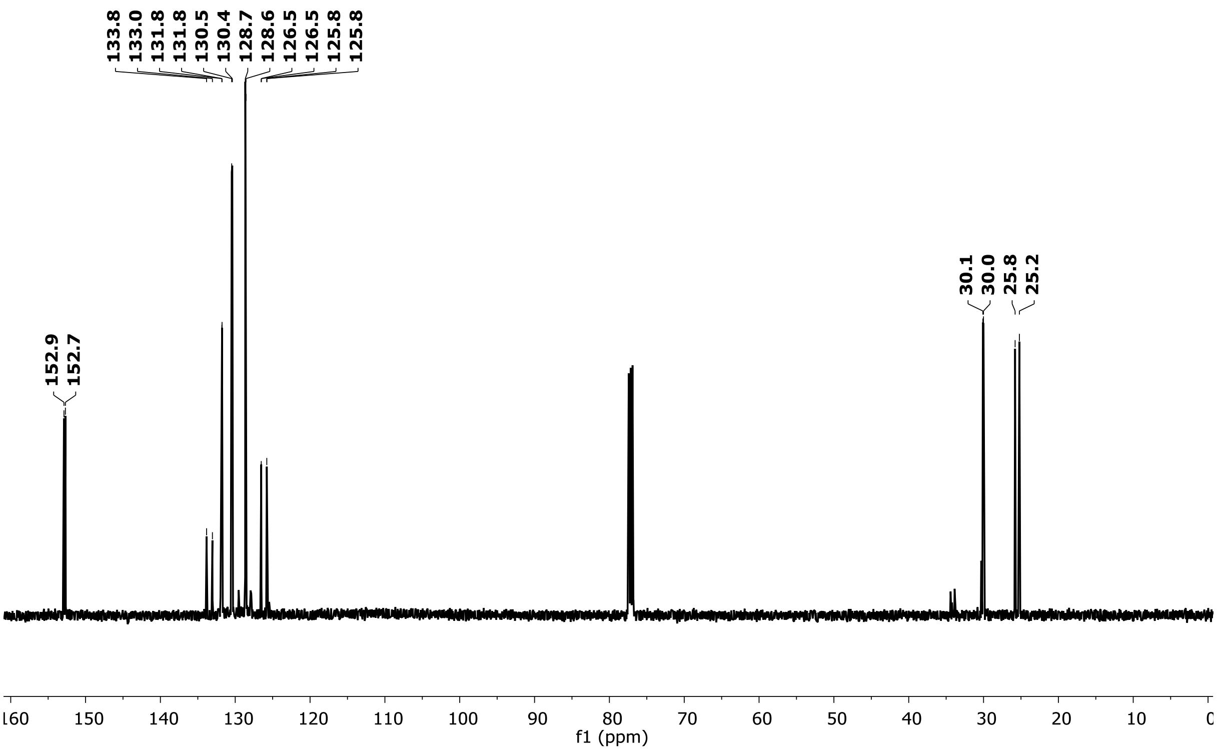

133.8
133.0
131.8
131.8
130.5
130.4
128.7
128.6
126.5
126.5
125.8
125.8
152.9
152.7
30.1
30.0
25.8
25.2
160
150
140
130
120
110
100
90
80
70
60
50
40
30
20
10
0
f1 (ppm)

cis-1-Phenyl-3,4-dimethyl-2-phospholene oxide (*cis*-**10**):
^{31}P NMR

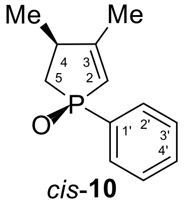

cis-**10**

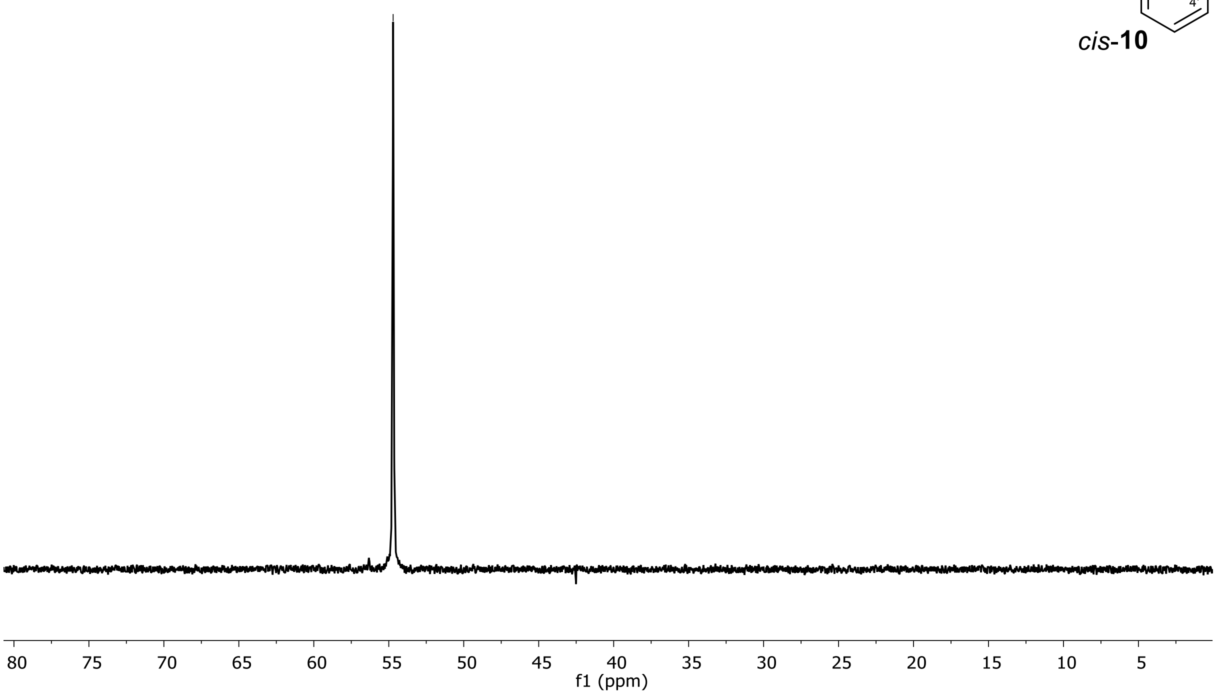

^{1}H NMR

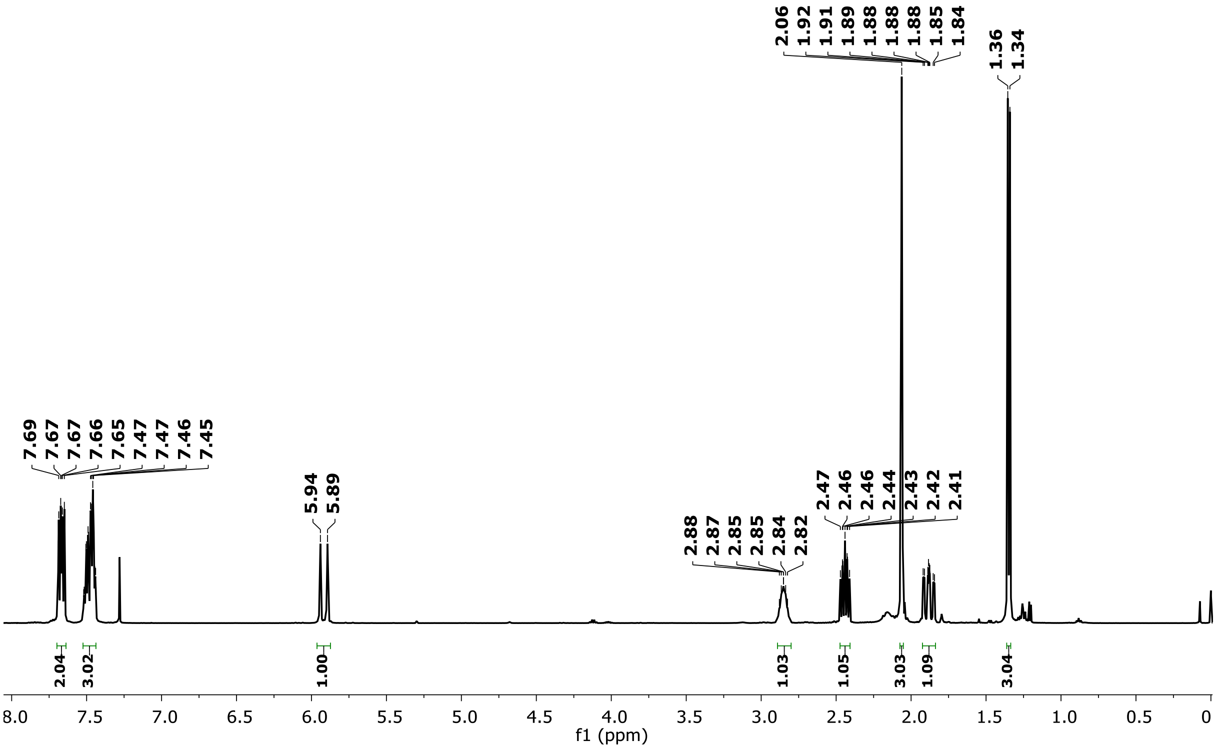

^{13}C NMR

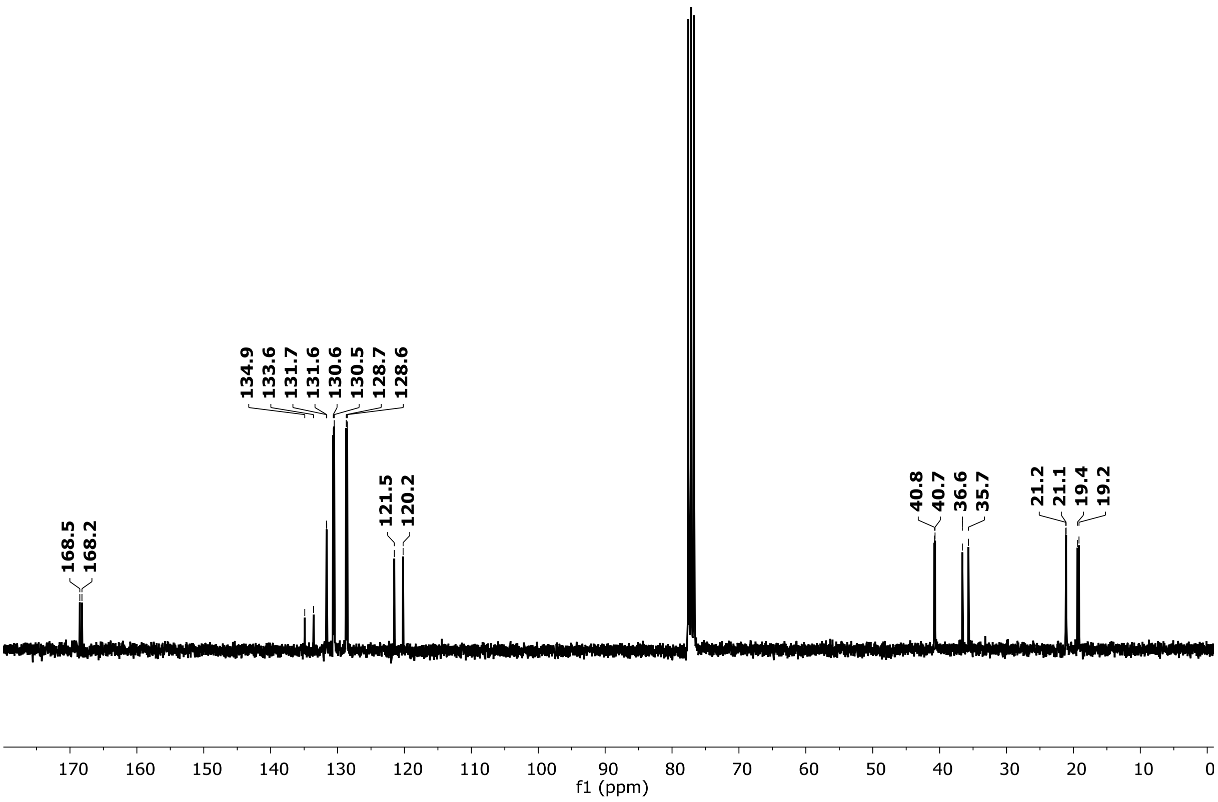
168.5
168.2
134.9
133.6
131.7
131.6
130.6
130.5
128.7
128.6
121.5
120.2
40.8
40.7
36.6
35.7
21.2
21.1
19.4
19.2
170 160 150 140 130 120 110 100 90 80 70 60 50 40 30 20 10 0
f1 (ppm)

trans-1-Phenyl-3,4-dimethyl-2-phospholene oxide (*trans*-**10**):

^{31}P NMR

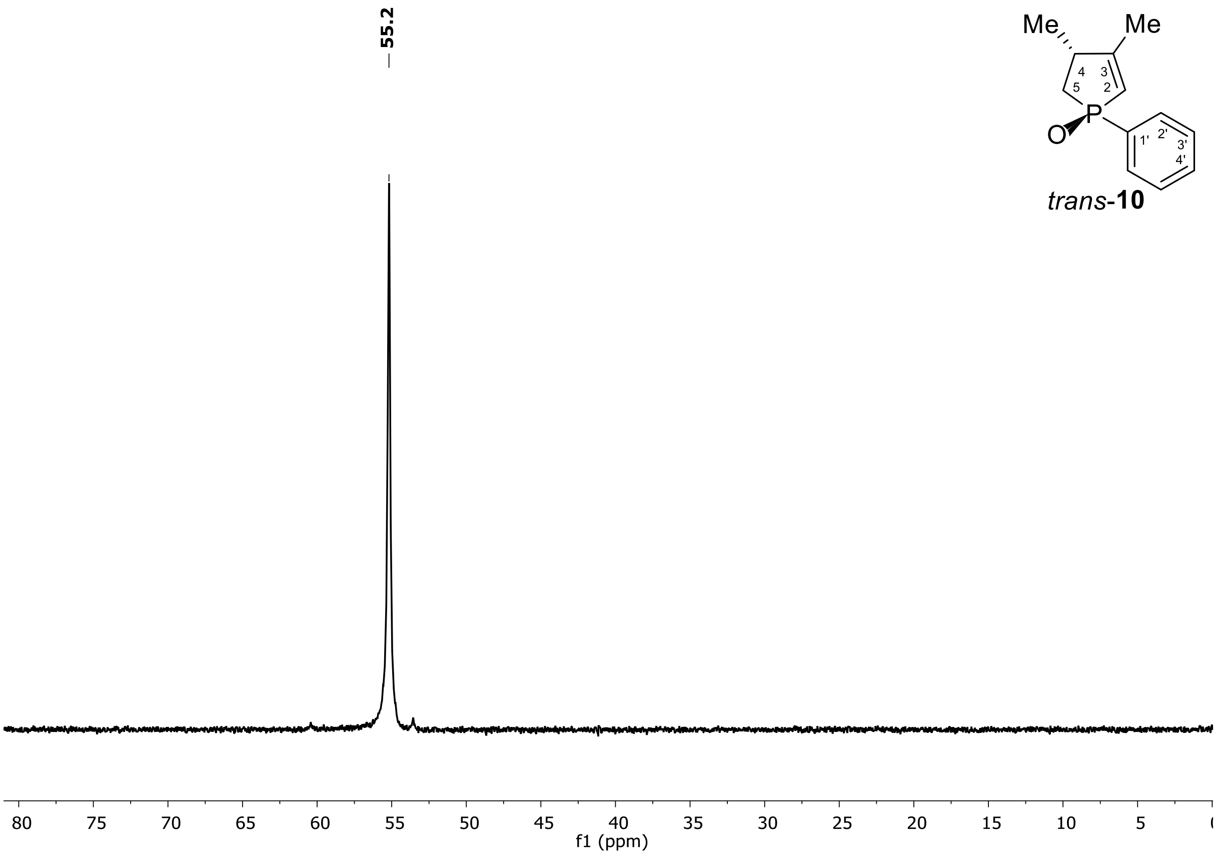

^{1}H NMR

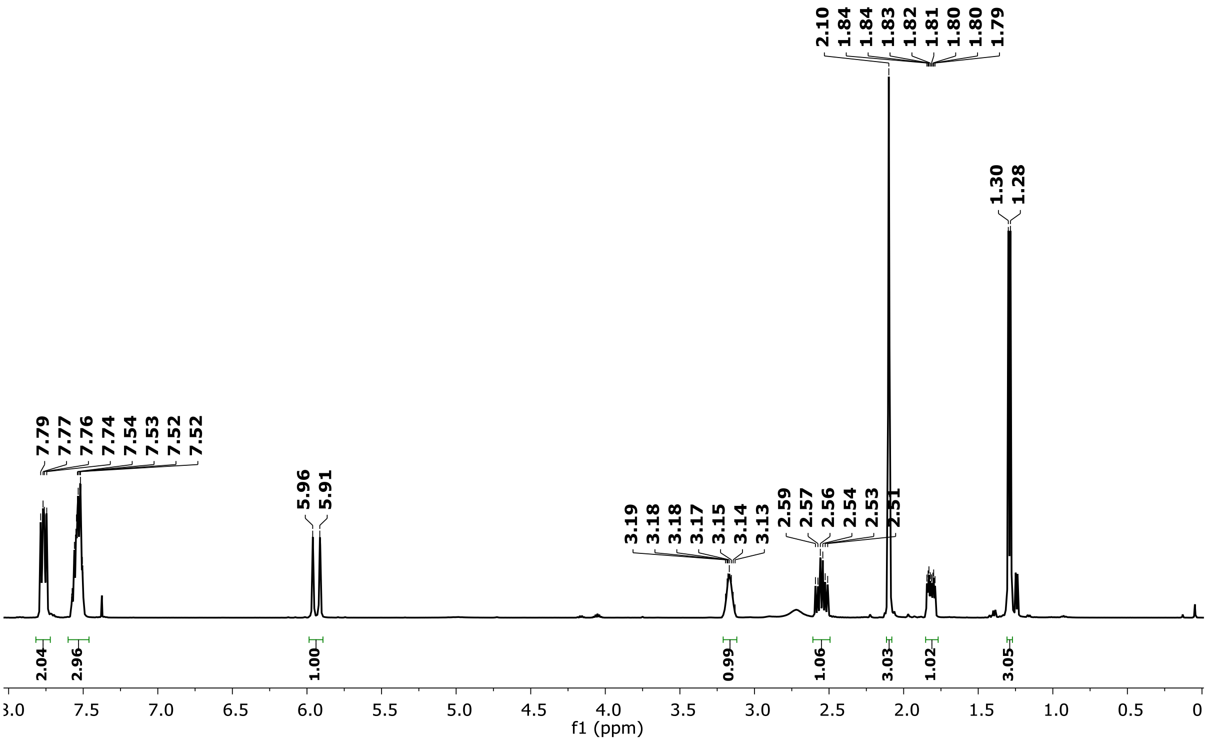

^{13}C NMR

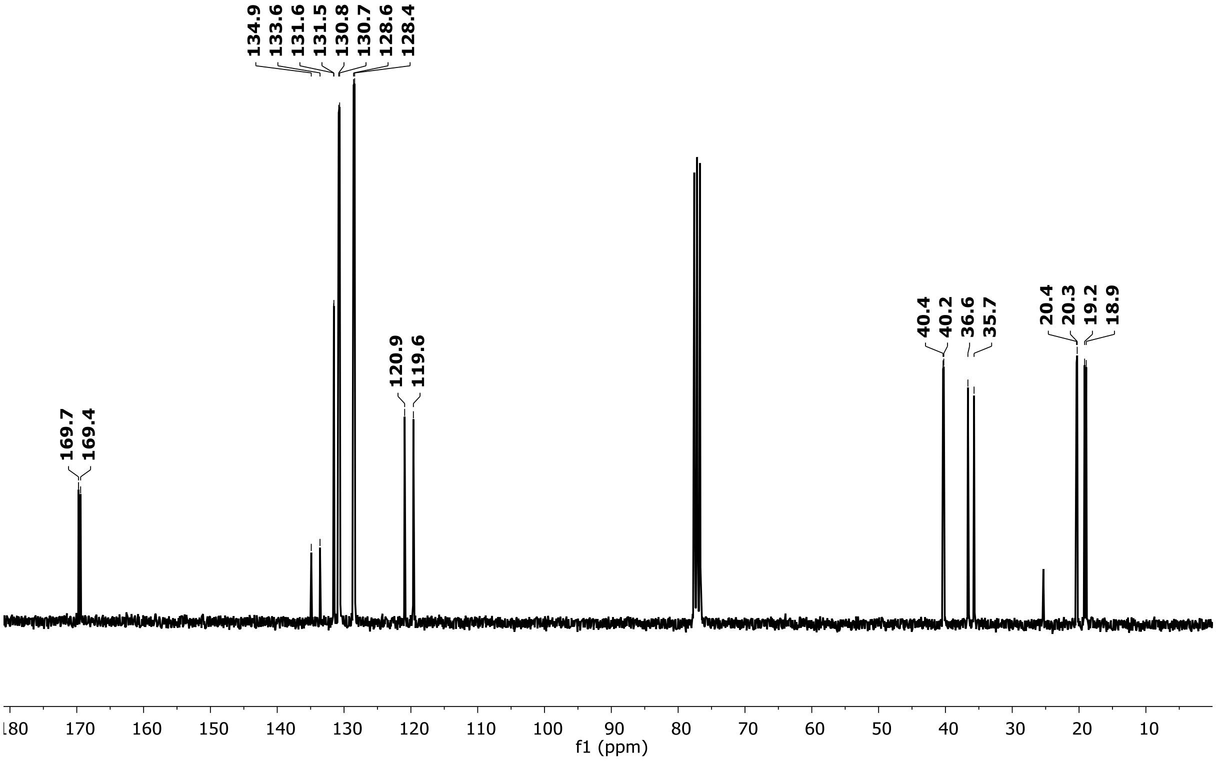

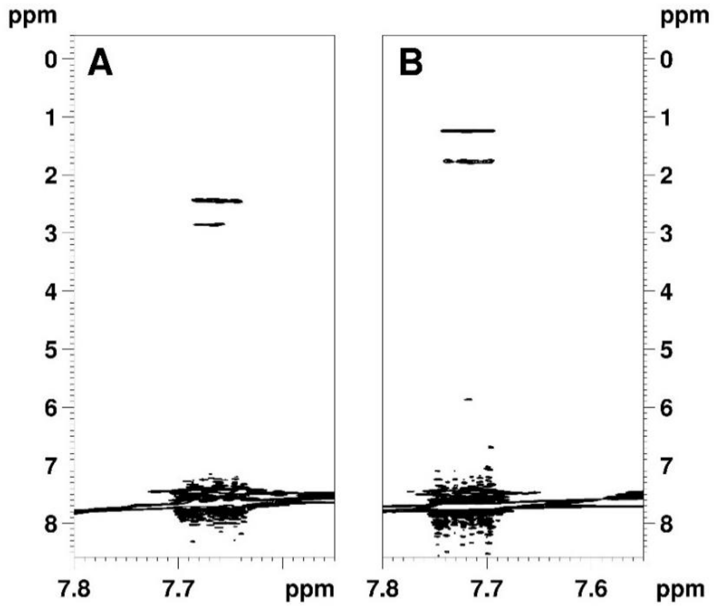

Supplementary Figure 2. 2D strip plots of ^{1}H-^{1}H ROESY NMR spectra of *cis*-**10** (A) and *trans*-**10** (B) diastereomers. These experiments were performed with 300 ms mixing time using the EASY ROESY pulse program [11] on a Bruker Avance II 500 MHz spectrometer equipped with a 5 mm TXI probe.

Theoretical calculations

All computations were carried out with the Gaussian09 program package (G09) [12], using convergence criteria of 3.0×10^{-4}, 4.5×10^{-4}, 1.2×10^{-3} and 1.8×10^{-3}, for the gradients of the root mean square (RMS) Force, Maximum Force, RMS displacement and maximum displacement vectors, respectively. Computations were carried out at MP2 level of theory, including all electrons, with 6-31++G(d,p) and 6-311++G(2d,2p) basis sets. The IEFPCM method was also applied to model the solvent effect, by using the default settings of G09, modelling tetrahydrofuran solvent, which was the best compromise to model neat enviroment. The vibrational frequencies were computed at the same levels of theory, in order to confirm properly all structures as residing at minima on their potential energy hypersurfaces (PESs).

Olefinicity percentage and its resonance enthalpy (OL%)

The “olefinicity scale”, quantifying alkene bond strength on a linear scale [13-15] based on the computed enthalpy of hydrogenation [ΔH_{H2}(OL), Figure S2] of the compound examined (**I**), compared to reference compounds **G** and **H** (Eq. 1). The ΔH_{H2}(OL) value for allyl anion (**G**) is used to define equivalent conjugation (OL% = +100%), while ethylene (**G**) represents complete absence of conjugation (OL% = 0%), This olefinicity value is transformed to resonance enthalpy [H_{RE}(OL); Eq. 2].

$$\mathrm{OL\%} = m_{OL}\, \Delta H_{H2}(\mathrm{OL}) + b_{OL} \qquad \text{Eq. 1}$$

$$H_{RE}(\mathrm{OL}) = \mathrm{OL\%} / m_{OL} \qquad \text{Eq. 2}$$

Here m_{OL} = 0.6978; b_{OL} = 101.8449 at B3LYP/6-31G(d,p) *in vacuo*.

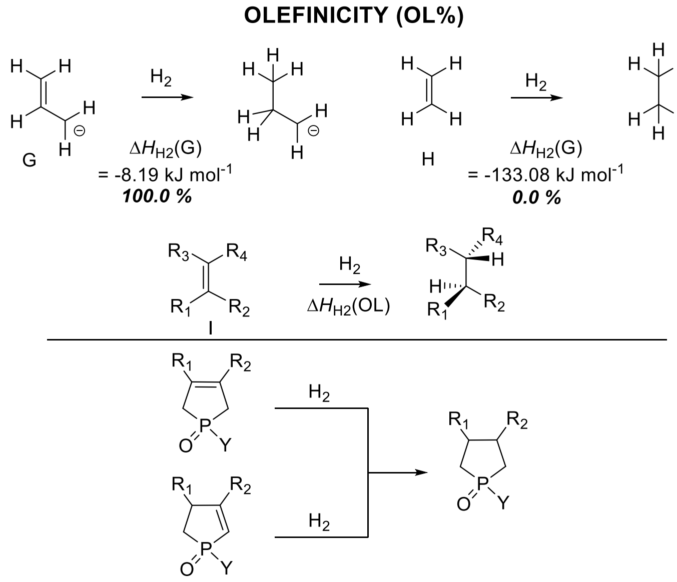

Figure S2. The definition of the olefinicity percentage (OL%) based on the enthalpy of hydrogenation (ΔH_{H2}) of the double bond and the application on compound **1** and **4**.

Tables containing the computed raw data

Table S9. Computed energies (*E*), zero point energies, internal energies (*U*), enthalpies (*H*) and Gibbs free energies (*G*) given in Hartree as well as entropies (*S*) given in J mol^{-1} K^{-1} at MP2(full)/6-311++G(2d,2p) basis set with the consideration of PCM solvent method using the parameter set of THF for compounds **1**, **4, 5, 7, 8** and **10**.

Compound	E	ZPE	U	H	G	S
**1a conf1**	-839.90842081	-842.054577	-842.042239	-842.041295	-842.094038	111.007
**1a conf2**	-839.91135588	-842.059223	-842.046938	-842.045993	-842.099025	111.615
**1c conf1**	-881.50087598	-881.259108	-881.244871	-881.243927	-881.301787	121.777
**1c conf2**	-881.50556663	-881.263741	-881.249605	-881.248661	-881.306701	122.157
**1d conf1**	-1178.88921419	-1178.670663	-1178.654674	-1178.653729	-1178.717437	134.085
**1d conf2**	-1178.89462374	-1178.676011	-1178.660132	-1178.659188	-1178.722045	132.293
**1e conf-1**	-956.60177365	-956.354496	-956.339621	-956.338677	-956.397609	124.034
**1e conf-2**	-956.60643981	-956.359158	-956.344381	-956.343437	-956.402082	123.429
**1f conf-1**	-920.72780977	-920.457095	-920.441902	-920.440957	-920.498767	121.671
**1f conf-2**	-920.72772664	-920.456948	-920.441853	-920.440909	-920.498331	120.856
**1h**	-690.14120065	-689.951428	-689.940949	-689.940005	-689.987030	98.973
**4a**	-839.91530293	-842.060946	-842.048716	-842.047771	-842.099974	109.869
**4c**	-881.50785346	-881.265378	-881.251250	-881.250305	-881.307661	120.715
**4d**	-1178.89698075	-1178.677739	-1178.661860	-1178.660916	-1178.723412	131.534
**4e**	-956.60868917	-956.360753	-956.345972	-956.345028	-956.403400	122.855
**4f conf-1**	-920.72863282	-920.457382	-920.442181	-920.441237	-920.499151	121.889
**4f conf-2**	-920.73012664	-920.458685	-920.443447	-920.442503	-920.500713	122.513
**4h**	-690.14108813	-689.950813	-689.940171	-689.939226	-689.987059	100.672
**5**	-690.14120065	-689.951428	-689.940949	-689.940005	-689.987030	98.973
**7**	-878.95884528	-881.264725	-881.251578	-881.250634	-881.304426	113.216
**8**	-690.14108813	-689.950813	-689.940171	-689.939226	-689.987059	100.672
**10**	-878.96074053	-881.261498	-881.247941	-881.246997	-881.302118	116.011

Table S10.Computed energies (*E*), zero point energies, internal energies (*U*), enthalpies (*H*) and Gibbs free energies (*G*) given in Hartree as well as entropies (*S*) given in J mol^{-1} K^{-1} at MP2(full)/6-311++G(2d,2p) basis set with the consideration of PCM solvent method using the parameter set of THF for olefinicity calculations.

Compound	E	ZPE	U	H	G	S
**H_2**	-1.16294408	-1.15264	-1.15028	-1.149336	-1.164113	31.1
**1a + H2**	-841.09489451	-843.248189	-843.235764	-843.234820	-843.287398	110.66
**1c + H2**	-882.71893500	-882.452522	-882.438234	-882.437289	-882.494636	120.697
**1d + H2**	-1180.10510974	-1179.861842	-1179.846599	-1179.845655	-1179.906738	128.56
**1e + H2**	-957.81977446	-957.547892	-957.532958	-957.532014	-957.590324	122.724
**1f + H2**	-921.94166138	-921.646358	-921.630882	-921.629938	-921.688561	123.383
**1h + H2**	-691.35493046	-691.140541	-691.129867	-691.128923	-691.176565	100.271
**5+H2**	-691.35493046	-691.140541	-691.129867	-691.128923	-691.176565	100.271
**7+H2…**	-879.39244977	-881.668419	-881.653932	-881.652988	-881.710248	120.516
**CH2CH2**	-78.40932103	-78.358042	-78.354991	-78.354047	-78.380222	55.091
**CH3CH3**	-79.63642513	-79.560512	-79.557053	-79.556108	-79.583596	57.852
**CH2CHCH2–**	-117.08603534	-117.023066	-117.018208	-117.017264	-117.048597	65.945
**CH3CH2CH2–**	-118.27193595	-118.183189	-118.178735	-118.177791	-118.208631	64.908

Table S11.Computed energies (*E*), zero point energies, internal energies (*U*), enthalpies (*H*) and Gibbs free energies (*G*) given in Hartree as well as entropies (*S*) given in J mol^{-1} K^{-1} at MP2(full)/6-311++G(2d,2p) basis set with the consideration of PCM solvent method using the parameter set of THF for Mechanism A.

Compound	E	ZPE	U	H	G	S
**TS(1a->11a)**	-839.79735156	-841.975554	-841.963377	-841.962433	-842.014668	109.939
**4a**	-839.84886536	-842.005249	-841.992290	-841.991346	-842.045614	114.218
**TS(11a->4a)**	-839.79223879	-841.969097	-841.957172	-841.956228	-842.008285	109.564
**TS(1h->11h)**	-690.05090464	-689.867731	-689.857275	-689.856331	-689.903433	99.136
**4h**	-690.08478488	-689.897408	-689.886310	-689.885366	-689.933479	101.261
**TS(11h->4h)**	-690.04667857	-689.862618	-689.852511	-689.851567	-689.897885	97.485

Table S12. Computed energies €, zero point energies, internal energies (*U*), enthalpies (*H*) and Gibbs free energies (*G*) given in Hartree as well as entropies (*S*) given in J mol^{-1} K^{-1} at MP2(full)/6-31++G(d,p) basis set with the consideration of PCM solvent method using the parameter set of THF for Mechanism B.

Compound	E	ZPE	U	H	G	S
**12a..**	-1687.24610149	-1686.819387	-1686.792672	-1686.791728	-1686.882427	190.893
**TS(12a→13a)**	-1687.20089645	-1686.776584	-1686.749722	-1686.748778	-1686.839675	191.31
**13a**	-1687.24885069	-1686.821677	-1686.794857	-1686.793913	-1686.885197	192.124
**12h**	-1382.40195784	-1382.025206	-1382.001828	-1382.000883	-1382.082460	171.693
**TS(12h→13h)**	-1382.35587390	-1381.980873	-1381.957648	-1381.956704	-1382.035226	165.263
**13h..**	-1382.40374426	-1382.026511	-1382.003084	-1382.002140	-1382.083354	170.931

Table S13. Computed energies €, zero point energies, internal energies (*U*), enthalpies (*H*) and Gibbs free energies (*G*) given in Hartree as well as entropies (*S*) given in J mol^{-1} K^{-1} at MP2(full)/6-31++G(d,p) basis set with the consideration of PCM solvent method using the parameter set of THF for Mechanism C1.

Compound	E	ZPE	U	H	G	S
**12a..**	-1687.24610149	-1686.819387	-1686.792672	-1686.791728	-1686.882427	190.893
**TS(12a→14a)**	-1687.20089645	-1686.776584	-1686.749722	-1686.748778	-1686.839675	191.31
**14a**	-1687.24885069	-1686.821677	-1686.794857	-1686.793913	-1686.885197	192.124
**TS(14a→4a)**	-1687.24885069	-1686.821677	-1686.794857	-1686.793913	-1686.885197	192.124
**12h**	-1382.40195784	-1382.025206	-1382.001828	-1382.000883	-1382.082460	171.693
**TS(12h→14h)**	-1382.35587390	-1381.980873	-1381.957648	-1381.956704	-1382.035226	165.263
**14h**	-1382.40374426	-1382.026511	-1382.003084	-1382.002140	-1382.083354	170.931
**TS(14h→4h).**	-1382.40374426	-1382.026511	-1382.003084	-1382.002140	-1382.083354	170.931

Table S14. Computed energies €, zero point energies, internal energies (*U*), enthalpies (*H*) and Gibbs free energies (*G*) given in Hartree as well as entropies (*S*) given in J mol^{-1} K^{-1} at MP2(full)/6-31++G(d,p) basis set with the consideration of PCM solvent method using the parameter set of THF for Mechanism C2.

Compound	E	ZPE	U	H	G	S
**12a..**	-1687.24610149	-1686.819387	-1686.792672	-1686.791728	-1686.882427	190.893
**TS(12a→15a)**	-1687.20089645	-1686.776584	-1686.749722	-1686.748778	-1686.839675	191.31
**15a**	-1687.24885069	-1686.821677	-1686.794857	-1686.793913	-1686.885197	192.124
**TS(15a→4a)**	-1687.24885069	-1686.821677	-1686.794857	-1686.793913	-1686.885197	192.124
**15h**	-1382.40195784	-1382.025206	-1382.001828	-1382.000883	-1382.082460	171.693
**TS(15h→14h)**	-1382.35587390	-1381.980873	-1381.957648	-1381.956704	-1382.035226	165.263
**15h**	-1382.40374426	-1382.026511	-1382.003084	-1382.002140	-1382.083354	170.931
**TS(15h→4h).**	-1382.40374426	-1382.026511	-1382.003084	-1382.002140	-1382.083354	170.931

Table S15. Computed energies €, zero point energies, internal energies (*U*), enthalpies (*H*) and Gibbs free energies (*G*) given in Hartree as well as entropies (*S*) given in J mol^{-1} K^{-1} at MP2(full)/6-311++G(2d,2p) basis set with the consideration of PCM solvent method using the parameter set of THF for acidic mechanism.

Compound	E	ZPE	U	H	G	S
**16a..**	-1353.74310607	-1353.489602	-1353.472269	-1353.471325	-1353.536326	136.807
**17a..**	-1353.72587924	-1353.472121	-1353.454385	-1353.453441	-1353.519631	139.308
**TS(17a→18a)**	-1353.67957544	-1353.429306	-1353.412570	-1353.411625	-1353.474868	133.104
**18a**	-1353.73308432	-1353.475612	-1353.459648	-1353.458704	-1353.518416	125.675
**TS(18a→19a)**	-1353.68674540	-1353.436035	-1353.419756	-1353.418812	-1353.479702	128.152
**19a**	-1353.74442467	-1353.490580	-1353.473181	-1353.472237	-1353.537226	136.78
**16h**	-1353.74310607	-1353.489602	-1353.472269	-1353.471325	-1353.536326	136.807
**17h**	-1353.72587924	-1353.472121	-1353.454385	-1353.453441	-1353.519631	139.308
**TS(17h→18h)**	-1353.67957544	-1353.429306	-1353.412570	-1353.411625	-1353.474868	133.104
**18h**	-1353.73308432	-1353.475612	-1353.459648	-1353.458704	-1353.518416	125.675
**TS(18h→19h)**	-1353.68674540	-1353.436035	-1353.419756	-1353.418812	-1353.479702	128.152
**19h**	-1353.74442467	-1353.490580	-1353.473181	-1353.472237	-1353.537226	136.78

Table S16. Computed energies €, zero point energies, internal energies (*U*), enthalpies (*H*) and Gibbs free energies (*G*) given in Hartree as well as entropies (*S*) given in J mol^{-1} K^{-1} at MP2(full)/6-311++G(2d,2p) basis set with the consideration of PCM solvent method using the parameter set of THF for basic mechanism.

Compound	E	ZPE	U	H	G	S
**20a + TEA..**	-981.93153053	-981.673618	-981.658979	-981.658035	-981.714800	113.216
**21a + TEA+H+**	-981.89637853	-981.638635	-981.622701	-981.621756	-981.682431	116.011
**20a + EtOH**	-844.37840769	-844.120495	-844.105856	-844.104912	-844.161677	119.473
**21a + EtO**	-844.38142753	-844.123684	-844.107750	-844.106805	-844.167480	127.701
**20a + KCO3-**	-1553.23731482	-1553.031664	-1553.014448	-1553.013504	-1553.078280	136.333
**21a +HKCO3**	-1553.23429553	-1553.028034	-1553.011066	-1553.010122	-1553.074075	134.601
**20h + TEA**	-981.93153053	-981.673618	-981.658979	-981.658035	-981.714800	113.216
**21h + TEA+H+**	-981.89637853	-981.638635	-981.622701	-981.621756	-981.682431	116.011
**20h + EtOH**	-844.37840769	-844.120495	-844.105856	-844.104912	-844.161677	119.473
**21h + EtO**	-844.38142753	-844.123684	-844.107750	-844.106805	-844.167480	127.701
**20h + KCO3-**	-1553.23731482	-1553.031664	-1553.014448	-1553.013504	-1553.078280	136.333
**21h + KCO3-**	-1553.23429553	-1553.028034	-1553.011066	-1553.010122	-1553.074075	134.601

Additional tables containing XYZ coordinates of computed species

1h:

301aa_POcpent3Me_Et_MP2_6311++2d2p_PCMthf.log

Input orientation:

Center Number	Atomic Number	Atomic Type	Coordinates (Angstroms) X	Y	Z
1	15	0	1.082190	-0.060306	-0.207511
2	6	0	-0.457281	-0.989369	-0.526327
3	1	0	-0.466861	-1.943883	-0.004035
4	1	0	-0.544106	-1.192470	-1.593424
5	6	0	0.267531	1.577148	-0.182958
6	1	0	0.713379	2.240985	0.552891
7	1	0	0.382578	2.042417	-1.160960
8	6	0	-1.161918	1.227024	0.120629
9	1	0	-1.861063	1.982932	0.450837
10	6	0	-1.537044	-0.053259	-0.047504
11	6	0	-2.915371	-0.576517	0.187453
12	1	0	-3.585675	0.211870	0.516564
13	1	0	-3.317267	-1.016477	-0.723318
14	1	0	-2.903628	-1.361394	0.941540
15	8	0	2.240420	-0.253355	-1.146842
16	6	0	1.475798	-0.419840	1.524049
17	1	0	1.686692	-1.486222	1.576421
18	1	0	0.579252	-0.233565	2.112719
19	6	0	2.664489	0.388348	2.035463
20	1	0	2.892024	0.121561	3.062540
21	1	0	3.546815	0.198366	1.433210
22	1	0	2.462453	1.455177	2.008345

1h+H2:

301ab_POcpent3Me_Et_+H2OL_MP2_6311++2d2p_PCMthf.log

Input orientation:

Center Number	Atomic Number	Atomic Type	Coordinates (Angstroms) X	Y	Z
1	15	0	1.083343	-0.112070	-0.176573
2	6	0	-0.452209	-1.084979	0.039190
3	1	0	-0.540034	-1.380192	1.084652
4	1	0	-0.419849	-1.984808	-0.567160
5	6	0	0.238667	1.498850	-0.354223
6	1	0	0.766766	2.298861	0.154584
7	1	0	0.217016	1.727464	-1.417912
8	6	0	-1.176728	1.247182	0.170301
9	1	0	-1.870447	2.020186	-0.151331
10	6	0	-1.598950	-0.136060	-0.333286
11	6	0	-2.939996	-0.587120	0.217465
12	1	0	-3.729817	0.106014	-0.062311
13	1	0	-3.208124	-1.572915	-0.153237
14	1	0	-2.900913	-0.635222	1.304313
15	1	0	-1.179369	1.241934	1.261610
16	1	0	-1.657236	-0.085819	-1.422064
17	8	0	1.997088	-0.515288	-1.304250
18	6	0	1.899205	-0.085273	1.441846
19	1	0	2.111812	-1.119296	1.707002
20	1	0	1.179477	0.291632	2.167327
21	6	0	3.177531	0.746684	1.435099
22	1	0	3.652424	0.726827	2.410824
23	1	0	3.882022	0.360078	0.706283
24	1	0	2.974042	1.784294	1.185652

4h:

302aa_POcpent2Me_Et_MP2_6311++2d2p_PCMthf.log

Input orientation:

Center Number	Atomic Number	Atomic Type	Coordinates (Angstroms) X	Y	Z
1	15	0	1.028165	-0.078987	-0.156952
2	6	0	-0.486530	-1.023279	-0.058201
3	6	0	0.198764	1.499006	0.257193
4	1	0	0.401693	1.732502	1.298731
5	1	0	0.595207	2.303005	-0.353017
6	6	0	-1.302425	1.258415	0.042325
7	1	0	-1.643760	1.689789	-0.898126
8	6	0	-1.566890	-0.227078	0.013918
9	6	0	-2.983270	-0.697577	0.033107
10	1	0	-3.534303	-0.257673	-0.796164
11	1	0	-3.050463	-1.777903	-0.035706
12	1	0	-3.474956	-0.371694	0.947702
13	1	0	-1.892774	1.727383	0.826861
14	1	0	-0.549754	-2.098428	-0.127282
15	8	0	1.772218	-0.126512	-1.466491
16	6	0	2.077037	-0.531092	1.249067
17	1	0	2.376845	-1.566413	1.099846
18	1	0	1.465773	-0.493680	2.148055
19	6	0	3.300640	0.374554	1.364143
20	1	0	3.923656	0.072440	2.199888
21	1	0	3.898733	0.328449	0.460195
22	1	0	3.010984	1.410051	1.522255

11h:

303aab_POcpent3Me_Et_ikerion_MP2_6311++2d2p_PCMthf.log

Input orientation:

Center Number	Atomic Number	Atomic Type	Coordinates (Angstroms) X	Y	Z
1	15	0	-0.900933	-0.140446	0.026800
2	6	0	0.515288	-1.024506	-0.171357
3	6	0	-0.134788	1.514200	-0.023346
4	1	0	-0.422970	1.995982	-0.958588
5	1	0	-0.504877	2.131280	0.792737
6	6	0	1.341040	1.216040	0.038787
7	1	0	2.078447	1.995573	0.145101
8	6	0	1.640921	-0.104302	-0.057319
9	6	0	3.039109	-0.639980	-0.059723
10	1	0	3.766171	0.162286	0.026535
11	1	0	3.188294	-1.335063	0.764315
12	1	0	3.237649	-1.187792	-0.979048
13	8	0	-1.771322	-0.340324	1.412564
14	1	0	-1.167660	-0.522427	2.143133
15	1	0	0.611330	-2.091074	-0.272920
16	6	0	-2.279254	-0.289944	-1.130911
17	1	0	-2.588362	-1.333227	-1.118331
18	1	0	-1.863673	-0.090674	-2.114572
19	6	0	-3.453575	0.637008	-0.826541
20	1	0	-4.215922	0.537620	-1.592851
21	1	0	-3.899190	0.398381	0.132081
22	1	0	-3.138067	1.676518	-0.804881

11h + H2:

303ab_POcpent3Me_Et_ikerion_+H2OL_MP2_6311++2d2p_PCMthf.log

Input orientation:

Center Number	Atomic Number	Atomic Type	Coordinates (Angstroms) X	Y	Z
1	15	0	0.935158	-0.140031	-0.070804
2	6	0	-0.466973	-1.025536	-0.224567
3	6	0	0.170213	1.508775	0.011326
4	1	0	0.629225	2.147893	0.760041
5	1	0	0.305511	1.982233	-0.959506
6	6	0	-1.304469	1.199194	0.288807
7	1	0	-1.949507	2.017809	-0.027958
8	6	0	-1.672804	-0.121433	-0.404808
9	6	0	-2.936946	-0.722111	0.193190
10	1	0	-3.778641	-0.036776	0.101115
11	1	0	-3.204127	-1.651121	-0.304748
12	1	0	-2.778663	-0.938336	1.248162
13	1	0	-1.439610	1.056948	1.360401
14	1	0	-1.880682	0.093799	-1.459801
15	8	0	2.083428	-0.123813	-1.270596
16	1	0	1.639114	-0.190984	-2.124017
17	1	0	-0.520099	-2.096256	-0.339295
18	6	0	2.084839	-0.467059	1.292652
19	1	0	2.300296	-1.534024	1.259960
20	1	0	1.508596	-0.292881	2.197997
21	6	0	3.370005	0.354621	1.286898
22	1	0	3.953543	0.144608	2.178394
23	1	0	3.975436	0.123022	0.418716
24	1	0	3.156755	1.419876	1.273750

16Ah:

341aa_POcpent3Me_Et_MsOH_MP2_6311++2d2p_PCMthf.log

Input orientation:

Center Number	Atomic Number	Atomic Type	Coordinates (Angstroms) X	Y	Z
1	15	0	-1.176463	-0.976932	-0.107595
2	6	0	-1.678380	0.470981	-1.083014
3	1	0	-2.434831	0.209783	-1.820235
4	1	0	-0.813635	0.880173	-1.601672
5	6	0	-1.057441	-0.110207	1.488905
6	1	0	-1.432700	-0.721465	2.305431
7	1	0	-0.015293	0.130102	1.686448
8	6	0	-1.882602	1.121342	1.239015
9	1	0	-2.194948	1.749035	2.061847
10	6	0	-2.193144	1.423203	-0.033619
11	6	0	-2.974293	2.621564	-0.459221
12	1	0	-3.272749	3.220547	0.395763
13	1	0	-2.381558	3.242223	-1.128552
14	1	0	-3.866968	2.323911	-1.006136
15	8	0	0.049719	-1.751258	-0.571997
16	6	0	-2.626162	-2.044774	-0.002824
17	1	0	-2.863494	-2.347045	-1.020635
18	1	0	-3.449847	-1.430366	0.357393
19	8	0	1.996182	-0.619505	-1.663840
20	1	0	1.179586	-1.081358	-1.227824
21	8	0	1.286660	1.239614	-0.207068
22	6	0	3.265671	-0.263554	0.590614
23	8	0	3.431519	1.324448	-1.491918
24	16	0	2.456719	0.563087	-0.743622
25	1	0	2.545933	-0.918989	1.065625
26	1	0	4.098502	-0.826021	0.189863
27	1	0	3.609806	0.494111	1.283401
28	6	0	-2.397412	-3.262722	0.887663
29	1	0	-3.285991	-3.884893	0.908213
30	1	0	-1.572570	-3.861753	0.516545
31	1	0	-2.172169	-2.972971	1.909763

17h:

342ab_POcpent3Me_Et+H+_MsO_B_SCANeleje_HO1_MP2_6311++2d2p_PCMthf.log

Input orientation:

Center Number	Atomic Number	Atomic Type	Coordinates (Angstroms) X	Y	Z
1	15	0	-2.205691	-0.609782	-0.301928
2	6	0	-0.958222	0.391449	-1.185534
3	1	0	-1.399395	0.952537	-2.006568
4	1	0	-0.185442	-0.255912	-1.597801
5	6	0	-1.479855	-0.326860	1.357087
6	1	0	-2.244684	-0.208661	2.119776
7	1	0	-0.873621	-1.189333	1.631653
8	6	0	-0.663922	0.919632	1.154787
9	1	0	1.200760	0.008860	0.905213
10	6	0	-0.398716	1.291126	-0.115847
11	6	0	0.368279	2.511851	-0.502231
12	1	0	0.746874	3.035719	0.370984
13	1	0	1.201419	2.245651	-1.149031
14	1	0	-0.270827	3.190319	-1.064431
15	8	0	-2.416861	-2.031999	-0.735426
16	6	0	-3.704168	0.407289	-0.332574
17	1	0	-3.997822	0.490415	-1.377239
18	1	0	-3.433504	1.404549	0.011326
19	8	0	2.277985	-0.245868	-1.283218
20	8	0	3.626812	-1.923024	0.010318
21	6	0	4.130944	0.621283	0.357850
22	8	0	1.945183	-0.586226	1.142440
23	16	0	2.978826	-0.639499	-0.078993
24	1	0	4.894478	0.633392	-0.410040
25	1	0	3.608505	1.568422	0.392661
26	1	0	4.557956	0.370844	1.319737
27	6	0	-4.836176	-0.187140	0.500015
28	1	0	-5.723114	0.434585	0.433249
29	1	0	-5.092337	-1.181052	0.148293
30	1	0	-4.563285	-0.262103	1.548712
31	1	0	-0.323402	1.507426	1.997453

TS(17h->18h):

342ae_POcpent3Me_Et+H+_MsO_B_MP2_6311++2d2p_PCMthf_TS_HO1_uj.log

Input orientation:

Center Number	Atomic Number	Atomic Type	Coordinates (Angstroms) X	Y	Z
1	15	0	-2.311445	-0.582546	-0.369468
2	6	0	-0.924757	0.223688	-1.294650
3	1	0	-1.171140	0.787730	-2.185807
4	1	0	-0.273228	-0.612057	-1.564048
5	6	0	-1.246678	-0.807592	1.098804
6	1	0	-1.812656	-0.871125	2.021128
7	1	0	-0.699774	-1.738147	0.968300
8	6	0	-0.321916	0.405595	1.049688
9	1	0	0.785177	0.040492	1.148397
10	6	0	-0.259937	0.995266	-0.243816
11	6	0	0.216900	2.362354	-0.468344
12	1	0	0.823733	2.725484	0.352784
13	1	0	0.731871	2.449936	-1.417324
14	1	0	-0.686837	2.979288	-0.527107
15	8	0	-2.951769	-1.774787	-1.001620
16	6	0	-3.453177	0.759271	0.040182
17	1	0	-3.826762	1.148982	-0.904680
18	1	0	-2.893101	1.558812	0.523534
19	8	0	1.923521	0.106077	-1.020233
20	8	0	3.667016	-1.559710	-0.467587
21	6	0	4.073543	0.920573	0.212907
22	8	0	2.245970	-0.582132	1.300376
23	16	0	2.906282	-0.394336	-0.028864
24	1	0	4.561758	1.115248	-0.733726
25	1	0	3.539897	1.798341	0.554751
26	1	0	4.796298	0.601688	0.953144

27	6	0	-4.601545	0.278543	0.924386
28	1	0	-5.283588	1.096260	1.131289
29	1	0	-5.158194	-0.513592	0.435460
30	1	0	-4.238321	-0.100241	1.875203
31	1	0	-0.381465	1.130105	1.857097

18h:

342ba_POcpent3Me_Et+H+_MsO_B_MP2_6311++2d2p_PCMthf.log

Input orientation:

Center Number	Atomic Number	Atomic Type	Coordinates (Angstroms) X	Y	Z
1	15	0	-1.744970	-0.544284	-0.368768
2	6	0	-0.551349	0.548858	-1.228680
3	1	0	-1.079510	1.250176	-1.868372
4	1	0	0.086836	-0.063817	-1.854588
5	6	0	-0.937508	-0.420704	1.257868
6	1	0	-1.649549	-0.536796	2.068415
7	1	0	-0.203932	-1.216366	1.331349
8	6	0	-0.295483	0.965930	1.239887
9	1	0	0.480926	1.079225	1.989547
10	6	0	0.264347	1.291832	-0.157781
11	6	0	0.301285	2.784349	-0.396546
12	1	0	0.855912	3.278607	0.396032
13	1	0	0.770041	3.001933	-1.351344
14	1	0	-0.714063	3.170625	-0.409771
15	8	0	-1.989789	-1.908377	-0.947436
16	6	0	-3.254088	0.453707	-0.235691
17	1	0	-3.600210	0.616261	-1.254935
18	1	0	-2.989961	1.428604	0.171226
19	8	0	1.698375	0.940911	-0.265853
20	8	0	1.490836	-1.537721	-0.524772
21	6	0	3.841257	-0.332099	-0.610532
22	8	0	2.407539	-0.588654	1.580516
23	16	0	2.259195	-0.506430	0.142900
24	1	0	3.703024	-0.215198	-1.676601
25	1	0	4.335339	0.524785	-0.172795
26	1	0	4.383187	-1.243328	-0.386806
27	6	0	-4.335540	-0.224033	0.599847
28	1	0	-5.238517	0.377798	0.615388
29	1	0	-4.584157	-1.197146	0.189354
30	1	0	-4.013805	-0.364781	1.627678
31	1	0	-1.056935	1.713420	1.454255

TS(18h->19h):

342cb_POcpent3Me_Et+H+_MsO_B_MP2_6311++2d2p_PCMthf_TS_HO2.log

Input orientation:

Center Number	Atomic Number	Atomic Type	Coordinates (Angstroms) X	Y	Z
1	15	0	-1.844490	-0.605715	-0.262507
2	6	0	-0.481489	0.380800	-0.992713
3	1	0	-0.421633	0.586062	-2.053265
4	1	0	0.389856	-0.406106	-0.743697
5	6	0	-1.296994	-0.163053	1.417723
6	1	0	-2.087033	-0.241798	2.155406
7	1	0	-0.501502	-0.858449	1.676086
8	6	0	-0.751418	1.254020	1.268423
9	1	0	-0.039999	1.543930	2.033914
10	6	0	-0.169458	1.418422	-0.093591
11	6	0	0.354399	2.728052	-0.515949
12	1	0	0.823806	3.256995	0.304025
13	1	0	1.032357	2.636195	-1.355916
14	1	0	-0.513726	3.303893	-0.849352
15	8	0	-1.914558	-2.050070	-0.643247
16	6	0	-3.368617	0.309205	-0.598893
17	1	0	-3.501009	0.292838	-1.678816
18	1	0	-3.223381	1.348008	-0.308118
19	8	0	1.894328	0.847788	0.695164

20	8	0	1.527696	-1.311153	-0.375623
21	6	0	3.625453	0.059204	-1.089953
22	8	0	3.367598	-1.059323	1.252953
23	16	0	2.552403	-0.406587	0.241123
24	1	0	3.027582	0.525940	-1.863211
25	1	0	4.361621	0.752438	-0.702973
26	1	0	4.105499	-0.835175	-1.466434
27	6	0	-4.574973	-0.305838	0.106764
28	1	0	-5.477059	0.243216	-0.142031
29	1	0	-4.711462	-1.338695	-0.195011
30	1	0	-4.457444	-0.281659	1.186300
31	1	0	-1.565880	1.986089	1.308806

19h:

342cc_POcpent3Me_Et+H+_MsO_B_SCAN_HOvege_MP2_6311++2d2p_PCMthf.log

Input orientation:

Center Number	Atomic Number	Atomic Type	Coordinates (Angstroms) X	Y	Z
1	15	0	-1.415916	-0.742166	-0.222284
2	6	0	-1.669274	0.860984	-0.939079
3	1	0	-1.822308	1.047735	-1.990230
4	1	0	0.925438	-0.820823	-1.251655
5	6	0	-1.240207	-0.132120	1.484555
6	1	0	-1.998426	-0.578593	2.119972
7	1	0	-0.265101	-0.436467	1.848118
8	6	0	-1.368425	1.398777	1.411791
9	1	0	-0.472568	1.885246	1.789599
10	6	0	-1.594879	1.831569	-0.012623
11	6	0	-1.712721	3.290834	-0.295953
12	1	0	-0.794865	3.795611	0.000297
13	1	0	-1.896230	3.484961	-1.346999
14	1	0	-2.519208	3.726017	0.290898
15	8	0	-0.235219	-1.542102	-0.769726
16	6	0	-2.924239	-1.723852	-0.349655
17	1	0	-3.135356	-1.845536	-1.409979
18	1	0	-3.729819	-1.130287	0.077820
19	8	0	3.597146	1.116636	-0.907816
20	8	0	1.784913	-0.337579	-1.589003
21	6	0	3.187979	-1.072430	0.483242
22	8	0	1.542295	0.951071	0.504670
23	16	0	2.515217	0.319691	-0.370872
24	1	0	3.870126	-1.585724	-0.181399
25	1	0	3.707785	-0.700422	1.357200
26	1	0	2.365811	-1.716153	0.770978
27	6	0	-2.794245	-3.079483	0.340632
28	1	0	-3.716609	-3.642493	0.242764
29	1	0	-1.991986	-3.660538	-0.101230
30	1	0	-2.584692	-2.965520	1.400638
31	1	0	-2.200338	1.747193	2.021929

TS(1h->11h):

352aa_POcpent3Me_Et_MP2_6311++2d2p_PCMthf_TS_HO.log

Input orientation:

Center Number	Atomic Number	Atomic Type	Coordinates (Angstroms) X	Y	Z
1	15	0	-0.971774	-0.071987	0.005438
2	6	0	0.472464	-0.991879	-0.364386
3	1	0	0.536156	-1.743721	-1.138214
4	1	0	-0.241313	-1.390499	0.985622
5	6	0	-0.206716	1.557300	0.230087
6	1	0	-0.497404	2.229637	-0.576804
7	1	0	-0.535798	1.996635	1.169874
8	6	0	1.262572	1.214729	0.204950
9	1	0	2.004600	1.948747	0.482503
10	6	0	1.586615	-0.051871	-0.151978
11	6	0	2.995968	-0.544835	-0.256905
12	1	0	3.708364	0.247040	-0.045000

13	1	0	3.167637	-1.364474	0.437613
14	1	0	3.191541	-0.927107	-1.257009
15	8	0	-1.262073	-0.821139	1.373232
16	6	0	-2.441623	-0.008587	-1.031513
17	1	0	-2.726602	-1.038655	-1.231536
18	1	0	-2.154584	0.444938	-1.976689
19	6	0	-3.582044	0.759945	-0.364950
20	1	0	-4.452546	0.773263	-1.012690
21	1	0	-3.863107	0.295415	0.574472
22	1	0	-3.297883	1.788768	-0.164203

20h:
363aa_POcpent3Me_Et_+KCO3-_MP2_6311++2d2p_PCMthf.log

Input orientation:

Center Number	Atomic Number	Atomic Type	Coordinates (Angstroms) X	Y	Z
1	15	0	0.803324	-0.880138	-0.339847
2	6	0	1.008553	0.911188	-0.565411
3	1	0	0.349254	1.478507	0.094039
4	1	0	0.757135	1.173595	-1.593586
5	6	0	2.562531	-1.282908	-0.652675
6	1	0	2.903973	-2.111332	-0.037149
7	1	0	2.683679	-1.571470	-1.696320
8	6	0	3.243802	0.019025	-0.331362
9	1	0	4.309285	0.062201	-0.149721
10	6	0	2.473462	1.121297	-0.285272
11	6	0	2.971951	2.496980	0.010642
12	1	0	2.747820	3.169513	-0.815419
13	1	0	2.470974	2.899475	0.888650
14	1	0	4.044538	2.502096	0.183569
15	8	0	-0.218017	-1.594880	-1.197815
16	6	0	0.549467	-1.110611	1.430519
17	1	0	-0.393903	-0.601512	1.642696
18	1	0	1.349262	-0.580997	1.945628
19	6	0	0.493556	-2.580289	1.834413
20	1	0	0.304612	-2.672441	2.899752
21	1	0	-0.305549	-3.094069	1.308616
22	1	0	1.423964	-3.099935	1.618425
23	8	0	-2.294913	0.328428	1.208377
24	8	0	-2.356613	1.758516	-0.525573
25	6	0	-1.981361	1.491840	0.697190
26	8	0	-1.285317	2.339503	1.373620
27	19	0	-2.784867	-0.747501	-1.115944

20h:
363ab_POcpent3Me_Et_+KCO3-_MP2_6311++2d2p_PCMthf.log

Input orientation:

Center Number	Atomic Number	Atomic Type	Coordinates (Angstroms) X	Y	Z
1	15	0	1.400543	-0.481213	-0.026524
2	6	0	0.235271	0.819041	0.463022
3	1	0	0.076537	0.842309	1.539019
4	1	0	-0.728906	0.644237	-0.028221
5	6	0	2.145160	0.474422	-1.395726
6	1	0	3.208457	0.275815	-1.503201
7	1	0	1.656614	0.187662	-2.326290
8	6	0	1.834549	1.891333	-0.996864
9	1	0	2.342120	2.725279	-1.461963
10	6	0	0.883679	2.071203	-0.062400
11	6	0	0.417807	3.395989	0.441633
12	1	0	0.930303	4.213256	-0.057869
13	1	0	-0.653696	3.500023	0.284558
14	1	0	0.589562	3.477768	1.513686
15	8	0	0.853496	-1.850124	-0.359031
16	6	0	2.659881	-0.520590	1.275695
17	1	0	2.148213	-0.828150	2.185748
18	1	0	3.005761	0.500791	1.424997

19	6	0	3.813130	-1.467146	0.958948
20	1	0	4.526168	-1.487519	1.777159
21	1	0	3.449468	-2.477552	0.802181
22	1	0	4.346356	-1.159494	0.063915
23	8	0	-3.077208	-0.690182	0.927457
24	8	0	-3.408209	1.510534	0.547273
25	6	0	-3.015082	0.355852	0.146125
26	8	0	-2.508201	0.201808	-1.055201
27	19	0	-1.798660	-2.282611	-0.681439

21h:

363bb_POcpent3Me_Et_+KHCO3_MP2_6311++2d2p_PCMthf.log

Input orientation:

Center Number	Atomic Number	Atomic Type	Coordinates (Angstroms) X	Y	Z
1	15	0	1.705300	0.684621	-0.000656
2	6	0	1.321472	-0.815655	-0.787559
3	6	0	2.727585	-0.090580	1.319688
4	1	0	3.594495	0.519347	1.565057
5	1	0	2.100575	-0.141107	2.215373
6	6	0	3.044315	-1.454046	0.760469
7	1	0	3.689420	-2.157971	1.267228
8	6	0	2.274134	-1.793004	-0.317880
9	6	0	2.377088	-3.133755	-0.984746
10	1	0	3.137407	-3.751772	-0.513482
11	1	0	1.425778	-3.661638	-0.940139
12	1	0	2.629346	-3.023802	-2.038521
13	8	0	0.577240	1.602585	0.472006
14	6	0	2.889583	1.676137	-0.979913
15	1	0	2.394210	1.911817	-1.920869
16	1	0	3.722112	1.011887	-1.212042
17	6	0	3.368650	2.948006	-0.288496
18	1	0	4.091301	3.482039	-0.900364
19	1	0	2.534209	3.613113	-0.090775
20	1	0	3.845507	2.724026	0.662565
21	8	0	-4.090129	-0.677733	1.012645
22	8	0	-5.998665	-0.071934	-0.063854
23	6	0	-4.769327	-0.143966	0.110622
24	8	0	-3.965156	0.488590	-0.883551
25	19	0	-1.541725	-0.057970	0.303397
26	1	0	0.936447	-0.866702	-1.797916
27	1	0	-4.591009	0.861420	-1.511408

20h

364aa_POcpent3Me_Et_+EtO-_MP2_6311++2d2p_PCMthf.log

Input orientation:

Center Number	Atomic Number	Atomic Type	Coordinates (Angstroms) X	Y	Z
1	15	0	-0.978904	-0.751342	-0.227573
2	6	0	0.255966	0.072416	-1.269409
3	1	0	-0.126044	0.237342	-2.276510
4	1	0	1.204727	-0.504425	-1.340617
5	6	0	-0.540615	0.159555	1.300811
6	1	0	-1.415111	0.364775	1.913721
7	1	0	0.146315	-0.448175	1.888812
8	6	0	0.113288	1.396131	0.753005
9	1	0	0.282632	2.264177	1.376248
10	6	0	0.504616	1.360876	-0.534242
11	6	0	1.193561	2.481392	-1.239829
12	1	0	1.342443	3.333393	-0.581661
13	1	0	2.161803	2.150880	-1.611592
14	1	0	0.614092	2.805339	-2.103328
15	8	0	-1.031212	-2.255293	-0.156897
16	6	0	-2.565136	-0.026068	-0.742777
17	1	0	-2.711902	-0.322026	-1.780134
18	1	0	-2.454073	1.056726	-0.725089
19	6	0	-3.738298	-0.486366	0.115948

20	1	0	-4.669369	-0.056224	-0.240780
21	1	0	-3.833076	-1.567125	0.088840
22	1	0	-3.613265	-0.188088	1.153219
23	8	0	2.964897	-1.187540	-1.181747
24	6	0	2.859843	-1.370339	0.179590
25	6	0	3.546708	-0.275387	1.004839
26	1	0	3.277521	-2.337934	0.518717
27	1	0	1.803477	-1.400655	0.520441
28	1	0	3.433133	-0.445096	2.078058
29	1	0	4.609527	-0.242485	0.772198
30	1	0	3.112952	0.692870	0.762799

21h:
364ab_POcpent3Me_Et_+EtOH_MP2_6311++2d2p_PCMthf.log

Input orientation:

Center Number	Atomic Number	Atomic Type	Coordinates (Angstroms) X	Y	Z
1	15	0	-1.111919	-0.536702	-0.375645
2	6	0	-0.026744	0.563248	-1.188888
3	6	0	-0.672236	0.051102	1.313453
4	1	0	-1.533195	0.033052	1.978483
5	1	0	0.066808	-0.649504	1.713631
6	6	0	-0.094472	1.417977	1.056499
7	1	0	0.177617	2.100200	1.850304
8	6	0	0.230265	1.635997	-0.253617
9	6	0	0.876323	2.906848	-0.720358
10	1	0	1.019519	3.603071	0.102889
11	1	0	1.845240	2.699792	-1.172242
12	1	0	0.267527	3.394020	-1.481098
13	8	0	-1.063046	-2.038530	-0.609637
14	6	0	-2.839875	0.034023	-0.591332
15	1	0	-3.049971	-0.019233	-1.658783
16	1	0	-2.856666	1.087109	-0.310282
17	6	0	-3.865183	-0.771270	0.198921
18	1	0	-4.873954	-0.398886	0.037613
19	1	0	-3.836341	-1.815122	-0.096417
20	1	0	-3.665045	-0.724847	1.266609
21	8	0	2.701036	-0.395143	-0.925496
22	6	0	2.779189	-0.835568	0.426737
23	6	0	4.218252	-1.167018	0.748721
24	1	0	2.154369	-1.718062	0.576239
25	1	0	2.409923	-0.055449	1.094910
26	1	0	4.311183	-1.519088	1.773064
27	1	0	4.585507	-1.944027	0.084406
28	1	0	4.846623	-0.288963	0.629180
29	1	0	-0.137411	0.787899	-2.242640
30	1	0	1.753116	-0.129564	-1.077653

1f
1301baa_POcpent3Me_odiMe_MP2_6311++2d2p_PCMthf.log

Input orientation:

Center Number	Atomic Number	Atomic Type	Coordinates (Angstroms) X	Y	Z
1	15	0	-0.659011	-0.251331	0.629430
2	6	0	-1.751174	1.002950	-0.140623
3	1	0	-1.279234	1.446645	-1.016642
4	1	0	-1.981494	1.798212	0.563070
5	6	0	-1.395014	-1.630607	-0.332601
6	1	0	-0.828805	-1.807742	-1.245782
7	1	0	-1.394071	-2.544764	0.253059
8	6	0	-2.775223	-1.117969	-0.628905
9	1	0	-3.566292	-1.794936	-0.919523
10	6	0	-2.967682	0.206220	-0.534117
11	6	0	-4.252794	0.915422	-0.806560
12	1	0	-5.033361	0.218445	-1.096116
13	1	0	-4.582385	1.459892	0.076265
14	1	0	-4.126600	1.645905	-1.603584

15	6	0	1.064298	0.018762	0.110691
16	6	0	1.905503	-1.087046	-0.139017
17	6	0	1.578506	1.330573	0.024492
18	6	0	3.210157	-0.857508	-0.581031
19	6	0	2.889208	1.514553	-0.421111
20	6	0	3.698102	0.432981	-0.748874
21	1	0	3.852909	-1.705034	-0.773168
22	1	0	3.281386	2.519548	-0.488469
23	8	0	-0.822659	-0.379445	2.119745
24	1	0	4.708657	0.592996	-1.094004
25	6	0	1.488340	-2.515757	0.100496
26	1	0	0.830734	-2.607877	0.959503
27	1	0	0.984495	-2.945695	-0.761109
28	1	0	2.369375	-3.119077	0.296565
29	6	0	0.803987	2.554104	0.443963
30	1	0	0.190119	2.947549	-0.361995
31	1	0	0.160046	2.354962	1.295085
32	1	0	1.498597	3.336071	0.735145

1f:

1301bab_POcpent3Me_odiMe_MP2_6311++2d2p_PCMthf.log

Input orientation:

Center Number	Atomic Number	Atomic Type	Coordinates (Angstroms) X	Y	Z
1	15	0	0.559301	-0.892433	0.064168
2	6	0	1.742321	-0.101436	-1.085233
3	1	0	1.333830	0.761998	-1.604692
4	1	0	2.007039	-0.843754	-1.837202
5	6	0	1.420346	-0.388577	1.603001
6	1	0	0.868471	0.363208	2.163949
7	1	0	1.507793	-1.272791	2.231506
8	6	0	2.749140	0.105781	1.109097
9	1	0	3.543481	0.333585	1.806948
10	6	0	2.918553	0.257475	-0.216358
11	6	0	4.174139	0.745884	-0.861386
12	1	0	4.936370	0.971904	-0.121794
13	1	0	4.566333	-0.001610	-1.548288
14	1	0	3.978561	1.643899	-1.444957
15	6	0	-1.049232	-0.034067	-0.017928
16	6	0	-2.250509	-0.774972	-0.130797
17	6	0	-1.094579	1.376678	0.054853
18	6	0	-3.462576	-0.080864	-0.176869
19	6	0	-2.333260	2.021982	0.011689
20	6	0	-3.515557	1.305953	-0.106929
21	1	0	-4.378914	-0.647082	-0.262833
22	1	0	-2.359702	3.100983	0.074543
23	8	0	0.501322	-2.383249	-0.110134
24	6	0	0.115370	2.274818	0.147967
25	1	0	0.999068	1.800078	0.553894
26	1	0	0.377098	2.661010	-0.835838
27	1	0	-0.117172	3.129550	0.777000
28	6	0	-2.324361	-2.279982	-0.200745
29	1	0	-1.794577	-2.670180	-1.062063
30	1	0	-1.881707	-2.747308	0.671712
31	1	0	-3.366002	-2.579812	-0.265191
32	1	0	-4.465304	1.818788	-0.139243

1f+H2:

1301bba_POcpent3Me_odiMe_+H2OL_MP2_6311++2d2p_PCMthf.log

Input orientation:

Center Number	Atomic Number	Atomic Type	Coordinates (Angstroms) X	Y	Z
1	15	0	-0.601749	-0.351812	0.662706
2	6	0	-1.798793	0.953875	0.228986
3	1	0	-1.463874	1.495105	-0.655358
4	1	0	-1.943104	1.651912	1.046150
5	6	0	-1.297247	-1.559764	-0.542213
6	1	0	-0.584818	-1.826438	-1.313214
7	1	0	-1.530233	-2.456062	0.027493
8	6	0	-2.561394	-0.904438	-1.117966
9	1	0	-3.328911	-1.642738	-1.335204
10	6	0	-3.053110	0.152065	-0.129174
11	6	0	-4.161561	1.023413	-0.692681
12	1	0	-5.025474	0.422947	-0.967660
13	1	0	-4.484201	1.769080	0.029044
14	1	0	-3.812737	1.542655	-1.583684
15	6	0	1.091983	0.063400	0.137690
16	6	0	1.966477	-1.007359	-0.152542
17	6	0	1.559495	1.393593	0.069717
18	6	0	3.256162	-0.726523	-0.607758
19	6	0	2.856373	1.628688	-0.393765
20	6	0	3.697319	0.582753	-0.755418
21	1	0	3.922966	-1.548239	-0.829054
22	1	0	3.213376	2.647517	-0.447580
23	1	0	-2.316911	-0.401299	-2.053808
24	1	0	-3.411643	-0.350997	0.770767
25	8	0	-0.683836	-0.798202	2.100449
26	6	0	1.599377	-2.457043	0.048406
27	1	0	0.839276	-2.588828	0.811506
28	1	0	1.244362	-2.915129	-0.871594
29	1	0	2.480279	-3.007886	0.365104
30	6	0	0.758651	2.587022	0.523953
31	1	0	0.073385	2.939979	-0.241718
32	1	0	0.183147	2.372455	1.418591
33	1	0	1.434712	3.403856	0.757056
34	1	0	4.696322	0.785183	-1.111835

1f + H2:

1301bbb_POcpent3Me_odiMe_+H2OL_MP2_6311++2d2p_PCMthf.log

Input orientation:

Center Number	Atomic Number	Atomic Type	Coordinates (Angstroms) X	Y	Z
1	15	0	-0.548520	0.760066	0.307267
2	6	0	-1.640769	0.296427	-1.075106
3	1	0	-1.337321	-0.645010	-1.529180
4	1	0	-1.614178	1.071994	-1.835406
5	6	0	-1.521587	-0.111957	1.597565
6	1	0	-0.965619	-0.858929	2.149625
7	1	0	-1.784947	0.685115	2.288981
8	6	0	-2.768069	-0.659544	0.885626
9	1	0	-3.638675	-0.625774	1.535929
10	6	0	-3.008529	0.154727	-0.393690
11	6	0	-4.054890	-0.475332	-1.296455
12	1	0	-5.005624	-0.576725	-0.778190
13	1	0	-4.218642	0.122807	-2.188843
14	1	0	-3.732321	-1.467288	-1.608175
15	6	0	1.110074	0.026447	0.064000
16	6	0	1.296411	-1.375819	0.082386
17	6	0	2.219063	0.877530	-0.169427
18	6	0	2.579391	-1.898498	-0.098308
19	6	0	3.482536	0.303390	-0.338620
20	6	0	3.675167	-1.071153	-0.297506
21	1	0	2.709374	-2.971436	-0.086344

22	1	0	4.325577	0.955753	-0.515491
23	1	0	-2.618372	-1.702474	0.611457
24	1	0	-3.341600	1.152282	-0.103076
25	8	0	-0.531852	2.242874	0.562107
26	6	0	2.145078	2.381250	-0.271650
27	1	0	1.415660	2.702473	-1.006667
28	1	0	1.856185	2.835377	0.668918
29	1	0	3.119950	2.762070	-0.561376
30	6	0	0.193595	-2.381900	0.292981
31	1	0	0.004433	-2.552228	1.349978
32	1	0	-0.741603	-2.093223	-0.168354
33	1	0	0.487598	-3.333397	-0.139655
34	1	0	4.661088	-1.490309	-0.432732

4f:

1302ba_POcpent2Me_odiMe_MP2_6311++2d2p_PCMthf.log

Input orientation:

Center Number	Atomic Number	Atomic Type	Coordinates (Angstroms) X	Y	Z
1	15	0	0.549535	-0.961761	0.029440
2	6	0	1.733394	-0.156637	-1.035852
3	6	0	1.442632	-0.528151	1.569752
4	1	0	0.854189	0.146745	2.181722
5	1	0	1.569477	-1.452499	2.125018
6	6	0	2.786487	0.082447	1.139101
7	1	0	3.625203	-0.539783	1.447683
8	6	0	2.820159	0.248932	-0.359469
9	6	0	4.031052	0.869284	-0.971975
10	1	0	4.921524	0.303471	-0.704499
11	1	0	3.956909	0.916293	-2.053092
12	1	0	4.168618	1.878056	-0.585037
13	6	0	-1.037262	-0.051643	-0.016746
14	6	0	-2.261267	-0.756271	-0.126166
15	6	0	-1.047132	1.362922	0.066249
16	6	0	-3.455532	-0.029571	-0.164004
17	6	0	-2.269371	2.039149	0.027832
18	6	0	-3.471911	1.356519	-0.090982
19	1	0	-4.386867	-0.571315	-0.246469
20	1	0	-2.268414	3.117769	0.095082
21	1	0	2.943977	1.054000	1.607634
22	1	0	1.639714	-0.090479	-2.109001
23	8	0	0.404983	-2.438638	-0.210681
24	6	0	0.186153	2.225169	0.178378
25	1	0	0.887545	1.871386	0.925303
26	1	0	0.720222	2.280119	-0.765825
27	1	0	-0.108094	3.232681	0.456476
28	6	0	-2.387799	-2.258185	-0.198309
29	1	0	-1.897444	-2.662191	-1.076187
30	1	0	-1.935596	-2.744176	0.658618
31	1	0	-3.440867	-2.521231	-0.232271
32	1	0	-4.407229	1.895544	-0.119591

4f:

1302bb_POcpent2Me_odiMe_MP2_6311++2d2p_PCMthf.log

Input orientation:

Center Number	Atomic Number	Atomic Type	Coordinates (Angstroms) X	Y	Z
1	15	0	-0.621641	-0.496926	0.680565
2	6	0	-1.886889	0.755997	0.509845
3	6	0	-1.272464	-1.484072	-0.726942
4	1	0	-0.611697	-1.345581	-1.577116
5	1	0	-1.274577	-2.536525	-0.465321
6	6	0	-2.673525	-0.942656	-1.033441
7	1	0	-3.448823	-1.625249	-0.686573
8	6	0	-2.859401	0.377817	-0.337540
9	6	0	-4.110169	1.150464	-0.594877
10	1	0	-4.981470	0.538611	-0.368711

11	1	0	-4.154006	2.056937	-0.000898
12	1	0	-4.174964	1.416883	-1.648252
13	6	0	1.029954	0.080007	0.160639
14	6	0	1.963568	-0.927986	-0.171814
15	6	0	1.412376	1.437207	0.110334
16	6	0	3.244749	-0.561325	-0.587916
17	6	0	2.703711	1.758726	-0.316810
18	6	0	3.617232	0.774315	-0.674385
19	1	0	3.955540	-1.337054	-0.836126
20	1	0	2.994586	2.798894	-0.354051
21	1	0	-2.824573	-0.826939	-2.105052
22	1	0	-1.962117	1.643932	1.113439
23	8	0	-0.575716	-1.170371	2.027920
24	6	0	0.514421	2.575404	0.518322
25	1	0	-0.315759	2.704049	-0.170352
26	1	0	0.103649	2.426408	1.512849
27	1	0	1.083743	3.499565	0.533173
28	6	0	1.645234	-2.400490	-0.098546
29	1	0	0.972919	-2.632439	0.721170
30	1	0	1.191678	-2.752913	-1.022251
31	1	0	2.561236	-2.964453	0.049126
32	1	0	4.611313	1.044705	-0.998118

11f:

1303baa_POcpent3Me_odiMe_ikerion_MP2_6311++2d2p_PCMthf.log

Input orientation:

Center Number	Atomic Number	Atomic Type	Coordinates (Angstroms) X	Y	Z
1	15	0	0.607160	-0.785648	0.044639
2	6	0	1.819449	-0.296084	-1.015360
3	6	0	1.477563	-0.355445	1.596667
4	1	0	0.877979	0.314247	2.209463
5	1	0	1.604451	-1.282599	2.157003
6	6	0	2.779507	0.230219	1.117549
7	1	0	3.551434	0.543211	1.803067
8	6	0	2.924964	0.229850	-0.234454
9	6	0	4.141959	0.751584	-0.933894
10	1	0	4.877085	1.115801	-0.221915
11	1	0	4.601830	-0.024888	-1.542137
12	1	0	3.876603	1.567350	-1.604478
13	6	0	-1.053727	-0.046004	-0.026986
14	6	0	-2.277793	-0.748634	-0.153585
15	6	0	-1.054737	1.369602	0.060648
16	6	0	-3.466937	-0.012227	-0.156547
17	6	0	-2.274099	2.050513	0.064413
18	6	0	-3.479337	1.371431	-0.039191
19	1	0	-4.401703	-0.546172	-0.248328
20	1	0	-2.266531	3.128301	0.143144
21	8	0	0.253503	-2.379498	0.063307
22	1	0	1.053750	-2.852818	-0.201875
23	1	0	1.741753	-0.212800	-2.085424
24	1	0	-4.414941	1.910655	-0.035891
25	6	0	-2.426600	-2.243825	-0.292685
26	1	0	-1.883401	-2.628042	-1.148311
27	1	0	-2.060987	-2.771050	0.581306
28	1	0	-3.478242	-2.479984	-0.420662
29	6	0	0.193005	2.216286	0.090028
30	1	0	0.994784	1.827026	0.710397
31	1	0	0.605151	2.310587	-0.912349
32	1	0	-0.059302	3.209764	0.450680

11f + H2:

1303bba_POcpent3Me_odiMe_ikerion_+H2OL_MP2_6311++2d2p_PCMthf.log

Input orientation:

Center Number	Atomic Number	Atomic Type	Coordinates (Angstroms) X	Y	Z
1	15	0	0.649017	-0.316846	-0.535112
2	6	0	1.832081	0.855203	-0.387257
3	6	0	1.471449	-1.598273	0.472663
4	1	0	0.821839	-2.008104	1.234761
5	1	0	1.754245	-2.404260	-0.201838
6	6	0	2.689626	-0.879779	1.058377
7	1	0	3.493455	-1.583753	1.267721
8	6	0	3.126806	0.220208	0.085827
9	6	0	4.036534	1.238568	0.757000
10	1	0	4.935947	0.763862	1.146122
11	1	0	4.340800	2.014107	0.058219
12	1	0	3.510344	1.714253	1.582706
13	6	0	-1.072875	0.094827	-0.113798
14	6	0	-1.958413	-0.943871	0.259501
15	6	0	-1.527981	1.433085	-0.139433
16	6	0	-3.257037	-0.614210	0.655425
17	6	0	-2.838054	1.710043	0.256918
18	6	0	-3.700705	0.701709	0.669111
19	1	0	-3.928959	-1.410735	0.943295
20	1	0	-3.181972	2.734257	0.229345
21	1	0	2.404056	-0.399246	1.992724
22	1	0	3.690829	-0.246377	-0.730219
23	8	0	0.399097	-1.212776	-1.931239
24	1	0	-0.129025	-0.710167	-2.562581
25	1	0	1.830524	1.823180	-0.853925
26	6	0	-1.598914	-2.409140	0.260472
27	1	0	-0.857689	-2.657207	-0.489506
28	1	0	-1.222493	-2.721827	1.231785
29	1	0	-2.489899	-2.996376	0.057660
30	6	0	-0.679578	2.584800	-0.598146
31	1	0	0.111872	2.797511	0.115096
32	1	0	-0.206523	2.382874	-1.556048
33	1	0	-1.295617	3.471426	-0.711010
34	1	0	-4.709351	0.935915	0.975359

11f:

1303bba_POcpent3Me_odiMe_ikerion_MP2_6311++2d2p_PCMthf.log

Input orientation:

Center Number	Atomic Number	Atomic Type	Coordinates (Angstroms) X	Y	Z
1	15	0	0.675716	0.234888	0.474798
2	6	0	1.889306	-0.905821	0.216636
3	6	0	1.427982	1.546858	-0.556909
4	1	0	0.844811	1.633515	-1.474177
5	1	0	1.388647	2.504504	-0.043998
6	6	0	2.816167	1.038540	-0.824267
7	1	0	3.555904	1.639755	-1.328649
8	6	0	3.016593	-0.240629	-0.424977
9	6	0	4.297179	-0.990334	-0.624154
10	1	0	5.037478	-0.372982	-1.124677
11	1	0	4.705853	-1.317847	0.329883
12	1	0	4.129459	-1.883177	-1.223559
13	6	0	-1.063428	-0.083344	0.080980
14	6	0	-1.926618	0.996211	-0.207399
15	6	0	-1.546536	-1.409095	0.055956
16	6	0	-3.244254	0.721060	-0.579609
17	6	0	-2.872114	-1.634555	-0.319249
18	6	0	-3.718119	-0.583224	-0.652702
19	1	0	-3.906117	1.546978	-0.798871
20	1	0	-3.242132	-2.649799	-0.335911
21	8	0	0.427494	0.788983	2.011591
22	1	0	1.276004	0.845488	2.471981
23	1	0	1.933167	-1.913578	0.582819

24	1	0	-4.740046	-0.776499	-0.942963
25	6	0	-1.511047	2.440852	-0.112582
26	1	0	-0.846184	2.617957	0.726647
27	1	0	-1.011212	2.776099	-1.017911
28	1	0	-2.390515	3.062773	0.022539
29	6	0	-0.705329	-2.592960	0.442981
30	1	0	0.044756	-2.809079	-0.312658
31	1	0	-0.184791	-2.426137	1.383072
32	1	0	-1.336643	-3.467650	0.563953

11f + H2:

1303bbb_POcpent3Me_odiMe_ikerion_+H2OL_MP2_6311++2d2p_PCMthf.log

Input orientation:

Center Number	Atomic Number	Atomic Type	Coordinates (Angstroms) X	Y	Z
1	15	0	-0.649048	-0.316609	0.535107
2	6	0	-1.832155	0.855394	0.387130
3	6	0	-1.471395	-1.598128	-0.472615
4	1	0	-0.821780	-2.007925	-1.234732
5	1	0	-1.754102	-2.404147	0.201891
6	6	0	-2.689667	-0.879792	-1.058323
7	1	0	-3.493440	-1.583857	-1.267577
8	6	0	-3.126858	0.220214	-0.085806
9	6	0	-4.036823	1.238394	-0.756939
10	1	0	-4.936187	0.763516	-1.145969
11	1	0	-4.341162	2.013903	-0.058156
12	1	0	-3.510778	1.714136	-1.582706
13	6	0	1.072884	0.094923	0.113793
14	6	0	1.958314	-0.943929	-0.259509
15	6	0	1.528185	1.433158	0.139364
16	6	0	3.256968	-0.614463	-0.655517
17	6	0	2.838281	1.709921	-0.257005
18	6	0	3.700777	0.701430	-0.669223
19	1	0	3.928790	-1.411073	-0.943388
20	1	0	3.182348	2.734087	-0.229436
21	1	0	-2.404179	-0.399290	-1.992713
22	1	0	-3.690728	-0.246402	0.730332
23	8	0	-0.399163	-1.212385	1.931333
24	1	0	0.128668	-0.709599	2.562776
25	1	0	-1.830646	1.823404	0.853741
26	6	0	1.598674	-2.409150	-0.260376
27	1	0	0.857295	-2.657067	0.489498
28	1	0	1.222426	-2.721911	-1.231732
29	1	0	2.489571	-2.996439	-0.057307
30	6	0	0.679861	2.584930	0.598116
31	1	0	-0.111536	2.797762	-0.115153
32	1	0	0.206739	2.382959	1.555979
33	1	0	1.295963	3.471502	0.711090
34	1	0	4.709448	0.935497	-0.975497

1f+H+:

1305baa_POcpent3Me_odiMe_+H+_MP2_6311++2d2p_PCMthf.log

Input orientation:

Center Number	Atomic Number	Atomic Type	Coordinates (Angstroms) X	Y	Z
1	15	0	-0.618342	-0.217244	0.392374
2	6	0	-1.775831	1.021613	-0.232487
3	1	0	-1.403041	1.446982	-1.163550
4	1	0	-1.917673	1.827529	0.481236
5	6	0	-1.431992	-1.643074	-0.369471
6	1	0	-0.969926	-1.865937	-1.329642
7	1	0	-1.353551	-2.521267	0.262376
8	6	0	-2.839077	-1.127592	-0.523501
9	1	0	-3.647663	-1.819600	-0.703655
10	6	0	-3.025216	0.199313	-0.459807
11	6	0	-4.328523	0.904542	-0.624413
12	1	0	-5.130257	0.199233	-0.815071
13	1	0	-4.569344	1.476261	0.269042
14	1	0	-4.276465	1.607142	-1.453033
15	6	0	1.111858	0.029516	0.057365
16	6	0	1.946089	-1.093863	-0.139528
17	6	0	1.622492	1.346383	0.010203
18	6	0	3.282679	-0.864972	-0.464363
19	6	0	2.967199	1.513665	-0.317870
20	6	0	3.789991	0.423839	-0.574553
21	1	0	3.932581	-1.714072	-0.615370
22	1	0	3.371249	2.514448	-0.354879
23	8	0	-0.725012	-0.310518	1.983402
24	1	0	-1.621619	-0.443086	2.323452
25	1	0	4.827459	0.577168	-0.829621
26	6	0	0.812733	2.573111	0.333972
27	1	0	0.191505	2.883752	-0.501263
28	1	0	0.178021	2.427112	1.203194
29	1	0	1.483888	3.394334	0.560803
30	6	0	1.487899	-2.518719	0.021350
31	1	0	0.841565	-2.647900	0.884840
32	1	0	0.965605	-2.880903	-0.859831
33	1	0	2.352146	-3.157109	0.169476

1f+H+:

1305bab_POcpent3Me_odiMe_+H+_MP2_6311++2d2p_PCMthf.log

Input orientation:

Center Number	Atomic Number	Atomic Type	Coordinates (Angstroms) X	Y	Z
1	15	0	-0.576889	0.673993	0.205178
2	6	0	-1.727333	0.191965	-1.104934
3	1	0	-1.304440	-0.575887	-1.749256
4	1	0	-1.936286	1.061068	-1.727367
5	6	0	-1.506720	0.037707	1.622369
6	1	0	-0.992864	-0.790933	2.100689
7	1	0	-1.597486	0.833855	2.359225
8	6	0	-2.817273	-0.353726	0.995618
9	1	0	-3.628868	-0.688382	1.624388
10	6	0	-2.940306	-0.284522	-0.340916
11	6	0	-4.165009	-0.650700	-1.109828
12	1	0	-4.957792	-0.980796	-0.447011
13	1	0	-4.521488	0.199459	-1.686942
14	1	0	-3.943445	-1.448626	-1.815503
15	6	0	1.090729	0.066102	0.022739
16	6	0	1.212057	-1.342935	0.022342
17	6	0	2.219984	0.905298	-0.134861
18	6	0	2.481852	-1.899597	-0.128801
19	6	0	3.462841	0.285021	-0.273642
20	6	0	3.604047	-1.097392	-0.270832
21	1	0	2.579366	-2.975466	-0.135749
22	1	0	4.335267	0.909508	-0.394331
23	8	0	-0.504851	2.260966	0.298716
24	1	0	-1.363863	2.684650	0.430533

25	6	0	2.196687	2.411732	-0.175709
26	1	0	1.565049	2.787182	-0.973556
27	1	0	1.838286	2.837466	0.754953
28	1	0	3.205229	2.771401	-0.346507
29	6	0	0.065402	-2.306797	0.203719
30	1	0	0.017436	-2.650478	1.235014
31	1	0	-0.911909	-1.912246	-0.052153
32	1	0	0.226736	-3.178755	-0.422282
33	1	0	4.580537	-1.542580	-0.385293

1f-H+:

1305bbb_POcpent3Me_odiMe_-H+A_MP2_6311++2d2p_PCMthf_FRQ.log

Input orientation:

Center Number	Atomic Number	Atomic Type	Coordinates (Angstroms) X	Y	Z
1	15	0	-0.702288	-0.240450	0.665134
2	6	0	-1.823967	0.939900	0.068817
3	6	0	-1.424688	-1.585120	-0.391732
4	1	0	-0.801962	-1.668146	-1.285974
5	1	0	-1.393648	-2.538571	0.130718
6	6	0	-2.802439	-1.091380	-0.722978
7	1	0	-3.564293	-1.717866	-1.163442
8	6	0	-2.955265	0.244850	-0.486622
9	6	0	-4.222211	0.986961	-0.801493
10	1	0	-4.973089	0.322646	-1.222663
11	1	0	-4.635591	1.448990	0.093922
12	1	0	-4.035246	1.787939	-1.515390
13	6	0	1.028923	0.043690	0.105405
14	6	0	1.886528	-1.046721	-0.166845
15	6	0	1.548765	1.355065	0.024664
16	6	0	3.193113	-0.808220	-0.602969
17	6	0	2.862242	1.555618	-0.407970
18	6	0	3.683660	0.485032	-0.744517
19	1	0	3.836303	-1.652817	-0.811284
20	1	0	3.244100	2.566025	-0.466073
21	8	0	-0.606606	-0.572908	2.153409
22	1	0	-1.911172	1.953484	0.422919
23	1	0	4.696002	0.654210	-1.081769
24	6	0	1.474799	-2.482976	0.030424
25	1	0	0.820543	-2.593799	0.889277
26	1	0	0.954221	-2.879727	-0.837730
27	1	0	2.356787	-3.096359	0.193327
28	6	0	0.746353	2.565579	0.413508
29	1	0	0.017621	2.814107	-0.353273
30	1	0	0.194883	2.400491	1.335661
31	1	0	1.406834	3.415732	0.561336

1f+H+_+H2:

1306ca_POcpent3Me_odiMe_+H+_+H2OL_MP2_6311++2d2p_PCMthf_FRQ.log

Input orientation:

Center Number	Atomic Number	Atomic Type	Coordinates (Angstroms) X	Y	Z
1	15	0	-0.582458	-0.228203	0.513120
2	6	0	-1.728304	1.153725	0.340284
3	6	0	-1.530497	-1.409528	-0.493281
4	1	0	-0.933550	-1.790566	-1.312063
5	1	0	-1.797254	-2.246326	0.148567
6	6	0	-2.765514	-0.621258	-0.958042
7	1	0	-3.608300	-1.287285	-1.111747
8	6	0	-3.073314	0.454425	0.083131
9	6	0	-4.136935	1.439258	-0.368762
10	1	0	-5.072492	0.923790	-0.566150
11	1	0	-4.319185	2.196708	0.387670
12	1	0	-3.821461	1.936895	-1.283131
13	6	0	1.117343	-0.020590	0.019419
14	6	0	1.852671	-1.193878	-0.267379
15	6	0	1.705371	1.260388	-0.076458

16	6	0	3.163360	-1.051882	-0.721441
17	6	0	3.018238	1.340086	-0.541028
18	6	0	3.739797	0.201869	-0.879615
19	1	0	3.737468	-1.940671	-0.938787
20	1	0	3.481735	2.312418	-0.617853
21	1	0	-2.548593	-0.133664	-1.906978
22	1	0	-3.407931	-0.029773	1.002456
23	8	0	-0.483536	-0.693911	2.041762
24	1	0	-1.711754	1.807668	1.204772
25	6	0	1.320827	-2.592111	-0.084299
26	1	0	0.587692	-2.668030	0.712176
27	1	0	0.877494	-2.974667	-1.000186
28	1	0	2.141851	-3.252464	0.175380
29	6	0	1.024137	2.541094	0.325856
30	1	0	0.343038	2.900377	-0.440586
31	1	0	0.474673	2.440427	1.256289
32	1	0	1.774261	3.310044	0.476054
33	1	0	4.754585	0.290609	-1.236385
34	1	0	-1.452109	1.723059	-0.545498
35	1	0	-1.324806	-0.880750	2.479736

11f:

1309ba_POcpent3Me_odiMePh_ikerion_TS1_HO_MP2_631++dp_PCMthf.log

Input orientation:

Center Number	Atomic Number	Atomic Type	Coordinates (Angstroms) X	Y	Z
1	15	0	-0.567496	-0.683975	0.030041
2	6	0	-1.876550	-0.086573	1.040577
3	6	0	-1.372275	-0.471562	-1.593441
4	1	0	-0.804744	0.219986	-2.220516
5	1	0	-1.393663	-1.438344	-2.104422
6	6	0	-2.742575	0.033968	-1.213924
7	1	0	-3.513370	0.146290	-1.966113
8	6	0	-2.963653	0.265083	0.109465
9	6	0	-4.270484	0.755089	0.656674
10	1	0	-4.986044	0.944018	-0.142303
11	1	0	-4.696598	0.021576	1.342225
12	1	0	-4.126709	1.679111	1.219237
13	6	0	1.103014	-0.003443	0.041737
14	6	0	2.249022	-0.833920	0.047933
15	6	0	1.219387	1.408077	0.008482
16	6	0	3.506832	-0.218448	0.071421
17	6	0	2.500668	1.971141	0.026500
18	6	0	3.638796	1.169103	0.069766
19	1	0	4.392921	-0.841113	0.077325
20	1	0	2.600958	3.049401	0.003430
21	8	0	-0.662756	-2.178089	0.608780
22	1	0	-1.559730	-1.625550	1.214787
23	1	0	-1.700793	0.536192	1.911211
24	6	0	2.188237	-2.340263	0.001847
25	1	0	1.806517	-2.757651	0.930741
26	1	0	1.538172	-2.692138	-0.797238
27	1	0	3.187045	-2.735453	-0.173867
28	6	0	0.029517	2.337002	-0.019946
29	1	0	-0.767571	1.993806	-0.677975
30	1	0	-0.403921	2.457948	0.973264
31	1	0	0.346118	3.318131	-0.370374
32	1	0	4.622115	1.621073	0.085717

1d:
1201baa_POcpent3Me_pCF3Ph_MP2_631++2d2p_PCMthf.log

Input orientation:

Center Number	Atomic Number	Atomic Type	Coordinates (Angstroms) X	Y	Z
1	15	0	-2.005788	-0.619424	0.453358
2	6	0	-2.821157	1.011119	0.527881
3	1	0	-2.153236	1.810014	0.208607
4	1	0	-3.144976	1.223759	1.544561
5	6	0	-2.702203	-1.086650	-1.170245
6	1	0	-1.993489	-0.902194	-1.975614
7	1	0	-2.953752	-2.144235	-1.172334
8	6	0	-3.906462	-0.190240	-1.267658
9	1	0	-4.676807	-0.384626	-2.000574
10	6	0	-3.978009	0.856076	-0.428735
11	6	0	-5.087444	1.854686	-0.396649
12	1	0	-5.840482	1.631304	-1.146034
13	1	0	-5.562456	1.865210	0.582331
14	1	0	-4.704718	2.857459	-0.576941
15	6	0	-0.227110	-0.353008	0.255422
16	6	0	0.530110	-1.211259	-0.547229
17	6	0	0.415866	0.665313	0.966795
18	6	0	1.907720	-1.049905	-0.650966
19	1	0	0.052654	-2.010476	-1.094964
20	6	0	1.792117	0.833177	0.868873
21	1	0	-0.151003	1.331432	1.600632
22	6	0	2.532369	-0.028653	0.060991
23	1	0	2.487216	-1.714451	-1.271876
24	1	0	2.283355	1.620294	1.419763
25	8	0	-2.343934	-1.551395	1.582192
26	6	0	4.003148	0.192284	-0.077832
27	9	0	4.650749	-0.911743	-0.485786
28	9	0	4.565982	0.581439	1.080862
29	9	0	4.273008	1.160310	-0.981498

1d:
1201bab_POcpent3Me_pCF3Ph_MP2_631++2d2p_PCMthf.log

Input orientation:

Center Number	Atomic Number	Atomic Type	Coordinates (Angstroms) X	Y	Z
1	15	0	-2.137162	1.188392	-0.067791
2	6	0	-3.004962	-0.024190	-1.121336
3	1	0	-2.351981	-0.413235	-1.900003
4	1	0	-3.849114	0.463674	-1.608767
5	6	0	-2.845474	0.590649	1.508741
6	1	0	-2.092577	0.569576	2.292557
7	1	0	-3.634991	1.273865	1.819834
8	6	0	-3.374695	-0.768629	1.149961
9	1	0	-3.692082	-1.457421	1.920752
10	6	0	-3.460437	-1.085985	-0.153594
11	6	0	-3.979720	-2.381012	-0.683986
12	1	0	-4.289278	-3.041125	0.120575
13	1	0	-4.829821	-2.210718	-1.341884
14	1	0	-3.216249	-2.884672	-1.274792
15	6	0	-0.409547	0.638991	-0.048362
16	6	0	0.587279	1.611328	-0.143616
17	6	0	-0.051652	-0.709798	0.066699
18	6	0	1.931808	1.249225	-0.120505
19	1	0	0.304830	2.648567	-0.240836
20	6	0	1.287644	-1.076625	0.090870
21	1	0	-0.811759	-1.473687	0.140462
22	6	0	2.274367	-0.093871	-0.001319
23	1	0	2.699723	2.002297	-0.195245
24	1	0	1.563227	-2.116898	0.179271
25	8	0	-2.265192	2.648349	-0.391014
26	6	0	3.702337	-0.528854	0.024784
27	9	0	3.991767	-1.211512	1.152535
28	9	0	3.987358	-1.356626	-1.002728

29	9	0	4.560853	0.499899	-0.046479

1d+H2:

1201bba_POcpent3Me_pCF3Ph_+H2OL_MP2_631++2d2p_PCMthf.log

Input orientation:

Center Number	Atomic Number	Atomic Type	Coordinates (Angstroms) X	Y	Z
1	15	0	2.031080	-0.945245	-0.589102
2	6	0	2.926550	0.617432	-0.924616
3	1	0	2.236612	1.457119	-0.880840
4	1	0	3.371746	0.589794	-1.914451
5	6	0	2.756662	-1.210779	1.062700
6	1	0	2.061413	-1.642535	1.773040
7	1	0	3.580250	-1.909433	0.923559
8	6	0	3.287875	0.167998	1.455619
9	1	0	3.979355	0.109210	2.292373
10	6	0	3.957823	0.747545	0.207082
11	6	0	4.438892	2.175389	0.392786
12	1	0	5.169581	2.238215	1.195528
13	1	0	4.898954	2.558001	-0.514409
14	1	0	3.601049	2.822828	0.645194
15	6	0	0.294685	-0.490188	-0.308821
16	6	0	-0.481809	-1.207781	0.607602
17	6	0	-0.311968	0.513001	-1.071681
18	6	0	-1.830509	-0.916063	0.776901
19	1	0	-0.044477	-2.002809	1.192335
20	6	0	-1.661329	0.809329	-0.912420
21	1	0	0.259714	1.067884	-1.800465
22	6	0	-2.415271	0.092714	0.013959
23	1	0	-2.420439	-1.473229	1.487994
24	1	0	-2.120386	1.583808	-1.506377
25	1	0	2.463744	0.817960	1.755404
26	1	0	4.810024	0.109973	-0.033458
27	8	0	2.185020	-2.057025	-1.589881
28	6	0	-3.849782	0.442489	0.238480
29	9	0	-4.589624	-0.634278	0.558714
30	9	0	-3.988977	1.323052	1.254553
31	9	0	-4.413091	1.007180	-0.843221

1d+H2:

1202ba_POcpent2Me_pCF3Ph_MP2_631++2d2p_PCMthf.log

Input orientation:

Center Number	Atomic Number	Atomic Type	Coordinates (Angstroms) X	Y	Z
1	15	0	-2.099569	1.215940	-0.153799
2	6	0	-3.000370	-0.017539	-1.079341
3	6	0	-2.846474	0.730414	1.437513
4	1	0	-2.130666	0.772269	2.251017
5	1	0	-3.627391	1.463174	1.627775
6	6	0	-3.451314	-0.661355	1.215288
7	1	0	-4.402632	-0.775740	1.730792
8	6	0	-3.625826	-0.887881	-0.267813
9	6	0	-4.424554	-2.064058	-0.718985
10	1	0	-5.445363	-1.983599	-0.350020
11	1	0	-4.446067	-2.147399	-1.800040
12	1	0	-4.009665	-2.980572	-0.303152
13	6	0	-0.376750	0.646830	-0.117214
14	6	0	0.647144	1.595918	-0.114490
15	6	0	-0.057401	-0.713817	-0.056519
16	6	0	1.979380	1.196535	-0.047696
17	1	0	0.395920	2.644021	-0.173103
18	6	0	1.269128	-1.119983	0.013089
19	1	0	-0.840568	-1.457913	-0.080499
20	6	0	2.282331	-0.160371	0.019075
21	1	0	2.769071	1.930583	-0.049167
22	1	0	1.515045	-2.170524	0.056204
23	1	0	-2.793673	-1.436606	1.609700

24	1	0	-3.007910	-0.091144	-2.155633
25	8	0	-2.191089	2.662326	-0.547526
26	6	0	3.696159	-0.634684	0.089335
27	9	0	4.583223	0.372104	0.097055
28	9	0	3.917797	-1.368586	1.200500
29	9	0	4.005570	-1.429346	-0.956959

11d:

1203baa_POcpent3Me_pCF3Ph_ikerion_MP2_631++2d2p_PCMthf.log

Input orientation:

Center Number	Atomic Number	Atomic Type	Coordinates (Angstroms) X	Y	Z
1	15	0	-2.020639	0.702906	-0.153972
2	6	0	-2.985154	-0.404569	-0.960476
3	6	0	-2.764335	0.483002	1.498791
4	1	0	-2.031858	0.004182	2.148247
5	1	0	-3.003048	1.454201	1.928839
6	6	0	-3.962318	-0.388569	1.228010
7	1	0	-4.683053	-0.621906	1.995462
8	6	0	-4.041598	-0.837617	-0.048781
9	6	0	-5.127261	-1.736710	-0.551913
10	1	0	-5.825618	-1.988247	0.240920
11	1	0	-5.676735	-1.261486	-1.362226
12	1	0	-4.707846	-2.659339	-0.948729
13	6	0	-0.231156	0.459794	-0.124201
14	6	0	0.688903	1.508374	-0.207230
15	6	0	0.228693	-0.853900	0.022331
16	6	0	2.055261	1.248350	-0.143166
17	1	0	0.342299	2.521725	-0.325465
18	6	0	1.590272	-1.117440	0.089368
19	1	0	-0.482384	-1.665702	0.072013
20	6	0	2.498935	-0.062166	0.006839
21	1	0	2.763929	2.058039	-0.209979
22	1	0	1.942314	-2.132055	0.199944
23	8	0	-2.006658	2.286024	-0.589046
24	1	0	-2.893813	2.551134	-0.862434
25	1	0	-2.899672	-0.716452	-1.986427
26	6	0	3.955933	-0.382607	0.074972
27	9	0	4.267869	-1.027764	1.218276
28	9	0	4.331996	-1.195413	-0.934432
29	9	0	4.731816	0.710583	0.012780

11d+H2:

1203bba_POcpent3Me_pCF3Ph_ikerion_+H2OL_MP2_631++2d2p_PCMthf.log

Input orientation:

Center Number	Atomic Number	Atomic Type	Coordinates (Angstroms) X	Y	Z
1	15	0	-1.991707	0.719429	-0.298807
2	6	0	-2.868426	-0.542994	-0.931260
3	6	0	-2.912982	0.925454	1.254604
4	1	0	-2.255573	1.003761	2.114630
5	1	0	-3.481411	1.849204	1.172471
6	6	0	-3.822319	-0.308269	1.282025
7	1	0	-4.715557	-0.122240	1.876198
8	6	0	-4.167670	-0.706992	-0.163054
9	6	0	-4.687630	-2.135136	-0.236650
10	1	0	-5.580579	-2.258602	0.374007
11	1	0	-4.938035	-2.409486	-1.258472
12	1	0	-3.925235	-2.824427	0.121937
13	6	0	-0.206033	0.431010	-0.192177
14	6	0	0.701119	1.477204	0.016043
15	6	0	0.269526	-0.881776	-0.274451
16	6	0	2.061192	1.215569	0.139744
17	1	0	0.345664	2.493487	0.080477
18	6	0	1.630459	-1.147259	-0.171946
19	1	0	-0.436867	-1.685263	-0.418734
20	6	0	2.520551	-0.097142	0.046946

21	1	0	2.754887	2.023940	0.310315
22	1	0	1.992349	-2.161139	-0.244140
23	1	0	-3.281323	-1.140124	1.730545
24	1	0	-4.962217	-0.043516	-0.521720
25	8	0	-2.002826	2.303455	-0.817705
26	1	0	-1.595849	2.383684	-1.688228
27	1	0	-2.678902	-1.032807	-1.873082
28	6	0	3.985785	-0.375677	0.120173
29	9	0	4.247186	-1.589553	0.637411
30	9	0	4.639536	0.527377	0.872508
31	9	0	4.556610	-0.345862	-1.104749

1d+H+:

1205bab_POcpent3Me_pCF3Ph_+H+_MP2_6311++2d2p_PCMthf_FRQ.log

Input orientation:

Center Number	Atomic Number	Atomic Type	Coordinates (Angstroms) X	Y	Z
1	15	0	-1.923139	0.629897	0.106195
2	6	0	-2.876026	-0.335497	-1.077457
3	1	0	-2.301427	-1.197234	-1.415007
4	1	0	-3.112429	0.267850	-1.951361
5	6	0	-2.729957	0.046933	1.608401
6	1	0	-2.102529	-0.686833	2.111683
7	1	0	-2.882215	0.878529	2.292210
8	6	0	-4.002421	-0.544730	1.058723
9	1	0	-4.802347	-0.812394	1.732108
10	6	0	-4.089254	-0.739140	-0.267425
11	6	0	-5.257454	-1.333024	-0.978973
12	1	0	-6.044174	-1.599396	-0.281360
13	1	0	-5.657458	-0.630895	-1.706980
14	1	0	-4.955992	-2.224796	-1.523713
15	6	0	-0.164439	0.394940	0.056514
16	6	0	0.342036	-0.878536	0.343442
17	6	0	0.689521	1.448828	-0.274231
18	6	0	1.711464	-1.093434	0.295808
19	1	0	-0.312969	-1.698301	0.602243
20	6	0	2.062184	1.226930	-0.318402
21	1	0	0.293932	2.427314	-0.493136
22	6	0	2.563332	-0.039409	-0.035498
23	1	0	2.111877	-2.071221	0.514887
24	1	0	2.729927	2.033971	-0.571713
25	8	0	-2.104284	2.191497	-0.116293
26	1	0	-3.016378	2.507938	-0.164638
27	6	0	4.035474	-0.312368	-0.065124
28	9	0	4.324738	-1.327953	-0.899977
29	9	0	4.746973	0.750774	-0.461518
30	9	0	4.486820	-0.669923	1.152332

1d-H+:
1205bba_POcpent3Me_pCF3Ph_-H+A_MP2_6311++2d2p_PCMthf.log

Input orientation:

Center Number	Atomic Number	Atomic Type	Coordinates (Angstroms) X	Y	Z
1	15	0	2.206048	-1.165405	-0.291925
2	6	0	3.099763	0.055981	-1.125477
3	6	0	2.822379	-0.689481	1.377894
4	1	0	2.036580	-0.788117	2.123695
5	1	0	3.616336	-1.401146	1.630743
6	6	0	3.353017	0.705576	1.170625
7	1	0	3.763523	1.301585	1.973610
8	6	0	3.519337	1.024951	-0.152081
9	6	0	4.128557	2.325435	-0.589785
10	1	0	4.391926	2.943959	0.264838
11	1	0	5.026161	2.152098	-1.181402
12	1	0	3.437471	2.885863	-1.218622
13	6	0	0.433930	-0.673205	-0.197723
14	6	0	-0.564168	-1.608456	0.086724
15	6	0	0.072961	0.666548	-0.374498
16	6	0	-1.900304	-1.224771	0.183746
17	1	0	-0.291383	-2.645424	0.215598
18	6	0	-1.255635	1.065864	-0.274061
19	1	0	0.847923	1.384997	-0.603845
20	6	0	-2.238618	0.115016	0.005872
21	1	0	-2.666790	-1.954866	0.391358
22	1	0	-1.528351	2.102057	-0.413362
23	8	0	2.230098	-2.641079	-0.651708
24	1	0	2.983683	0.298961	-2.170775
25	6	0	-3.653940	0.571200	0.088937
26	9	0	-4.504908	-0.412917	0.427987
27	9	0	-4.090997	1.077249	-1.087314
28	9	0	-3.813347	1.558150	0.999314

1d-H+:
1205bbb_POcpent3Me_pCF3Ph_-H+A_MP2_6311++2d2p_PCMthf.log

Input orientation:

Center Number	Atomic Number	Atomic Type	Coordinates (Angstroms) X	Y	Z
1	15	0	-2.206178	1.166908	-0.286421
2	6	0	-3.101621	-0.052523	-1.121003
3	6	0	-2.819993	0.687745	1.383399
4	1	0	-2.033090	0.784959	2.128223
5	1	0	-3.613654	1.398819	1.638812
6	6	0	-3.350777	-0.706970	1.174138
7	1	0	-3.759733	-1.304736	1.976608
8	6	0	-3.519555	-1.023543	-0.148928
9	6	0	-4.129383	-2.323195	-0.588281
10	1	0	-4.391557	-2.943364	0.265519
11	1	0	-5.027800	-2.148766	-1.178345
12	1	0	-3.439138	-2.882408	-1.219130
13	6	0	-0.434108	0.673827	-0.196172
14	6	0	0.564995	1.607910	0.088584
15	6	0	-0.074211	-0.665721	-0.376526
16	6	0	1.901086	1.223231	0.182363
17	1	0	0.293060	2.644757	0.220238
18	6	0	1.254326	-1.066001	-0.279334
19	1	0	-0.850008	-1.383203	-0.606065
20	6	0	2.238340	-0.116318	0.000929
21	1	0	2.668338	1.952464	0.390195
22	1	0	1.526229	-2.102027	-0.421440
23	8	0	-2.230318	2.643296	-0.643234
24	1	0	-2.987383	-0.293320	-2.167011
25	6	0	3.653575	-0.573372	0.080475
26	9	0	3.814131	-1.562408	0.988366
27	9	0	4.088329	-1.076982	-1.097681
28	9	0	4.505606	0.409592	0.420208

1d+H+ + H2:

1206ca_POcpent3Me_pCF3Ph_+H+_+H2OL_MP2_6311++2d2p_PCMthf_JO.log

Input orientation:

Center Number	Atomic Number	Atomic Type	Coordinates (Angstroms) X	Y	Z
1	15	0	-2.017088	0.780109	-0.233680
2	6	0	-1.260000	-0.297857	-1.462288
3	6	0	-3.585571	-0.103345	-0.068035
4	1	0	-3.872094	-0.215013	0.971666
5	1	0	-4.341998	0.500585	-0.567186
6	6	0	-3.341181	-1.418962	-0.823226
7	1	0	-4.282721	-1.860752	-1.133179
8	6	0	-2.438520	-1.113092	-2.023459
9	6	0	-1.968626	-2.362202	-2.746955
10	1	0	-2.816910	-2.924267	-3.126836
11	1	0	-1.324588	-2.111467	-3.584344
12	1	0	-1.411232	-3.003200	-2.067372
13	6	0	-1.093704	0.909495	1.279900
14	6	0	-0.731483	2.153625	1.801913
15	6	0	-0.754128	-0.272657	1.946520
16	6	0	-0.024816	2.211133	2.997161
17	1	0	-0.993904	3.062554	1.285565
18	6	0	-0.051521	-0.209473	3.142709
19	1	0	-1.030464	-1.239391	1.550316
20	6	0	0.313905	1.032450	3.658110
21	1	0	0.264335	3.167137	3.403997
22	1	0	0.215403	-1.116658	3.660279
23	1	0	-2.840975	-2.136056	-0.172940
24	1	0	-2.994412	-0.483475	-2.720244
25	8	0	-2.178485	2.292741	-0.695390
26	1	0	-0.699340	0.272054	-2.196398
27	1	0	-2.690838	2.432827	-1.502305
28	6	0	1.028653	1.111763	4.971969
29	9	0	0.157444	1.283936	5.986397
30	9	0	1.725220	-0.004610	5.234779
31	9	0	1.886837	2.143752	5.012162
32	1	0	-0.564838	-0.952856	-0.936219

TS(1d->11d):

1209ba_POcpent3Me_pCF3Ph_ikerion_TS_HO_MP2_6311++2d2p_PCMthf.log

Input orientation:

Center Number	Atomic Number	Atomic Type	Coordinates (Angstroms) X	Y	Z
1	15	0	1.945977	0.589621	-0.210224
2	6	0	3.048168	0.113382	1.062258
3	6	0	2.712104	-0.315404	-1.581632
4	1	0	2.063034	-1.125643	-1.911949
5	1	0	2.860043	0.358628	-2.423777
6	6	0	3.993834	-0.802753	-0.955814
7	1	0	4.771229	-1.240893	-1.563976
8	6	0	4.119992	-0.633449	0.382982
9	6	0	5.310458	-1.089538	1.166789
10	1	0	6.030333	-1.596267	0.530936
11	1	0	5.800584	-0.244462	1.645390
12	1	0	5.005606	-1.772624	1.957263
13	6	0	0.173275	0.344719	-0.133057
14	6	0	-0.669324	1.437971	0.082357
15	6	0	-0.366222	-0.941721	-0.256895
16	6	0	-2.045896	1.251471	0.168058
17	1	0	-0.247395	2.426486	0.175590
18	6	0	-1.738678	-1.128524	-0.174225
19	1	0	0.276897	-1.796520	-0.406334
20	6	0	-2.573004	-0.029614	0.037192
21	1	0	-2.697903	2.094421	0.330485
22	1	0	-2.156615	-2.119296	-0.269525
23	8	0	2.297627	2.128981	-0.148537
24	1	0	3.037847	1.663643	0.719993
25	1	0	2.741380	-0.154362	2.063233

26	6	0	-4.044686	-0.272375	0.120771
27	9	0	-4.515794	-0.842461	-1.007499
28	9	0	-4.745292	0.854140	0.319006
29	9	0	-4.349714	-1.115084	1.129056

1a:

101aa_POcpent3Me_Ph_MP2_6311++2d2p_PCMthf.log

Input orientation:

Center Number	Atomic Number	Atomic Type	Coordinates (Angstroms) X	Y	Z
1	15	0	-0.515101	-0.463334	0.577065
2	6	0	-1.547281	1.022856	0.330404
3	1	0	-1.024166	1.784400	-0.246167
4	1	0	-1.822318	1.452588	1.291387
5	6	0	-1.265087	-1.419197	-0.789907
6	1	0	-0.650737	-1.376503	-1.687275
7	1	0	-1.369137	-2.460925	-0.496747
8	6	0	-2.584937	-0.723589	-0.980317
9	1	0	-3.373357	-1.189316	-1.554917
10	6	0	-2.738462	0.490844	-0.427707
11	6	0	-3.970456	1.328387	-0.530238
12	1	0	-4.737721	0.830982	-1.115604
13	1	0	-4.370582	1.541061	0.459164
14	1	0	-3.744593	2.285822	-0.996024
15	6	0	1.185219	-0.060074	0.125409
16	6	0	1.988781	-1.010995	-0.512803
17	6	0	1.733972	1.177757	0.477424
18	6	0	3.320139	-0.723774	-0.804256
19	1	0	1.581761	-1.975044	-0.783184
20	6	0	3.065383	1.464488	0.185637
21	1	0	1.128246	1.919393	0.978597
22	6	0	3.859411	0.514455	-0.456244
23	1	0	3.932692	-1.461664	-1.299987
24	1	0	3.480369	2.423359	0.457332
25	1	0	4.890430	0.737921	-0.684877
26	8	0	-0.647226	-1.099260	1.933366

1a+H2:

101ab_POcpent3Me_Ph_+H2OL_MP2_6311++2d2p_PCMthf.log

Input orientation:

Center Number	Atomic Number	Atomic Type	Coordinates (Angstroms) X	Y	Z
1	15	0	-0.479861	-0.758064	0.726515
2	6	0	-1.609954	0.679981	0.848665
3	1	0	-1.088106	1.585417	0.547348
4	1	0	-1.953149	0.803957	1.871220
5	6	0	-1.282452	-1.482620	-0.744086
6	1	0	-0.579768	-1.932183	-1.435819
7	1	0	-1.947282	-2.262452	-0.376001
8	6	0	-2.091280	-0.325212	-1.330183
9	1	0	-2.830459	-0.672225	-2.048026
10	6	0	-2.744321	0.392775	-0.146470
11	6	0	-3.497091	1.648480	-0.548287
12	1	0	-4.294889	1.416497	-1.249791
13	1	0	-3.938179	2.138020	0.315991
14	1	0	-2.819588	2.353849	-1.026180
15	6	0	1.119387	-0.113953	0.168384
16	6	0	1.936006	-0.886911	-0.665243
17	6	0	1.598118	1.114233	0.638686
18	6	0	3.198645	-0.431472	-1.037408
19	1	0	1.596309	-1.847044	-1.024845
20	6	0	2.862467	1.568103	0.270752
21	1	0	0.992547	1.720117	1.296834
22	6	0	3.663129	0.798122	-0.572098
23	1	0	3.816540	-1.034291	-1.685797
24	1	0	3.219413	2.518176	0.638920
25	1	0	4.640769	1.152238	-0.862058
26	1	0	-1.429555	0.370448	-1.849416
27	1	0	-3.438658	-0.308023	0.320113
28	8	0	-0.372237	-1.643634	1.938999

1a:

101ab_POcpent3Me_Ph_MP2_6311++2d2p_PCMthf.log

Input orientation:

Center Number	Atomic Number	Atomic Type	Coordinates (Angstroms) X	Y	Z
1	15	0	-0.502554	1.173450	-0.018418
2	6	0	-1.651850	0.261377	-1.109232
3	1	0	-1.112869	-0.287489	-1.878789
4	1	0	-2.313723	0.970034	-1.607145
5	6	0	-1.356144	0.735401	1.540611
6	1	0	-0.640031	0.462373	2.311748
7	1	0	-1.912228	1.603450	1.892671
8	6	0	-2.258338	-0.395716	1.137961
9	1	0	-2.778026	-0.978582	1.886190
10	6	0	-2.412379	-0.642311	-0.174306
11	6	0	-3.281661	-1.714449	-0.743120
12	1	0	-3.784112	-2.272837	0.041002
13	1	0	-4.034029	-1.286743	-1.403110
14	1	0	-2.690690	-2.406630	-1.340671
15	6	0	1.024676	0.205059	-0.021395
16	6	0	1.025269	-1.175873	0.208264
17	6	0	2.230887	0.867381	-0.260503
18	6	0	2.224755	-1.883169	0.199749
19	1	0	0.097046	-1.697678	0.394971
20	6	0	3.430133	0.156702	-0.269017
21	1	0	2.220529	1.932277	-0.438408
22	6	0	3.428523	-1.218219	-0.038743
23	1	0	2.220861	-2.948052	0.377997
24	1	0	4.359690	0.673640	-0.454455
25	1	0	4.357048	-1.768934	-0.044849
26	8	0	-0.267976	2.629783	-0.302025

1a+H2:

101abb_POcpent3Me_Ph_+H2OL_MP2_6311++2d2p_PCMthf.log

Input orientation:

Center Number	Atomic Number	Atomic Type	Coordinates (Angstroms) X	Y	Z
1	15	0	-0.438337	0.973129	0.013960
2	6	0	-1.546941	0.035961	-1.095346
3	1	0	-1.063217	-0.897668	-1.381467
4	1	0	-1.748971	0.609189	-1.995160
5	6	0	-1.269084	0.446651	1.559024
6	1	0	-0.557888	0.131546	2.315175
7	1	0	-1.792682	1.327396	1.926541
8	6	0	-2.265891	-0.637076	1.136552
9	1	0	-3.074423	-0.739337	1.856244
10	6	0	-2.792105	-0.260143	-0.251686
11	6	0	-3.673913	-1.334266	-0.863358
12	1	0	-4.539932	-1.531403	-0.236042
13	1	0	-4.028724	-1.040881	-1.847687
14	1	0	-3.114025	-2.261876	-0.969353
15	6	0	1.181752	0.170809	-0.028666
16	6	0	1.323804	-1.204218	0.186372
17	6	0	2.314015	0.952711	-0.266682
18	6	0	2.586833	-1.789837	0.163163
19	1	0	0.458012	-1.824633	0.372348
20	6	0	3.577904	0.365307	-0.293379
21	1	0	2.194796	2.013457	-0.429230
22	6	0	3.715675	-1.005275	-0.078168
23	1	0	2.690481	-2.851419	0.330449
24	1	0	4.449799	0.974095	-0.480146
25	1	0	4.694375	-1.460376	-0.098339
26	1	0	-1.767665	-1.605210	1.071250
27	1	0	-3.366060	0.663140	-0.153088
28	8	0	-0.351849	2.457488	-0.216647

4a:

102aa_POcpent2Me_Ph_MP2_6311++2d2p_PCMthf.log

Input orientation:

Center Number	Atomic Number	Atomic Type	Coordinates (Angstroms) X	Y	Z
1	15	0	-0.336583	1.465039	0.290590
2	6	0	-1.727659	0.387858	-0.024618
3	6	0	-0.728818	1.630151	2.065622
4	1	0	0.166407	1.639515	2.677459
5	1	0	-1.226094	2.592014	2.167116
6	6	0	-1.692787	0.484509	2.398110
7	1	0	-2.470014	0.798056	3.092151
8	6	0	-2.306726	-0.025254	1.115756
9	6	0	-3.458350	-0.969515	1.204233
10	1	0	-4.290962	-0.494327	1.719672
11	1	0	-3.790073	-1.294729	0.224195
12	1	0	-3.181746	-1.844273	1.790306
13	6	0	1.140429	0.425302	0.169442
14	6	0	2.320980	0.979253	-0.332714
15	6	0	1.132083	-0.903120	0.607576
16	6	0	3.483311	0.212900	-0.393955
17	1	0	2.317575	2.002056	-0.678622
18	6	0	2.295210	-1.667228	0.549497
19	1	0	0.217837	-1.346029	0.977855
20	6	0	3.472548	-1.109378	0.049631
21	1	0	4.391696	0.644123	-0.787223
22	1	0	2.282267	-2.693355	0.885209
23	1	0	4.372741	-1.703362	0.000813
24	1	0	-1.164351	-0.338453	2.880338
25	1	0	-2.040594	0.063038	-1.004775
26	8	0	-0.192528	2.737036	-0.497239

4a:
102ab_POcpent2Me_Ph_MP2_6311++2d2p_PCMthf.log

Input orientation:

Center Number	Atomic Number	Atomic Type	Coordinates (Angstroms) X	Y	Z
1	15	0	-0.493053	1.149587	-0.184321
2	6	0	-1.640440	0.130244	-1.100431
3	6	0	-1.353214	0.879428	1.402829
4	1	0	-0.656178	0.775029	2.226900
5	1	0	-1.950689	1.773121	1.567476
6	6	0	-2.254675	-0.344172	1.196885
7	1	0	-3.213881	-0.229105	1.697656
8	6	0	-2.456758	-0.558466	-0.284486
9	6	0	-3.493946	-1.535103	-0.727342
10	1	0	-4.474929	-1.222367	-0.374372
11	1	0	-3.522458	-1.631990	-1.807192
12	1	0	-3.298334	-2.513268	-0.291315
13	6	0	1.057656	0.218319	-0.111494
14	6	0	2.269020	0.914554	-0.132738
15	6	0	1.063649	-1.175372	0.008388
16	6	0	3.475366	0.224089	-0.032512
17	1	0	2.257252	1.989276	-0.235803
18	6	0	2.269692	-1.864410	0.112300
19	1	0	0.131784	-1.723764	0.004443
20	6	0	3.477000	-1.164979	0.093125
21	1	0	4.408786	0.766337	-0.054226
22	1	0	2.268757	-2.940523	0.200544
23	1	0	4.411691	-1.699851	0.169338
24	1	0	-1.794522	-1.239135	1.616826
25	1	0	-1.651076	0.034611	-2.175085
26	8	0	-0.271079	2.575310	-0.605579

11a:
103aa_POcpent3Me_Ph_ikerion_MP2_6311++2d2p_PCMthf.log

Input orientation:

Center Number	Atomic Number	Atomic Type	Coordinates (Angstroms) X	Y	Z
1	15	0	-0.520553	0.614193	-0.213557
2	6	0	-1.647237	-0.399514	-0.932279
3	6	0	-1.330888	0.715713	1.420421
4	1	0	-0.712644	0.186779	2.145544
5	1	0	-1.397733	1.755006	1.737327
6	6	0	-2.660893	0.046685	1.192873
7	1	0	-3.429142	0.029436	1.949587
8	6	0	-2.787370	-0.527487	-0.029487
9	6	0	-4.004750	-1.274916	-0.477103
10	1	0	-4.756610	-1.303256	0.306238
11	1	0	-4.439678	-0.810755	-1.360212
12	1	0	-3.748886	-2.297721	-0.747533
13	6	0	1.199802	0.091918	-0.103448
14	6	0	2.273670	0.987978	-0.149172
15	6	0	1.444461	-1.273074	0.081654
16	6	0	3.577440	0.517942	-0.008295
17	1	0	2.091205	2.040055	-0.297548
18	6	0	2.749449	-1.738608	0.221515
19	1	0	0.612363	-1.962125	0.105768
20	6	0	3.817809	-0.843362	0.177739
21	1	0	4.403002	1.212609	-0.046352
22	1	0	2.931288	-2.793777	0.359197
23	1	0	4.829877	-1.203740	0.284049
24	8	0	-0.249980	2.122902	-0.810806
25	1	0	-1.085522	2.500829	-1.112560
26	1	0	-1.596696	-0.835248	-1.914592

11a+H2:

103ab_POcpent3Me_Ph_ikerion_+H2OL_MP2_6311++2d2p_PCMthf.log

Input orientation:

Center Number	Atomic Number	Atomic Type	Coordinates (Angstroms) X	Y	Z
1	15	0	0.462057	-0.733063	-0.365764
2	6	0	0.396110	0.591718	-1.367324
3	6	0	2.263251	-0.739592	-0.093150
4	1	0	2.521697	-0.960319	0.938078
5	1	0	2.690305	-1.518552	-0.723139
6	6	0	2.687054	0.656394	-0.555052
7	1	0	3.742249	0.684182	-0.823993
8	6	0	1.786414	1.084459	-1.724798
9	6	0	1.822388	2.593267	-1.922248
10	1	0	2.835751	2.937914	-2.124084
11	1	0	1.192051	2.895992	-2.754933
12	1	0	1.459124	3.091221	-1.024913
13	6	0	-0.506561	-0.722286	1.162435
14	6	0	-0.900861	-1.890299	1.827298
15	6	0	-0.812274	0.522536	1.721390
16	6	0	-1.590086	-1.807547	3.034681
17	1	0	-0.675369	-2.854256	1.400075
18	6	0	-1.509164	0.602658	2.925418
19	1	0	-0.507363	1.418083	1.199261
20	6	0	-1.896330	-0.562519	3.585666
21	1	0	-1.891201	-2.711807	3.542197
22	1	0	-1.748399	1.568340	3.345053
23	1	0	-2.434572	-0.502304	4.519666
24	1	0	2.525508	1.361515	0.259743
25	1	0	2.172342	0.623249	-2.641274
26	8	0	0.019571	-2.259381	-0.835183
27	1	0	0.331085	-2.414258	-1.735115
28	1	0	-0.499406	0.997504	-1.809462

11a:

104aa_POcpent3Me_Ph_ikerion_B_MP2_6311++2d2p_PCMthf.log

Input orientation:

Center Number	Atomic Number	Atomic Type	Coordinates (Angstroms) X	Y	Z
1	15	0	-0.502847	0.611547	0.366775
2	6	0	-1.597611	0.148266	-1.020393
3	1	0	-1.079995	-0.572307	-1.654689
4	1	0	-1.825454	1.021550	-1.630500
5	6	0	-1.388947	-0.143572	1.573659
6	6	0	-2.647867	-0.594348	0.983393
7	1	0	-3.421311	-1.027238	1.605499
8	6	0	-2.809328	-0.458022	-0.355110
9	6	0	-4.009696	-0.841453	-1.156684
10	1	0	-4.766422	-1.289651	-0.517456
11	1	0	-4.459043	0.018720	-1.653579
12	1	0	-3.760093	-1.561359	-1.936640
13	6	0	1.212739	0.133626	0.090632
14	6	0	1.542609	-1.211553	0.288523
15	6	0	2.195097	1.029803	-0.345699
16	6	0	2.840472	-1.657675	0.052090
17	1	0	0.783421	-1.897492	0.636172
18	6	0	3.491845	0.579066	-0.582189
19	1	0	1.948294	2.068619	-0.493910
20	6	0	3.816825	-0.762937	-0.384871
21	1	0	3.088854	-2.696028	0.212015
22	1	0	4.246711	1.274733	-0.916600
23	1	0	4.823719	-1.107441	-0.566392
24	8	0	-0.268826	2.242232	0.380298
25	1	0	-1.061286	2.671501	0.726342
26	1	0	-1.120963	-0.243305	2.609730

1a+H+:
105aa_POcpent3Me_Ph_+H+_MP2_6311++2d2p_PCMthf.log

Input orientation:

Center Number	Atomic Number	Atomic Type	Coordinates (Angstroms) X	Y	Z
1	15	0	0.468782	0.170458	0.471489
2	6	0	1.572105	-1.083897	-0.184053
3	1	0	1.147679	-1.541006	-1.075987
4	1	0	1.742032	-1.861965	0.556611
5	6	0	1.289243	1.624887	-0.205860
6	1	0	0.783502	1.967710	-1.106188
7	1	0	1.296111	2.436833	0.516148
8	6	0	2.658577	1.061846	-0.500308
9	1	0	3.472750	1.734299	-0.723308
10	6	0	2.811935	-0.271324	-0.498009
11	6	0	4.079839	-0.996544	-0.797485
12	1	0	4.881686	-0.302158	-1.024132
13	1	0	4.376031	-1.610038	0.050217
14	1	0	3.943947	-1.662881	-1.646428
15	6	0	-1.248804	0.011519	0.067809
16	6	0	-1.957329	1.079811	-0.493122
17	6	0	-1.884897	-1.208283	0.336837
18	6	0	-3.308487	0.921378	-0.787169
19	1	0	-1.472175	2.021771	-0.697519
20	6	0	-3.233524	-1.354258	0.034181
21	1	0	-1.338462	-2.032723	0.772087
22	6	0	-3.943648	-0.291579	-0.526242
23	1	0	-3.860400	1.741616	-1.218685
24	1	0	-3.727641	-2.292291	0.233009
25	1	0	-4.990222	-0.410197	-0.760388
26	8	0	0.700148	0.059810	2.045546
27	1	0	0.172341	0.670687	2.576723

1a-H+:
105ab_POcpent3Me_Ph_-H+A_MP2_6311++2d2p_PCMthf.log

Input orientation:

Center Number	Atomic Number	Atomic Type	Coordinates (Angstroms) X	Y	Z
1	15	0	-0.573524	1.177128	-0.310821
2	6	0	-1.756185	0.183961	-1.090610
3	6	0	-1.306421	0.983308	1.371329
4	1	0	-0.525097	0.891127	2.122834
5	1	0	-1.859219	1.906490	1.575451
6	6	0	-2.216419	-0.209257	1.231075
7	1	0	-2.791760	-0.608202	2.054952
8	6	0	-2.447917	-0.556755	-0.074639
9	6	0	-3.394739	-1.659873	-0.451377
10	1	0	-3.834797	-2.119131	0.430605
11	1	0	-4.198110	-1.282937	-1.082542
12	1	0	-2.880832	-2.434149	-1.020270
13	6	0	1.009569	0.257669	-0.160926
14	6	0	2.225499	0.920557	0.033838
15	6	0	0.999631	-1.141030	-0.183026
16	6	0	3.410564	0.202269	0.194497
17	1	0	2.238104	2.000760	0.042847
18	6	0	2.180097	-1.864697	-0.015752
19	1	0	0.056932	-1.648106	-0.341343
20	6	0	3.389127	-1.193186	0.173399
21	1	0	4.346051	0.725547	0.331908
22	1	0	2.160381	-2.944989	-0.036308
23	1	0	4.305682	-1.751378	0.297345
24	8	0	-0.233101	2.592481	-0.750979
25	1	0	-1.693283	-0.158737	-2.112491

1a+H+:

105ba_POcpent3Me_Ph_+H+_MP2_6311++2d2p_PCMthf.log

Input orientation:

Center Number	Atomic Number	Atomic Type	Coordinates (Angstroms) X	Y	Z
1	15	0	-0.467911	-0.273697	0.425646
2	6	0	-1.566271	1.088838	0.011005
3	1	0	-1.150237	1.673453	-0.808237
4	1	0	-1.716194	1.747296	0.863095
5	6	0	-1.290222	-1.557344	-0.533443
6	1	0	-0.795252	-1.682028	-1.494918
7	1	0	-1.267520	-2.507818	-0.007520
8	6	0	-2.669988	-0.960135	-0.671369
9	1	0	-3.491245	-1.579286	-0.998425
10	6	0	-2.820623	0.348778	-0.409592
11	6	0	-4.092587	1.116107	-0.536521
12	1	0	-4.905352	0.473924	-0.858138
13	1	0	-4.360067	1.573653	0.413009
14	1	0	-3.975410	1.920364	-1.259558
15	6	0	1.241868	-0.016509	0.071546
16	6	0	2.041606	-1.103707	-0.300040
17	6	0	1.782707	1.269629	0.184367
18	6	0	3.392560	-0.896433	-0.557042
19	1	0	1.622236	-2.095154	-0.388941
20	6	0	3.135207	1.462738	-0.076182
21	1	0	1.163675	2.107443	0.470438
22	6	0	3.936988	0.382866	-0.445679
23	1	0	4.015378	-1.728589	-0.845461
24	1	0	3.559612	2.450995	0.006114
25	1	0	4.984931	0.538742	-0.650005
26	8	0	-0.531966	-0.602272	1.984130
27	1	0	-1.419188	-0.775176	2.328527

1a+H2:

106aa_POcpent3Me_Ph_+H+_+H2OL_MP2_6311++2d2p_PCMthf.log

Input orientation:

Center Number	Atomic Number	Atomic Type	Coordinates (Angstroms) X	Y	Z
1	15	0	0.461626	-0.733271	-0.366637
2	6	0	0.405529	0.590566	-1.370107
3	6	0	2.261030	-0.744335	-0.082774
4	1	0	2.512465	-0.964822	0.950243
5	1	0	2.689996	-1.524970	-0.709384
6	6	0	2.691400	0.650134	-0.543226
7	1	0	3.748352	0.674937	-0.805470
8	6	0	1.799318	1.079505	-1.719054
9	6	0	1.840336	2.588061	-1.917463
10	1	0	2.855826	2.930028	-2.113085
11	1	0	1.216085	2.891730	-2.754382
12	1	0	1.472606	3.087635	-1.022853
13	6	0	-0.516159	-0.717723	1.155702
14	6	0	-0.916727	-1.883647	1.820462
15	6	0	-0.822685	0.528743	1.710451
16	6	0	-1.612892	-1.797240	3.023599
17	1	0	-0.690677	-2.848894	1.396434
18	6	0	-1.526554	0.612562	2.910165
19	1	0	-0.512853	1.422664	1.188438
20	6	0	-1.919929	-0.550555	3.570367
21	1	0	-1.918757	-2.699940	3.531051
22	1	0	-1.766365	1.579533	3.326492
23	1	0	2.526495	1.356397	0.269906
24	1	0	2.189958	0.616604	-2.632674
25	8	0	0.017707	-2.258742	-0.836759
26	1	0	-0.486174	0.998241	-1.818175
27	1	0	0.333718	-2.415752	-1.734738
28	1	0	-2.463569	-0.487475	4.501046

TS(1a->11a):

109ba_POcpent3Me_Ph_ikerion_TS_HO_MP2_6311++2d2p_PCMthf.log

Input orientation:

Center Number	Atomic Number	Atomic Type	Coordinates (Angstroms) X	Y	Z
1	15	0	-0.474270	0.498451	0.262430
2	6	0	-1.685596	0.355728	-0.995225
3	6	0	-1.283804	-0.521060	1.525808
4	1	0	-0.721738	-1.439613	1.690949
5	1	0	-1.312970	0.026459	2.466552
6	6	0	-2.640977	-0.759386	0.915471
7	1	0	-3.436709	-1.202278	1.496079
8	6	0	-2.806524	-0.365639	-0.371047
9	6	0	-4.077653	-0.558312	-1.137454
10	1	0	-4.821860	-1.079410	-0.542396
11	1	0	-4.487213	0.400662	-1.447655
12	1	0	-3.892302	-1.134646	-2.041875
13	6	0	1.249958	0.081471	0.039119
14	6	0	2.205632	1.097938	-0.051812
15	6	0	1.637960	-1.258949	-0.074242
16	6	0	3.545277	0.771856	-0.250336
17	1	0	1.896994	2.128119	0.038236
18	6	0	2.978183	-1.577418	-0.270195
19	1	0	0.902317	-2.048861	-0.020960
20	6	0	3.932801	-0.563147	-0.358123
21	1	0	4.282926	1.557045	-0.317300
22	1	0	3.275987	-2.611219	-0.357330
23	1	0	4.971757	-0.813135	-0.510214
24	8	0	-0.658351	2.056901	0.463896
25	1	0	-1.482301	1.816873	-0.419221
26	1	0	-1.458415	0.213186	-2.042127

TS(11a->4a):

110aa_POcpent3Me_Ph_ikerion_A_TS2_MP2_6311++2d2p_PCMthf.log

Input orientation:

Center Number	Atomic Number	Atomic Type	Coordinates (Angstroms) X	Y	Z
1	15	0	0.475506	-0.283096	-0.054821
2	6	0	1.542659	1.063334	-0.395371
3	6	0	1.375375	-0.806842	1.412032
4	6	0	2.715215	-0.744705	0.644375
5	1	0	3.606658	-1.092250	1.148364
6	6	0	2.769848	0.510254	-0.056464
7	6	0	4.075742	1.107742	-0.477683
8	1	0	4.685261	0.359552	-0.978645
9	1	0	3.936652	1.950828	-1.146464
10	1	0	4.631111	1.442652	0.395961
11	6	0	-1.278352	-0.010137	-0.051479
12	6	0	-1.768921	1.284705	0.145243
13	6	0	-2.156066	-1.087992	-0.210078
14	6	0	-3.144173	1.498338	0.187545
15	1	0	-1.083250	2.111734	0.258762
16	6	0	-3.528701	-0.863870	-0.169353
17	1	0	-1.766829	-2.081819	-0.371976
18	6	0	-4.022314	0.426140	0.031271
19	1	0	-3.527930	2.496116	0.335432
20	1	0	-4.210786	-1.690722	-0.294999
21	1	0	-5.087924	0.595533	0.060049
22	8	0	0.840816	-1.516770	-0.985354
23	1	0	1.944790	-1.510846	-0.517987
24	1	0	1.405430	1.859032	-1.107137
25	1	0	1.310533	-0.088952	2.228391
26	1	0	1.075217	-1.802179	1.722750

303ab_POcpent_Ph_+H+_MP2_6311++2d2p_PCMthf.log

Input orientation:

Center Number	Atomic Number	Atomic Type	Coordinates (Angstroms) X	Y	Z
1	15	0	-0.962630	0.666794	-0.156203
2	6	0	-1.911592	-0.321070	-1.328701
3	1	0	-1.288633	-0.677773	-2.142763
4	1	0	-2.714312	0.280409	-1.753792
5	6	0	-1.833783	0.167241	1.346138
6	1	0	-1.148490	0.105399	2.185431
7	1	0	-2.623747	0.875410	1.592141
8	6	0	-2.381079	-1.167591	0.904348
9	1	0	-2.731328	-1.876555	1.637204
10	6	0	-2.422469	-1.408163	-0.414693
11	6	0	0.737011	0.171739	-0.119631
12	6	0	1.092518	-1.124617	-0.508555
13	6	0	1.701332	1.067777	0.356493
14	6	0	2.423231	-1.521059	-0.420048
15	1	0	0.350852	-1.820195	-0.871166
16	6	0	3.028848	0.659787	0.432715
17	1	0	1.423412	2.068115	0.650397
18	6	0	3.389241	-0.631400	0.048051
19	1	0	2.703146	-2.518590	-0.720380
20	1	0	3.778113	1.348954	0.789558
21	1	0	4.420650	-0.942358	0.109313
22	8	0	-0.905524	2.230610	-0.422503
23	1	0	-2.812084	-2.324313	-0.829002
24	1	0	-1.742053	2.704370	-0.328119

1b:

101ba_POcpent3Me_pMePh_MP2_6311++2d2p_PCMthf.log

Input orientation:

Center Number	Atomic Number	Atomic Type	Coordinates (Angstroms) X	Y	Z
1	15	0	-0.970002	-0.551043	0.514239
2	6	0	-1.907856	1.015001	0.443715
3	1	0	-1.315518	1.818774	0.008958
4	1	0	-2.208880	1.317492	1.444582
5	6	0	-1.705804	-1.257769	-1.004429
6	1	0	-1.046382	-1.131088	-1.860892
7	1	0	-1.884647	-2.321182	-0.866221
8	6	0	-2.972357	-0.459684	-1.149160
9	1	0	-3.755275	-0.788496	-1.818274
10	6	0	-3.085730	0.671518	-0.434115
11	6	0	-4.262506	1.590311	-0.466624
12	1	0	-5.024367	1.231469	-1.151903
13	1	0	-4.701672	1.682336	0.524846
14	1	0	-3.960062	2.589118	-0.775769
15	6	0	0.766166	-0.188990	0.192158
16	6	0	1.551887	-1.077041	-0.549097
17	6	0	1.368262	0.943904	0.749071
18	6	0	2.908287	-0.827102	-0.737310
19	1	0	1.113455	-1.967008	-0.978310
20	6	0	2.725116	1.186092	0.555897
21	1	0	0.785259	1.638662	1.337232
22	6	0	3.515548	0.308052	-0.192059
23	1	0	3.502993	-1.523694	-1.311876
24	1	0	3.176259	2.065756	0.993819
25	8	0	-1.204527	-1.362712	1.758721
26	6	0	4.972433	0.590923	-0.424858
27	1	0	5.530003	-0.329003	-0.571734
28	1	0	5.106256	1.204866	-1.312934
29	1	0	5.404974	1.126562	0.414752

1b:

101bab_POcpent3Me_pMePh_MP2_6311++2d2p_PCMthf.log

Input orientation:

Center Number	Atomic Number	Atomic Type	Coordinates (Angstroms) X	Y	Z
1	15	0	-1.051061	1.197298	-0.104959
2	6	0	-2.073343	0.076199	-1.123574
3	1	0	-1.479727	-0.407578	-1.896667
4	1	0	-2.855808	0.652915	-1.616905
5	6	0	-1.816460	0.722967	1.489207
6	1	0	-1.064636	0.625710	2.268223
7	1	0	-2.514191	1.503789	1.789958
8	6	0	-2.511209	-0.568365	1.165273
9	1	0	-2.902952	-1.197577	1.952856
10	6	0	-2.646807	-0.900646	-0.130254
11	6	0	-3.325455	-2.133622	-0.627967
12	1	0	-3.703837	-2.734956	0.193196
13	1	0	-4.156100	-1.874983	-1.282059
14	1	0	-2.635616	-2.738428	-1.214463
15	6	0	0.595672	0.455615	-0.088796
16	6	0	1.706172	1.301352	-0.134095
17	6	0	0.794737	-0.927987	-0.020478
18	6	0	2.993898	0.770816	-0.105766
19	1	0	1.556152	2.368702	-0.202065
20	6	0	2.085592	-1.447059	0.009194
21	1	0	-0.049978	-1.601621	0.009454
22	6	0	3.204873	-0.608419	-0.029868
23	1	0	3.844897	1.436486	-0.149631
24	1	0	2.226302	-2.518303	0.055636
25	8	0	-1.029312	2.658384	-0.454079
26	6	0	4.594198	-1.177478	0.030179
27	1	0	5.311748	-0.506546	-0.432052
28	1	0	4.900883	-1.330885	1.062653
29	1	0	4.645366	-2.137820	-0.474274

1b+H2:

101bb_POcpent3Me_pMePh_+H2OL_MP2_6311++2d2p_PCMthf_FRQ.log

Input orientation:

Center Number	Atomic Number	Atomic Type	Coordinates (Angstroms) X	Y	Z
1	15	0	0.923639	-0.999669	0.007443
2	6	0	1.916857	0.067412	-1.094536
3	1	0	1.330892	0.944173	-1.369228
4	1	0	2.177368	-0.470216	-2.001304
5	6	0	1.702963	-0.405515	1.555054
6	1	0	0.967339	-0.184247	2.320998
7	1	0	2.325915	-1.227165	1.904161
8	6	0	2.568048	0.789411	1.143121
9	1	0	3.364134	0.973635	1.860439
10	6	0	3.125402	0.491795	-0.252322
11	6	0	3.877009	1.666268	-0.853448
12	1	0	4.719330	1.951560	-0.227705
13	1	0	4.256294	1.427609	-1.843439
14	1	0	3.215607	2.526247	-0.943941
15	6	0	-0.773378	-0.382168	-0.018877
16	6	0	-1.071224	0.966666	0.201383
17	6	0	-1.817166	-1.280805	-0.244298
18	6	0	-2.392250	1.402963	0.192650
19	1	0	-0.282353	1.683569	0.383454
20	6	0	-3.137015	-0.834429	-0.254207
21	1	0	-1.589058	-2.323697	-0.407390
22	6	0	-3.445732	0.511055	-0.038062
23	1	0	-2.609208	2.447552	0.369472
24	1	0	-3.936900	-1.541124	-0.427482
25	1	0	1.962944	1.695625	1.092906
26	1	0	3.800678	-0.361876	-0.168222
27	8	0	1.003073	-2.481819	-0.241177
28	6	0	-4.867141	0.996529	-0.077348

29	1	0	-5.027645	1.793913	0.642308
30	1	0	-5.111374	1.388086	-1.062509
31	1	0	-5.561366	0.190850	0.140594

4b:
102ba_POcpent2Me_pMePh_MP2_6311++2d2p_PCMthf.log

Input orientation:

Center Number	Atomic Number	Atomic Type	Coordinates (Angstroms) X	Y	Z
1	15	0	-1.014491	1.199032	-0.222925
2	6	0	-2.040328	0.024841	-1.098105
3	6	0	-1.812977	0.858835	1.382689
4	1	0	-1.100851	0.877096	2.200273
5	1	0	-2.530256	1.663042	1.530290
6	6	0	-2.534116	-0.484592	1.219990
7	1	0	-3.486284	-0.499795	1.746498
8	6	0	-2.740804	-0.750462	-0.252639
9	6	0	-3.648780	-1.863820	-0.654977
10	1	0	-4.654593	-1.677649	-0.282917
11	1	0	-3.688317	-1.983670	-1.732200
12	1	0	-3.315257	-2.799301	-0.208952
13	6	0	0.637905	0.467552	-0.158513
14	6	0	1.755083	1.306497	-0.137741
15	6	0	0.824461	-0.915903	-0.078000
16	6	0	3.034996	0.767270	-0.038039
17	1	0	1.616579	2.375438	-0.205460
18	6	0	2.107878	-1.445740	0.023411
19	1	0	-0.027288	-1.581273	-0.108188
20	6	0	3.232480	-0.614531	0.044701
21	1	0	3.891444	1.427366	-0.022568
22	1	0	2.238554	-2.517536	0.084442
23	1	0	-1.937968	-1.296372	1.638223
24	1	0	-2.060505	-0.091111	-2.170649
25	8	0	-0.979869	2.632422	-0.674960
26	6	0	4.617194	-1.193314	0.118614
27	1	0	5.007709	-1.381341	-0.879253
28	1	0	5.299731	-0.511246	0.616574
29	1	0	4.618542	-2.136489	0.656689

11b:
103ba_POcpent3Me_pMePh_ikerion_MP2_6311++2d2p_PCMthf.log

Input orientation:

Center Number	Atomic Number	Atomic Type	Coordinates (Angstroms) X	Y	Z
1	15	0	-0.974344	0.643605	-0.205190
2	6	0	-2.024812	-0.444165	-0.932105
3	6	0	-1.767515	0.642523	1.440667
4	1	0	-1.097661	0.149118	2.144740
5	1	0	-1.913607	1.665364	1.783322
6	6	0	-3.042002	-0.127336	1.214354
7	1	0	-3.795199	-0.224797	1.980104
8	6	0	-3.138249	-0.683634	-0.019140
9	6	0	-4.296870	-1.518577	-0.468191
10	1	0	-5.032543	-1.625495	0.323721
11	1	0	-4.780981	-1.072703	-1.334994
12	1	0	-3.962222	-2.510869	-0.765019
13	6	0	0.782397	0.265033	-0.136873
14	6	0	1.777755	1.247984	-0.145746
15	6	0	1.150756	-1.078137	-0.011774
16	6	0	3.116377	0.883235	-0.033635
17	1	0	1.509294	2.287865	-0.242011
18	6	0	2.492740	-1.429815	0.098225
19	1	0	0.384945	-1.840737	-0.008068
20	6	0	3.496566	-0.456878	0.088923
21	1	0	3.876889	1.651864	-0.039308
22	1	0	2.763445	-2.472300	0.193379
23	8	0	-0.841666	2.184588	-0.767952

24	1	0	-1.713088	2.503128	-1.034519
25	1	0	-1.953412	-0.852459	-1.924886
26	6	0	4.945875	-0.842480	0.176167
27	1	0	5.358021	-1.007556	-0.817051
28	1	0	5.530649	-0.060042	0.650245
29	1	0	5.072593	-1.759939	0.742823

1b+H2:

103bb_POcpent3Me_pMePh_ikerion_+H2OL_MP2_6311++2d2p_PCMthf.log

Input orientation:

Center Number	Atomic Number	Atomic Type	Coordinates (Angstroms) X	Y	Z
1	15	0	-0.952835	0.695599	-0.295926
2	6	0	-1.931080	-0.488220	-0.934776
3	6	0	-1.889333	1.000073	1.234243
4	1	0	-1.245938	1.030788	2.107836
5	1	0	-2.373095	1.968862	1.130977
6	6	0	-2.904814	-0.147530	1.255138
7	1	0	-3.790253	0.122411	1.828881
8	6	0	-3.254326	-0.529795	-0.193321
9	6	0	-3.898691	-1.906528	-0.264376
10	1	0	-4.812104	-1.943134	0.327353
11	1	0	-4.150723	-2.169712	-1.288835
12	1	0	-3.208578	-2.656742	0.117742
13	6	0	0.795631	0.263115	-0.160798
14	6	0	1.792882	1.231851	0.009316
15	6	0	1.161947	-1.086764	-0.178184
16	6	0	3.123844	0.851040	0.153141
17	1	0	1.529714	2.278189	0.032193
18	6	0	2.498219	-1.455099	-0.050444
19	1	0	0.392843	-1.836462	-0.291225
20	6	0	3.500221	-0.495382	0.120364
21	1	0	3.880884	1.610445	0.293510
22	1	0	2.765250	-2.502811	-0.071637
23	1	0	-2.448601	-1.019145	1.722246
24	1	0	-3.980747	0.197538	-0.573384
25	8	0	-0.834557	2.272825	-0.831953
26	1	0	-0.369183	2.311445	-1.675491
27	1	0	-1.764492	-1.007562	-1.865071
28	6	0	4.943315	-0.895859	0.238686
29	1	0	5.421950	-0.895506	-0.738370
30	1	0	5.490176	-0.205080	0.873464
31	1	0	5.038861	-1.894857	0.653062

11b:

104ba_POcpent3Me_pMePh_ikerion_B_MP2_6311++2d2p_PCMthf.log

Input orientation:

Center Number	Atomic Number	Atomic Type	Coordinates (Angstroms) X	Y	Z
1	15	0	-0.958111	0.649422	0.345275
2	6	0	-1.972295	0.032877	-1.044069
3	1	0	-1.386034	-0.686196	-1.617515
4	1	0	-2.237115	0.851369	-1.712384
5	6	0	-1.827601	-0.102370	1.566916
6	6	0	-3.035146	-0.667526	0.968188
7	1	0	-3.797535	-1.118061	1.591493
8	6	0	-3.162254	-0.616305	-0.380104
9	6	0	-4.306557	-1.127588	-1.192305
10	1	0	-5.052437	-1.589403	-0.549978
11	1	0	-4.796006	-0.330900	-1.753087
12	1	0	-3.983095	-1.872506	-1.919960
13	6	0	0.795065	0.292720	0.155796
14	6	0	1.240774	-0.984427	0.509377
15	6	0	1.707313	1.210238	-0.377296
16	6	0	2.577090	-1.333906	0.337339
17	1	0	0.540101	-1.694837	0.924226
18	6	0	3.040047	0.847392	-0.547605

19	1	0	1.379230	2.199467	-0.653846
20	6	0	3.497661	-0.425886	-0.193306
21	1	0	2.908052	-2.324448	0.617281
22	1	0	3.735007	1.563997	-0.963316
23	8	0	-0.848231	2.292533	0.266356
24	1	0	-1.689379	2.678910	0.540330
25	1	0	-1.590751	-0.120880	2.615170
26	6	0	4.945461	-0.794831	-0.350061
27	1	0	5.385551	-0.294171	-1.207266
28	1	0	5.512472	-0.500876	0.530664
29	1	0	5.064230	-1.866448	-0.477534

1b+H+:

105ba_POcpent3Me_pMePh_+H+_MP2_6311++2d2p_PCMthf.log

Input orientation:

Center Number	Atomic Number	Atomic Type	Coordinates (Angstroms) X	Y	Z
1	15	0	0.905090	0.156981	0.448671
2	6	0	1.931522	-1.125419	-0.275745
3	1	0	1.458210	-1.540453	-1.163778
4	1	0	2.091269	-1.928384	0.439863
5	6	0	1.748234	1.587095	-0.252024
6	1	0	1.228502	1.948707	-1.136844
7	1	0	1.797912	2.396703	0.471606
8	6	0	3.089318	0.981737	-0.589186
9	1	0	3.921428	1.627538	-0.824272
10	6	0	3.193472	-0.356239	-0.609632
11	6	0	4.426380	-1.122681	-0.949578
12	1	0	5.247075	-0.455071	-1.189051
13	1	0	4.721198	-1.756338	-0.116305
14	1	0	4.245133	-1.774002	-1.801502
15	6	0	-0.837717	0.069490	0.162161
16	6	0	-1.513723	1.052134	-0.566829
17	6	0	-1.535763	-1.029884	0.682403
18	6	0	-2.884125	0.926019	-0.773084
19	1	0	-0.992366	1.907498	-0.967577
20	6	0	-2.901630	-1.136026	0.464453
21	1	0	-1.020552	-1.790587	1.250975
22	6	0	-3.596474	-0.163236	-0.265796
23	1	0	-3.405296	1.686734	-1.335513
24	1	0	-3.437121	-1.984845	0.864860
25	8	0	1.221501	0.009501	2.005431
26	1	0	0.740127	0.624132	2.574591
27	6	0	-5.074270	-0.296218	-0.485818
28	1	0	-5.452427	0.507222	-1.108470
29	1	0	-5.305872	-1.243129	-0.965875
30	1	0	-5.604003	-0.273303	0.463022

1b+H2:

106ca_POcpent3Me_pMePh_+H+_+H2OL_MP2_6311++2d2p_PCMthf_JO.log

Input orientation:

Center Number	Atomic Number	Atomic Type	Coordinates (Angstroms) X	Y	Z
1	15	0	-1.991778	0.765371	-0.240557
2	6	0	-1.248684	-0.360110	-1.435355
3	6	0	-3.592192	-0.068697	-0.095815
4	1	0	-3.888408	-0.172717	0.941918
5	1	0	-4.327122	0.557967	-0.597735
6	6	0	-3.384641	-1.392566	-0.847738
7	1	0	-4.335425	-1.799001	-1.177521
8	6	0	-2.447364	-1.118086	-2.028107
9	6	0	-2.018517	-2.378033	-2.757511
10	1	0	-2.882462	-2.902099	-3.155985
11	1	0	-1.351260	-2.145914	-3.582237
12	1	0	-1.497707	-3.046936	-2.075807
13	6	0	-1.091211	0.910407	1.276508
14	6	0	-0.730700	2.155984	1.795290

15	6	0	-0.753713	-0.264389	1.959000
16	6	0	-0.028643	2.214507	2.995195
17	1	0	-0.987511	3.062964	1.271651
18	6	0	-0.054876	-0.183080	3.156379
19	1	0	-1.025311	-1.235804	1.570166
20	6	0	0.319673	1.054308	3.692186
21	1	0	0.256346	3.178234	3.391648
22	1	0	0.209660	-1.092207	3.676827
23	1	0	-2.923220	-2.128479	-0.189817
24	1	0	-2.963229	-0.456206	-2.724967
25	8	0	-2.109314	2.267043	-0.758035
26	1	0	-0.636984	0.174932	-2.154718
27	1	0	-2.601225	2.388348	-1.580243
28	6	0	1.059680	1.126875	4.995642
29	1	0	-0.606610	-1.046062	-0.882070
30	1	0	1.571121	2.077707	5.102581
31	1	0	0.368879	1.021395	5.829070
32	1	0	1.790987	0.327944	5.071388

TS(1b->11b):

109ba_POcpent3Me_pMePh_ikerion_TS_HO_MP2_6311++2d2p_PCMthf.log

Input orientation:

Center Number	Atomic Number	Atomic Type	Coordinates (Angstroms) X	Y	Z
1	15	0	-0.918304	0.532538	0.251526
2	6	0	-2.078780	0.254805	-1.032719
3	6	0	-1.718753	-0.453492	1.547197
4	1	0	-1.117538	-1.330267	1.785129
5	1	0	-1.807459	0.146212	2.451724
6	6	0	-3.040470	-0.797563	0.910312
7	1	0	-3.831659	-1.248979	1.490627
8	6	0	-3.181992	-0.487411	-0.401760
9	6	0	-4.414785	-0.791310	-1.194440
10	1	0	-5.151375	-1.315783	-0.592836
11	1	0	-4.861996	0.124963	-1.573964
12	1	0	-4.169485	-1.408493	-2.056677
13	6	0	0.830075	0.199671	0.105861
14	6	0	1.732821	1.256418	-0.039264
15	6	0	1.302255	-1.117981	0.106643
16	6	0	3.092417	0.991643	-0.179219
17	1	0	1.370254	2.273046	-0.033032
18	6	0	2.663013	-1.366218	-0.032661
19	1	0	0.617123	-1.947720	0.207911
20	6	0	3.579040	-0.318440	-0.179244
21	1	0	3.784757	1.815443	-0.282493
22	1	0	3.019376	-2.386880	-0.026136
23	8	0	-1.193272	2.087659	0.356135
24	1	0	-1.971338	1.753693	-0.537464
25	1	0	-1.808792	0.061829	-2.061274
26	6	0	5.043422	-0.600229	-0.358287
27	1	0	5.268407	-0.799509	-1.403830
28	1	0	5.645816	0.246662	-0.044882
29	1	0	5.346284	-1.471171	0.215177

TS(11b->4b):

110aa_POcpent3Me_pMe_b3lyp631dp_SCAN_HOvege_MP2_631++dp_PCMthf_TS2_f.log

Input orientation:

Center Number	Atomic Number	Atomic Type	Coordinates (Angstroms) X	Y	Z
1	15	0	0.903643	-0.285492	-0.084539
2	6	0	1.929904	1.107434	-0.361963
3	1	0	1.757963	1.929483	-1.039570
4	1	0	2.416117	-1.461643	-0.617895
5	6	0	1.835616	-0.853283	1.349428
6	1	0	1.754432	-0.176453	2.204241
7	1	0	1.568161	-1.872634	1.623034
8	6	0	3.167662	-0.716474	0.580653

9	1	0	4.072651	-1.053913	1.074791
10	6	0	3.180503	0.578715	-0.053403
11	6	0	4.466046	1.250045	-0.431481
12	1	0	5.111549	0.550685	-0.962716
13	1	0	4.295050	2.119735	-1.062501
14	1	0	4.998000	1.567284	0.466410
15	8	0	1.315793	-1.478064	-1.082466
16	6	0	-0.860709	-0.080917	-0.071988
17	6	0	-1.409927	1.162552	0.273194
18	6	0	-1.698560	-1.162433	-0.380644
19	6	0	-2.795607	1.312119	0.317309
20	1	0	-0.763986	2.000546	0.504796
21	6	0	-3.081158	-0.992068	-0.332437
22	1	0	-1.272648	-2.118632	-0.654942
23	6	0	-3.649885	0.242604	0.013656
24	1	0	-3.218171	2.272953	0.585050
25	1	0	-3.727607	-1.828337	-0.569842
26	6	0	-5.142392	0.424414	0.025354
27	1	0	-5.436116	1.181125	0.750443
28	1	0	-5.499597	0.743560	-0.954430
29	1	0	-5.645206	-0.507766	0.276009

1e:

1101baa_POcpent3Me_pOMePh_MP2_6311++2d2p_PCMthf.log

Input orientation:

Center Number	Atomic Number	Atomic Type	Coordinates (Angstroms) X	Y	Z
1	15	0	-1.368389	-0.586034	0.478780
2	6	0	-2.326141	0.967094	0.382804
3	1	0	-1.717076	1.793794	0.020228
4	1	0	-2.708566	1.232547	1.366368
5	6	0	-1.984341	-1.265252	-1.104830
6	1	0	-1.266502	-1.114566	-1.908612
7	1	0	-2.162040	-2.332910	-1.001744
8	6	0	-3.245428	-0.476206	-1.325626
9	1	0	-3.970486	-0.795091	-2.061487
10	6	0	-3.427505	0.634544	-0.592990
11	6	0	-4.609439	1.541700	-0.694173
12	1	0	-5.312048	1.192233	-1.444530
13	1	0	-5.124601	1.604900	0.262342
14	1	0	-4.296152	2.550823	-0.955329
15	6	0	0.375994	-0.186492	0.287545
16	6	0	1.225684	-1.027684	-0.431751
17	6	0	0.923136	0.935837	0.924478
18	6	0	2.590739	-0.762756	-0.531144
19	1	0	0.834631	-1.905465	-0.926407
20	6	0	2.278393	1.210738	0.833391
21	1	0	0.292860	1.602063	1.496277
22	6	0	3.120348	0.363328	0.104342
23	1	0	3.216641	-1.431195	-1.098148
24	1	0	2.704207	2.076159	1.318437
25	8	0	-1.678853	-1.433231	1.682584
26	6	0	5.307722	-0.143950	-0.659555
27	1	0	5.023013	-0.189830	-1.707291
28	1	0	6.292025	0.292795	-0.568306
29	1	0	5.309638	-1.143912	-0.234328
30	8	0	4.431743	0.717490	0.069316

1e:
1101bab_POcpent3Me_pOMePh_MP2_6311++2d2p_PCMthf.log

Input orientation:

Center Number	Atomic Number	Atomic Type	Coordinates (Angstroms) X	Y	Z
1	15	0	-1.428337	1.197612	-0.111409
2	6	0	-2.448549	0.076535	-1.132596
3	1	0	-1.854082	-0.403952	-1.907063
4	1	0	-3.233005	0.652217	-1.624037
5	6	0	-2.201322	0.724338	1.479528
6	1	0	-1.453899	0.632550	2.263389
7	1	0	-2.904579	1.502654	1.773904
8	6	0	-2.888272	-0.570943	1.154776
9	1	0	-3.278010	-1.202451	1.941510
10	6	0	-3.018858	-0.904004	-0.141111
11	6	0	-3.688521	-2.141206	-0.640466
12	1	0	-4.065025	-2.745023	0.179751
13	1	0	-4.519212	-1.887851	-1.296567
14	1	0	-2.993388	-2.741500	-1.225317
15	6	0	0.215988	0.458150	-0.093738
16	6	0	1.324489	1.300750	-0.153778
17	6	0	0.421198	-0.926339	-0.006733
18	6	0	2.621872	0.788978	-0.124396
19	1	0	1.174297	2.367322	-0.231045
20	6	0	1.705951	-1.445323	0.024463
21	1	0	-0.419670	-1.603445	0.040039
22	6	0	2.813802	-0.591214	-0.032506
23	1	0	3.456793	1.467553	-0.175576
24	1	0	1.873179	-2.510025	0.089451
25	8	0	-1.406234	2.658976	-0.460396
26	8	0	4.030976	-1.197119	0.003716
27	6	0	5.172027	-0.340362	-0.056700
28	1	0	5.188999	0.343123	0.788059
29	1	0	6.032453	-0.993026	-0.014607
30	1	0	5.184930	0.223334	-0.985708

1e+H2:
1101bba_POcpent3Me_pOMePh_+H2OL_MP2_6311++2d2p_PCMthf.log

Input orientation:

Center Number	Atomic Number	Atomic Type	Coordinates (Angstroms) X	Y	Z
1	15	0	-1.391643	-0.872021	0.675172
2	6	0	-2.262194	0.725995	0.905214
3	1	0	-1.568337	1.550297	0.759589
4	1	0	-2.666121	0.790254	1.910972
5	6	0	-2.219850	-1.292547	-0.896036
6	1	0	-1.571441	-1.809894	-1.593481
7	1	0	-3.047823	-1.952863	-0.642747
8	6	0	-2.744819	0.051183	-1.401146
9	1	0	-3.477427	-0.072999	-2.194931
10	6	0	-3.341934	0.768017	-0.187387
11	6	0	-3.803944	2.181488	-0.493705
12	1	0	-4.570043	2.182508	-1.265460
13	1	0	-4.213523	2.663266	0.390287
14	1	0	-2.966216	2.780495	-0.846512
15	6	0	0.316826	-0.470824	0.243624
16	6	0	1.040263	-1.294253	-0.631371
17	6	0	0.980994	0.600159	0.844309
18	6	0	2.374224	-1.040175	-0.908906
19	1	0	0.568713	-2.145114	-1.100657
20	6	0	2.323412	0.865143	0.577868
21	1	0	0.462804	1.245799	1.538324
22	6	0	3.024247	0.043038	-0.307793
23	1	0	2.930013	-1.670155	-1.587054
24	1	0	2.799489	1.702463	1.059906
25	1	0	-1.923433	0.648119	-1.801754
26	1	0	-4.194424	0.178369	0.153676
27	8	0	-1.513117	-1.875987	1.790909

28	6	0	5.010177	1.308277	-0.026824
29	1	0	6.020851	1.278663	-0.408416
30	1	0	4.545681	2.253947	-0.293162
31	1	0	5.023121	1.195924	1.053934
32	8	0	4.329621	0.214683	-0.644419

1e+H2:
1101bbb_POcpent3Me_pOMePh_+H2OL_MP2_6311++2d2p_PCMthf.log

Input orientation:

Center Number	Atomic Number	Atomic Type	Coordinates (Angstroms) X	Y	Z
1	15	0	-1.297713	1.006515	0.002445
2	6	0	-2.289872	-0.053763	-1.107243
3	1	0	-1.711608	-0.939650	-1.368744
4	1	0	-2.530412	0.481778	-2.020778
5	6	0	-2.107313	0.431121	1.541857
6	1	0	-1.385969	0.205697	2.320061
7	1	0	-2.725595	1.262104	1.876955
8	6	0	-2.980708	-0.755646	1.124205
9	1	0	-3.788452	-0.927263	1.831563
10	6	0	-3.515391	-0.458013	-0.280060
11	6	0	-4.274288	-1.625459	-0.885659
12	1	0	-5.128648	-1.896648	-0.270024
13	1	0	-4.637093	-1.386660	-1.881787
14	1	0	-3.623232	-2.494534	-0.963276
15	6	0	0.389696	0.371728	0.002377
16	6	0	0.675279	-0.980945	0.231688
17	6	0	1.444668	1.254612	-0.215769
18	6	0	1.985276	-1.432771	0.241966
19	1	0	-0.119972	-1.692794	0.404308
20	6	0	2.767653	0.811851	-0.211245
21	1	0	1.230409	2.298409	-0.390671
22	6	0	3.039506	-0.538440	0.019350
23	1	0	2.213797	-2.473156	0.418302
24	1	0	3.560589	1.519675	-0.385670
25	1	0	-2.385553	-1.669065	1.086310
26	1	0	-4.180476	0.404823	-0.209229
27	8	0	-1.356898	2.488401	-0.254969
28	6	0	5.375223	-0.178125	-0.175312
29	1	0	6.271279	-0.779289	-0.114128
30	1	0	5.306546	0.277963	-1.159290
31	1	0	5.400063	0.595547	0.587456
32	8	0	4.287952	-1.077268	0.046541

4e:
1102ba_POcpent2Me_pOMePh_MP2_6311++2d2p_PCMthf.log

Input orientation:

Center Number	Atomic Number	Atomic Type	Coordinates (Angstroms) X	Y	Z
1	15	0	-1.398655	1.207555	-0.205535
2	6	0	-2.429878	0.049814	-1.096527
3	6	0	-2.195741	0.844591	1.396339
4	1	0	-1.480864	0.843159	2.211702
5	1	0	-2.905904	1.651834	1.560542
6	6	0	-2.928353	-0.490069	1.213880
7	1	0	-3.882575	-0.503319	1.736796
8	6	0	-3.133106	-0.735329	-0.262550
9	6	0	-4.043330	-1.840924	-0.681121
10	1	0	-5.048814	-1.658362	-0.306397
11	1	0	-4.083063	-1.944846	-1.759996
12	1	0	-3.711562	-2.783541	-0.249025
13	6	0	0.249938	0.474293	-0.162968
14	6	0	1.368625	1.305774	-0.182761
15	6	0	0.438155	-0.910781	-0.065152
16	6	0	2.658155	0.780505	-0.103721
17	1	0	1.232354	2.373564	-0.268180
18	6	0	1.714699	-1.444937	0.016768

19	1	0	-0.412724	-1.577616	-0.069497
20	6	0	2.832048	-0.601892	0.000392
21	1	0	3.502057	1.449511	-0.126251
22	1	0	1.868787	-2.511331	0.086708
23	1	0	-2.341756	-1.312803	1.624031
24	1	0	-2.449401	-0.052008	-2.170562
25	8	0	-1.363731	2.648600	-0.633490
26	8	0	4.041214	-1.219540	0.083617
27	6	0	5.192411	-0.374670	0.061843
28	1	0	6.043964	-1.035866	0.137957
29	1	0	5.245273	0.184986	-0.868182
30	1	0	5.185612	0.312200	0.904018

11e:

1103baa_POcpent3Me_pOMePh_ikerion_MP2_6311++2d2p_PCMthf.log

Input orientation:

Center Number	Atomic Number	Atomic Type	Coordinates (Angstroms) X	Y	Z
1	15	0	-1.346282	0.639453	-0.188110
2	6	0	-2.413850	-0.423064	-0.927847
3	6	0	-2.143108	0.633158	1.456123
4	1	0	-1.485526	0.116083	2.154880
5	1	0	-2.267971	1.654257	1.811936
6	6	0	-3.432732	-0.107095	1.217557
7	1	0	-4.189979	-0.198473	1.980089
8	6	0	-3.535480	-0.649275	-0.021803
9	6	0	-4.708392	-1.457382	-0.482738
10	1	0	-5.448566	-1.558143	0.305802
11	1	0	-5.181169	-0.993904	-1.346568
12	1	0	-4.391704	-2.452894	-0.788390
13	6	0	0.401426	0.235521	-0.128086
14	6	0	1.413486	1.194501	-0.194179
15	6	0	0.753152	-1.110668	0.041356
16	6	0	2.754400	0.827045	-0.094187
17	1	0	1.163009	2.234341	-0.329232
18	6	0	2.083166	-1.485196	0.141884
19	1	0	-0.021275	-1.863033	0.083515
20	6	0	3.092449	-0.518158	0.075915
21	1	0	3.512247	1.590189	-0.152220
22	1	0	2.361309	-2.520838	0.266774
23	8	0	-1.196325	2.186744	-0.730716
24	1	0	-2.062525	2.511980	-1.005792
25	1	0	-2.344881	-0.825455	-1.923243
26	6	0	5.408717	-0.007675	0.110442
27	1	0	6.332549	-0.559005	0.213037
28	1	0	5.393418	0.506667	-0.846695
29	1	0	5.321444	0.714418	0.917775
30	8	0	4.365642	-0.980948	0.182403

11e+H2:

1103bba_POcpent3Me_pOMePh_ikerion_+H2OL_MP2_6311++2d2p_PCMthf.log

Input orientation:

Center Number	Atomic Number	Atomic Type	Coordinates (Angstroms) X	Y	Z
1	15	0	-1.324670	0.697438	-0.292936
2	6	0	-2.328085	-0.469068	-0.924891
3	6	0	-2.235419	1.002259	1.253169
4	1	0	-1.578801	1.020671	2.117212
5	1	0	-2.710559	1.976653	1.163220
6	6	0	-3.263049	-0.134138	1.282530
7	1	0	-4.136256	0.141063	1.872371
8	6	0	-3.639684	-0.501686	-0.162926
9	6	0	-4.300857	-1.870442	-0.233623
10	1	0	-5.204934	-1.901230	0.372635
11	1	0	-4.572434	-2.123114	-1.255742
12	1	0	-3.613246	-2.631277	0.131656
13	6	0	0.416915	0.242644	-0.193323

14	6	0	1.432150	1.193841	-0.059671
15	6	0	0.767564	-1.114865	-0.205046
16	6	0	2.767588	0.811917	0.056993
17	1	0	1.186947	2.244524	-0.044589
18	6	0	2.093539	-1.504859	-0.105724
19	1	0	-0.011370	-1.856947	-0.296605
20	6	0	3.102098	-0.544601	0.030270
21	1	0	3.523671	1.571835	0.162553
22	1	0	2.368595	-2.548849	-0.124004
23	1	0	-2.809127	-1.014183	1.735929
24	1	0	-4.363887	0.236627	-0.525856
25	8	0	-1.196871	2.278142	-0.818248
26	1	0	-0.747241	2.316340	-1.670311
27	1	0	-2.181673	-0.983994	-1.861058
28	6	0	5.410503	-0.055835	0.274130
29	1	0	6.330226	-0.619854	0.337499
30	1	0	5.445740	0.606632	-0.586571
31	1	0	5.274721	0.526663	1.181397
32	8	0	4.370228	-1.023855	0.129742

11e+H+:

1105baa_POcpent3Me_pOMePh_+H+_MP2_6311++2d2p_PCMthf.log

Input orientation:

Center Number	Atomic Number	Atomic Type	Coordinates (Angstroms) X	Y	Z
1	15	0	-1.280019	-0.330503	0.378588
2	6	0	-2.210598	1.175992	0.060025
3	1	0	-1.717703	1.775186	-0.704093
4	1	0	-2.310451	1.777067	0.960452
5	6	0	-2.190984	-1.418444	-0.732171
6	1	0	-1.682414	-1.485849	-1.692296
7	1	0	-2.276434	-2.417882	-0.315012
8	6	0	-3.502472	-0.675522	-0.825420
9	1	0	-4.375662	-1.174718	-1.216897
10	6	0	-3.527033	0.610600	-0.437233
11	6	0	-4.716533	1.507903	-0.490268
12	1	0	-5.581873	0.986001	-0.884369
13	1	0	-4.954403	1.883556	0.502128
14	1	0	-4.511507	2.370360	-1.120608
15	6	0	0.455704	-0.242870	0.130885
16	6	0	1.162303	-1.381037	-0.287880
17	6	0	1.136993	0.951701	0.379615
18	6	0	2.532920	-1.315138	-0.450034
19	1	0	0.650938	-2.311394	-0.486714
20	6	0	2.515264	1.019033	0.216766
21	1	0	0.604930	1.835865	0.699390
22	6	0	3.218942	-0.118549	-0.197059
23	1	0	3.094087	-2.178051	-0.773249
24	1	0	3.020504	1.949730	0.410279
25	8	0	-1.487312	-0.795263	1.891960
26	1	0	-2.411517	-0.910076	2.153669
27	8	0	4.552549	-0.159963	-0.383792
28	6	0	5.287770	1.040203	-0.114330
29	1	0	5.168362	1.337284	0.923263
30	1	0	6.321716	0.796421	-0.309279
31	1	0	4.968133	1.842345	-0.772619

11e+H+:

1105bab_POcpent3Me_pOMePh_+H+_MP2_6311++2d2p_PCMthf.log

Input orientation:

Center Number	Atomic Number	Atomic Type	Coordinates (Angstroms) X	Y	Z
1	15	0	-1.267824	0.504973	0.218210
2	6	0	-2.297252	-0.100208	-1.131773
3	1	0	-1.834384	-0.972431	-1.592856
4	1	0	-2.412632	0.663108	-1.898074
5	6	0	-2.168178	-0.284749	1.565340
6	1	0	-1.666286	-1.202128	1.868624
7	1	0	-2.201200	0.378748	2.425729
8	6	0	-3.506521	-0.537475	0.919962
9	1	0	-4.357818	-0.800745	1.529210
10	6	0	-3.584818	-0.451896	-0.418650
11	6	0	-4.809406	-0.707632	-1.230157
12	1	0	-5.650245	-0.967347	-0.595892
13	1	0	-5.069749	0.170265	-1.816909
14	1	0	-4.634823	-1.521844	-1.930032
15	6	0	0.456997	0.156567	0.131520
16	6	0	0.890477	-1.170698	0.272120
17	6	0	1.383205	1.173089	-0.106893
18	6	0	2.236782	-1.467231	0.173061
19	1	0	0.190929	-1.972954	0.458352
20	6	0	2.738141	0.875265	-0.203234
21	1	0	1.057244	2.195360	-0.214766
22	6	0	3.169967	-0.448209	-0.064748
23	1	0	2.588719	-2.481402	0.280981
24	1	0	3.436006	1.675002	-0.383066
25	8	0	-1.345070	2.091074	0.321128
26	1	0	-2.238032	2.459189	0.295203
27	8	0	4.457947	-0.840496	-0.141561
28	6	0	5.435099	0.178683	-0.384153
29	1	0	5.434674	0.909400	0.419147
30	1	0	6.386175	-0.332347	-0.414245
31	1	0	5.249826	0.669006	-1.335192

11e-H+:

1105bba_POcpent3Me_pMePh_-H+A_MP2_6311++2d2p_PCMthf.log

Input orientation:

Center Number	Atomic Number	Atomic Type	Coordinates (Angstroms) X	Y	Z
1	15	0	1.487882	1.230662	0.240982
2	6	0	2.556260	0.126178	1.038360
3	6	0	2.143405	0.871433	-1.447304
4	1	0	1.334401	0.839735	-2.174157
5	1	0	2.795789	1.711038	-1.710391
6	6	0	2.909993	-0.413878	-1.272509
7	1	0	3.415633	-0.910800	-2.088835
8	6	0	3.129710	-0.732898	0.042583
9	6	0	3.949879	-1.923599	0.448117
10	1	0	4.316804	-2.463691	-0.421527
11	1	0	4.803085	-1.617957	1.051642
12	1	0	3.361909	-2.611226	1.055289
13	6	0	-0.203614	0.527603	0.190292
14	6	0	-1.334785	1.332882	0.063776
15	6	0	-0.382321	-0.861515	0.233956
16	6	0	-2.618920	0.785358	-0.005899
17	1	0	-1.215541	2.406175	0.035557
18	6	0	-1.649590	-1.423963	0.156962
19	1	0	0.487840	-1.495679	0.338058
20	6	0	-2.774674	-0.601550	0.038443
21	1	0	-3.470678	1.440416	-0.088900
22	1	0	-1.790751	-2.494562	0.193520
23	8	0	1.356107	2.698588	0.618325
24	1	0	2.474355	-0.166447	2.074471
25	6	0	-5.133737	-0.414435	-0.135019
26	1	0	-5.980933	-1.085621	-0.168756

27	1	0	-5.225799	0.246374	0.723536
28	1	0	-5.100511	0.177393	-1.046582
29	8	0	-3.981289	-1.244523	-0.021072

11e-H+:

1105bbb_POcpent3Me_pMePh_-H+A_MP2_6311++2d2p_PCMthf.log

Input orientation:

Center Number	Atomic Number	Atomic Type	Coordinates (Angstroms) X	Y	Z
1	15	0	-1.488008	1.233843	-0.225391
2	6	0	-2.558255	0.136774	-1.030553
3	6	0	-2.141105	0.860528	1.460687
4	1	0	-1.331271	0.822838	2.186321
5	1	0	-2.793092	1.698043	1.731389
6	6	0	-2.908004	-0.423210	1.276208
7	1	0	-3.412093	-0.927219	2.089136
8	6	0	-3.129982	-0.730895	-0.041213
9	6	0	-3.950699	-1.918149	-0.455598
10	1	0	-4.316200	-2.465692	0.409985
11	1	0	-4.804882	-1.607471	-1.055148
12	1	0	-3.363596	-2.600520	-1.069512
13	6	0	0.203243	0.529625	-0.183783
14	6	0	1.334908	1.333438	-0.052661
15	6	0	0.381462	-0.859121	-0.239799
16	6	0	2.619062	0.785008	0.009579
17	1	0	1.216033	2.406481	-0.014862
18	6	0	1.648713	-1.422535	-0.170308
19	1	0	-0.489052	-1.492169	-0.347631
20	6	0	2.774306	-0.601489	-0.047089
21	1	0	3.471151	1.439146	0.096376
22	1	0	1.789519	-2.492819	-0.216351
23	8	0	-1.356078	2.704896	-0.590331
24	1	0	-2.477575	-0.147310	-2.069147
25	6	0	5.133672	-0.416530	0.123773
26	1	0	5.980743	-1.088193	0.150476
27	1	0	5.224607	0.251594	-0.729229
28	1	0	5.101993	0.167495	1.040415
29	8	0	3.980835	-1.245308	0.004472

11e+H+:

1106ba_POcpent3Me_pOMePh_+H+_+H2OL_MP2_6311++2d2p_PCMthf.log

Input orientation:

Center Number	Atomic Number	Atomic Type	Coordinates (Angstroms) X	Y	Z
1	15	0	-1.324692	0.697568	-0.293079
2	6	0	-2.328134	-0.468948	-0.924975
3	6	0	-2.235442	1.002471	1.253012
4	1	0	-1.578797	1.021016	2.117031
5	1	0	-2.710656	1.976821	1.162983
6	6	0	-3.262969	-0.134004	1.282499
7	1	0	-4.136165	0.141155	1.872373
8	6	0	-3.639664	-0.501674	-0.162914
9	6	0	-4.300674	-1.870517	-0.233485
10	1	0	-5.204705	-1.901381	0.372838
11	1	0	-4.572292	-2.123292	-1.255568
12	1	0	-3.612945	-2.631242	0.131796
13	6	0	0.416885	0.242724	-0.193362
14	6	0	1.432172	1.193898	-0.059790
15	6	0	0.767445	-1.114837	-0.204879
16	6	0	2.767565	0.811914	0.057018
17	1	0	1.187029	2.244596	-0.044868
18	6	0	2.093397	-1.504870	-0.105401
19	1	0	-0.011535	-1.856875	-0.296388
20	6	0	3.101994	-0.544626	0.030517
21	1	0	3.523697	1.571794	0.162497
22	1	0	2.368420	-2.548871	-0.123508
23	1	0	-2.808945	-1.013978	1.735936

24	1	0	-4.363983	0.236525	-0.525840
25	8	0	-1.196825	2.278235	-0.818475
26	1	0	-2.181741	-0.983941	-1.861107
27	1	0	-0.747118	2.316371	-1.670500
28	6	0	5.410420	-0.055936	0.274334
29	1	0	5.274672	0.526725	1.181502
30	1	0	6.330122	-0.619977	0.337798
31	1	0	5.445698	0.606396	-0.586471
32	8	0	4.370102	-1.023917	0.130097

TS(1e->11e):

1109ba_POcpent3Me_pMePh_ikerion_TSfix_HO_MP2_6311++2d2p_PCMthf.log

Input orientation:

Center Number	Atomic Number	Atomic Type	Coordinates (Angstroms) X	Y	Z
1	15	0	-1.289138	0.515926	0.255945
2	6	0	-2.441731	0.278426	-1.042656
3	6	0	-2.139826	-0.440442	1.541672
4	1	0	-1.571872	-1.337365	1.786924
5	1	0	-2.218672	0.162614	2.444881
6	6	0	-3.464119	-0.739910	0.887946
7	1	0	-4.277260	-1.165435	1.457358
8	6	0	-3.577346	-0.425522	-0.426041
9	6	0	-4.809287	-0.688435	-1.234659
10	1	0	-5.570414	-1.188576	-0.642922
11	1	0	-5.221325	0.242106	-1.619341
12	1	0	-4.573398	-1.313128	-2.094127
13	6	0	0.448579	0.137008	0.140982
14	6	0	1.382548	1.165800	0.018105
15	6	0	0.889633	-1.194452	0.140210
16	6	0	2.742017	0.883214	-0.099783
17	1	0	1.048591	2.192079	0.019779
18	6	0	2.238850	-1.482054	0.025461
19	1	0	0.183930	-2.008836	0.219702
20	6	0	3.173453	-0.445676	-0.095067
21	1	0	3.440946	1.697564	-0.189727
22	1	0	2.590321	-2.502678	0.023239
23	8	0	-1.519792	2.080643	0.355026
24	1	0	-2.288818	1.772173	-0.542439
25	1	0	-2.165315	0.074493	-2.067414
26	6	0	5.439434	0.212607	-0.329782
27	1	0	6.396481	-0.283713	-0.403417
28	1	0	5.260023	0.798872	-1.226803
29	1	0	5.428118	0.860258	0.542568
30	8	0	4.470597	-0.830240	-0.201709

TS(11e->4e):

1110ba_POcpent3Me_pOMePh_b3lyp631dp_SCAN_HOvege_MP2_631++dp_PCMthf_TS2.log

Input orientation:

Center Number	Atomic Number	Atomic Type	Coordinates (Angstroms) X	Y	Z
1	15	0	-1.389856	0.590585	-0.260189
2	6	0	-2.456151	-0.581405	-0.824512
3	1	0	-2.424703	-1.078304	-1.781049
4	1	0	-2.183424	2.423137	-1.191157
5	6	0	-2.149804	0.778355	1.392933
6	1	0	-1.488311	0.298867	2.121678
7	1	0	-2.228672	1.834291	1.659426
8	6	0	-3.467762	0.060162	1.255232
9	1	0	-4.214572	0.090850	2.036305
10	6	0	-3.590441	-0.647004	0.096985
11	6	0	-4.793771	-1.488327	-0.247891
12	1	0	-4.951541	-2.269560	0.495399
13	1	0	-5.697595	-0.880371	-0.285805
14	1	0	-4.671327	-1.966948	-1.217299
15	8	0	-1.291315	2.096374	-0.984476
16	6	0	0.370624	0.239495	-0.228892

17	6	0	0.792139	-1.101180	-0.214405
18	6	0	1.331654	1.255881	-0.153451
19	6	0	2.143490	-1.413531	-0.135736
20	1	0	0.057028	-1.893925	-0.268544
21	6	0	2.692186	0.950233	-0.069778
22	1	0	1.026072	2.292859	-0.168792
23	6	0	3.099429	-0.390131	-0.062031
24	1	0	2.479026	-2.442076	-0.133444
25	1	0	3.408193	1.756380	-0.015774
26	8	0	4.400335	-0.802672	0.012153
27	6	0	5.404883	0.215969	0.099456
28	1	0	6.347451	-0.315771	0.155812
29	1	0	5.390097	0.850620	-0.785642
30	1	0	5.265712	0.819790	0.995235

TS(11e->4e):

1110ba_POcpent3Me_pOMePh_b3lyp631dp_SCAN_HOvege_MP2_6311++2d2p_PCMthf_TS2.log

Input orientation:

Center Number	Atomic Number	Atomic Type	Coordinates (Angstroms) X	Y	Z
1	15	0	1.307740	-0.039053	-0.498300
2	6	0	2.156259	0.873028	0.734776
3	1	0	1.898224	1.833149	1.147759
4	1	0	2.952151	-0.007704	-1.586747
5	6	0	2.285606	-1.531467	-0.259138
6	1	0	2.112153	-1.999747	0.708761
7	1	0	2.145074	-2.229862	-1.077591
8	6	0	3.596852	-0.717879	-0.321996
9	1	0	4.533732	-1.258030	-0.312471
10	6	0	3.455256	0.424470	0.540491
11	6	0	4.652418	1.133993	1.091478
12	1	0	5.162714	0.499886	1.813534
13	1	0	5.360486	1.347266	0.294255
14	1	0	4.381009	2.065999	1.576444
15	8	0	1.860393	0.348289	-1.936439
16	6	0	-0.460411	-0.036114	-0.491607
17	6	0	-1.179592	-0.433846	-1.627685
18	6	0	-1.144226	0.334310	0.666973
19	6	0	-2.562482	-0.459547	-1.594239
20	1	0	-0.658144	-0.711545	-2.531240
21	6	0	-2.536005	0.308018	0.705766
22	1	0	-0.595396	0.648050	1.542962
23	6	0	-3.248420	-0.091814	-0.428685
24	1	0	-3.133266	-0.759498	-2.459805
25	1	0	-3.042527	0.602118	1.609312
26	8	0	-4.600470	-0.150208	-0.499110
27	6	0	-5.323135	0.222256	0.677085
28	1	0	-6.368093	0.110059	0.426178
29	1	0	-5.072568	-0.431904	1.507490
30	1	0	-5.118725	1.255055	0.945020

12a:

1350aa_POcpent3Me_Ph_dimer_kiind_MP2_6311++2d2p_PCMthf.log

orientation:

Center Number	Atomic Number	Atomic Type	Coordinates (Angstroms) X	Y	Z
1	15	0	2.487415	0.279893	-0.256628
2	6	0	1.955514	0.144097	1.510957
3	1	0	2.284761	-0.805026	1.949168
4	1	0	0.857340	0.161071	1.548473
5	6	0	3.270703	1.949896	-0.073633
6	1	0	4.290958	1.961917	-0.473875
7	1	0	2.690176	2.677883	-0.654941
8	6	0	3.223026	2.245919	1.412861
9	6	0	2.578580	1.346977	2.174256
10	8	0	1.418784	0.144935	-1.318774
11	6	0	3.846721	-0.917439	-0.501105
12	6	0	4.940034	-0.988175	0.376484
13	6	0	3.784894	-1.785041	-1.598295
14	6	0	5.957116	-1.916210	0.154442
15	1	0	4.998638	-0.321786	1.233099
16	6	0	4.804386	-2.713646	-1.818119
17	1	0	2.934835	-1.725771	-2.270417
18	6	0	5.889970	-2.779508	-0.943093
19	1	0	6.800627	-1.966949	0.836456
20	1	0	4.749962	-3.384147	-2.670631
21	1	0	6.682897	-3.501646	-1.113938
22	1	0	2.476753	1.479310	3.249237
23	15	0	-2.487711	0.280169	0.256625
24	6	0	-1.955779	0.144841	-1.510989
25	1	0	-2.285002	-0.804184	-1.949435
26	1	0	-0.857606	0.161810	-1.548499
27	6	0	-3.271741	1.949853	0.073896
28	1	0	-4.292140	1.961256	0.473792
29	1	0	-2.691768	2.677927	0.655646
30	6	0	-3.223715	2.246393	-1.412482
31	6	0	-2.578888	1.347845	-2.174018
32	8	0	-1.418968	0.145615	1.318709
33	6	0	-3.846440	-0.917833	0.500979
34	6	0	-4.939798	-0.988841	-0.376528
35	6	0	-3.784115	-1.785680	1.597946
36	6	0	-5.956445	-1.917386	-0.154626
37	1	0	-4.998778	-0.322270	-1.232979
38	6	0	-4.803171	-2.714798	1.817631
39	1	0	-2.934015	-1.726195	2.269997
40	6	0	-5.888808	-2.780926	0.942689
41	1	0	-6.799996	-1.968335	-0.836575
42	1	0	-4.748368	-3.385490	2.669968
43	1	0	-6.681395	-3.503462	1.113428
44	1	0	-2.476816	1.480568	-3.248927
45	6	0	-3.863170	3.505549	-1.922116
46	1	0	-3.413441	4.390618	-1.454599
47	1	0	-3.756422	3.599463	-3.006358
48	1	0	-4.932395	3.532051	-1.677051
49	6	0	3.862357	3.505029	1.922760
50	1	0	3.412330	4.390167	1.455661
51	1	0	3.755867	3.598551	3.007061
52	1	0	4.931514	3.531830	1.677430

13a:

1355aa_POcpent3Me_Ph_dimer_zwitter_kiind_MP2_6311++2d2p_PCMthf.log

orientation:

Center Number	Atomic Number	Atomic Type	Coordinates (Angstroms) X	Y	Z
1	15	0	2.227119	-0.391347	0.031711
2	6	0	1.893394	-2.197937	-0.221047
3	1	0	2.843104	-2.728072	-0.374599
4	1	0	1.302278	-2.324587	-1.136206
5	6	0	1.746243	-0.347786	1.655661
6	6	0	1.136873	-1.628043	1.999170
7	6	0	1.181233	-2.604328	1.051102
8	8	0	1.466594	0.468169	-1.133467
9	1	0	0.814288	-3.614415	1.195840
10	15	0	-2.319488	-0.123442	-0.702943
11	6	0	-2.505903	-1.370513	0.646296
12	1	0	-2.670582	-0.880282	1.612952
13	1	0	-1.569724	-1.939722	0.732280
14	6	0	-3.816678	-0.645544	-1.648514
15	1	0	-4.560567	0.158037	-1.702002
16	1	0	-3.518299	-0.872814	-2.679129
17	6	0	-4.336321	-1.867452	-0.913966
18	6	0	-3.681938	-2.206943	0.207716
19	8	0	-1.027539	-0.205529	-1.504568
20	1	0	-3.975695	-3.068565	0.802518
21	6	0	-5.516269	-2.601853	-1.480467
22	1	0	-5.300814	-2.968517	-2.491778
23	1	0	-5.793699	-3.456104	-0.857121
24	1	0	-6.387240	-1.940147	-1.566188
25	6	0	0.526949	-1.824950	3.362048
26	1	0	0.137601	-2.839462	3.487405
27	1	0	1.266600	-1.641630	4.151447
28	1	0	-0.293939	-1.117455	3.533307
29	1	0	1.762974	0.511325	2.312258
30	1	0	0.501688	0.207973	-1.228185
31	6	0	-2.601899	1.536737	-0.012311
32	6	0	-3.795849	1.849868	0.656968
33	6	0	-1.602898	2.510319	-0.141959
34	6	0	-3.986393	3.125032	1.186894
35	1	0	-4.578012	1.103446	0.768840
36	6	0	-1.798170	3.785765	0.391590
37	1	0	-0.676298	2.266223	-0.650869
38	6	0	-2.987498	4.093779	1.053688
39	1	0	-4.911679	3.362801	1.702616
40	1	0	-1.020611	4.536533	0.289706
41	1	0	-3.137656	5.086584	1.466996
42	6	0	3.936154	0.125727	-0.338864
43	6	0	4.890615	0.088837	0.687948
44	6	0	4.329900	0.516279	-1.629245
45	6	0	6.216844	0.440305	0.432295
46	1	0	4.580042	-0.209069	1.684891
47	6	0	5.657741	0.859502	-1.883547
48	1	0	3.593361	0.561557	-2.423497
49	6	0	6.602137	0.822638	-0.854141
50	1	0	6.946961	0.416213	1.235840
51	1	0	5.954709	1.160896	-2.883808
52	1	0	7.634711	1.093348	-1.054653

1a+4a:

1360aa_POcpent3Me_Ph_heterodimer_kiind_MP2_6311++2d2p_PCMthf.log

orientation:

Center Number	Atomic Number	Atomic Type	Coordinates (Angstroms) X	Y	Z
1	15	0	2.403103	0.269940	-0.288292
2	6	0	1.861723	0.189755	1.479171
3	1	0	2.170574	-0.753767	1.943378
4	1	0	0.763645	0.230779	1.512025
5	6	0	3.188511	1.943718	-0.151500
6	1	0	4.204879	1.945822	-0.561627
7	1	0	2.602724	2.658894	-0.743471
8	6	0	3.154373	2.272904	1.328534
9	6	0	2.504524	1.398056	2.113135
10	8	0	1.341595	0.101344	-1.353102
11	6	0	3.764357	-0.933515	-0.489766
12	6	0	4.846742	-0.987834	0.402534
13	6	0	3.716582	-1.821128	-1.571636
14	6	0	5.866740	-1.919313	0.210028
15	1	0	4.894431	-0.305574	1.247153
16	6	0	4.739000	-2.752988	-1.761913
17	1	0	2.874970	-1.774846	-2.255296
18	6	0	5.813669	-2.802354	-0.872460
19	1	0	6.701648	-1.957213	0.903382
20	1	0	4.695391	-3.438871	-2.602738
21	1	0	6.608963	-3.526967	-1.020324
22	1	0	2.410233	1.554959	3.185483
23	15	0	-2.572646	0.337743	0.259265
24	6	0	-2.012594	0.402750	-1.501146
25	1	0	-2.548872	-0.358088	-2.074679
26	1	0	-0.942526	0.178939	-1.543038
27	6	0	-3.504868	1.872332	0.136783
28	6	0	-3.275191	2.515801	-1.022240
29	6	0	-2.346975	1.824944	-2.005679
30	8	0	-1.495306	0.267750	1.322736
31	6	0	-3.758728	-1.042702	0.447661
32	6	0	-4.928845	-1.121892	-0.322871
33	6	0	-3.483305	-2.046513	1.385055
34	6	0	-5.806639	-2.193440	-0.160351
35	1	0	-5.160118	-0.343236	-1.044826
36	6	0	-4.363111	-3.118677	1.547133
37	1	0	-2.580205	-1.975397	1.982681
38	6	0	-5.523571	-3.193338	0.774629
39	1	0	-6.711175	-2.247703	-0.758874
40	1	0	-4.142767	-3.893552	2.275477
41	1	0	-6.208120	-4.026967	0.901178
42	1	0	-1.437097	2.428549	-2.118763
43	6	0	-3.844456	3.853083	-1.394503
44	1	0	-3.038675	4.565051	-1.611365
45	1	0	-4.445436	3.774715	-2.308706
46	1	0	-4.469916	4.267426	-0.600767
47	6	0	3.810282	3.535762	1.807117
48	1	0	3.365846	4.415563	1.324785
49	1	0	3.712328	3.653117	2.889914
50	1	0	4.877914	3.546495	1.553967
51	1	0	-4.124289	2.266474	0.936093
52	1	0	-2.810836	1.794935	-2.998978

12h:

2350aa_POcpent3Me_Et_dimer_kiind_MP2_6311++2d2p_PCMthf.log

Input orientation:

Center Number	Atomic Number	Atomic Type	Coordinates (Angstroms) X	Y	Z
1	15	0	2.369788	0.369753	-0.676471
2	6	0	2.067573	-1.372710	-0.138468
3	1	0	2.370789	-2.086959	-0.913439
4	1	0	0.990946	-1.514413	0.031183
5	6	0	3.173635	0.896683	0.907344
6	1	0	4.094227	1.466823	0.734175
7	1	0	2.488847	1.559548	1.451833
8	6	0	3.424724	-0.397374	1.658770
9	6	0	2.877039	-1.502517	1.128276
10	8	0	1.173786	1.179466	-1.133989
11	1	0	2.988886	-2.476764	1.599285
12	15	0	-2.503103	-0.826976	0.033241
13	6	0	-2.228236	0.732805	-0.922339
14	1	0	-2.611326	0.652146	-1.946393
15	1	0	-1.148246	0.923365	-0.993607
16	6	0	-3.173434	0.015161	1.539479
17	1	0	-4.084666	-0.473947	1.904392
18	1	0	-2.432513	-0.049132	2.346781
19	6	0	-3.415169	1.448200	1.104442
20	6	0	-2.948430	1.780849	-0.109733
21	8	0	-1.310423	-1.739981	0.230771
22	1	0	-3.061325	2.786910	-0.508046
23	6	0	-3.949230	-1.667856	-0.728376
24	6	0	-3.639530	-2.333809	-2.074587
25	1	0	-4.748854	-0.923686	-0.827403
26	1	0	-4.294133	-2.413081	-0.001936
27	1	0	-4.527111	-2.842765	-2.461965
28	1	0	-3.323817	-1.601228	-2.824031
29	1	0	-2.841846	-3.074423	-1.971853
30	6	0	3.709002	0.271167	-1.931035
31	6	0	4.197408	1.638134	-2.424893
32	1	0	3.307210	-0.317144	-2.764273
33	1	0	4.529043	-0.311622	-1.494282
34	1	0	4.969068	1.515315	-3.190610
35	1	0	3.375628	2.212990	-2.860054
36	1	0	4.628668	2.230833	-1.611799
37	6	0	-4.124737	2.384017	2.039617
38	1	0	-3.580802	2.479389	2.987987
39	1	0	-4.231401	3.381835	1.604897
40	1	0	-5.125000	2.008334	2.290102
41	6	0	4.231249	-0.359796	2.924514
42	1	0	4.335426	-1.355928	3.363726
43	1	0	5.235581	0.042754	2.740957
44	1	0	3.763614	0.296342	3.669624

13h:

2355aa_POcpent3Me_Et_dimer_zwitter_kiind_MP2_6311++2d2p_PCMthf.log

Input orientation:

Center Number	Atomic Number	Atomic Type	Coordinates (Angstroms) X	Y	Z
1	15	0	-2.588976	-0.057763	-0.446329
2	6	0	-2.101954	1.295126	-1.611569
3	1	0	-2.990810	1.638136	-2.159339
4	1	0	-1.403240	0.896088	-2.357814
5	6	0	-2.235998	0.794790	0.979928
6	6	0	-1.616771	2.065220	0.624176
7	6	0	-1.510315	2.351758	-0.703208
8	8	0	-1.829032	-1.460777	-0.804324
9	1	0	-1.074150	3.261253	-1.100906
10	15	0	1.881150	-0.928756	-0.058050
11	6	0	1.871948	0.786419	0.625261
12	1	0	1.687150	0.784791	1.706244
13	1	0	1.052585	1.354136	0.163101
14	6	0	3.568141	-0.831649	-0.800312
15	1	0	4.219697	-1.629993	-0.424288
16	1	0	3.490985	-0.968703	-1.885965
17	6	0	4.077716	0.548856	-0.431325
18	6	0	3.235694	1.324704	0.268595
19	8	0	0.771412	-1.254956	-1.053018
20	1	0	3.508323	2.332195	0.574069
21	6	0	1.961086	-2.109724	1.339475
22	6	0	0.639533	-2.235423	2.109856
23	1	0	2.777798	-1.787913	1.996559
24	1	0	2.253669	-3.076695	0.914197
25	1	0	0.741353	-2.965205	2.918028
26	1	0	0.338625	-1.281643	2.553740
27	1	0	-0.169281	-2.563830	1.452216
28	6	0	-4.311412	-0.627011	-0.726980
29	6	0	-4.812412	-1.592540	0.355790
30	1	0	-4.357119	-1.095195	-1.716400
31	1	0	-4.934329	0.272785	-0.755787
32	1	0	-5.853423	-1.871352	0.166959
33	1	0	-4.215207	-2.507995	0.376950
34	1	0	-4.765022	-1.129440	1.346702
35	6	0	5.454855	0.952936	-0.871351
36	1	0	5.544968	0.913967	-1.964165
37	1	0	5.698419	1.966893	-0.542988
38	1	0	6.213289	0.268914	-0.470211
39	6	0	-1.146868	2.999058	1.709083
40	1	0	-0.710086	3.912427	1.294906
41	1	0	-1.978242	3.285040	2.365636
42	1	0	-0.396294	2.517725	2.348399
43	1	0	-2.381818	0.454592	1.995841
44	1	0	-0.830067	-1.365471	-0.830059

1h+4h:
2360aa_POcpent3Me_Et_heterodimer_kiind_MP2_6311++2d2p_PCMthf.log

Input orientation:

Center Number	Atomic Number	Atomic Type	Coordinates (Angstroms) X	Y	Z
1	15	0	2.456859	0.390169	-0.668133
2	6	0	2.126142	-1.306908	-0.018595
3	1	0	2.643988	-2.039616	-0.644670
4	1	0	1.052428	-1.511410	-0.071782
5	6	0	3.488906	0.854132	0.735464
6	6	0	3.473129	-0.075833	1.706751
7	6	0	2.661485	-1.329507	1.432659
8	8	0	1.250780	1.271251	-0.940211
9	1	0	1.838928	-1.386617	2.157296
10	15	0	-2.417798	-0.919533	-0.061393
11	6	0	-2.125149	0.738688	-0.826072
12	1	0	-2.480061	0.774341	-1.862719
13	1	0	-1.044334	0.938881	-0.845705
14	6	0	-3.105223	-0.253189	1.522868
15	1	0	-4.014308	-0.786772	1.825269
16	1	0	-2.369083	-0.398439	2.324056
17	6	0	-3.356399	1.216686	1.243503
18	6	0	-2.872521	1.684749	0.081790
19	8	0	-1.231959	-1.856945	0.042744
20	1	0	-2.990101	2.727204	-0.206215
21	6	0	-3.858655	-1.657599	-0.931837
22	6	0	-3.536088	-2.164767	-2.342624
23	1	0	-4.653318	-0.901978	-0.952821
24	1	0	-4.215641	-2.479901	-0.300620
25	1	0	-4.420898	-2.621385	-2.795973
26	1	0	-3.209549	-1.351812	-2.998733
27	1	0	-2.742080	-2.915980	-2.317640
28	6	0	3.523298	0.211174	-2.155743
29	6	0	4.021022	1.549987	-2.714596
30	1	0	2.922422	-0.318061	-2.905462
31	1	0	4.362558	-0.443736	-1.894690
32	1	0	4.605288	1.393449	-3.626410
33	1	0	3.183207	2.209142	-2.957521
34	1	0	4.661412	2.069786	-1.994648
35	6	0	-4.092671	2.037564	2.262275
36	1	0	-3.565264	2.035225	3.224678
37	1	0	-4.204555	3.075563	1.936616
38	1	0	-5.092123	1.626364	2.453126
39	6	0	4.175448	0.042984	3.027817
40	1	0	3.457870	-0.044607	3.852918
41	1	0	4.897670	-0.772710	3.155530
42	1	0	4.704031	0.993738	3.127162
43	1	0	4.006008	1.804311	0.823508
44	1	0	3.279213	-2.217622	1.613610

TS(17a->18a):
1342af_POcpent3Me_Ph+H+_MsO_B_MP2_6311++2d2p_PCMthf_TS_HO1_uj.log

orientation:

Center Number	Atomic Number	Atomic Type	Coordinates (Angstroms) X	Y	Z
1	15	0	-1.247033	-1.093821	0.035557
2	6	0	-0.170683	-0.146655	-1.164527
3	1	0	-0.641408	0.173378	-2.096279
4	1	0	0.660743	-0.823420	-1.418501
5	6	0	-0.277617	-0.509289	1.502246
6	1	0	-0.926636	-0.291057	2.352734
7	1	0	0.388140	-1.328105	1.789915
8	6	0	0.519547	0.722751	1.033720
9	1	0	1.659856	0.489967	1.160845
10	6	0	0.420200	0.957789	-0.381702
11	6	0	0.742988	2.257999	-0.999017
12	1	0	1.356819	2.885921	-0.351685
13	1	0	1.210446	2.128753	-1.976739
14	1	0	-0.218916	2.770438	-1.163418
15	8	0	-1.348184	-2.573581	-0.206559
16	8	0	2.773787	0.035971	-1.063680
17	8	0	4.528268	-1.481655	-0.124956
18	6	0	5.017146	1.113200	-0.166730
19	8	0	3.188093	-0.002681	1.376104
20	16	0	3.801466	-0.202893	0.003005
21	1	0	5.483811	1.026332	-1.148879
22	1	0	4.514195	2.076226	-0.071275
23	1	0	5.766565	0.998418	0.617323
24	1	0	0.408839	1.635089	1.628675
25	6	0	-2.873726	-0.272782	0.058487
26	6	0	-3.960200	-0.976738	-0.480900
27	6	0	-3.074136	1.017043	0.576083
28	6	0	-5.228360	-0.395634	-0.509234
29	1	0	-3.805801	-1.979648	-0.865189
30	6	0	-4.343542	1.593968	0.545889
31	1	0	-2.253715	1.577865	1.014129
32	6	0	-5.420219	0.889345	0.001429
33	1	0	-6.065056	-0.947592	-0.926034
34	1	0	-4.492471	2.589980	0.951022
35	1	0	-6.407474	1.340635	-0.019805

TS(18a->19a):
1342cc_POcpent3Me_Et+H+_MsO_B_MP2_6311++2d2p_PCMthf_TS_HO2_uj2.log

orientation:

Center Number	Atomic Number	Atomic Type	Coordinates (Angstroms) X	Y	Z
1	15	0	-0.953886	-0.915543	0.015633
2	6	0	0.167334	0.344326	-0.755039
3	1	0	-0.017830	0.708125	-1.767111
4	1	0	1.183982	-0.295334	-0.858534
5	6	0	-0.326483	-0.506403	1.704805
6	1	0	-1.071457	-0.691038	2.480122
7	1	0	0.529147	-1.165328	1.881200
8	6	0	0.121685	0.961151	1.637838
9	1	0	0.943734	1.197307	2.317941
10	6	0	0.491952	1.321541	0.230566
11	6	0	0.916552	2.703725	-0.096509
12	1	0	1.545246	3.123879	0.689567
13	1	0	1.421362	2.759170	-1.061630
14	1	0	0.006952	3.319675	-0.160305
15	8	0	-0.822202	-2.343304	-0.435461
16	8	0	2.812305	0.719731	0.748667
17	8	0	2.502281	-1.022606	-0.974469
18	6	0	4.599508	0.568880	-1.204035
19	8	0	4.350339	-1.253281	0.690740
20	16	0	3.513250	-0.318362	-0.079993
21	1	0	4.004689	1.261619	-1.800117
22	1	0	5.335828	1.113137	-0.611223
23	1	0	5.096090	-0.156881	-1.849091

24	1	0	-0.703690	1.639255	1.906046
25	6	0	-2.664810	-0.300819	-0.141982
26	6	0	-2.989434	1.043921	-0.373145
27	6	0	-3.697475	-1.242721	-0.005546
28	6	0	-4.324511	1.442974	-0.450391
29	1	0	-2.211469	1.787458	-0.513064
30	6	0	-5.030384	-0.840939	-0.081684
31	1	0	-3.450207	-2.288261	0.146783
32	6	0	-5.345143	0.502170	-0.301208
33	1	0	-4.565386	2.485360	-0.634356
34	1	0	-5.821916	-1.576229	0.024839
35	1	0	-6.383315	0.813856	-0.363527

TS(12a->13a):
1354aa_POcpent3Me_Ph_dimer_zwitter_kiind_MP2_6311++2d2p_TSvissza.log

orientation:

Center Number	Atomic Number	Atomic Type	Coordinates (Angstroms) X	Y	Z
1	15	0	2.554790	0.364392	0.090004
2	6	0	2.408645	0.550690	1.881713
3	1	0	3.377538	0.676368	2.359068
4	1	0	1.925098	-0.314996	2.324368
5	6	0	1.931405	1.909364	-0.388318
6	6	0	1.351271	2.490101	0.849116
7	6	0	1.541975	1.812806	1.985147
8	8	0	1.391013	-0.449549	-0.560661
9	1	0	1.109317	2.083466	2.926656
10	15	0	-2.166275	0.121034	-0.021278
11	6	0	-2.213207	0.209171	1.799708
12	1	0	-3.123071	0.672206	2.164956
13	1	0	-1.369187	0.802015	2.142043
14	6	0	-1.567621	-1.595481	-0.092142
15	1	0	-2.097575	-2.190664	-0.827937
16	1	0	-0.511096	-1.577925	-0.343736
17	6	0	-1.762034	-2.133217	1.322648
18	6	0	-2.077453	-1.239867	2.246366
19	8	0	-1.394621	1.164026	-0.743608
20	1	0	-2.209351	-1.494597	3.280109
21	6	0	-1.543409	-3.601837	1.568866
22	1	0	-0.531205	-3.880858	1.291648
23	1	0	-1.698318	-3.855110	2.610796
24	1	0	-2.223364	-4.194306	0.963631
25	6	0	0.461280	3.695987	0.710507
26	1	0	0.131757	4.063775	1.675027
27	1	0	0.975876	4.496071	0.188504
28	1	0	-0.397700	3.393920	0.121634
29	1	0	2.316758	2.551886	-1.155585
30	1	0	0.987094	0.696140	-0.850750
31	6	0	-3.881906	0.092371	-0.569191
32	6	0	-4.836951	-0.776024	-0.046972
33	6	0	-4.249590	0.988839	-1.564296
34	6	0	-6.136192	-0.744992	-0.515581
35	1	0	-4.567242	-1.471862	0.722838
36	6	0	-5.552205	1.018146	-2.032961
37	1	0	-3.507225	1.652684	-1.958274
38	6	0	-6.494338	0.152521	-1.509610
39	1	0	-6.867354	-1.415614	-0.109527
40	1	0	-5.827285	1.712949	-2.801351
41	1	0	-7.503293	0.174447	-1.871645
42	6	0	4.134290	-0.268506	-0.447841
43	6	0	5.327397	0.333402	-0.056510
44	6	0	4.167051	-1.375234	-1.288186
45	6	0	6.535253	-0.171071	-0.495213
46	1	0	5.310662	1.197601	0.578995
47	6	0	5.379677	-1.877247	-1.727055
48	1	0	3.244300	-1.827184	-1.589696
49	6	0	6.560834	-1.277654	-1.330056
50	1	0	7.451228	0.296112	-0.193689
51	1	0	5.400042	-2.730436	-2.374981
52	1	0	7.499690	-1.666906	-1.670690

2350aa_POcpent3Me_Et_dimer_kiind_MP2_6311++2d2p_.log

orientation:

Center Number	Atomic Number	Atomic Type	Coordinates (Angstroms) X	Y	Z
1	15	0	2.177461	0.316059	-0.621423
2	6	0	2.005389	-1.467744	-0.338664
3	1	0	2.279936	-2.054653	-1.209623
4	1	0	0.970163	-1.689176	-0.088014
5	6	0	2.858718	0.676334	1.030604
6	1	0	3.655452	1.412764	1.011443
7	1	0	2.072490	1.057193	1.677837
8	6	0	3.353199	-0.680597	1.529344
9	6	0	2.930117	-1.728207	0.841620
10	8	0	0.989270	1.094218	-1.056138
11	1	0	3.188137	-2.735331	1.105301
12	15	0	-2.278174	-0.716698	0.057114
13	6	0	-2.147857	0.674479	-1.100507
14	1	0	-2.508439	0.430852	-2.094295
15	1	0	-1.104361	0.971799	-1.179166
16	6	0	-2.805720	0.302073	1.473385
17	1	0	-3.572048	-0.177903	2.074413
18	1	0	-1.955821	0.505778	2.120203
19	6	0	-3.311648	1.592707	0.830804
20	6	0	-2.985166	1.760205	-0.439987
21	8	0	-1.100751	-1.602795	0.243417
22	1	0	-3.260470	2.634585	-0.996886
23	6	0	-3.774543	-1.609729	-0.425738
24	6	0	-3.546770	-2.488747	-1.680972
25	1	0	-4.580600	-0.902871	-0.597540
26	1	0	-4.069562	-2.247083	0.403601
27	1	0	-4.435223	-3.068523	-1.900319
28	1	0	-3.319832	-1.879614	-2.548296
29	1	0	-2.720124	-3.167354	-1.514073
30	6	0	3.586339	0.480070	-1.743015
31	6	0	3.935624	1.959753	-2.037884
32	1	0	3.325600	-0.014618	-2.675018
33	1	0	4.444186	-0.041598	-1.329516
34	1	0	4.723798	2.020091	-2.778701
35	1	0	3.063419	2.479312	-2.413204
36	1	0	4.276859	2.467813	-1.143050
37	6	0	-4.092195	2.565730	1.672804
38	1	0	-3.496961	2.897032	2.518967
39	1	0	-4.382570	3.436568	1.097455
40	1	0	-4.989965	2.097664	2.066779
41	6	0	4.232822	-0.728673	2.749718
42	1	0	4.504361	-1.747903	2.996970
43	1	0	5.143582	-0.158659	2.588688
44	1	0	3.720686	-0.293895	3.603377

13h:
2353aa_POcpent3Me_Et_dimer_zwitter_kiind_MP2_6311++2d2p_SCANvissza.log

orientation:

Center Number	Atomic Number	Atomic Type	Coordinates (Angstroms) X	Y	Z
1	15	0	-1.896007	0.803515	0.343000
2	6	0	-2.699367	-0.007019	1.771277
3	1	0	-3.357955	0.662037	2.317101
4	1	0	-1.936047	-0.352540	2.465500
5	6	0	-2.135078	-0.552323	-0.779759
6	6	0	-3.159496	-1.439345	-0.128591
7	6	0	-3.445487	-1.180113	1.142028
8	8	0	-0.483702	1.269384	0.540415
9	1	0	-4.122846	-1.767846	1.729672
10	15	0	1.816768	-0.753413	-0.235278
11	6	0	2.064567	-0.211893	1.472589
12	1	0	2.452263	-0.983297	2.125745
13	1	0	1.118728	0.160161	1.847253
14	6	0	2.152497	0.866190	-0.965401
15	1	0	2.604406	0.817279	-1.949207
16	1	0	1.222654	1.422246	-0.998292
17	6	0	3.077017	1.501613	0.077946
18	6	0	3.030144	0.955799	1.282263
19	8	0	0.556791	-1.496405	-0.607864
20	1	0	3.598934	1.322308	2.113432
21	6	0	3.238742	-1.786251	-0.650370
22	6	0	3.129868	-3.204050	-0.034518
23	1	0	4.151099	-1.297849	-0.326469
24	1	0	3.278913	-1.872179	-1.732758
25	1	0	3.953090	-3.820840	-0.373401
26	1	0	3.164203	-3.164002	1.048029
27	1	0	2.199181	-3.668089	-0.332588
28	6	0	-3.006516	2.165721	-0.097114
29	6	0	-2.551176	2.897608	-1.383211
30	1	0	-3.032146	2.873426	0.727414
31	1	0	-4.010766	1.771489	-0.223536
32	1	0	-3.207922	3.732302	-1.597990
33	1	0	-1.542919	3.273376	-1.260706
34	1	0	-2.568063	2.227371	-2.235091
35	6	0	3.905719	2.692916	-0.315942
36	1	0	3.261801	3.503137	-0.644482
37	1	0	4.503390	3.046119	0.515524
38	1	0	4.569756	2.444550	-1.138833
39	6	0	-3.756624	-2.568999	-0.925079
40	1	0	-4.468553	-3.136793	-0.337832
41	1	0	-4.262661	-2.184422	-1.805987
42	1	0	-2.970404	-3.236453	-1.263727
43	1	0	-2.336181	-0.316270	-1.816512
44	1	0	-0.726947	-1.169715	-0.725192

TS(12h->13h):
2353aa_POcpent3Me_Et_dimer_zwitter_kiind_MP2_6311++2d2p_SCANvissza_xyz

Center Number	Atomic Number	Atomic Type	Coordinates (Angstroms) X	Y	Z
P			-2.63782400	0.36275500	0.04436900
C			-2.35393900	0.25843400	1.82340000
H			-3.28195800	0.33882400	2.38585500
H			-1.67944800	1.03807500	2.16410500
C			-2.36015800	-1.27956200	-0.38197200
C			-1.78483500	-1.89599300	0.83324200
C			-1.72750900	-1.14121900	1.93787600
O			-1.37075200	0.87234400	-0.74921000
H			-1.26305500	-1.44175300	2.85424000
P			2.02377300	-0.33654500	-0.54040500
C			2.18707800	-0.28690800	1.27366500
H			2.93380900	-0.97705400	1.65088900
H			1.23243200	-0.55021200	1.72116200
C			1.80012200	1.45918400	-0.72208300
H			2.30527200	1.86271200	-1.59410000
H			0.73787900	1.66922700	-0.80566900
C			2.36125700	2.03419200	0.57613400
C			2.54761900	1.16535000	1.55651300
O			0.99987000	-1.24127100	-1.12063300
H			2.91469300	1.44952400	2.52355100
C			3.69749100	-0.65478500	-1.14814700
C			4.09655900	-2.14452400	-1.00292600
H			4.40501500	-0.01680200	-0.62735800
H			3.72592900	-0.38233500	-2.19991800
H			5.06535600	-2.32051300	-1.45469000
H			4.15497100	-2.43413400	0.04010800
H			3.36401000	-2.77391800	-1.49150200
C			-4.10526600	1.28977700	-0.42753700
C			-4.35830200	1.19648700	-1.95527600
H			-3.98091300	2.33065600	-0.14350000
H			-4.95870300	0.90080400	0.11803800
H			-5.22536700	1.78781100	-2.22134400
H			-3.50207800	1.56837700	-2.50265400
H			-4.54006000	0.17069300	-2.25173000
C			2.62340200	3.51358000	0.66012000
H			1.70806300	4.06829500	0.47599500
H			3.00484000	3.79178700	1.63527200
H			3.34599000	3.81560200	-0.09254800
C			-1.15445100	-3.25679700	0.69914600
H			-0.80298700	-3.63140900	1.65319100
H			-1.86099600	-3.96763700	0.28338600
H			-0.31965500	-3.15643300	0.01444700
H			-2.88876000	-1.87644600	-1.09797800
H			-1.23086100	-0.24876700	-0.97089800

TS(13h->4):
2354aa_POcpent3Me_Et_dimer_zwitter_kiind_MP2_6311++2d2p_TSvissza

Center Number	Atomic Number	Atomic Type	Coordinates (Angstroms) X	Y	Z
P			-2.63782400	0.36275500	0.04436900
C			-2.35393900	0.25843400	1.82340000
H			-3.28195800	0.33882400	2.38585500
H			-1.67944800	1.03807500	2.16410500
C			-2.36015800	-1.27956200	-0.38197200
C			-1.78483500	-1.89599300	0.83324200
C			-1.72750900	-1.14121900	1.93787600
O			-1.37075200	0.87234400	-0.74921000
H			-1.26305500	-1.44175300	2.85424000
P			2.02377300	-0.33654500	-0.54040500
C			2.18707800	-0.28690800	1.27366500
H			2.93380900	-0.97705400	1.65088900
H			1.23243200	-0.55021200	1.72116200
C			1.80012200	1.45918400	-0.72208300
H			2.30527200	1.86271200	-1.59410000
H			0.73787900	1.66922700	-0.80566900
C			2.36125700	2.03419200	0.57613400

C	2.54761900	1.16535000	1.55651300
O	0.99987000	-1.24127100	-1.12063300
H	2.91469300	1.44952400	2.52355100
C	3.69749100	-0.65478500	-1.14814700
C	4.09655900	-2.14452400	-1.00292600
H	4.40501500	-0.01680200	-0.62735800
H	3.72592900	-0.38233500	-2.19991800
H	5.06535600	-2.32051300	-1.45469000
H	4.15497100	-2.43413400	0.04010800
H	3.36401000	-2.77391800	-1.49150200
C	-4.10526600	1.28977700	-0.42753700
C	-4.35830200	1.19648700	-1.95527600
H	-3.98091300	2.33065600	-0.14350000
H	-4.95870300	0.90080400	0.11803800
H	-5.22536700	1.78781100	-2.22134400
H	-3.50207800	1.56837700	-2.50265400
H	-4.54006000	0.17069300	-2.25173000
C	2.62340200	3.51358000	0.66012000
H	1.70806300	4.06829500	0.47599500
H	3.00484000	3.79178700	1.63527200
H	3.34599000	3.81560200	-0.09254800
C	-1.15445100	-3.25679700	0.69914600
H	-0.80298700	-3.63140900	1.65319100
H	-1.86099600	-3.96763700	0.28338600
H	-0.31965500	-3.15643300	0.01444700
H	-2.88876000	-1.87644600	-1.09797800
H	-1.23086100	-0.24876700	-0.97089800

TS(12h->13h):
2358aa_POcpent3Me_Et_dimer_zwitter_kiind_MP2_6311++2d2p_TSelore.log

orientation:

Center Number	Atomic Number	Atomic Type	Coordinates (Angstroms) X	Y	Z
1	15	0	-3.594970	-0.169023	-0.198083
2	6	0	-4.062557	1.268498	-1.155967
3	1	0	-4.860272	1.862092	-0.724887
4	1	0	-4.255688	1.045759	-2.197058
5	6	0	-2.954320	0.734482	1.135939
6	6	0	-2.358529	1.807571	0.506159
7	6	0	-2.626212	1.902862	-0.924288
8	8	0	-2.419705	-0.668323	-1.110369
9	1	0	-2.425821	2.835352	-1.416491
10	15	0	2.406377	0.137593	0.116909
11	6	0	3.317072	-0.743677	1.429648
12	1	0	2.924596	-1.736803	1.617698
13	1	0	3.249425	-0.182385	2.357727
14	6	0	3.901288	0.837961	-0.659157
15	1	0	3.863249	0.805310	-1.743623
16	1	0	4.017957	1.877737	-0.364037
17	6	0	5.048858	-0.006458	-0.111161
18	6	0	4.749547	-0.783242	0.916609
19	8	0	1.353943	1.087597	0.540559
20	1	0	5.473333	-1.410714	1.399141
21	6	0	1.890268	-1.157277	-1.027578
22	6	0	0.698800	-2.008397	-0.516381
23	1	0	2.747399	-1.794184	-1.230566
24	1	0	1.608153	-0.687744	-1.966121
25	1	0	0.538319	-2.843836	-1.187980
26	1	0	0.897554	-2.410579	0.471392
27	1	0	-0.212761	-1.429251	-0.485803
28	6	0	-4.745982	-1.501388	0.112422
29	6	0	-4.072132	-2.671489	0.880241
30	1	0	-5.124177	-1.858840	-0.839611
31	1	0	-5.583877	-1.111301	0.681660
32	1	0	-4.780691	-3.478961	1.014329
33	1	0	-3.221448	-3.043080	0.323929
34	1	0	-3.734547	-2.348417	1.857545
35	6	0	6.408048	0.118753	-0.744997
36	1	0	6.762270	1.143336	-0.679470
37	1	0	7.129889	-0.525736	-0.258278
38	1	0	6.363168	-0.145880	-1.797393
39	6	0	-1.351589	2.698337	1.177429

40	1	0	-0.364907	2.288233	0.985679
41	1	0	-1.400451	3.704968	0.779559
42	1	0	-1.514612	2.728301	2.247864
43	1	0	-2.754697	0.426152	2.138333
44	1	0	-2.041733	0.428514	-1.320955

17a:
313aa_POcpent3_Ph_MsOH_B_MP2_6311++2d2p_PCMthf.log

orientation:

Center Number	Atomic Number	Atomic Type	Coordinates (Angstroms) X	Y	Z
1	15	0	0.943262	-0.523671	-0.000071
2	6	0	0.228795	0.514681	1.367405
3	1	0	0.964326	0.719903	2.151055
4	1	0	-0.600120	-0.039350	1.825246
5	6	0	0.228732	0.514966	-1.367299
6	1	0	0.964229	0.720362	-2.150936
7	1	0	-0.600173	-0.038990	-1.825253
8	6	0	-0.250273	1.767047	-0.672468
9	1	0	-0.566876	2.628654	-1.255305
10	6	0	-0.250218	1.766907	0.672848
11	8	0	0.609853	-1.993259	-0.000227
12	8	0	-3.421770	1.322565	0.001092
13	1	0	-2.448338	1.477405	0.000752
14	8	0	-3.203706	-0.855442	1.259204
15	6	0	-5.479069	-0.219263	0.001546
16	8	0	-3.206241	-0.852902	-1.261867
17	16	0	-3.693031	-0.285897	-0.000270
18	1	0	-5.807880	0.298105	0.902344
19	1	0	-5.809630	0.299965	-0.897542
20	1	0	-5.829558	-1.251848	0.000818
21	6	0	2.746032	-0.226718	-0.000054
22	6	0	3.592313	-1.343169	0.000173
23	6	0	3.302028	1.062633	-0.000220
24	6	0	4.978248	-1.174615	0.000242
25	1	0	3.155427	-2.336545	0.000289
26	6	0	4.686932	1.227295	-0.000157
27	1	0	2.660419	1.939232	-0.000395
28	6	0	5.525848	0.109227	0.000077
29	1	0	5.627876	-2.044708	0.000422
30	1	0	5.111466	2.226698	-0.000293
31	1	0	6.603834	0.240477	0.000127
32	1	0	-0.566753	2.628413	1.255876

18a:
315aa_POcpent3_Ph+H+_MsO_B_MP2_6311++2d2p_PCMthf.log

orientation:

Center Number	Atomic Number	Atomic Type	Coordinates (Angstroms) X	Y	Z
1	15	0	0.810329	0.845731	-0.127256
2	6	0	-0.187534	-0.266883	-1.228383
3	1	0	0.448417	-0.788375	-1.946891
4	1	0	-0.872018	0.368535	-1.792273
5	6	0	-0.010233	0.291202	1.429507
6	1	0	0.668155	0.336431	2.284119
7	1	0	-0.839646	0.980018	1.612941
8	6	0	-0.501108	-1.136998	1.139669
9	1	0	-1.293702	-1.452315	1.820821
10	6	0	-0.963822	-1.262473	-0.334074
11	8	0	0.793951	2.322930	-0.428802
12	8	0	-2.422745	-1.159850	-0.519330
13	8	0	-2.701074	1.353640	-0.275890
14	6	0	-4.779183	-0.229308	-0.879555
15	8	0	-3.589644	-0.182015	1.510203
16	16	0	-3.307202	0.069035	0.093539
17	1	0	-4.533667	-0.099282	-1.932755
18	1	0	-5.131098	-1.240038	-0.675354
19	1	0	-5.516654	0.507516	-0.558409
20	1	0	0.329202	-1.838560	1.273151
21	1	0	-0.809332	-2.285777	-0.678055
22	6	0	2.516861	0.182312	-0.106378
23	6	0	3.556293	1.093773	0.133750
24	6	0	2.828075	-1.172854	-0.297392
25	6	0	4.879911	0.657091	0.192065
26	1	0	3.320205	2.145498	0.259641
27	6	0	4.152833	-1.608200	-0.237754
28	1	0	2.048721	-1.899882	-0.505409
29	6	0	5.179514	-0.694508	0.009126
30	1	0	5.676293	1.372037	0.375448
31	1	0	4.381956	-2.658499	-0.390132
32	1	0	6.209905	-1.034395	0.052433

References

1. Keglevich, G.; Petneházy, I.; Miklós, P.; Almásy, A.; Tóth, G.; Tőke, L.; Quin, L. D. *J. Org. Chem.* **1987**, *52*, 3983–3986.

2. Keglevich, G.; Szelke, H.; Bálint, Á.; Imre, T.; Ludányi, K.; Nagy, Z.; Hanusz, M.; Simon, K.; Harmat, V.; Tőke, L. *Heteroat. Chem.* **2003**, *14*, 443–451.

3. Novák, T.; Ujj, V.; Schindler, J.; Czugler, M.; Kubinyi, M.; Mayer, Z. A.; Fogassy, E.; Keglevich, G. *Tetrahedron: Asymmetry* **2007**, *18*, 2965–2972.

4. Bagi, P.; Kovács, T.; Szilvási, T.; Pongrácz, P.; Kollár, L.; Drahos, L.; Fogassy, E.; Keglevich, G. *J. Organomet. Chem.* **2014**, *751*, 306–313.

5. Coyle, E. E.; Doonan, B. J.; Holohan, A. J.; Walsh, K. A.; Lavigne, F.; Krenske, E. H.; O'Brien, C. J. *Angew. Chem. Int. Ed.* **2014**, *53,* 12907–12911.

6. Shriver, D. F.; Drezdzon, M. A. *The Manipulation of Air-Sensitive Compounds*, Wiley & Sons, New York, **1986**.

7. Armarego, W. L. F. *Purification of Laboratory Chemicals*, Butterworth-Heinemann, Oxford, **2017**.

8. Pedersen, D.; Rosenbohm, C. *Synthesis* **2004**, 2431–2434.

9. Yamada, M.; Yamashita, M.; Suyama, T.; Yamashita, J.; Asai, K.; Niimi, T.; Ozaki, N.; Fujie, M.; Maddali, K.; Nakamura, S. et al., *Bioorg. Med. Chem. Lett.* **2010**, *20,* 5943–5946.

10. Magiera, D.; Moeller, S.; Drzazga, Z.; Pakulski, Z.; Pietrusiewicz, K. M.; Duddeck, H. *Chirality* **2003**, *15,* 391–399.

11. Thiele, C. M.; Petzold, K.; Schleucher, J. *Chem. Eur. J.* **2009***, 15*, 585–588.

12. Frisch, M. J.; Trucks, G. W.; Schlegel, H. B.; Scuseria, G. E.; Robb, M. A.; Cheeseman, J. R.; Scalmani, G.; Barone, V.; Mennucci, B.; Petersson, G. A.; Nakatsuji, H.; Caricato, M.; Li, X.; Hratchian, H. P.; Izmaylov, A. F.; Bloino, J.; Zheng, G.; Sonnenberg, J. L.; Hada, M.; Ehara, M.; Toyota, K.; Fukuda, R.; Hasegawa, J.; Ishida, M.; Nakajima, T.; Honda, Y.; Kitao, O.; Nakai, H.; Vreven, T.; Montgomery, J. A., Jr.; Peralta, J. E.; Ogliaro, F.; Bearpark, M.; Heyd, J. J.; Brothers, E.; Kudin, K. N.; Staroverov, V. N.; Kobayashi, R.; Normand, J.; Raghavachari, K.; Rendell, A.; Burant, J. C.; Iyengar, S. S.; Tomasi, J.; Cossi, M.; Rega, N.; Millam, J. M.; Klene, M.; Knox, J. E.; Cross, J. B.; Bakken, V.; Adamo, C.; Jaramillo, J.; Gomperts, R.; Stratmann, R. E.; Yazyev, O.; Austin, A. J.; Cammi, R.; Pomelli, C.; Ochterski, J. W.; Martin, R. L.; Morokuma, K.; Zakrzewski, V. G.; Voth, G. A.; Salvador, P.; Dannenberg, J. J.; Dapprich, S.; Daniels, A. D.; Farkas, O.; Foresman, J. B.; Ortiz, J. V.; Cioslowski, J.; Fox, D. J. Gaussian 09, Revision A.1, Gaussian, Inc., Wallingford, CT, 2009.

13. Mucsi, Z. ; Chass, G. A; Viskolcz, B.; Csizmadia I. G. *J. Phys. Chem. A* **2009**, *113*, 7953-7962.

14. Mucsi, Z.; Porcs-Makkay, M.; Simig, G.; Csizmadia, I. G.; Volk, B. *J. Org. Chem*. **2012**, *77*, 7282-7290.

15. Kondacs, L. A.; Pilipecz, M. V.; Mucsi, Z.; Balazs, B.; Gati, T.; Nyerges, M.; Dancso, A.; Nemes, P. *Eur. J. Org. Chem.* **2015**, *31*, 6872-6890.
